# Postdiagenetic Bacterial Transformation of Nickel and Vanadyl Sedimentary Porphyrins of Organic-Rich Shale Rock (Fore-Sudetic Monocline, Poland)

**DOI:** 10.3389/fmicb.2021.772007

**Published:** 2021-11-30

**Authors:** Robert Stasiuk, Renata Matlakowska

**Affiliations:** Department of Geomicrobiology, Institute of Microbiology, Faculty of Biology, University of Warsaw, Warsaw, Poland

**Keywords:** geoporphyrins, *Pseudomonas*, pyrrole, shale rock, mass spectrometry, atomic emission spectrometry

## Abstract

Nickel and vanadyl porphyrins belong to the so-called fossil geo- or sedimentary porphyrins. They occur in different types of organic matter-rich sediments but mostly occur in crude oils and their source rocks, oil shales, coals, and oil sands. In this study, we aimed to understand the process of bacterial transformation of geoporphyrins occurring in the subsurface shale rock (Fore-Sudetic Monocline, SW Poland). We studied these transformations in rock samples directly obtained from the field; in rock samples treated with bacterial strain isolated from shale rock (strain LM27) in the laboratory; and using synthetic nickel and vanadyl porphyrins treated with LM27. Our results demonstrate the following: (i) cleavage and/or degradation of aliphatic and aromatic substituents of porphyrins; (ii) degradation of porphyrin (tetrapyrrole) ring; (iii) formation of organic compounds containing 1, 2, or 3 pyrrole rings; (iv) formation of nickel- or vanadium-containing organic compounds; and (v) mobilization of nickel and vanadium. Our results also showed that the described bacterial processes change the composition and content of geoporphyrins, composition of extractable organic matter, as well as nickel and vanadium content in shale rock.

## Introduction

Tetrapyrrole pigments such as chlorophyll and bacteriochlorophylls, as well as heme, are the most abundant and evolutionarily amongst the oldest pigments on the earth (Gueneli et al., 2018). These compounds are the biological precursors of sedimentary porphyrins, which are also called geoporphyrins or petroporphyrins. These compounds occur in fossil fuels and asphalts as well as in oil shales and their associated source rocks dominantly as nickel(II) and vanadyl (oxovanadium(IV); VO(IV)) metal complexes. Since the discovery of geoporphyrins in the 1930s, researchers have paid much attention to develop methods for the extraction, identification, and characterization of the structure of geoporphyrins, as well as to explain their origin and evolution during diagenesis (Hodgson, 1973; Filby and Van Berkel, 1987; Callot et al., 1990; Callot and Ocampo, 2000). One of the major fields of research in geochemistry is related to the transformation of geoporphyrins, both under biotic and abiotic conditions, that take place after diagenesis. Several researchers have shown that biodegradation had a little impact on geoporphyrins distribution in the highly biodegraded tar-sand bitumens from the Ardmore and Anadarko Basins (Oklahoma, United States), oils and tars from the Permian Phosphoria Formation (Wyoming, United States), and Alberta oil sand (Canada) (Barwise and Park, 1983; Palmer, 1983; Strong and Filby, 1987; Lin et al., 1989; Sundararaman and Hwang, 1993). It was believed that due to the processes of primary demetallization and secondary metallization with nickel or vanadium, geoporphyrins are much more stable than that of their precursors. However, other studies have shown some different data regarding the chemical weathering of geoporphyrins. For example, according to Riley and Saxby (1982), the destruction of porphyrins by weathering had led to the release of vanadium from the organic matter in the Toolebuc Formation oil shale in Australia. Grosjean et al. (2004) showed substantial depletion of geoporphyrins during degradation of a Lower Toarcian shale of the Paris Basin in France. Interestingly, these authors did not take biological transformation into account, although the presence of microorganisms in the tested samples was not excluded.

It is noteworthy that the aforementioned processes of biodegradation of geoporphyrins were studied in deposits that underwent the process of catagenesis. The process of catagenesis also releases geoporphyrins from kerogens (Barwise and Roberts, 1984). At the same time, we must also consider the fact that in oil shale or bitumens, many carbon-containing organic compounds are more readily available to microorganisms and are more easily degradable than that of porphyrins, such as aliphatic and aromatic hydrocarbons. This might explain the lack of biodegradation of geoporphyrins in these types of deposits. On the contrary, in shale rocks, organic carbon dominates in the form of complex macromolecular kerogen. The role of bacteria in the processes of transformation of geoporphyrins occurring in kerogen-bearing shale rocks is still unknown.

In this study, we aimed to determine the role of bacteria in the transformation of fossil nickel and vanadyl geoporphyrins occurring in the shale rock located in Fore-Sudetic Monocline in SW Poland. It is a Lopingian sedimentary rock of marine origin containing large amounts of organic carbon (up to 16 wt%) which is represented by kerogen type II. Previous research has shown that this shale rock contains nickel and vanadium porphyrins (Tokarska, 1971; Sawłowicz, 1985; Szubert et al., 2006). So far, we have shown that the community of microorganisms inhabiting the shale rocks were responsible for the oxidation and dehydrogenation of kerogen and for the mobilization of fossil organic carbon in the oxidized form (Stasiuk et al., 2017; Włodarczyk et al., 2018).

Further we investigated whether these processes affect the distribution and/or transformation of nickel and vanadyl geoporphyrins, and how these porphyrin transformations is affecting are potentially linked with the composition of the extractable organic matter.

To answer these questions, we divided this study into two parts. In the first part, we analyzed the transformation of fossil geoporphyrins by using two field samples collected from the Lubin underground copper mine. The first sample (SR) was shale rock taken from a freshly exposed rock profile in which microorganisms were not detected (Stasiuk et al., 2017). The second sample was a bacteria-inhabited shale rock (BISR) collected from the profile created 12 years ago (Włodarczyk et al., 2018). In the second part of our study, SR was exposed to the bacterium *Pseudomonas* sp. LM27 isolated from BISR for 30 days (SR–BC), and the obtained results were compared with SR incubated in sterile mineral medium (sterile control; SR–SC). In addition, series of experiments were conducted using three synthetic nickel and vanadyl porphyrins with aliphatic and aromatic substituents that were treated with the *Pseudomonas* sp. strain LM27 (bacterial culture, BC) and incubated as mentioned previously in a sterile medium (sterile control, SC).

*Pseudomonas* sp. strain LM27 was isolated from BISR and showed 99% similarity to *Pseudomonas stutzeri* (Stasiuk and Matlakowska, under review). It was decided to use this strain in the presented research due to the dominance of *P. stutzeri* in the rock microbiocenosis (Włodarczyk et al., 2018).

The bacterial transformations of nickel and vanadyl fossil geoporphyrins were further studied by comparing the content of porphyrins and potential products of their transformation in the samples of shale rock inhabited by microorganisms (BISR and SR-BC) to uninhabited (SR and SR-SC), including both natural (SR and BISR) and laboratory conditions (BC and SC). Analogous comparisons were made for the growth of strain LM27 on synthetic porphyrins (BCs) and SCs.

We hypothesized that nickel and vanadyl geoporphyrin biological transformations include cleavage/degradation of aliphatic or aromatic substituents and cleavage/degradation of the tetrapyrrole ring. The first process could be confirmed by detecting the substituents and/or products of substitutes transformation, such as organic compounds containing phenyl, butyl, and ethyl groups and their derivatives, as well as by detecting porphyrins without substituents or with a reduced number of substituents.

The cleavage/degradation of the tetrapyrrole ring of porphyrins could be detected by the changes in the absorption spectrum of the studied samples, as well as by the presence of specific organic compounds, such as pyrrole rings containing organic compounds or vanadium-, nickel-, and/or nitrogen-containing organic compounds. We also hypothesized that geoporphyrin transformations can lead to changes in the content of nickel and vanadium in the studied shale rocks or aqueous phase of the BC. Due to the complexity of the studied shale rocks samples (both SR and BISR), we did not carry out the detection of organic compounds derived from geoporphyrins substituents. However, they were analyzed only in experiments with synthetic porphyrins. Moreover, isolation and analysis of bacterial (meta)proteomes were performed to identify enzymes that are potentially responsible for the bacterial processes.

## Materials and Methods

### Field Samples

Shale rock and BISR were respectively collected from a freshly exposed outcrop (2 days) and from a 12-year-old outcrop at a depth of 770 m in Lubin copper mine, which is located in Lubińsko-Głogowski Copper District (SW Poland). All samples were aseptically collected (from outcrops to a depth of 10 cm) and were stored at –80°C or –4° until further use. The geochemical characteristics of the samples are presented in our previous publication (Włodarczyk et al., 2018).

### Synthetic Porphyrins

Three synthetic porphyrins were used in the laboratory experiments: 2,3,7,8,12,13,17,18-octaethyl-21H23H-porphine nickel(II) (henceforth referred to as Ni(OEP)); 2,3,7,8, 12,13,17,18-octaethyl-21H23H-porphine vanadium(IV) oxide (henceforth referred to as VO(OEP)); and 5,10,15,20-tetraphenyl-21H23H-porphine vanadium(IV) oxide (vanadyl(IV) *meso*-tetraphenylporphine) (henceforth referred to as VO(MTPP)) (Frontiers Specialty Chemicals, United States). Table 1 and Supplementary Material A (Supplementary Figures A.1–A.8) present a detailed description of these porphyrins.

**TABLE 1:** List of porphyrins and other organic compounds studied in this work.

Name	Symbol	Chemical formula	Specific ion *m/z*
**Porphyrins**			
Meso-tetraphenyl vanadyl porphyrin*	VO(MTPP)	C_44_H_28_N_4_VO	679
Meso-tetraphenyl nickel porphyrin	Ni(MTPP)	C_44_H_28_N_4_Ni	670
Octaethyl vanadyl porphyrin*	VO(OEP)	C_36_H_44_N_4_VO	599
Octaethyl nickel porphyrin*	Ni(OEP)	C_36_H_44_N_4_Ni	591
Diphenyl vanadyl porphyrin	VO(DPP)	C_32_H_38_N_4_VO	528
Tetraethyl nickel porphyrin	Ni(TEP)	C_28_H_29_N_4_Ni	481
Tetraethyl vanadyl porphyrin	VO(TEP)	C_28_H_29_N_4_VO	472
Nickel porphyrin	NiP	C_20_H_15_N_4_Ni	368
Vanadyl porphyrin	VOP	C_20_H_12_N_4_VO	361
**Other organic compounds**			
Organic compounds containing ethyl group			45
Organic compounds containing 1 pyrrole ring			67
Organic compounds containing phenyl group			77
Butanoic acid			88
Butanedioic acid			118
Organic compounds containing 2 pyrrole rings			134
Organic compounds containing 3 pyrrole rings			201

*porphyrin used in laboratory experiments.

### Bacteria

Strain LM27 was isolated from BISR using procedures in Stasiuk and Matlakowska (under review).

### Culture Media

Bacteria were grown in lysogeny broth (LB), LB agar (Sambrook and Russell, 2001), or in a modified mineral salts medium (MBS, prepared by dissolving 800 mg of K_2_HPO_4_ and 200 mg of KH_2_PO_4_ × 2H_2_O in a liter of demineralized water, pH 7.0 (Hartmans et al., 1989)) supplemented with 10 mM glucose.

### Experiment on Biodegradation of Geoporphyrins

Modified MBS medium (500 ml) was supplemented with 50 g of SR. The SR was crushed to increase the surface area for the exposure of factors (bacteria, medium, and oxygen); the fragments were produced with a diameter ranging 0.125–0.25 mm, 0.25–0.5 mm, 0.5–1.0 mm, and 1.0–2.0 mm. Next, the crushed SR was mixed and pasteurized by heating thrice for 1 h at 100°C, on 3 successive days. This method of sterilization was previously confirmed to minimize the degradation of fossil organic matter (data not shown).

The SR-BCs were prepared from the bacteria growing in the exponential phase in LB medium and harvested by centrifugation (8000 × *g*, 4°C, 10 min). The pellet was washed thrice in MBS medium (to remove traces of LB medium), resuspended, and used to inoculate the MBS medium. The final number of bacterial cells in the inoculated MBS medium was 1.5 × 10^6^ cells/ml. All bacterial experiments were conducted in duplicate and grown under static aerobic conditions at 22°C for 30 days in darkness. Sterile MBS medium with SR was used as the chemical control. Bacterial growth was estimated once every 5 days by plating culture dilutions on solid LB medium to determine the number of colony-forming units (CFUs/ml). After 30 days, BCs and control samples were centrifuged (8000 × *g*, 4°C, 10 min) and the obtained aqueous phases were filtered through a Whatman filter paper with a 0.22 μm pore size to obtain cell-free samples.

### Biodegradation Experiment of Synthetic Porphyrins

To study the biodegradation of synthetic porphyrins, we supplemented MBS medium with glucose with one of the synthetic porphyrins (0.5 mM) as a powder which was suspended in the medium. Then, the medium was inoculated and the incubation was carried out as described in subsection “Experiment on Biodegradation of Geoporphyrins.”

### Microscopic Observations

Microscopic observations were performed using a Nikon Eclipse 80i microscope (Nikon Corporation, Japan) equipped with NIS Elements Basic research v.2.3 software (Nikon). For scanning electron microscopy, the samples were fixed in paraformaldehyde vapor, gold-coated, and observed under Leo 1430VP, LEO Electron Microscopy Inc., United States.

### Extraction of Organic Compounds From the Aqueous Phase

Organic compounds were extracted from 100 ml of the cell-free aqueous phase of the BCs and SCs samples in 25 ml of chloroform in a separatory funnel for 3 min. This procedure was repeated thrice. Chloroform extracts were pooled and dried with anhydrous Na_2_SO_4_, and the solvent was evaporated under the N_2_ stream. Samples were then derivatized with 0.5 ml of N,O-Bis(trimethylsilyl)trifluoroacetamide with 1% Trimethylchlorosilane (Supelco, United States) for 30 min at 70°C. A blank sample was prepared according to the same procedure.

### Extraction of Organic Compounds From Solid Samples

Shale rock, BISR and residues after the laboratory experiment, were dried at 60°C and powdered. Organic compounds were extracted from 20 g of samples by a mixture of dichloromethane/methanol (vol. ratio 9:1) for 4 h using an automatic Soxhlet apparatus SER 158 (Velp, Italy). Asphaltenes were precipitated with hexane, next the solvent was evaporated under N_2_ stream, and the samples were derivatized with 0.5 ml of BSTFA:TMCS, 99:1 (Supelco, United States) for 30 min at 70°C. A blank sample was prepared according to the same procedure.

### Gas Chromatography With Mass Spectrometry

The separation of organic compounds was performed using an Agilent 7890A Series Gas Chromatograph interfaced to an Agilent 5973c Network Mass Selective Detector and an Agilent 7683 Series Injector (Agilent Technologies, United States). A 5 μL sample was injected with a split ratio of 1:5 by 0.3% standard deviation (SD) to an HP-5MS column (30 m × 0.25 mm I.D., 0.25 μm film thickness, Agilent Technologies, United States) using helium as the carrier gas at 1 ml/min. The sample was excited into a gaseous state at a temperature of 400°C. The ion source was maintained at 250°C; the GC oven was programmed with a temperature gradient starting at 100°C (for 3 min), which was gradually increased to 300°C (for 5 min) at 8°C/min.

Mass spectrometry was carried out in the electron-impact mode at an ionizing potential of 70 eV. Mass spectra were recorded from the mass-to-charge (*m*/*z*) ratio in the range of 40–800 (0–30 min) by applying the selected ion monitoring (SIM) method. Table 1 presents the list of analyzed ions.

Identification of the organic compounds containing pyrrole ring, ethyl, butyl and phenyl group were performed with an Agilent Technologies Enhanced ChemStation (G1701EA ver. E.02.00.493) and The Wiley Registry of Mass Spectral Data (version 3.2, Copyright 1988–2000 by the Palisade Corporation with, 8th Edition with Structures, Copyright 2000 by John Wiley and Sons, Inc.) using a 3% cutoff threshold. The results of the analysis were statistically evaluated using Student’s *t*-test. The mass spectra of identified compounds are presented in Supplementary Material I (Supplementary Figures I.1–I.30).

### Gas Chromatography With Atomic Emission Detector

Nickel-, vanadium-, and/or nitrogen-containing organic compounds were detected by GC (GC 7890A, Agilent Technologies, United States) coupled with AED (JAS G2350A, Germany). The samples (5 μL) were manually injected by using a 10 μL syringe. Each sample was injected thrice. The injected sample was gaseous at 350°C and diluted with a helium stream with a split ratio of 1:5 v/v. The organic compounds were separated using an HP-5MS column (30 m, 0.25 mm I.D., 0.25 μm particle size (Agilent Technologies, United States)) using helium as the carrier gas (1 ml/min). The temperature program was set at three levels. The sample was held at 180°C for 1 min, followed by an increase in temperature to 300°C at a rate of 8°C/min. The final temperature was maintained at 300°C for 3 min. The total analysis time for a single sample was 20.7 min.

The AED detector uses helium plasma and detection of the element lines C (496 nm), H (486 nm), Ni (301 nm), V (292 nm), and N (174 nm). The number of H atoms in the compounds detected was not calculated. The temperature of the transfer line was 300°C. The flows of the remaining reaction gases (oxygen, a mixture of carbon dioxide with methane (86%:14%), and hydrogen) necessary for the determination of the relevant elements were determined according to standard settings recommended by the manufacturer.

The chemical formula (C, Ni, V, and N) of the detected organic compounds were determined using the Joint Analytical System software (D.02.01 Version 486; JAS, Germany) based on the peak area corresponding to the respective detection lines of the elements tested in relation to the standards, which were chloroform solution of 0.1 mM synthetic nickel and vanadyl porphyrins. The results of the analysis were statistically evaluated using Student’s *t*-test.

### Gas Chromatography With Flame Ionization Detector

Qualitative and quantitative analysis of pyrrole was performed using a 7890 A gas chromatograph (Agilent Technologies, United States) equipped with FID using helium as the carrier gas. The operating parameters of the detector were as follows: flow rate of nitrogen, hydrogen, and air was 25 ml/min, 30 ml/min, and 200 ml/min, respectively. The temperature of the injector port and the detector was 280°C and 300°C, respectively. The analysis was conducted by using the same chromatographic conditions (column temperature program, split, injection volume, etc.) as the GC-MS.

Prior to the analysis, calibration standards were run to check the performance of the column, peak height and resolution, and the limits of detection. With each set of the samples to be analyzed, a solvent blank, a standard mixture, and procedural blank were run in sequence to check for contamination, peak identification, and quantification. Supplementary Figure A.9D (Supplementary Material A) presents the calibration curve for pyrrole. The results of the analysis were statistically evaluated using Student’s *t*-test.

### High-Performance Liquid Chromatography With Photodiode Array Detector

The quantitative analysis of porphyrins was performed using HPLC (Separation module 2695, Waters Alliance, United States) equipped with a PAD (2996, Waters Alliance, United States). An inverted C18 (reverse phase) Hypersil ion exchange column (150 mm × 2.1 mm length × width, 5 μm particle size) was used. During the chromatographic separation, 5 μL of the sample was applied to the column. Samples were eluted isocratically with a 1:4 (v/v) mixture of acetone and methanol. The flow rate of the mobile phase was set at 0.8 ml/min. The samples and column were heated to 30°C. The detector scanned the adsorption spectrum of the sample in the range of 200–700 nm, and the analysis time for a single sample was 10 min. Porphyrins were detected at 425 nm.

The concentration of porphyrins was determined based on the standard curves (0.0675, 0.125, 0.25, 0.50, 0.75, and 1.00 mM). Concentration curves were plotted based on the peak areas corresponding to the metalloporphyrin tested (Supplementary Material A Figure A.7). The results of the analysis were statistically evaluated using Student’s *t*-test.

### Atomic Absorption Spectrometry

The concentration of nickel and vanadium in studied samples were determined by conducting atomic absorption spectrometry using Solar M6 spectrometer (TJA Solution) and by following the manufacturer’s instructions. All experiments were performed in triplicate. Samples were prepared by digestion with 65% nitric acid.

### Isolation of Proteins

Proteins were extracted based on the modified procedure of Ram et al. (2005). Briefly, 10 g of rock sample and 0.5 g of bacterial biomass was resuspended in 120 ml of 20 mM Tris–HCl (pH 8), shaken for 3 min, and sonicated on ice for up to 10 times for 1 min, with 1-min pauses (Sonics Vibracell; LABOPLUS, ModelCV18head). Next, 100 ml of 0.4 M Na_2_CO_3_ (pH 11) was added to the suspension of the lysed cells, and the suspension was centrifuged at 6000 × *g* for 20 min at 4°C to remove the unlysed cells and fragments of the cell membrane. Then, the supernatant was filtered through filter paper with a pore size of 0.22 μm. Next, the proteins from the solution were precipitated with trichloroacetic acid (TCA) at a ratio of 1:10 (v/v). The mixture was allowed to precipitate overnight at 4°C and then centrifuged at 20,000 × *g* for 10 min at 4°C. The aqueous phase was discarded and the protein pellet was resuspended in 0.5 ml of methanol precooled to 4°C and centrifuged at 20,000 × *g* for 10 min at 4°C. The resulting precipitate was dried and stored at −80°C. The analysis was performed in triplicate.

### Identification of Proteins

The proteins were identified by liquid chromatography coupled to tandem mass spectrometry (LC-MS-MS/MS) using a Nano-Acquity (Waters) LC system and Orbitrap Velos mass spectrometer (Thermo Electron Corp., San Jose, CA, United States). The experiment was conducted at the Environmental Laboratory of Mass Spectrometry, Institute of Biophysics and Biochemistry (Polish Academy of Sciences, Warsaw, Poland).

Prior to the analysis, proteins were subjected to a standard “in-solution digestion” procedure during which proteins were reduced with 50 mM Tris(2-carboxyethyl)phosphine (for 60 min at 60°C), alkylated with 200 mM *S*-methyl methanethiosulfonate (45 min at room temperature), and digested overnight with trypsin (Sequencing Grade Modified Trypsin; Promega V5111).

The peptide mixture was applied to an RP-18 precolumn (nanoACQUITY Symmetry^®^ C18; Waters 186003514) using water containing 0.1% trifluoroacetic acid as the mobile phase and then transferred to a nano-HPLC RP-18 column (nanoACQUITY BEH C18; Waters 186003545) using an acetonitrile (ACN) gradient (5–35% ACN in 180 min) in the presence of 0.05% formic acid with a flow rate of 250 μL/min. The column outlet was directly coupled to the ion source of the spectrometer working in the regime of data-dependent MS to MS/MS switch. A blank run ensuring a lack of cross-contamination from previous samples preceded each analysis.

Acquired raw data were processed by Mascot Distiller followed by Mascot Search (Matrix Science, London, United Kingdom, on-site license) against the NCBI protein database. Peptides with a Mascot score exceeding a threshold value corresponding to <5% expectation value, calculated by Mascot procedure, were considered to have been positively identified.

## Results and Discussion

### Detection of Nickel and Vanadyl Sedimentary Porphyrins in the Shale Rock – Field Studies

**FIGURE 1:**
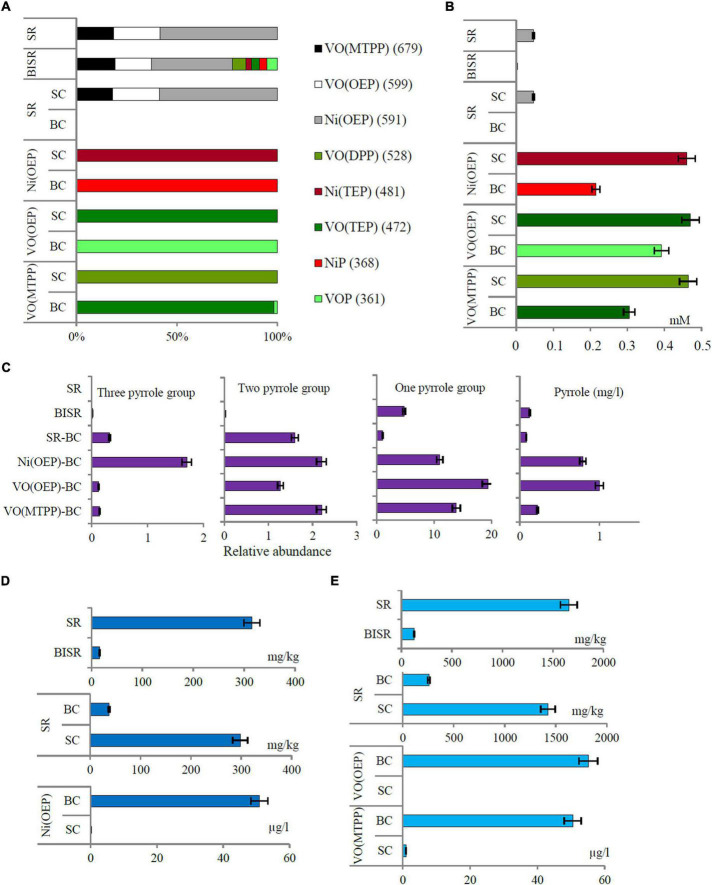
**(A)** The percentage distribution of porphyrins with and without substituents in the studied samples based on the relative content of specific ions; **(B)** the content of porphyrins in the shale sample; **(C)** relative abundance of organic compounds containing 1, 2, and 3 pyrrole rings and the concentration of pyrrole in the studied sample; **(D)** the concentration of nickel and **(E)** vanadium in shale rock (SR), bacteria-inhabited shale rock (BISR), and aqueous phases of bacterial culture and sterile control of SR and synthetic porphyrins (SR-BC/SC, VO(OEP)-BC/SC, and VO(MTPP)-BC/SC). All differences are statistically significant (significance level: *p* < 0.05).

Three mass ions *m/z:* 679, 599, and 591 specific for VO(MTPP), VO(OEP), and Ni(OEP), respectively, were identified in the SR sample (Table 2 and Supplementary Material B Figure B.1). Their relative content was respectively calculated at 8978; 11114; and 28198 raw detector signal in peak area (Figure 1A). No specific ions were identified for the other porphyrins tested, with or without substituents. Two organic compounds of the HPLC retention time (RT) of 1.20 min and 1.48 min (425 nm) were identified in the SR sample (Table 2 and Supplementary Material B Figure B.2). These compounds showed an intense Soret band with maximum absorption at 380 nm and 401 nm and two smaller Q bands at 516/549 nm, and 529/571 nm, respectively. Based on the RTs, these compounds were identified as nickel porphyrin (RT = 1.20 min) and vanadyl porphyrin (RT = 1.48 min). However, taking the spectra of synthetic porphyrins into account, as well as the results of porphyrin-specific ion analysis in the SR sample, it can be assumed that the second compound with an RT of 1.48 can be a mixture of two vanadyl porphyrins (VO(OEP) and VO(MTPP)). The concentration of these compounds estimated on the basis of the standard curve (Supplementary Material A Figure A.7) was 0.034 mM and 0.013 mM, respectively (Figure 1B).

**TABLE 2:** Summary of chemical characteristics of shale rock (SR), bacteria-inhabited shale rock (BISR), bacterial culture (SR-BC), and sterile control (SR-SC).

Analysis	Field samples		Laboratory studies	
	SR	BISR	SR-BC	SR-SC
		**Sedimentary porphyrins – monitoring of specific ion**		
SIM	VO(MTPP), VO(OEP), Ni(OEP)	VO(MTPP), VO(OEP), Ni(OEP), VO(DPP), Ni(TEP), VO(TEP), NiP, VOP	-	VO(MTPP), VO(OEP), Ni(OEP)
		**Organic compounds containing porphyrin ring – detection of specific absorption maxima UV-VIS**		
Soret band	380, 401	420	-	380, 401
Q band	516/549, 529/571	538/576	-	516/549, 529/571
		**Organic compounds containing pyrrole ring – detection of specific ion**		
3 pyrrole rings (*m/z:* 201)	-	3-[2-[[3-(2-Carboxyethyl)-5-[(3.4-dimethyl-5-oxopyrrol-2-ylidene)methyl]-4-methyl-1H-pyrrol-2-yl]methylidene]-4-methyl-5-oxopyrrol-3-yl]propanoic acid 3-[(5Z)-5-[[4-Ethenyl-5-[(Z)-(4-ethenyl-3-methyl-5-oxopyrrol-2-ylidene)methyl]-3-methyl-1H-pyrrol-2-yl]methylidene]-4-methyl-2-oxopyrrol-3-yl]propanoate	3-[(5Z)-5-[[4-Ethenyl-5-[(Z)-(4-ethenyl-3-methyl-5-oxopyrrol-2-ylidene)methyl]-3-methyl-1H-pyrrol-2-yl]methylidene]-4-methyl-2-oxopyrrol-3-yl]propanoate	-
2 pyrrole rings (*m/z:* 134)	-	3,3′-Bipyrrole, 2,2′-Bipyrrole, 1,1′-Bipyrrole-2,2′,5,5′-tetraone	3,3′-Bipyrrole, 2,2′-Bipyrrole, 3,3′,4,4′-Tetramethyl-1H,1′H-2,2′-bipyrrole-5,5′-dicarboxylic acid	-
1 pyrrole ring (*m/z:* 67)	-	1H-Pyrrole, Indole acetic acid, Indole carbaldehyde	1H-Pyrrole-2-carboxylic acid Indole acetic acid 1H-Indole-2-carboxylic acid Indole carbaldehyde 2H-Pyrrol-2-one, 5-[[2-[(4-aminophenyl)methylene]-3,4-dimethyll]methylene]-3-ethyl-1,5-dihydro- 4-methyl	-
		**Organic compound containing nickel, vanadium and/or nitrogen**		
Nickel	C_31_H_x_N_4_Ni	C_4_H_x_NNi, C_14_H_x_N_2_Ni	C_8_H_x_N_2_Ni, C_10_H_x_Ni, C_12_H_x_N_2_Ni, C_14_H_x_NNi, C_16_H_x_NNi, C_18_H_x_NNi	C_31_H_x_N_4_Ni
Vanadium	C_44_H_x_N_4_V	C_4_H_x_V, C_6_H_x_N_2_V, C_8_H_x_N_2_V, C_24_H_x_N_4_V, C_44_H_x_N_4_V	C_4_H_x_V, C_4_H_x_NV, C_5_H_x_NV, C_8_H_x_N_2_V, C_12_H_x_N_2_V, C_18_H_x_N_2_V	C_44_H_x_N_4_V

The following organic compounds were detected in the SR sample and their molecular formula was determined based on the GC-AED analysis: C_31_H_x_N_4_Ni and C_44_H_x_N_4_V (Table 2 and Supplementary Material B Figure B.3). On comparison with their emission spectra, they were found to be nickel and vanadyl porphyrin. The SR sample did not show the presence of non-porphyrin organic compounds containing pyrrole rings as well as no other organic compounds containing nickel, vanadium, and/or nitrogen (Figure 1C, Table 2, and Supplementary Material B Figure B.4). The average concentration of nickel and vanadium in the SR sample was 315 mg/kg and 1658 mg/kg, respectively (Figures 1D,E).

The geochemical characterization of the SR sample made as part of our earlier studies has shown that SR of the area of Fore-Sudetic Monocline is typically characterized by kerogen type II and contains extractable organic matter at a level of ∼2% (w/w) (Włodarczyk et al., 2018).

### Detection of Transformation of Nickel and Vanadyl Sedimentary Porphyrins in Bacteria-Inhabited Shale Rock – Field Studies

In the BISR sample, mass ions specific for the three doped tested nickel and vanadyl porphyrins were identified (Figure 1A, Table 2, and Supplementary Material C Figure C.1). However, their content was significantly lower than that in the SR sample, which was estimated at 4818; 4501; and 10081 raw detector signal in peak area for VO(MTPP), VO(OEP), and Ni(OEP), respectively (Figure 1A and Table 2). Moreover, one organic compound with an RT of 1.48 min (425 nm) was identified in the BISR sample (Table 2 and Supplementary Material C Figure C.2) as vanadyl porphyrin. Its concentration was 0.0013 mM, which was ∼32% of the its content in SR (Figure 1B). Analysis of the UV-Vis absorption spectra of the BISR sample showed a decrease in both bands as well as a shift to longer wavelengths (420 nm for Soret band and 538/576 for Q bands, respectively) (Supplementary Material C Figure C.2C). These changes may confirm cleavage and/or partial degradation of tetrapyrrole ring as well as indicate the disconnection of metal from the tetrapyrrole ring.

In contrast to SR, in the BISR sample, we identified two nickel-containing organic compounds (C_4_H_x_NNi and C_14_H_x_N_2_Ni) and five vanadium-containing organic compounds (C_4_H_x_V, C_6_H_x_N_2_V, C_8_H_x_N_2_V, C_24_H_x_N_4_V, and C_44_H_x_N_4_V) (Table 2 and Supplementary Material C Figure C.3).

In contrast to SR, in the BISR sample, we detected organic compounds containing 1, 2, or 3 pyrrole rings. Table 2 and Supplementary Figures C.4–C.6 (Supplementary Material C) present the SIM chromatograms and list of proposed names of the compounds detected. The content of pyrrole in the BISR sample estimated on the basis of the standard curve (Supplementary Material A Figure A.9D) was 0.0125 mg/kg (Figure 1C).

In the BISR sample, we detected two organic compounds containing 3 pyrrole rings: 3-[(5Z)-5-[[4-ethenyl-5-[(Z)-(4-ethenyl-3-methyl-5-oxopyrrol-2-ylidene) methyl]-3-methyl-1H-pyrrol-2-yl] methylidene]-4-methyl-2-oxopyrrol-3-yl] propanoate (C_24_H_24_N_3_O_4_) and 3-[2-[[3- (2-carboxyethyl)-5-[(3.4-dimethyl-5-oxopyrrol-2-ylidene) methyl]-4-methyl-1H-pyrrol-2-yl] methylidene]-4-methyl-5-oxopyrrol-3-yl] (propanoic acid) (Supplementary Materials C, I Figures C.4, I.1, I.2). These compounds accounted for ∼0.015% of all compounds (Figure 1C). The first of these compounds was identified among the degradation products of VO(OEP) and VO(MTPP) as well as of Ni(OEP) by the strain LM27 (Tables 3–5). This compound was also detected as the product of an alternative heme degradation reaction by *Escherichia coli* O157: H7 (Quellet et al., 2016). The second of the compounds containing 3 pyrrole rings was identified as a product of bacterial degradation of Ni(OEP) (Table 3). In addition, organic compounds containing two pyrrole rings—2,2′-bipyrrole and 3,3′-bipyrrole (C_8_H_6_N_2_), as well as 1,1′-bipyrrole-2,2′,5,5′-tetraone (C_8_H_4_N_2_O_4_), were detected in the BISR sample (Table 2 and Supplementary Materials C, I Figures C.5, I.3–I.5). These compounds accounted for ∼0.02% of all organic compounds identified in this sample (Figure 1C). Bipyrroles have also been identified as the degradation products of Ni(OEP), VO(OEP), and VO(MTPP) by the strain LM27 (Tables 3–5). In addition, we detected compounds containing a single pyrrole ring in the BISR sample, such as 1H-pyrrole (C_4_H_5_N), indole acetic acid (C_10_H_9_NO_2_), and indole carboxaldehyde (C_9_H_7_NO) (Supplementary Materials C, I Figures C.6, I.6 – I.8). These compounds constitutes a significant ∼4.79% of all organic compounds identified in this sample (Figure 1C). All the aforementioned compounds containing a single pyrrole ring have been identified among the degradation products of the aforementioned synthetic vanadyl and nickel porphyrins (Tables 3–5). It is noteworthy that the derivatives of pyrrole—maleimides (1H-pyrrole-2,5 diones)—are considered as the degradation products of tetrapyrrole pigments such as bacteriochlorophylls *c, d*, and *e* synthesized by green sulfur bacteria (*Chlorobiaceae*) (Grice et al., 1996, 1997; Naeher and Grice, 2015).

**TABLE 3:** Summary of chemical characteristics of bacterial culture and sterile control on Ni(OEP) after 30 days of the experiment.

Ni(OEP)	Bacterial culture	Sterile control
**Specific ion**		
Ni(OEP)	NiP	Ni(TEP)
**Soret band**		
380	405	380
**Q band**		
516/549	-	516/549
**Organic compounds containing ethyl group (*m/z:* 45)**		
-	Ethanimidic acid, Ethanoic acid, Ethanedioic acid, Ethane-2,2-diol	Ethanimidic acid
**Organic compounds containing 3 pyrrole rings (*m/z:* 201)**		
-	3-[2-[[3-(2-Carboxyethyl)-5-[(3,4-dimethyl-5-oxopyrrol-2-ylidene)methyl]-4-methyl-1H-pyrrol-2-yl]methylidene]-4-methyl-5-oxopyrrol-3-yl]propanoic acid 3-[(5Z)-5-[[4-Ethenyl-5-[(Z)-(4-ethenyl-3-methyl-5-oxopyrrol-2-ylidene)methyl]-3-methyl-1H-pyrrol-2-yl]methylidene]-4-methyl-2-oxopyrrol-3-	-
**Organic compounds containing 2 pyrrole rings (*m/z:* 134)**		
-	3,3′-Bipyrrole, 2,2′-Bipyrrole 1,1′-Bipyrrole-2,2′,5,5′-tetraone 3,3′,4,4′-Tetramethyl-1H,1′H-2,2′-bipyrrole-5,5′-dicarboxylic acid	-
**Organic compounds containing 1 pyrrole ring (*m/z:* 67)**		
-	1H-Pyrrole Indole acetic acid	-
**Organic compounds containing nickel**		
C_36_H_44_N_4_Ni	C_20_H_x_N_4_Ni, C_15_H_x_NNi, C_10_H_x_N_2_Ni, C_5_H_x_N_2_Ni, C_5_H_x_NNi	C_28_H_x_N_4_Ni

**TABLE 4:** Summary of chemical characteristics of bacterial culture and sterile control on VO(OEP) after 30 days of the experiment.

VO(OEP)	Bacterial culture	Sterile control
**Specific ion**		
VO(OEP)	VOP	VO(TEP)
**Soret band**		
401	407	401
**Q band**		
529/571	532/575	529/571
**Organic compounds containing ethyl group (*m/z:* 45)**		
-	Ethanimidic acid, Ethanedioic acid, Ethane-2,2-diol	Ethanimidic acid
**Organic compounds containing 3 pyrrole rings (*m/z:* 201)**		
-	3-[(5Z)-5-[[4-Ethenyl-5-[(Z)-(4-ethenyl-3-methyl-5-oxopyrrol-2-ylidene)methyl]-3-methyl-1H-pyrrol-2-yl]methylidene]-4-methyl-2-oxopyrrol-3-yl]propanoate	-
**Organic compounds containing 2 pyrrole rings (*m/z:* 134)**		
-	3,3′-Bipyrrole, 2,2′-Bipyrrole, 3,3′,4,4′-Tetramethyl-1H,1′H-2,2′-bipyrrole-5,5′-dicarboxylic acid 2H-Pyrrol-2-one, 5-[[2-[(4 aminophenyl)methylene]-3,4-dimethyl-2H pyrrol-5-yl]methylene]-3-ethyl-1,5-dihydro-4- methyl-, (Z,Z)	-
**Organic compounds containing 1 pyrrole ring (*m/z:* 67)**		
-	1H-Pyrrole, 2-Pentanone, 5-(5-methyl-4-cyclononen-1-ylidene), 1H-Pyrrole-2-carboxylic acid, 1-Methylpyrroline, Methyl 3-((Z)-2-bromobut-2-en-1-yl)-2,3,3A,4,5,7-hexahydro-4-hydroxy-1H-pyrrolo[2,3-D]carbazole-6-carboxylate, Indole acetic acid, Indole carbaldehyde, 2H-Pyrrol-2-one, 5-[[2-[(4-aminophenyl)methylene]-3,4-dimethyll]methylene]-3-ethyl-1,5-dihydro-4-methyl	-
**Organic compounds containing vanadium**		
C_36_H_44_N_4_VO	C_20_H_x_N_4_V, C_12_H_x_V, C_7_H_x_V, C_5_H_x_N_2_V, C_4_H_x_V	C_28_H_x_N_4_V

**TABLE 5:** Summary of chemical characteristics of bacterial culture and sterile control on VO(MTPP) after 30 days of the experiment.

VO(MTPP)	Bacterial culture	Sterile control
**Specific ion**		
VO(MTPP)	VO(TEP), VOP	VO(DPP)
**Soret band**		
401	419	401
**Q band**		
529/571	547/584	529/571
**Organic compounds containing phenyl group (*m/z:* 77)**		
-	1,2-Benzenediol, Pentafluorobenzaaldehyde, Butylphenol	Benzenol
**Organic compounds containing butyl group**		
-	Butanoic acid, Butanedioic acid, Butylphenol	-
**Organic compounds containing ethyl group (*m/z:* 45)**		
-	Ethanimidic acid, Ethanedioic acid	-
**Organic compounds containing 3 pyrrole rings (*m/z:* 201)**		
-	3-[(5Z)-5-[[4-Ethenyl-5-[(Z)-(4-ethenyl-3-methyl-5-oxopyrrol-2-ylidene)methyl]-3-methyl-1H-pyrrol-2-yl]methylidene]-4-methyl-2-oxopyrrol-3-yl]propanoate	-
**Organic compounds containing 2 pyrrole rings (*m/z:* 134)**		
-	3,3′-Bipyrrole, 2,2′-Bipyrrole, 1,1′-Bipyrrole-2,2′,5,5′-tetraone, 3,3′,4,4′-Tetramethyl-1H,1′H-2,2′-bipyrrole-5,5′-dicarboxylic acid	-
**Organic compounds containing 1 pyrrole ring (*m/z:* 67)**		
-	1H-Indene, 2,3-dihydro-1,1,5-trimethyl, 1H-Indene, 2,3-dihydro-1,4,7-trimethyl-1H- Inden-1-one, 2,3-dihydro-3,3,5,7-tetramethyl, Indole acetic acid, Indole carbaldehyde, Piperidine, 1-(5-trifluoromethyl-2-pyridyl)-4-(1H-pyrrol-1-yl), 2-[1-(4-Chlorobenzoyl)-5-methoxy-2-methylindol-3-yl]acetic acid, 2H-Pyrrol-2-one, 5-[[2-[(4-aminophenyl)methylene]-3,4-dimethyll]methylene]-3-ethyl-1,5-dihydro-4-methyl	-
**Organic compounds containing vanadium**		
C_44_H_x_N_4_VO	C_28_H_x_N_4_V, C_25_H_x_N_2_V, C_20_H_x_N_2_V, C_10_H_x_N_2_V, C_8_H_x_N_2_V, C_3_H_x_NV, C_5_H_x_N_2_V	C_32_H_x_N_4_V

The average concentration of nickel and vanadium in the BISR sample was much lower than that in the SR sample, which amounted to 16 mg/kg and 126 mg/kg, respectively (Figures 1D,E).

The geochemical characterization of the BISR sample made as part of our earlier studies has shown an unusually high ratio of oxygen to hydrogen compared to that of the SR sample, which shows that kerogens are strongly oxidized (Włodarczyk et al., 2018). Simultaneously, we observed a considerably reduced (∼86%) content of organic carbon dominated by residual carbon (91%). Similarly, reduced content of free hydrocarbons (∼96%) and hydrocarbon potential (∼91%) confirmed the dehydrogenation of kerogen. All these parameters suggest that kerogen of BISR was transformed from oil-prone type II to a non-productive, residual, and hydrogen-free kerogen type IV.

Furthermore, BISR sample compared to the SR sample showed a higher extractable yield and a higher content of aromatic hydrocarbons and their derivatives relative to the aliphatic hydrocarbons. This might be related to the bioweathering of kerogen and the partial biodegradation of the primary aliphatic hydrocarbons. About 45% of all the extractable organic compounds identified in the BISR sample were oxygenated alcohols, aldehydes, ketones, esters, and above all carboxylic acids. In the SR sample, the content of oxidized organic compounds was estimated to be around 8–10% (Włodarczyk et al., 2018).

The kerogen and bitumen transformations described above have been attributed to the activity of microorganisms that inhabit BISR. Among them, 23 bacterial phyla (78%) were identified (Włodarczyk et al., 2018). Bacteria were dominated by *Proteobacteria* (55%) mostly represented by γ-Proteobacteria class (29%). *Pseudomonadaceae* (20%) was the most abundant family, and it was dominated by the *P. stutzeri* group (14%). Metagenome analysis allowed the identification of 5748 bacterial genes potentially involved in the oxidative metabolism of aromatic and aliphatic hydrocarbons, as well as the metabolism of alcohols, aldehydes, and ketones. Among these, bacterial mono- and dioxygenases (2373 unique reads (URs)), aldehyde dehydrogenases (1106 URs), and alcohol dehydrogenases (912 URs) were dominant. A considerable number of identified proteins encoded genes potentially involved in the metabolism of hydrocarbons were derived from *Pseudomonas* spp. (451 URs).

### Bacterial Transformation of Nickel and Vanadyl Sedimentary Porphyrins by LM27 – Laboratory Studies

The strain LM27 was able to grow on a mineral medium with SR (SR-BC) as the only carbon and energy source. During the 30-day experiment, there was an increase in the number of CFUs/ml from an initial value of 10^6^ CFUs/ml to ∼2 × 10^7^ CFUs/ml on day 25 of the experiment (Supplementary Material D Figure D.1).

In the SR-BC sample, no specific ions were identified for any of the porphyrins tested (Figures 1A,B, Table 2, and Supplementary Material D Figure D.2) and no compounds containing the tetrapyrrole ring were identified (Supplementary Material D Figure D.3). However, it showed the presence of one compound containing three pyrrole rings: 3-[(5Z)-5-[[4-Ethenyl-5-[(Z)-(4-ethenyl-3-methyl-5-oxopyrrol-2-ylidene)methyl]-3-methyl-1H-pyrrol-2-yl]methylidene]-4-methyl-2-oxopyrrol-3-yl]propanoate, which accounted for ∼0.32% of all compounds detected in this sample (Figure 1A and Supplementary Materials D, I Figures D.5, I.1). This compound was also detected in the BISR sample but was not detected in SR and SR-SC sample. In addition, three compounds containing 2 pyrrole rings, which constituted ∼1.6% of all compounds, were detected in the SR-BC sample (Figure 1C and Supplementary Material D Figure D.6). Among them, 2,2′-bipyrrole and 3,3′-bipyrrole were identified, which were also identified in the BISR sample (Table 2 and Supplementary Material I Figures I.3, I.4). Moreover, 3,3′, 4,4′-tetramethyl-1H, 1′H-2,2′-bipyrrole-5,5′-dicarboxylic acid was detected in the SR-BC sample (Supplementary Material I Figure I.9). All these compounds were identified among the degradation products of the aforementioned synthetic vanadyl and nickel porphyrins (Tables 3–5). In addition, five single-pyrrole compounds such as indole acetic acid (C_10_H_9_NO_2_); indole carbaldehyde (C_9_H_7_NO); 1H-indole-2-carboxylic acid (C_9_H_7_NO_2_); 1H-pyrrole-2-carboxylic acid (C_5_H_5_NO_2_); and 2H-pyrrol-2-one, 5-[[2-[(4-aminophenyl) methylene]-3,4-dimethyll] methylene]-3-ethyl-1,5-dihydro-4-methyl (C_21_H_23_N_3_O) have been identified (Supplementary Materials D, I Figures D.7, I.7, I.8, I.10–I.12). These compounds accounted for ∼1.05% of all compounds detected in this sample (Figure 1C). The concentration of pyrrole in the SR-BC sample accounted for 0.082 mg/kg (Figure 1C). Indole carbaldehyde was also found in the BISR sample, and indole acetic acid was identified in both BISR and BC with three synthetic porphyrins (Tables 2–5).

Two nickel-containing (C_10_H_x_Ni and C_12_H_x_N_2_Ni) and four vanadium-containing organic compounds (C_4_H_x_NV, C_5_H_x_NV, C_8_H_x_N_2_V, and C_12_H_x_N_2_V) were found in the aqueous phase of SR-BC sample (Table 2 and Supplementary Material D Figure D3). Moreover, four nickel-containing (C_8_H_x_N_2_Ni, C_14_H_x_NNi, C_16_H_x_NNi, and C_18_H_x_NNi), as well as elemental nickel, and two vanadium-containing organic compounds (C_4_H_x_V and C_18_H_x_N_2_V) were identified in the culture sediment (Supplementary Material D Figure D.4). Two of the aforementioned compounds were also detected in the BISR sample (C_4_H_x_V and C_8_H_x_N_2_V). The organic compounds containing four nitrogen atoms were not detected, but the organic compound containing a single nitrogen atom (C_8_H_x_N) was detected. The average concentration of nickel and vanadium in the SR-BC sample was 315 mg/kg and 1658 mg/kg, respectively (Table 2).

Analysis of the SR-SC sample showed that abiotic processes are of minor importance in geoporphyrin transformations. Three mass ions—679, 599, and 591—specific for VO(MTPP), VO(OEP), and Ni(OEP) were identified in the SR-SC sample (Figure 1A, Table 2, and Supplementary Material D Figure D.8). Their relative content was very similar to the SR sample (Figure 1B). No specific ions were identified in other porphyrins tested.

Similar to the SR sample, two organic compounds with an RT of 1.20 min and 1.48 min (425 nm) were also identified in the SR-SC sample (Table 2 and Supplementary Material D Figure D.9B). These compounds showed an intense Soret band with maximum absorption at 380 nm and 401 nm and two smaller Q bands at 529/571 nm and 510/549 nm, respectively (Supplementary Material D Figure D.9C). Based on the RTs, these compounds were identified as nickel geoporphyrin (RT = 1.20 min) and vanadyl geoporphyrin (RT = 1.48 min). The concentration of these compounds was analogous to that of the SR sample (Figure 1B).

GC-AED analysis confirmed the presence of geoporphyrins in the SR-SC sample with the same formula as that obtained for SR sample (C_31_H_x_N_4_Ni and C_44_H_x_N_4_V) and confirmed the absence of other nickel- and vanadium-containing organic compounds (Table 2 and Supplementary Material D Figure D.10). Only two nitrogen-containing organic compounds were identified—C_19_H_x_N and C_8_H_x_N. The concentration of nickel and vanadium in the SR-SC sample was 298 mg/kg and 1423 mg/kg, respectively (Figures 1D,E). No organic compounds containing pyrrole rings were detected in the SR-SC sample (Supplementary Material D Figure D.11).

Supplementary Material H presents the results of the analysis of the strain LM27 culture in MBS-BC and MBS-SC samples. We did not detect the presence of any pyrrole-, nickel-, or vanadium-containing organic compounds in these two samples.

### Bacterial Transformation of Ni(OEP) and VO(OEP) by LM27

Supplementary Figures E.1A, F.1A (Supplementary Materials E, F) present the growth curves of LM27 on mineral medium with Ni(OEP) and VO(OEP) during 30 days of the experiment. Both these compounds stimulated the growth of LM27. The duplication time was ∼1.8 and ∼3 times shorter and the maximal number of cells after 30 days of cultivation was ∼1.6 and ∼7 times higher in the Ni(OEP)-BC and VO(OEP)-BC, respectively, than those in culture without porphyrins (Supplementary Material H Figure H.1). Based on the light and scanning electron microscopy, BCs of both Ni(OEP) and VO(OEP) revealed the formation of a thin biofilm on the surface (Supplementary Materials E, F Figures E1.B, F1.B). Supplementary Figures E.2, F.2 (Supplementary Materials E, F) show an image of the porphyrins before the bacterial colonization.

According to our hypothesis, the first stage of transformation of Ni(OEP) and VO(OEP) is the cleavage and/or degradation of its substituents. The cleavage of the aliphatic substituents from Ni(OEP) by the strain LM27 is evidenced by the presence of an organic compound with the chemical formula C_20_H_x_N_4_Ni in the Ni(OEP)-BC resulting from the cleavage of 16 carbon atoms, which may correspond to 8 ethyl groups (Table 3 and Supplementary Material E Figure E.3). This compound is a porphyrin without any substituents. In the Ni(OEP)-SC, we detected an organic compound with the chemical formula C_28_H_x_N_4_Ni resulting from the cleavage of 8 carbon atoms, which may correspond to 4 ethyl groups (Supplementary Material E Figure E.10). This compound is a porphyrin still with some aliphatic substituents. The presence of compounds with the aforementioned chemical formulas was confirmed by monitoring the specific ions (*m/z:* 368 and 481, respectively) (Figures 1A, Table 3, and Supplementary Material E Figures E.4, E.11).

Similarly, in the case of VO(OEP)-BC, the cleavage of aliphatic substituents by the strain LM27 is evidenced by the presence of an organic compound with the chemical formula C_20_H_x_N_4_V resulting from the cleavage of 16 carbon atoms, which may correspond to 8 ethyl groups (Table 4 and Supplementary Material F Figure F.3). This compound is a porphyrin without any substituents. The analysis of the VO(OEP)-SC confirmed the presence of an organic compound with the chemical formula C_28_H_x_N_4_V resulting from the cleavage of 8 carbon atoms, which may correspond to 4 ethyl groups (Table 4 and Supplementary Material F Figure F.10). This compound is a porphyrin with some aliphatic substituents. The presence of compounds with the aforementioned formulas was confirmed by monitoring the selected specific ions (*m/z:* 361 and 472, respectively) (Figure 1A, Table 4, and Supplementary Material F Figures F.4, F.11).

**FIGURE 2:**
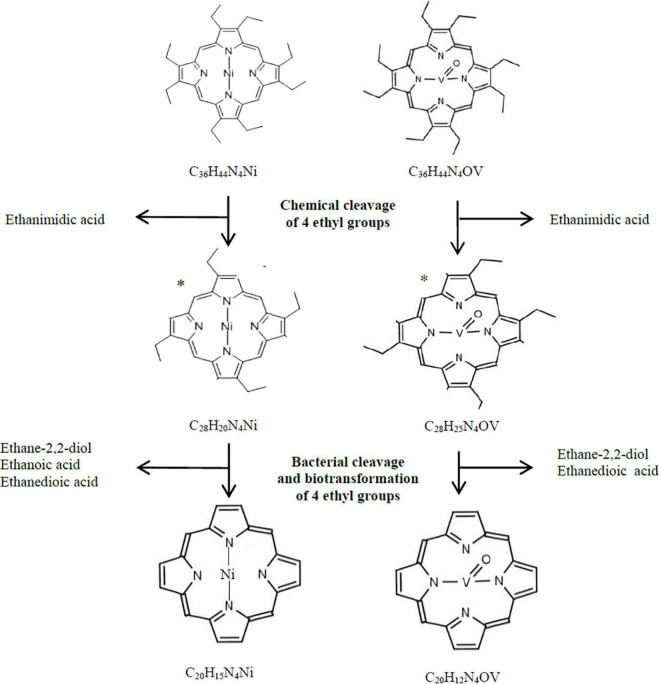
Hypothetical bacterial and chemical transformation of Ni(OEP) and VO(OEP) to nickel and vanadyl porphines. *, hypothetical structural formula.

In the SCs sample, the chemical cleavage of four ethyl groups was detected by the presence of ethanimidic acid (Tables 3, 4 and Supplementary Materials E, F Figures E.12, F.12). In the both BCs, this process could additionally be combined with the degradation of the other 4 ethyl groups. In the extract of both cultures, different 4 organic compounds containing ethyl group were detected, among them (1) ethane-1,2-diol, which may have arisen as a result of terminal oxidation by monooxygenase; (2) ethanedioic acid and (3) 2-hydroxyethanoic acid, which may have arisen from ethane-1,2-diol, with the participation of alcohol and aldehyde dehydrogenases (Tables 3, 4 and Supplementary Materials E, F, I Figures E.5, F.5, I.13–I.16). The presence of these enzymes was confirmed in the proteome of the strain LM27 cultured on the medium with VO(OEP) (Table 6). Figure 2 shows the proposed potential transformation of Ni(OEP) and VO(OEP) leading to the formation of NiP and VOP.

**TABLE 6:** Enzymes detected in bacteria-inhabited shale rock (BISR) and cultures of LM27 on shale rock (SR-BC), synthetic porphyrins (Ni(OEP)-, VO(OEP)-, VO(MTPP)-BC), and mineral medium with glucose (MBS-BC) of potential importance in porphyrins biotransformation (Supplementary results are presented in Supplementary Material J).

Enzyme	BISR	SR-BC	Ni(OEP)-BC	VO(OEP)-BC	VO(MTPP)-BC	MBS-BC
Monooxygenases	gi| 517722695 gi| 306921967	gi| 489174927 WP_108997997.1	gi| 489174927	gi| 489174927	gi| 489174927 WP_123314309.1 WP_108997997.1	gi| 489174927
Dioxygenases	gi| 490244293 gi| 496990711 gi| 490758414	gi| 119225932 gi| 504664205 gi| 119225932	gi| 119225932 gi| 493449991 gi| 504664205	gi| 119225932 gi| 493449991 gi| 489379953 gi| 504664205 gi| 489386821	WP_018343223.1 gi| 119225932 gi| 493449991 gi| 551285924 gi| 489386821 gi| 504664205	gi| 119225932 gi| 504664205 gi| 493449991
Alcohol dehydrogenases	gi| 446065336 gi| 491126456 gi| 499688088 gi| 654498023 gi| 517727322 gi| 481050357 gi| 663398328 gi| 663143141	gi| 497365707 gi| 489376667 WP_041105468.1	gi| 497365707 gi| 504634675 gi| 489376667	gi| 497365707 gi| 500251385 gi| 489376667	gi| 497365707 gi| 489376667 gi| 496697725 WP_041105468.1 WP_100535563.1	gi| 497365707 gi| 489376667
Aldehyde dehydrogenase	gi| 490789497 gi| 491126465 gi| 491118422 gi| 493682336 gi| 500262322 gi| 489377479	gi| 497821071 gi| 489976938 gi| 54022357 gi| 499520282 gi| 502838067 gi| 489387324 WP_102651941.1 WP_089390756.1	gi| 497227518 gi| 497821071 gi| 489976938 gi| 54022357 gi| 499520282 gi| 639919105 gi| 655195683 gi| 695879780 gi| 502838067 gi| 489391996 gi| 489387324	gi| 61105804 gi| 522201862 gi| 497821071 gi| 653101528 gi| 489976938 gi| 54022357 gi| 499520282 gi| 739160356 gi| 495843407 gi| 494507689 gi| 502838067 gi| 489387324 gi| 739194103	gi| 497821071 gi| 489976938 gi| 54022357 gi| 499520282 gi| 489376354 gi| 502838067 gi| 489387324 WP_089390756.1 WP_041108853.1 WP_041107939.1 WP_065832814.1 WP_052964493.1 WP_054092922.1 WP_017939693.1 WP_085021235.1 WP_111086861.1 WP_028473261.1	gi| 497821071 gi|489976938 gi| 54022357 gi| 499520282 gi| 502838067 gi| 489387324

The next stage of the research was confirmation of the biodegradation of tetrapyrrole ring. The HPLC-PDA analysis of the BCs showed a decrease in the area of absorbance maximum of Ni(OEP) and VO(OEP) after 30 days of the experiment (Tables 3, 4 and Supplementary Materials E, F Figures E.6B, F.6B). The decrease was calculated as ∼57% (0.215 mM) and ∼21.6% (0.392 mM) for Ni(OEP) and VO(OEP), respectively. In the SCs, the decrease was at the level ∼8% (0.46 mM) and ∼6% (0.47 mM) for Ni(OEP) and VO(OEP), respectively (Figure 1B).

Ni(OEP) and VO(OEP) showed the presence of an intense Soret band with maximum absorption at 380 nm and 401 nm as well as two smaller Q bands at 516/549 and 529/571 nm (Tables 3, 4 and Supplementary Materials E, F Figures E.6C, F.6C). Analysis of the UV-Vis absorption spectra of the BCs after 30 days of the experiment showed a decrease as well as a shift to longer wavelengths of Soret band (405/407 nm), complete quenching of the Q band in the case of Ni(OEP), and decrease and shift of Q band for VO(OEP) (532/575 nm). These changes confirmed the partial biodegradation of tetrapyrrole ring as well as confirmed the mobilization of nickel/vanadium (Figures 1B,D,E).

In the case of Ni(OEP)-BC and VO(OEP)-BC, we detected the presence of 7 and 13 organic compounds, respectively, containing 1, 2, and 3 pyrrole rings (Tables 3, 4). The SIM chromatograms, list of the proposed names of detected compounds are respectively presented in Supplementary Materials E, F, I (Figures E.7–E.9, F.7–F.9, I.1–I.9, I.11, I.12, I.18–I.20). One of the detected compounds present in both cultures was 3-[(5Z)-5-[[4-ethenyl-5-[(Z)-(4-ethenyl-3-methyl-5-oxopyrrol-2-ylidene) methyl]-3-methyl-1H-pyrrol-2-yl] methylidene]-4-methyl-2-oxopyrrol-3-yl] propanoate (Supplementary Material I Figures I.1 and I.2), which has also been identified in the BISR and SR-BC samples (Table 2). In addition, compounds containing two and one pyrrole ring were also identified in both Ni(OEP)-BC and VO(OEP)-BC, which were also detected in the BISR and SR-BC samples (Supplementary Material I Figures I.3–I.9, I.11, I.12, I.18–I.20). The contribution of organic compounds containing 1, 2, and 3 pyrrole rings was estimated to be respectively around 11%, 2.2%, and 1.7% for Ni(OEP)-BC and 19.4%, 1.27%, and 0.12% for VO(OEP)-BC. The concentration of pyrrole in Ni(OEP)-BC and VO(OEP)-BC samples was 0.79 mg/kg and 0.99 mg/kg, respectively (Figure 1C).

Nickel and vanadium-containing organic compounds were identified both in the cell-free aqueous phase (soluble) as well as in sediment (insoluble) of Ni(OEP)-BC and VO(OEP)-BC. In the case of Ni(OEP)-BC sample, we detected the presence of three nickel-containing organic compounds (C_5_H_x_NNi, C_5_H_x_N_2_Ni, and C_10_H_x_N_2_Ni) in the aqueous phase and one in sediment (C_15_H_3_NNi) (Table 3 and Supplementary Material E Figure E.3). In the case of VO(OEP)-BC sample, we detected the presence of three vanadium-containing organic compounds (C_4_H_x_V, C_5_H_x_N_2_V, and C_7_H_x_V) in the aqueous phase and one in sediment (C_12_H_x_V) (Table 4 and Supplementary Material F Figure F.3). Two organic compounds containing 4 nitrogen atoms identified in the sediments of Ni(OEP)-BC and VO(OEP)-BC samples have been potentially described as nickel porphyrin (C_20_H_x_N_4_Ni) and vanadyl (C_20_H_x_N_4_V) resulting from the cleavage or breakdown of the substituents of Ni(OEP) and VO(OEP), respectively. In addition to nickel- and vanadium-containing organic compounds, we also detected the presence of two nitrogen atoms-containing compounds. It is possible that these compounds were formed as a result of the breakdown of the tetrapyrrole ring.

Next, we determined the concentrations of nickel and vanadium in the aqueous phase of the LM27 culture media after 30 days of the experiment (Figures 1D,E), which was around 50.9 and 55.2 μg/L for Ni(OEP)-BC and VO(OEP)-BC, respectively. The presence of nickel and vanadium in the culture media confirms the mobilization of these elements and/or soluble metal-containing compounds from porphyrins by the strain LM27.

### Bacterial Transformation of VO(MTPP) by LM27

Supplementary Figure G.1A (Supplementary Material G) presents the growth curve of LM27 in mineral medium with VO(MTPP) during 30 days of the experiment, which shows that VO(MTPP) stimulated the growth of strain LM27. The duplication time was ∼3.5 times shorter and the maximal number of cells after 30 days of cultivation was ∼12 times higher in the VO(MTPP)-BC than that in the culture without porphyrin (MBS-BC) (Supplementary Materials G, H Figures G.1B, H.1B). The observation of BCs by light and scanning electron microscopy revealed that the cells formed a biofilm on the VO(MTPP) (Supplementary Material G Figure G.2).

Cleavage and degradation of aromatic substituents (i.e., phenyl rings) were confirmed by the presence of compounds with chemical formula C_28_H_x_N_4_V and C_20_H_x_N_2_V in the VO(MTPP)-BC sample (Table 5 and Supplementary Material G Figure G.3). These two identified organic compounds were formed by the cleavage of 16 and 24 carbon atoms, respectively. The presence of these compounds was further confirmed by the identification of the *m*/*z:* 472 and 361 ions in the tested samples, respectively (Figure 1A, Table 5, and Supplementary Figure G.4). Considering the chemical formula of these compounds, we can say that the first of them is vanadyl porphyrin having substituents of the summary formula C_8_H_14_ (VO(TEP)) and the second one is a vanadyl porphyrin without any substituents (VOP).

The analysis of VO(MTPP)-SC confirmed the presence of an organic compound with the chemical formula C_32_H_x_N_4_V resulting from the cleavage of 12 carbon atoms (Table 5 and Supplementary Material G Figure G.13). Based on the sum formula, it can be assumed that this compound is vanadyl porphyrin having substituents of the formula C_12_Hx, which can be confirmed by identification of the *m*/*z:* 528 ion in the tested extracts (Figure 1A and Supplementary Material G Figure G.14). It can be assumed that the 12 carbon atoms cleaved belong to two phenyl groups (C_6_H_5_), and therefore the compound with 32 carbon atoms may belong to diphenyl vanadyl porphine (VO(DPP)). The cleavage of two phenyl groups is indicated by the presence of phenyl derivative – benzenol (phenol) in the SC (Table 5 and Supplementary Materials G, I Figures G.15, I.21), which is detected by ion *m/z:* 77 specific for phenyl group.

**FIGURE 3:**
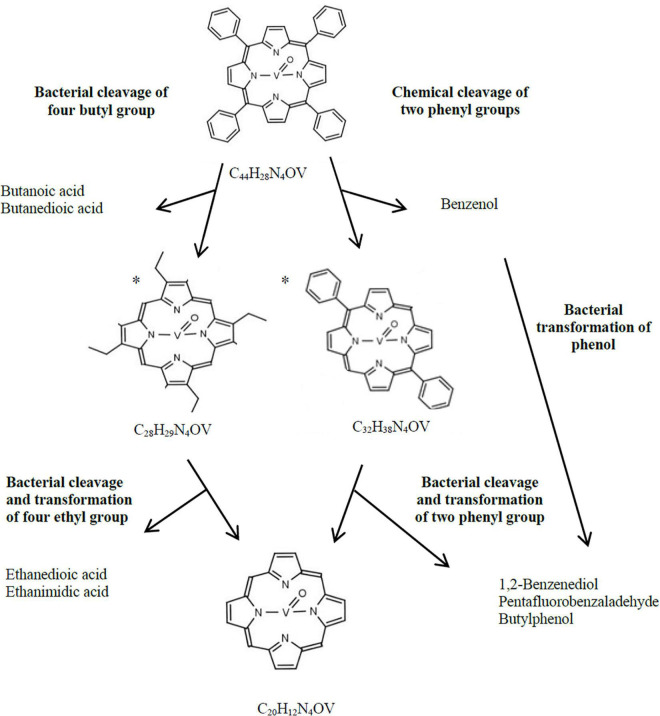
Hypothetical bacterial and chemical transformation of VO(MTPP) to vanadyl porphine. *, hypothetical structural formula.

In the case of BC, the abiotic process of cleavage of two phenyl rings could additionally be combined with the enzymatic degradation of the other two phenyl rings or cleavage of four phenyl groups first to ethyl groups resulting in a compound identified as C28. The cleavage of substituents was confirmed by the presence of compounds containing phenyl groups in the BC (ion *m*/*z:* 77), such as 1,2-benzenediol (C_6_H_6_O_2_), pentafluorobenzaldehyde (C_6_F_5_CHO) and buthylphenol (C_10_H_14_O) (Table 5 and Supplementary Materials G, I Figures G.5, I.22–I.24). Moreover, the enzymatic degradation of phenyl groups and the cleavage of four carbon atoms was confirmed by the presence of C4 compounds in the chloroform extract of the BC (Table 5 and Supplementary Material G Figures G.6, G.7), which were found to be butane derivatives—butanoic acid (C_4_H_8_O_2_) and butanedioic acid (C_4_H_6_O_4_) detected by the monitoring of ions *m*/*z:* 88 and *m*/*z:* 118. These compounds were not detected in the SC sample (Supplementary Materials G, I Figures G.16, I.25, I.26). Another confirmation of the aforementioned transformations leading to the formation of VOP is the presence of ethanedioic acid and ethanimidic acid only in VO(MTPP)-BC, which might be formed as a result of cleavage of ethyl groups from VO(TEP) (Supplementary Material I Figures I.13, I.15). Figure 3 shows the proposed potential pathway for abiotic and biotic VO(MTPP) transformations leading to the formation of VOP.

The HPLC-PDA analysis of VO(MTPP)-BC showed a decrease in the area of absorbance maximum of VO(MTPP) after 30 days of the experiment (Supplementary Material G Figure G.9B), which was calculated to be ∼39% (0.305 mM) (Figure 1B). Analysis of the UV-Vis absorption spectra of the sediment of the BC after 30 days showed a decrease in both bands as well as shift to longer wavelengths (419 nm for Soret band and 547/584 for Q bands) (Table 5 and Supplementary Material G Figure G.9C). These changes confirmed the partial biodegradation of tetrapyrrole ring as well as indicate the cleavage of vanadium from the tetrapyrrole ring.

The degradation of the tetrapyrrole ring was also confirmed by the detection of compounds containing 1–3 pyrrole rings in the BC, as well as vanadium-containing organic compounds, and the mobilization of vanadium to the aqueous phase in BC (Table 5).

An organic compound containing 3 pyrrole rings identified in VO(MTPP)-BC was 3-[(5Z)-5-[[4-ethenyl-5-[(Z)-(4-ethenyl-3-methyl-5-oxopyrrol-2-ylidene)methyl]-3-methyl-1H-pyrrol-2-yl] methylidene]-4-methyl-2-oxopyrrol-3-yl]propanoate (C_24_H_24_N_3_O_4_). Its relative contribution from the total content was calculated to be ∼0.14% (Figure 1C and Supplementary Materials G, I Figures G.10, I.1). This compound was also detected in all the tested BCs as well as in the BISR sample (Tables 2–4).

Among the organic compounds containing 2 pyrrole rings were bipyrrole (C_8_H_6_N_2_); 1,1′-bipyrrole-2,2′, 5,5′-tetraone (C_8_H_4_N_2_O_4_); and 3,3′, 4,4′-tetramethyl-1H, 1′H-2,2′-bipyrrole-5,5′-dicarboxylic acid (C_14_H_16_N_2_O_4_) (Table 5 and Supplementary Materials G, I Figures G.11, I.3–I.5, I.9). These compounds constituted ∼2.2% of all the identified compounds in this sample (Figure 1C).

Among the pyrrole-containing organic compounds, the maximum belonged to monopyrroles (Table 5). Three derivatives of polycyclic aromatic hydrocarbon indene found in VO(MTPP)-BC were detected: 1H-indene—2,3-dihydro-1,1,5-trimethyl (C_12_H_16_); 1H-indene—2,3-dihydro-1,4,7-trimethyl- (C_12_H_16_); and 1H-inden-1-one—2,3-dihydro-3,3,5,7-tetramethyl (C_13_H_16_O) (Supplementary Materials G, I Figures G.12, I.27–I.29). Moreover, two derivatives of indole were detected: indole acetic acid (C_10_H_9_NO_2_) and indole carbaldehyde (C_9_H_7_NO) (Supplementary Material I Figures I.7, I.8). The monopyrroles constituted ∼13.9%, and the concentration of pyrrole was 0.222 mg/kg (Figure 1C).

A large group of potential degradation products of the tetrapyrrole ring was identified by the GC-AED analysis. Vanadium-containing organic compounds were detected both in the cell-free aqueous phase (soluble) and in sediment (insoluble) of VO(MTPP)-BC. In the case of VO(MTPP)-BC sample, four vanadium-containing organic compounds were detected in the aqueous phase (C_3_H_x_NV, C_5_H_x_N_2_V, C_8_H_x_N_2_V, and C_10_H_x_N_2_V) (Table 5 and Supplementary Material G Figure G.3A). Three other compounds containing vanadium were detected in the solid phase of the BC (C_20_H_x_N_2_V, C_28_H_x_N_4_V, and C_25_H_x_N_2_V) (Table 5 and Supplementary Material G Figure G.3B). One of the mentioned organic compounds identified in the sediments in strain LM27 cultures containing four nitrogen atoms (C_28_H_x_N_4_V) has been previously described as a product formed after the cleavage of part of the substituents of VO(MTPP). Among the compounds with vanadium are also those that contain two nitrogen atoms.

The concentration of vanadium in the aqueous phase of the BC was determined after 30 days of culture (Figure 1E), which was around 55.514 μg/L. In this study, the concentration of vanadium mobilized by the strain LM27 was very low compared to those reported by Mogolloń et al. (1998),García-Arellano et al. (2004). García-Arellano et al. (2004) used chemically modified cytochrome c to degrade vanadyl mesotetraphenyl porphyrins and removed 95% of the vanadium, as well as eliminated the Soret peak. Mogolloń et al. (1998) used chloroperoxidase to remove 27% of the vanadium from petroporphyrin-rich fractions of Castilla crude oil.

Chemical analysis of the VO(MTPP)-SC showed a slight degradation of the tetrapyrrole ring (∼7.2%) (Figure 1B). However, neither pyrrole-containing compounds (Supplementary Material G Figure G.18) nor vanadium- and/or nitrogen-containing compounds (Supplementary Material G Figure G.13) that could be formed by the breakdown of tetrapyrrole ring was detected in the SC sample. We detected only a slight (0.994 μg/L) mobilization of vanadium into the aqueous phase (Figure 1E).

### Detection of Bacterial Enzymes Potentially Involved in Geoporphyrin Transformation

A number of enzymes were detected and considered to be involved in the degradation of organic compounds, including aromatics, in the metaproteome of bacteria that colonize BISR and in the proteome of the strain LM27 grown on SR or porphyrins (Table 6 and Supplementary Material J). The enzymes were primarily identified as oxygenases (mono- and di-) catalyzing the oxygenation of aliphatic and aromatic organic compounds, respectively. In addition, alcohol and aldehyde dehydrogenases that are involved in the dehydrogenation of alcohols and aldehydes to carboxylic acids were also identified.

Thus, we can say that these enzymes are involved in the oxidation and dehydrogenation of the substituents of porphyrins and are responsible for such compounds found in BCs with Ni (OEP) and VO (OEP) such as the previously mentioned ethane-2,2- a diol; ethanoic acid; and ethanedioic acid (Tables 3, 4). In the case of BCs with VO (MTPP), the enzyme activity can be combined with the presence of butanoic and butanedioic acid, as well as phenol derivatives probably resulting from biotransformation of phenyl substituents (Table 6).

## Conclusion

Our study has demonstrated the following: (i) cleavage and/or degradation of aliphatic and aromatic substituents; (ii) degradation of porphyrin (tetrapyrrole) ring; (iii) formation of organic compounds containing 1, 2, or 3 pyrrole rings, (iv) formation of nickel- or vanadium- and/or nitrogen-containing organic compounds with a chain length of C4 – C24, (v) mobilization of nickel and vanadium from porphyrins.

Detection of biological processes in the environment can be difficult, especially for rocks that are as complex as shale rocks. The applied concept and research methodology in this study has enabled the detection of bacterial geoporphyrin transformation in the environmental samples and simultaneous verification of the results of field studies by performing laboratory studies. There is no SC in the field and there is no way to distinguish between biotic and abiotic changes. The results obtained in the laboratory experiment indicate that the observed geoporphyrin transformations in the BISR sample are, however, of a biological nature.

It is noteworthy that the results of our research using samples collected in the field are consistent with our laboratory experiments, including experiments using synthetic porphyrins. In addition, many of the identified organic compounds that are porphyrin degradation products occur in all or most of the experimental variants (BISR, SR-BC, Ni(OEP)-BC, VO(OEP)-BC, and VO(MTPP)-BC). Particular attention should be paid to pyrrole compounds resulting from the breakdown of the tetrapyrrole ring, such as tripyrrole — 3 - [(5Z) -5 - [[4-Ethenyl-5 - [(Z) - (4-ethenyl-3- methyl-5-oxopyrrol-2-ylidene) methyl] -3-methyl-1H-pyrrol-2-yl] methylidene] -4-methyl-2-opyrrol-3-yl] propanoate, bipyrrole, or indole acetic acid containing a single pyrrole ring. These compounds were not detected in the SR sample or in any SC samples but were detected in all the tested variants in which bacteria were present. We can consider pyrrole-containing compounds as markers of biological transformations of geoporphyrins. The same is true for nickel- or vanadium- and/or nitrogen-containing organic compounds with a chain length of C4 – C24. These compounds were neither detected in the SR sample nor in any SC samples; therefore it can be assumed that these compounds were formed by the breakdown of the tetrapyrrole ring. Based on the established chemical formulas, it can be assumed that among them are both those that contain two pyrrole rings (compounds containing two atoms of nitrogen) and one pyrrole ring (compounds containing one nitrogen atom). We should also emphasize the impact of bacteria inhabiting shale rock on the changes of the composition and content of geoporphyrins, composition of extractable organic matter, as well as on the nickel and vanadium content in the shale rocks.

Summarizing, the presented research indicates the breakdown of complex tetrapyrroles occurring in the shale rock into simpler compounds. This process also means mobilization of fossil organic carbon associated for millions of years in fossil sedimentary rock to global cycle of carbon on the Earth. The obtained results can also be applied to other sedimentary formations containing geoporphyrins, especially if we take into account that the bacteria described in our research are common in fossil fuels as well as their associated source rocks.

## Data Availability Statement

The original contributions presented in the study are included in the article/Supplementary Material, further inquiries can be directed to the corresponding author/s.

## Author Contributions

RS performed the experiments and analyzes, analyzed the data, and wrote the manuscript. RM invented the idea of research, designed the experiments, conceived the idea of the manuscript, analyzed the data, and wrote the manuscript. Both authors contributed to the article and approved the submitted version.

## Conflict of Interest

The authors declare that the research was conducted in the absence of any commercial or financial relationships that could be construed as a potential conflict of interest.

## Publisher’s Note

All claims expressed in this article are solely those of the authors and do not necessarily represent those of their affiliated organizations, or those of the publisher, the editors and the reviewers. Any product that may be evaluated in this article, or claim that may be made by its manufacturer, is not guaranteed or endorsed by the publisher.
