# Supplementary material for: Postdiagenetic Bacterial Transformation of Nickel and Vanadyl Sedimentary Porphyrins of Organic-Rich Shale Rock (Fore-Sudetic Monocline, Poland)
Source: Front Microbiol. 2021 Nov 30;12:772007. doi: 10.3389/fmicb.2021.772007 (PMC8669743; doi:10.3389/fmicb.2021.772007)
Supplement: Supplementary file 1 [file Table_1.DOCX]

**Supplementary Material A. Supplementary results for synthetic porphyrins and other organic compounds studied in the presented work**


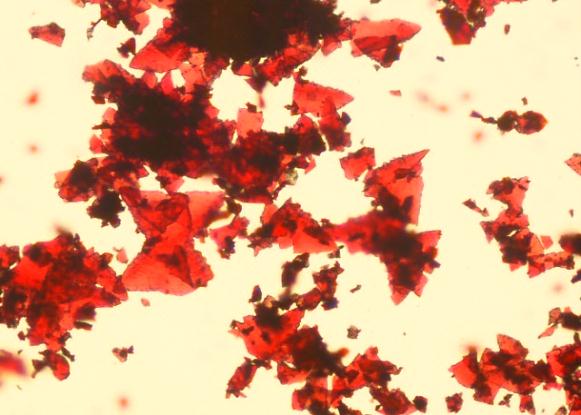

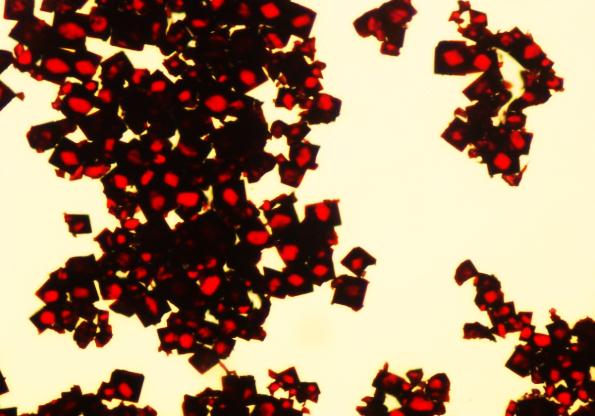

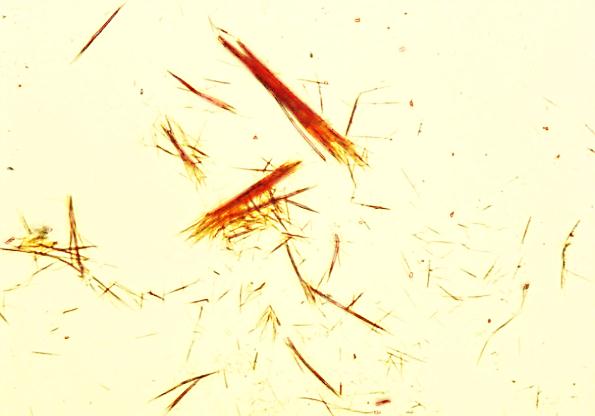

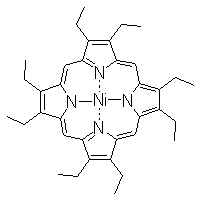

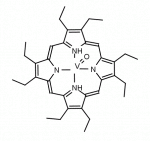

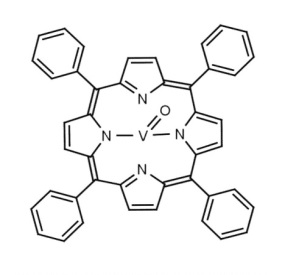


**A**

**B**

**C**

Figure A.1. Light microphotographs and structural formula of three synthetic porphyrins studied in laboratory experiment: Ni(OEP) (A), VO(OEP) (B), and VO(MTPP) (C); magnification 40x


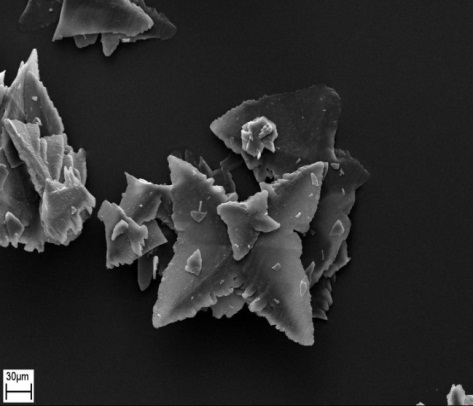

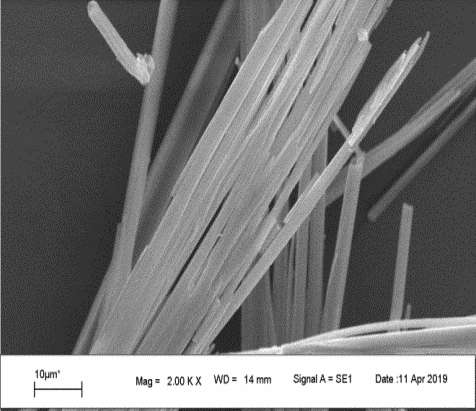

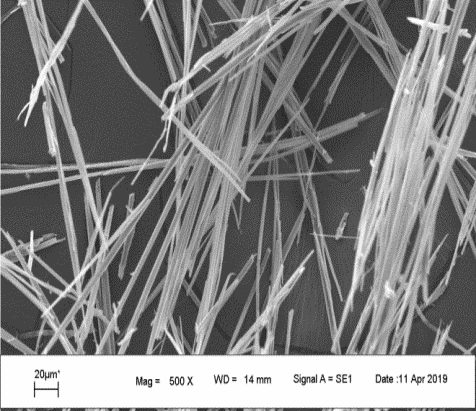

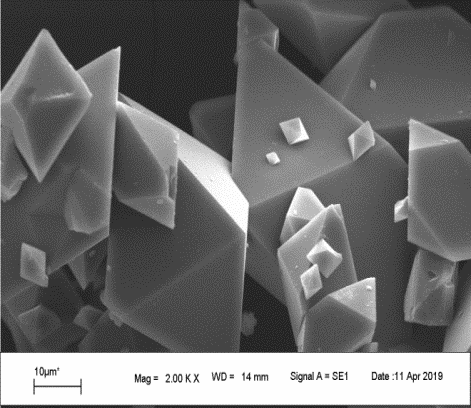

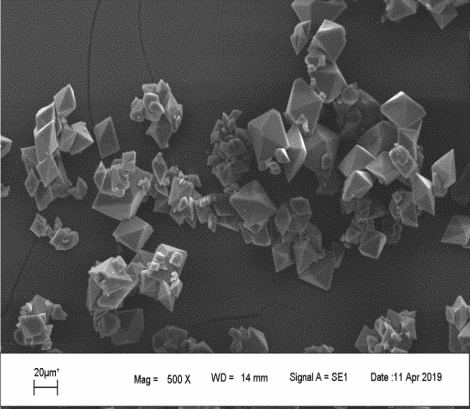

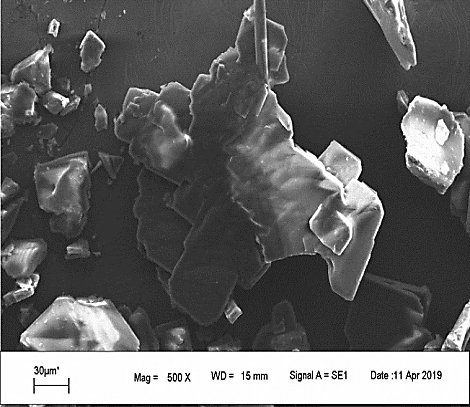


**E**

**A**

**C**

**B**

**D**

**F**

Figure A.2. Scanning electron microphotographs of three synthetic porphyrins studied in laboratory experiment: Ni(OEP) (A, B), VO(OEP) (C, D), and VO(MTPP) (E, F)


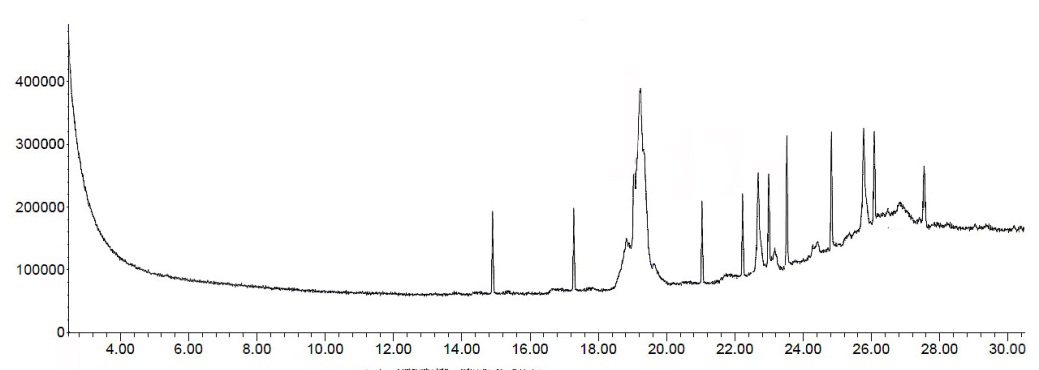


Time (min)

Abundance


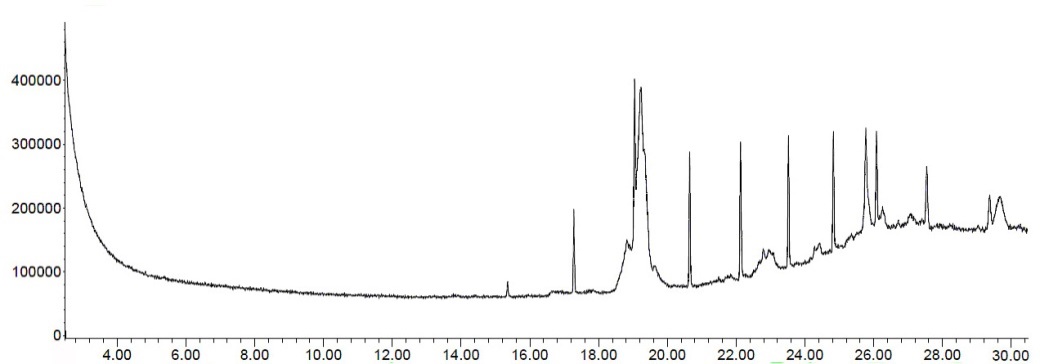


Time (min)

Abundance


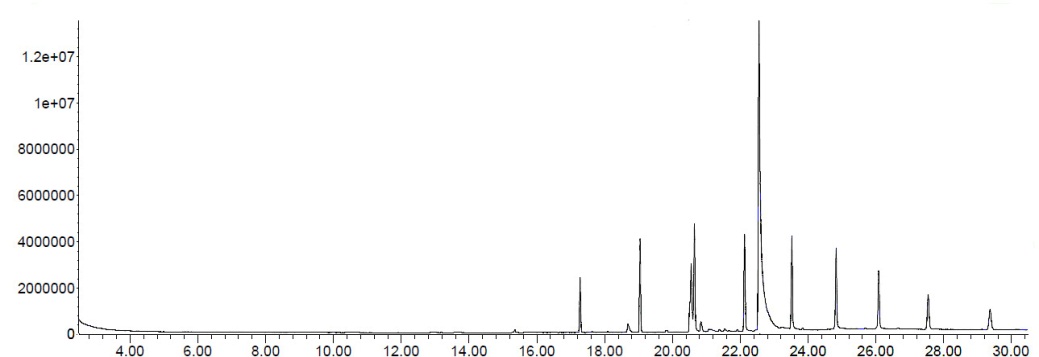


Time (min)

Abundance

Figure A.3. Selected ion monitoring chromatograms of three synthetic porphyrins studied in laboratory experiment: Ni(OEP) (m/z 591), VO(OEP) (m/z 599) and VO(MTPP) (m/z 679)

VO(MTPP) - m/z 679

VO(OEP) - m/z 599

Ni(OEP) - m/z 591


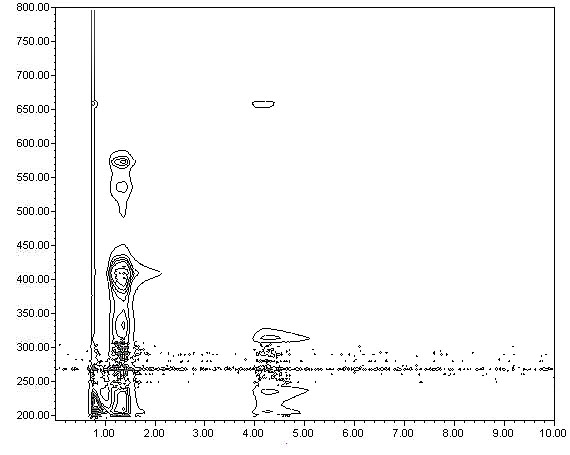


Time (min)

Wavelength (nm)

**A**


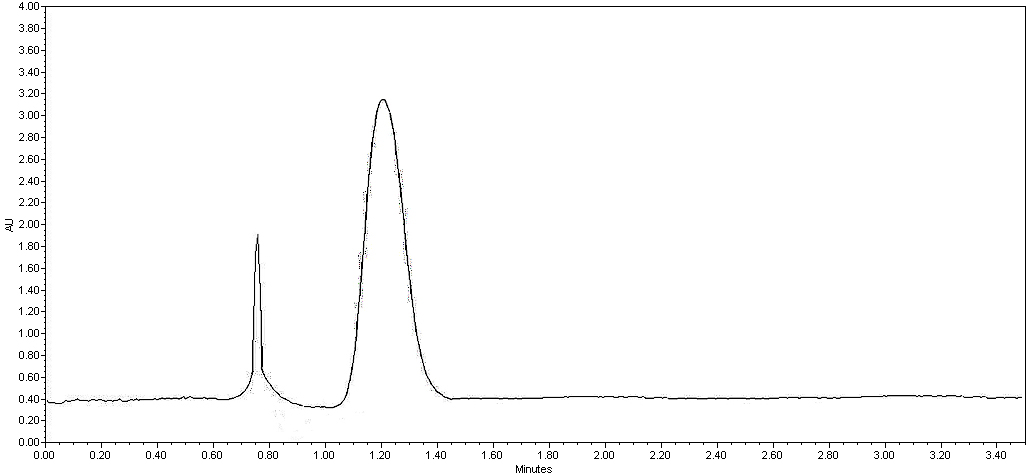


Ni(OEP)

Chloroform

Abundance

Time (min)

**B**


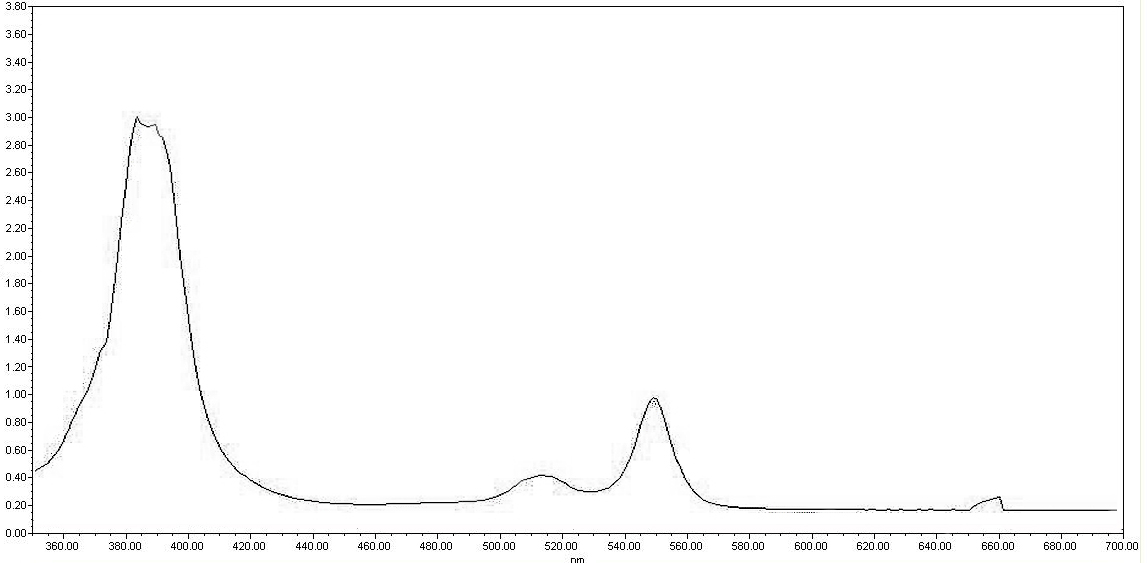


380 nm

516 nm

549 nm

660 nm

Wavelength (nm)

Abundance

**C**

Figure A.4. HPLC-PDA 3D chromatogram (A), 425 nm chromatogram (B) and UV-Vis spectrum (C) of Ni (OEP)


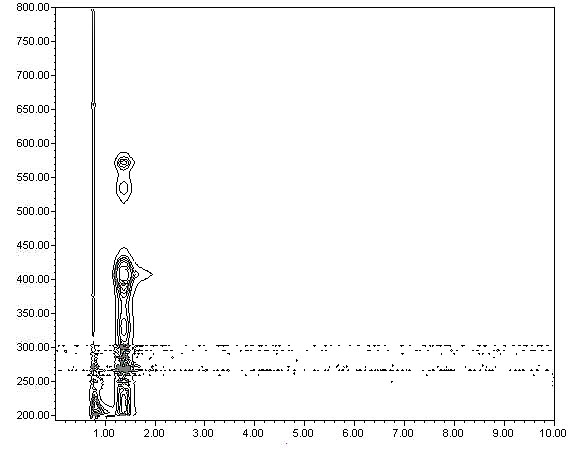


Time (min)

Wavelength (nm)

**A**


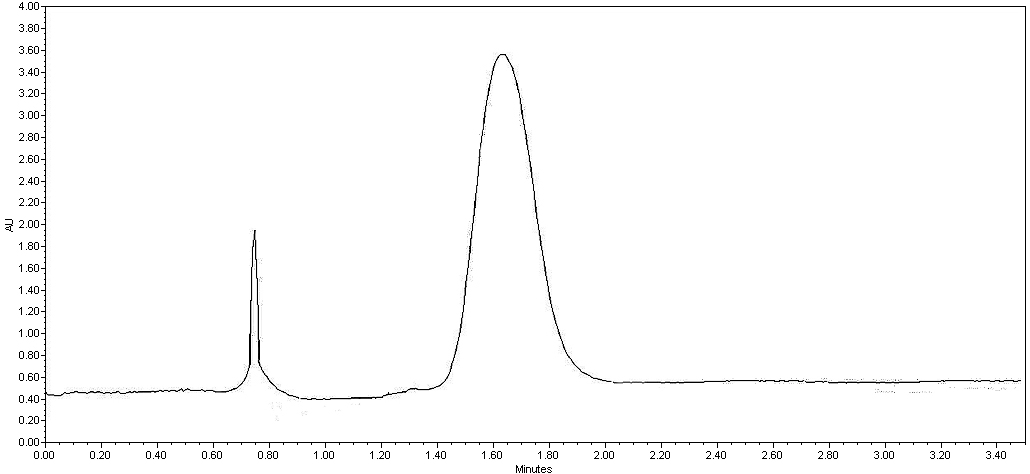


VO(OEP)

Chloroform

Abundance

Time (min)

**B**


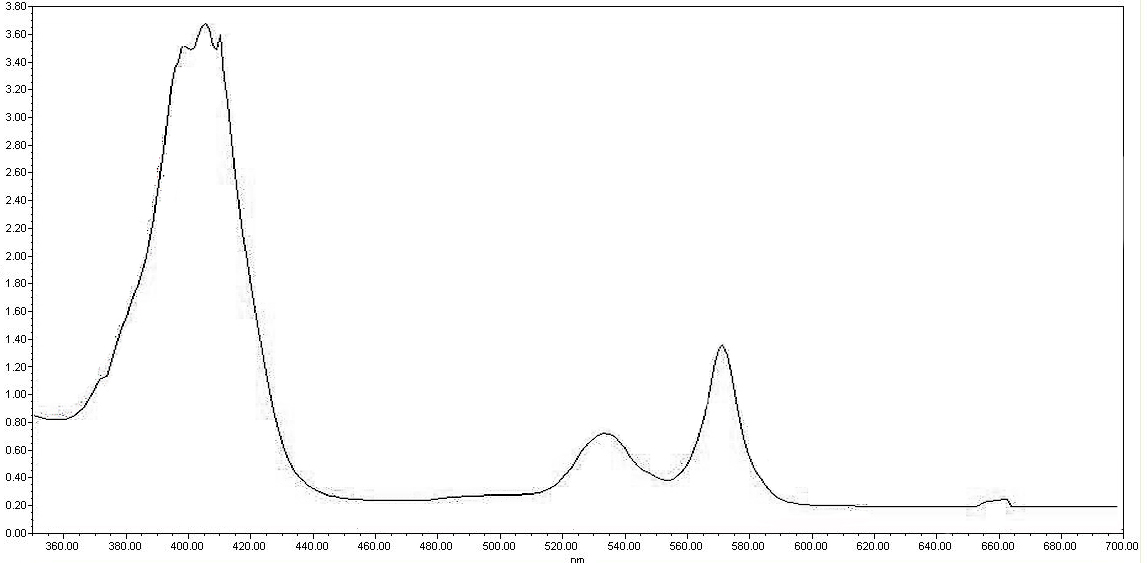


401 nm

529 nm

571 nm

660 nm

Wavelength (nm)

Abundance

**C**

Figure A.5. HPLC-PDA 3D chromatogram (A), 425 nm chromatogram (B) and UV-Vis spectrum (C) of VO(OEP)


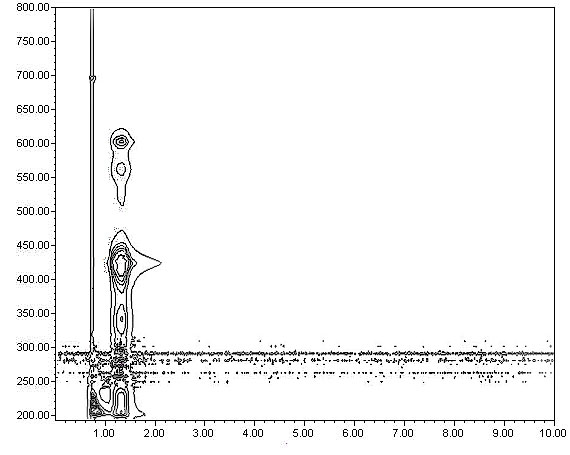


Time (min)

Wavelength (nm)

**A**


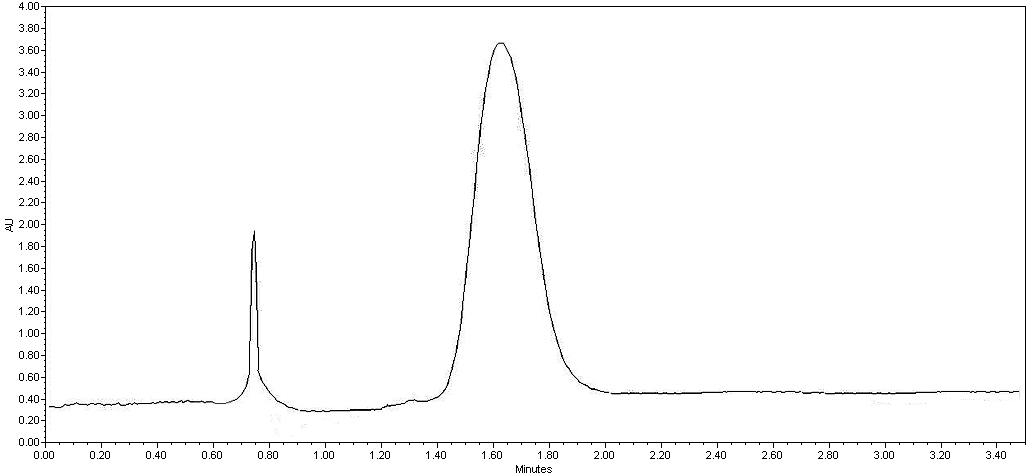


VO(MTPP)

Chloroform

Abundance

Time (min)

**B**


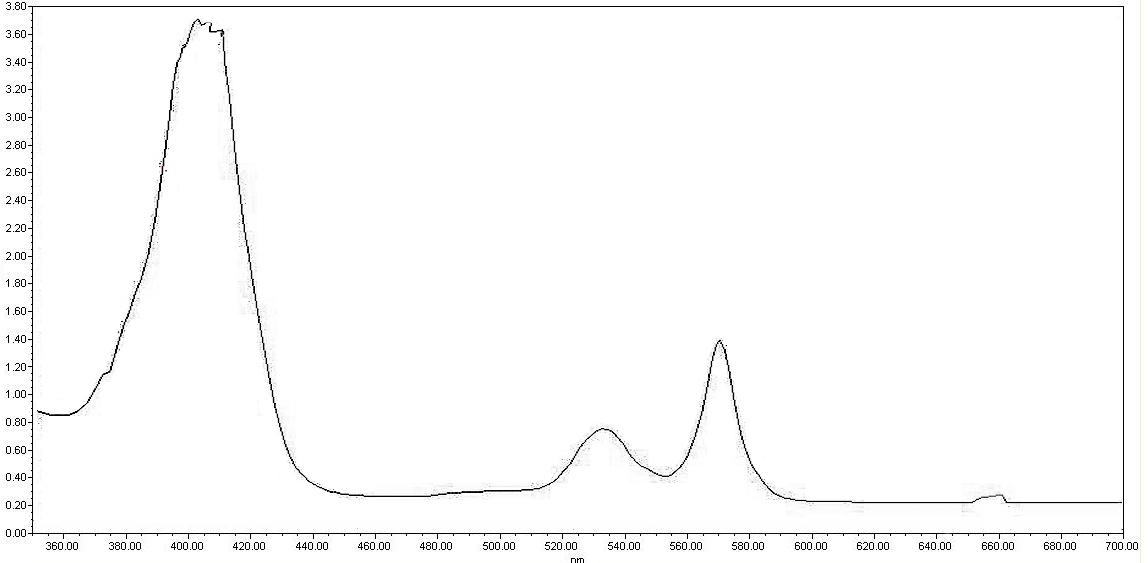


401 nm

529 nm

571 nm

660 nm

Wavelength (nm)

Abundance

**C**

Figure A.6. HPLC-PDA 3D chromatogram (A), 425 nm chromatogram (B) and UV-Vis spectrum (C) of VO(MTPP)


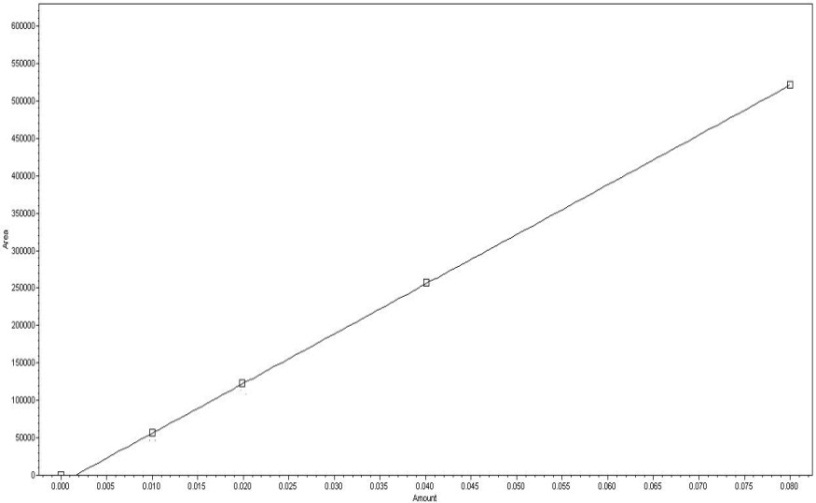


Area

Concentration (mM)


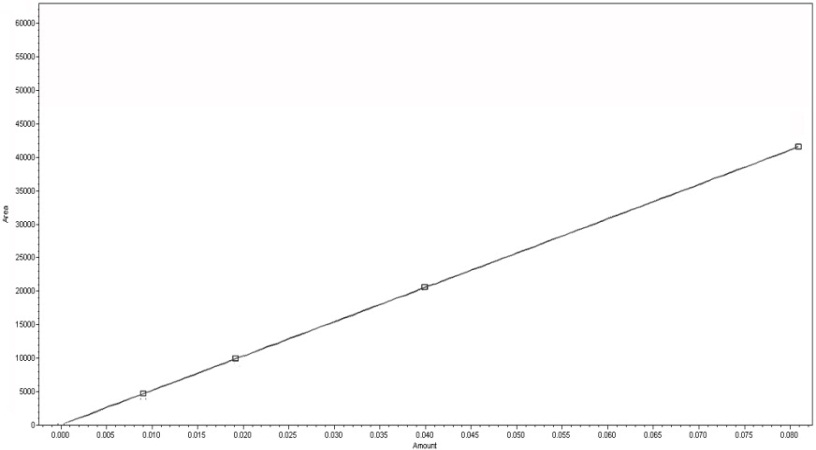


Area

Concentration (mM)

**A**

**B**

**C**

**D**


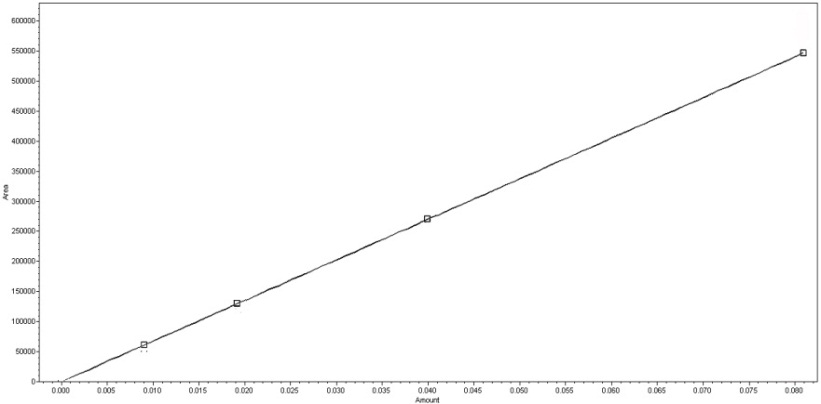


Area

Concentration (mM

| **Porphyrin** | **R** | **R^2^** |
| --- | --- | --- |
| Ni(OEP) | 0,997500 | 0,995007 |
| VO(OEP) | 0,997262 | 0,994531 |
| VO(MTPP) | 0,996281 | 0,991382 |

Figure A.7. Calibration curves for determining concentration of three synthetic porphyrins studied in laboratory experiment: Ni(OEP) (A), VO(OEP) (B), and VO(MTPP) (C); correlation coefficient (r) and coefficient of determination (r2) (D)

C_36_H_44_N_4_Ni

**A**

**B**

**C**


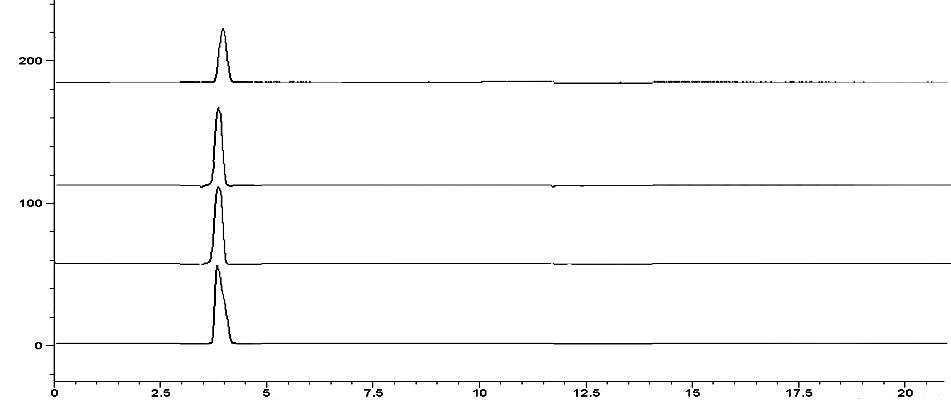


**N**

**H**

**C**

**Ni**

Time (min)

Abundance

C_36_H_44_N_4_V


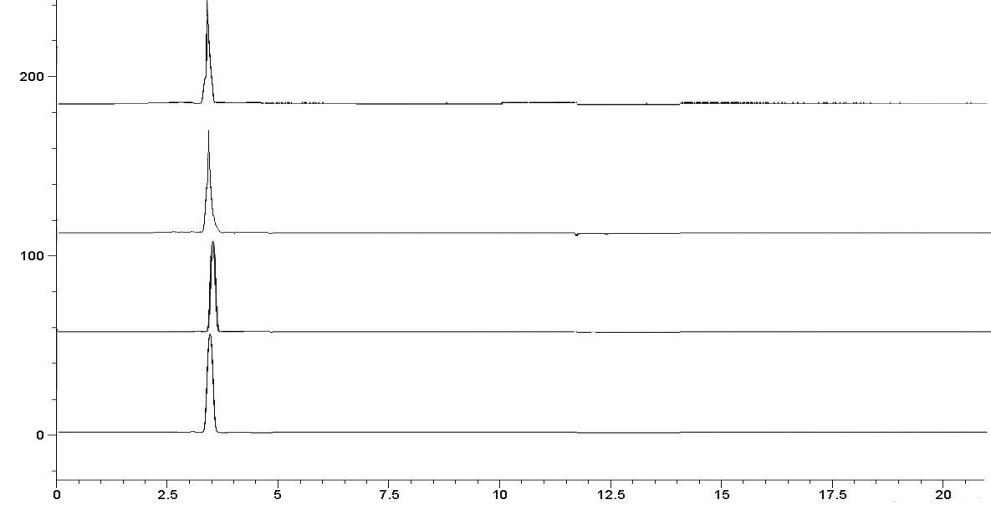


**N**

**H**

**C**

**V**

Time (min)

Abundance

C_44_H_28_N_4_V


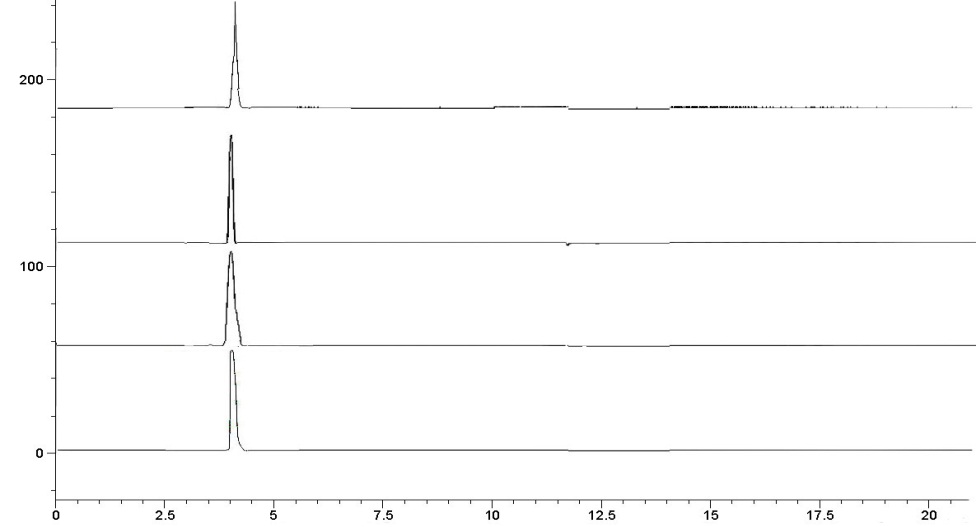


**N**

**H**

**C**

**V**

Time (min)

Abundance

Figure A.8. Atomic emission spectra of three synthetic porphyrins studied in laboratory experiment: Ni(OEP) (A), VO(OEP) (B), and VO(MTPP) (C)


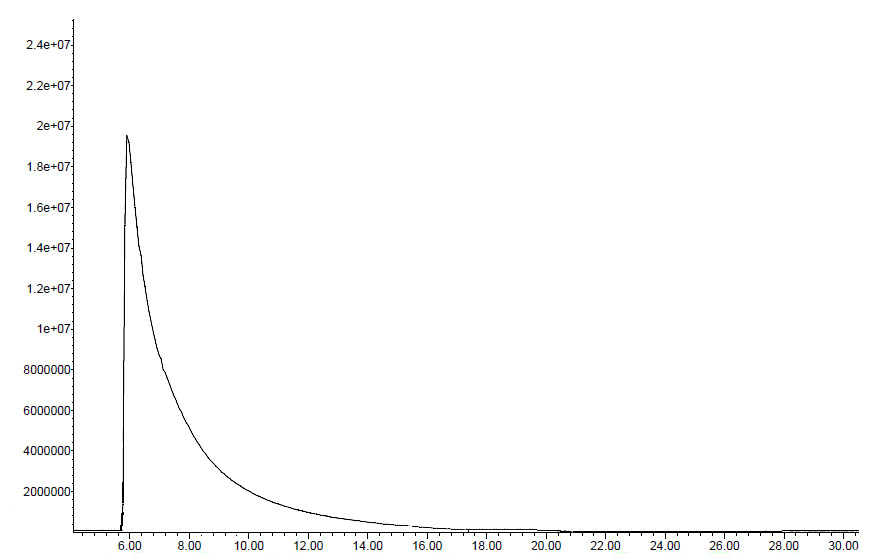


Time (min)

Abundance


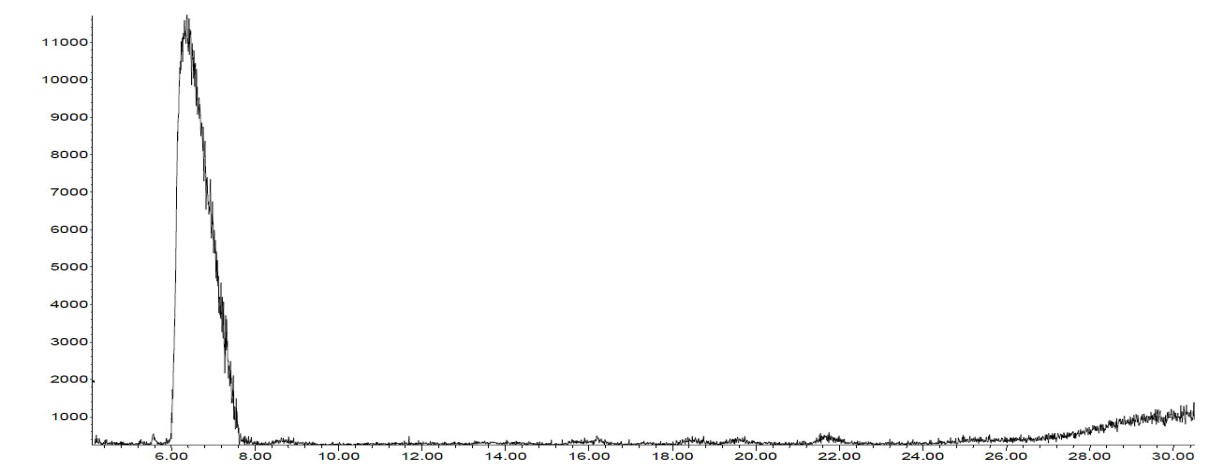


Abundance

Time (min)

**A**

**A**

**B**

**C**

Time (min)

**B**


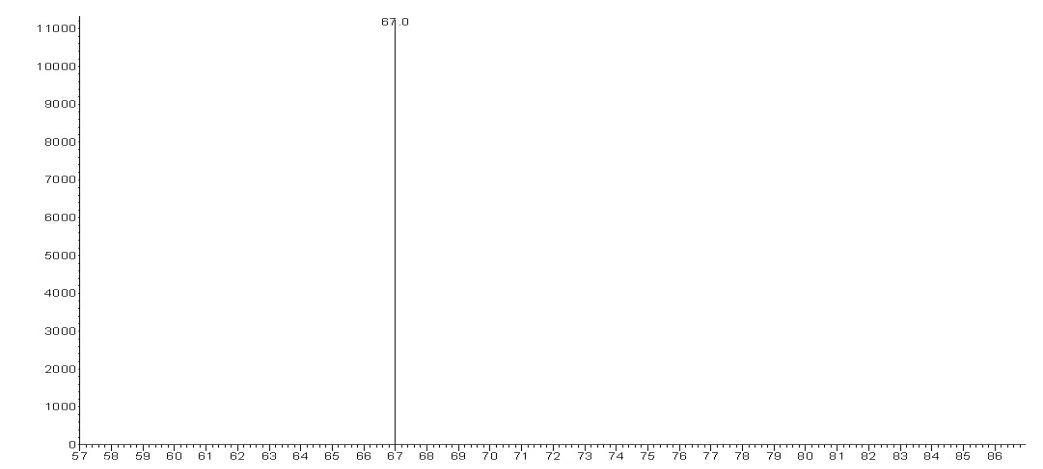


m/z

Abundance

**C**

**D**

Figure A.9. Detection of pyrrole: total ion current chromatogram (A), selected ion (m/z 67) monitoring chromatogram (B), mass spectrum (C) and calibration curve for determining pyrrole concentration (D)


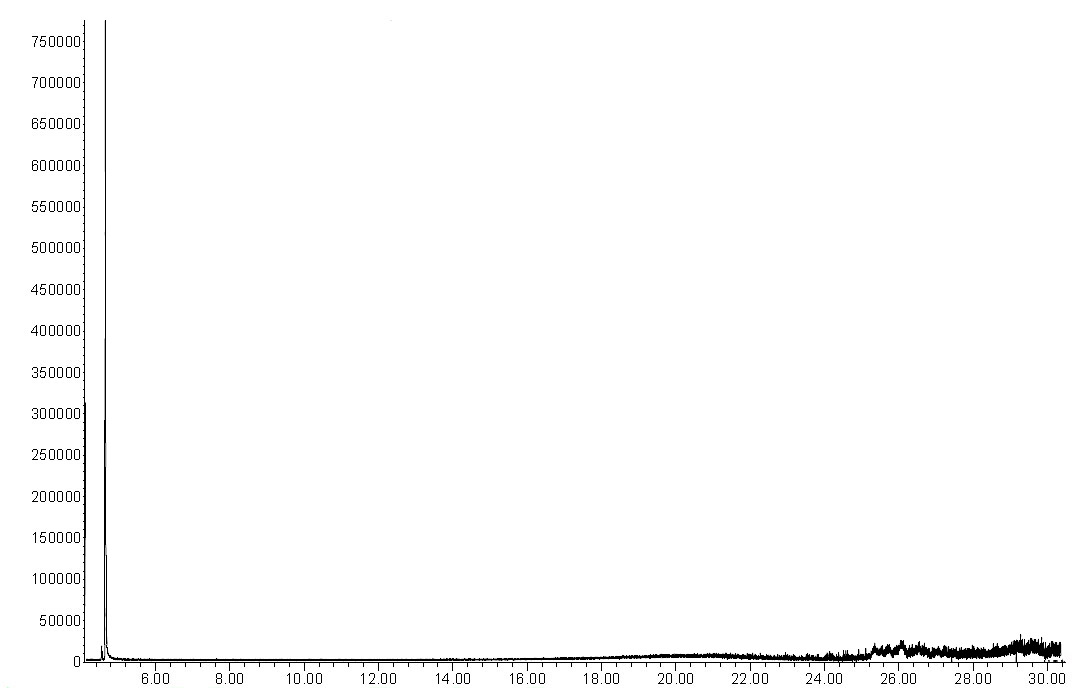


Time (min)

Abundance

4.468


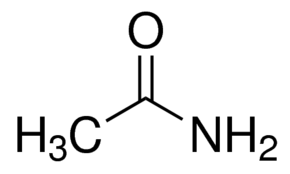


**A**


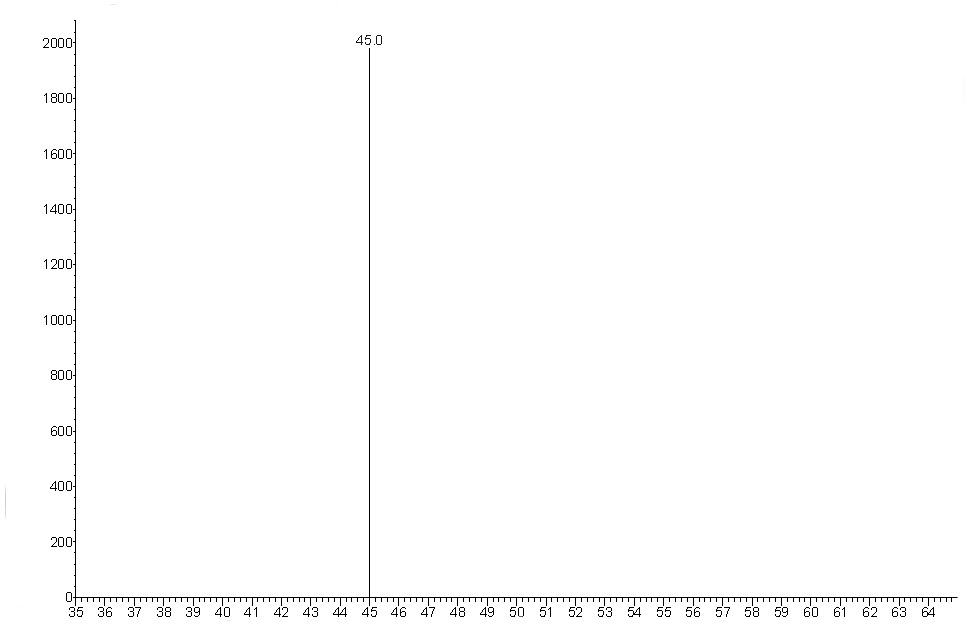


m/z

Abundance

**B**

Figure A.10. Detection of organic compounds containing ethyl group: selected ion ( m/z 45) monitoring chromatogram (A) and mass spectrum (B)


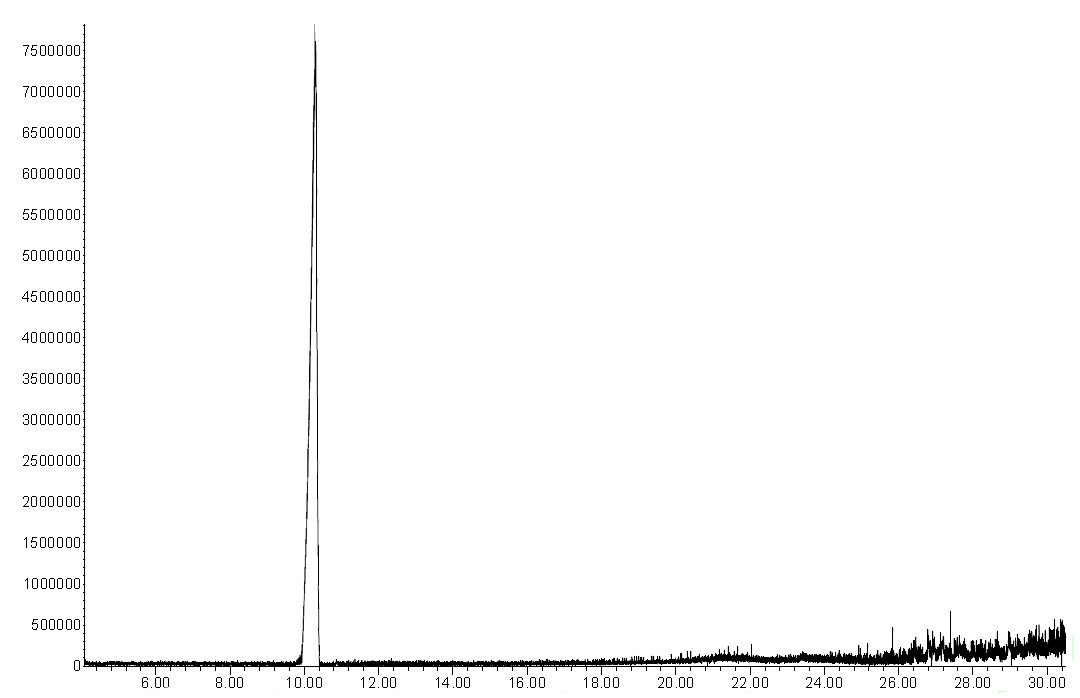


Time (min)

Abundance

10.094


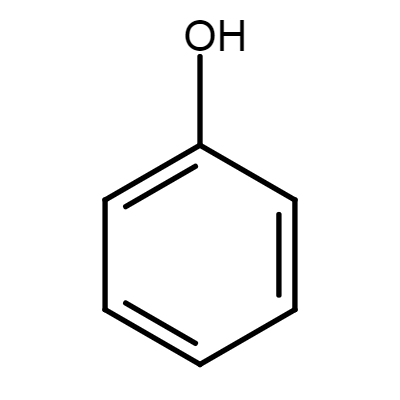


**A**


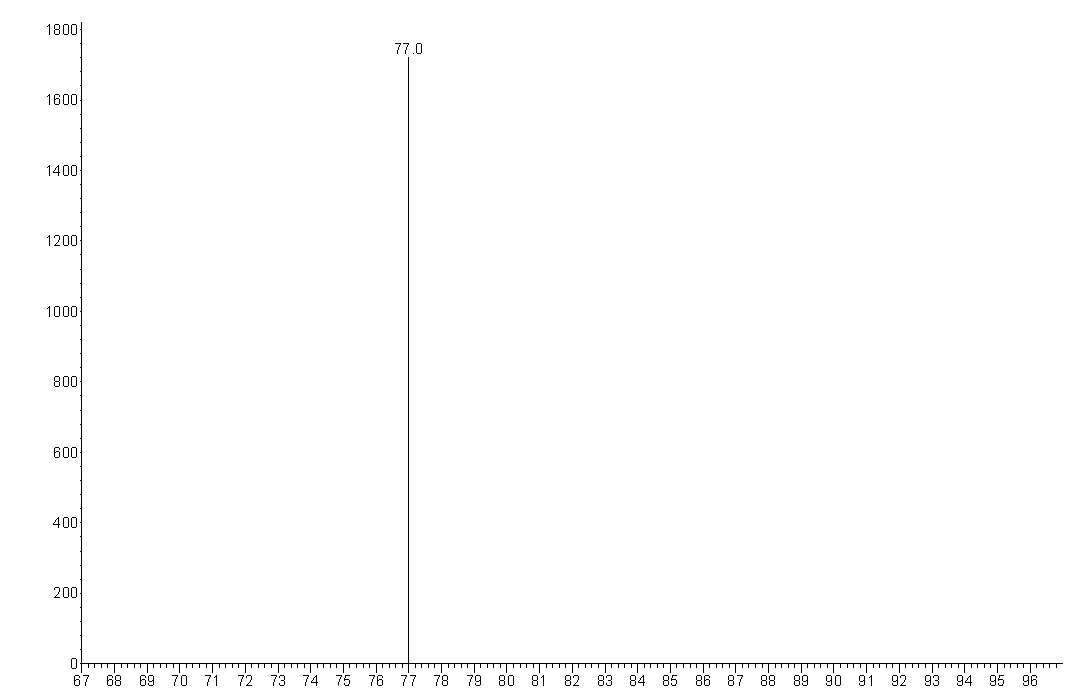


m/z

Abundance

**B**

Figure A.11. Detection of organic compounds containing phenyl group: selected ion (m/z 77) monitoring chromatogram (A) and mass spectrum (B)

Figure A.12. Detection of butanoic acid: selected ion (m/z 88) monitoring chromatogram (A) and mass spectrum (B)


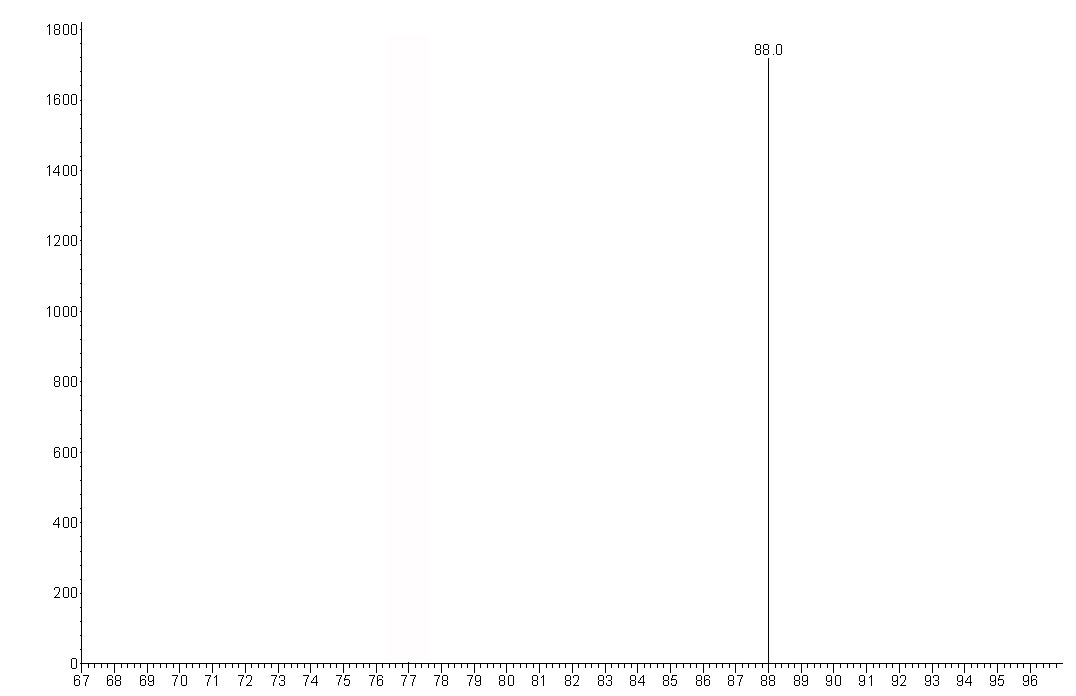


m/z

Abundance

**B**


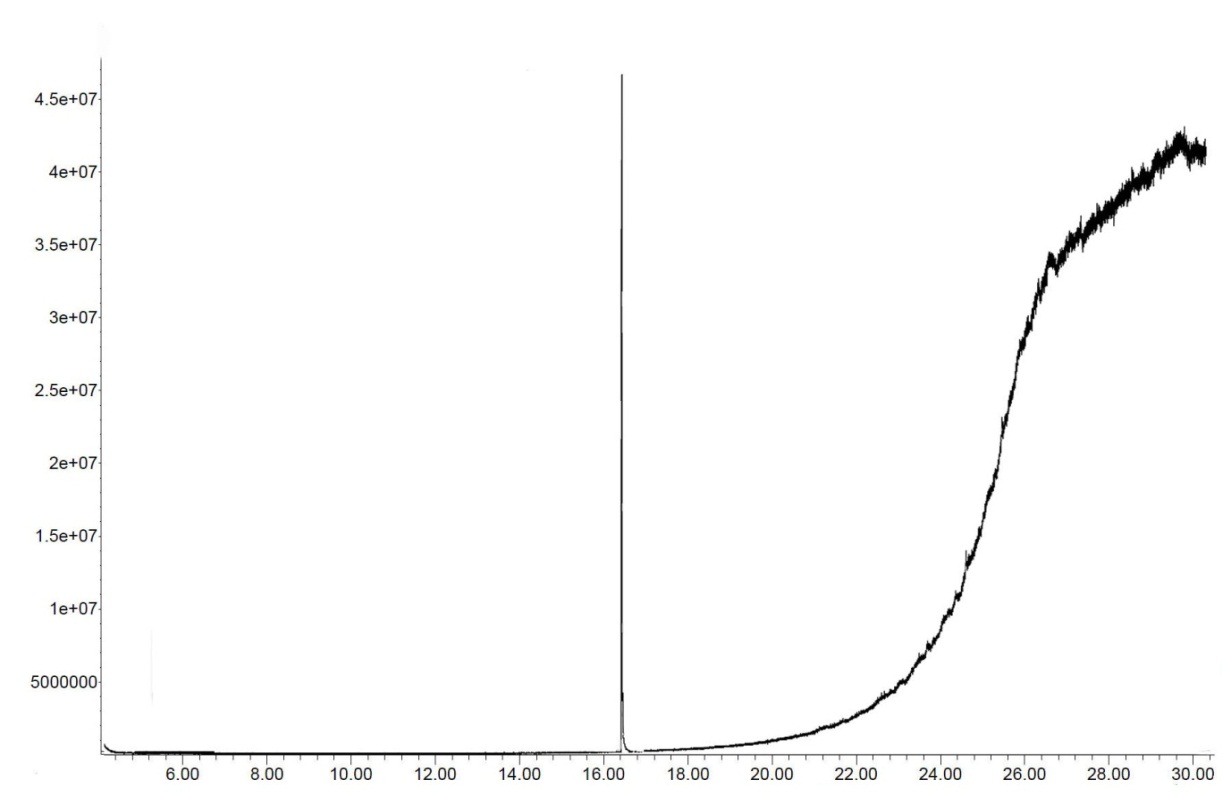


Time (min)

Abundance

16.621


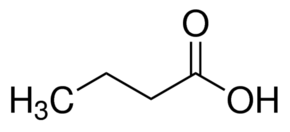


**A**
