# Supplementary material for: Postdiagenetic Bacterial Transformation of Nickel and Vanadyl Sedimentary Porphyrins of Organic-Rich Shale Rock (Fore-Sudetic Monocline, Poland)
Source: Front Microbiol. 2021 Nov 30;12:772007. doi: 10.3389/fmicb.2021.772007 (PMC8669743; doi:10.3389/fmicb.2021.772007)
Supplement: Supplementary file 2 [file Table_2.DOCX]

**Supplementary Material B. Supplementary results for shale rock (SR)**


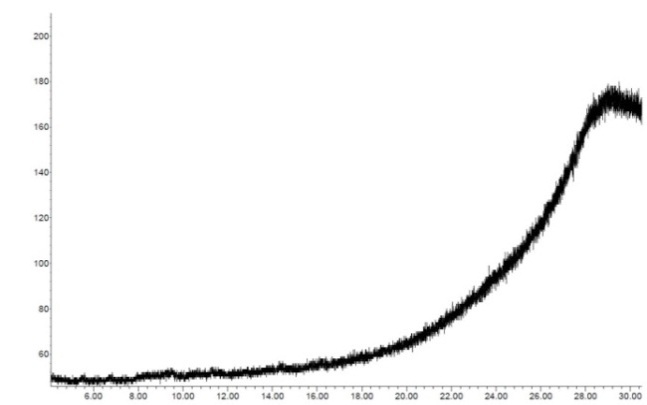


*m/z:* 368 - total peak area: 0

Time (min)

Abundance


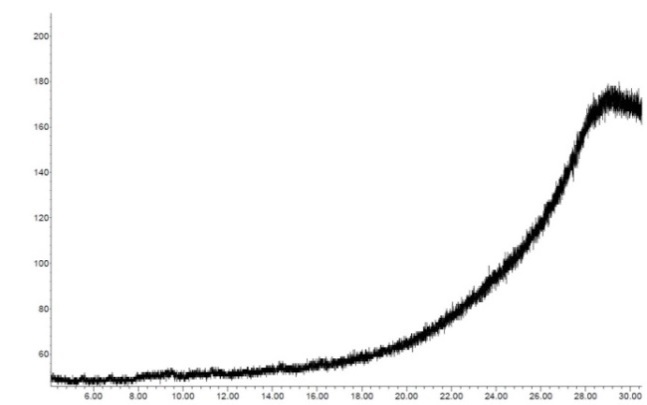


*m/z:* 361 - total peak area: 0

Time (min)

Abundance


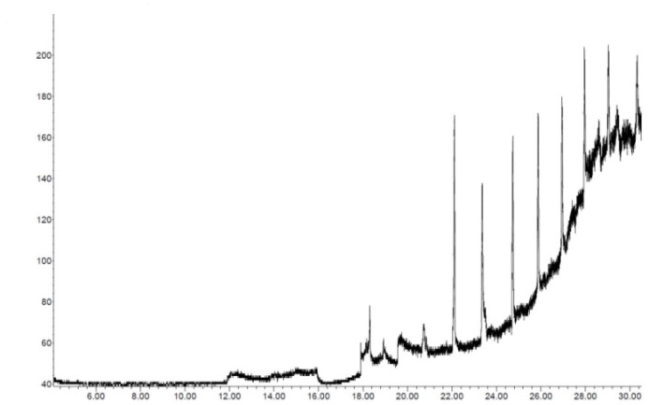


*m/z:* 591 - total peak area: 28198

Abundance


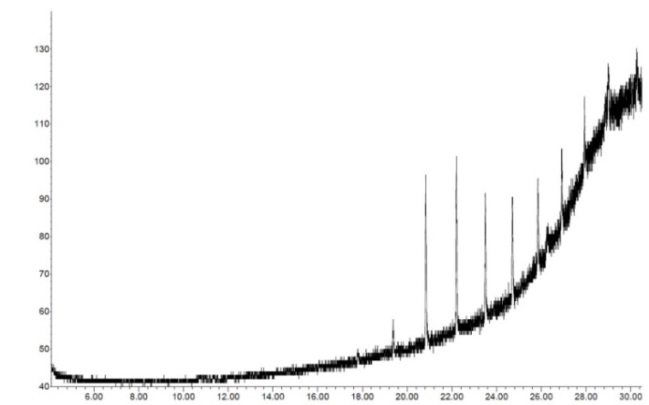


*m/z:* 599 - total peak area: 11114

Abundance


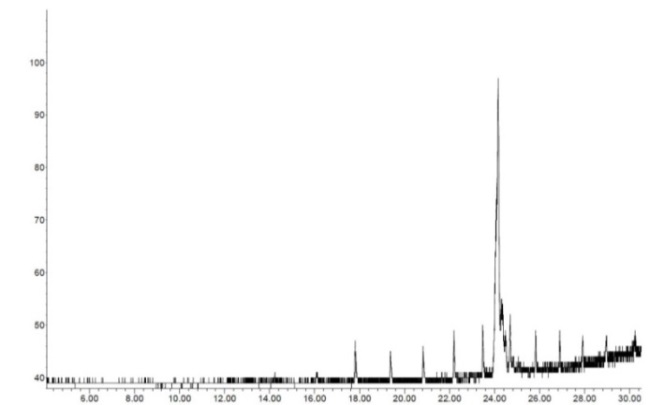


*m/z:* 679 - total peak area: 8978

Abundance


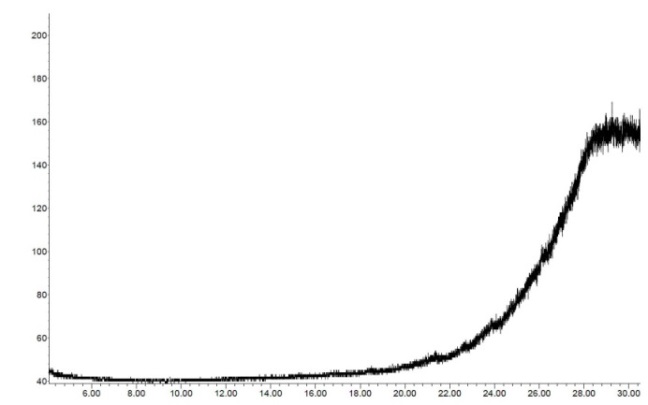


*m/z:* 528 - total peak area: 0

Abundance


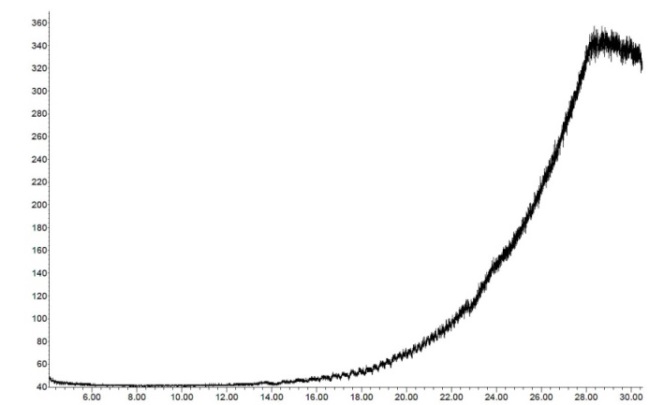


*m/z:* 472 - total peak area: 0

Abundance


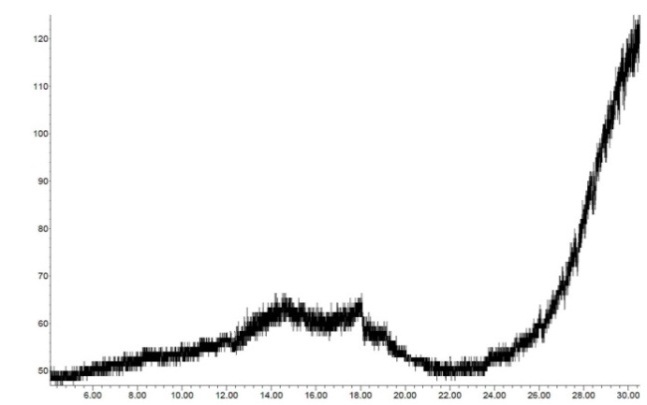


*m/z:* 481 - total peak area: 0

Abundance

**Figure B.1.** Selected ions (*m/z:* 679, 599, 591, 528, 481, 472, 368, and 361) monitoring chromatograms of shale rock (SR)


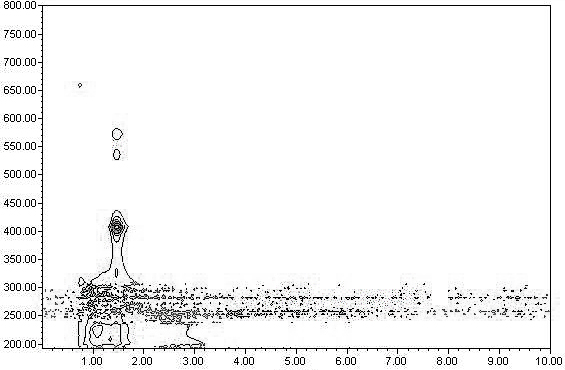


Time (min)

Wavelength (nm)

**A**


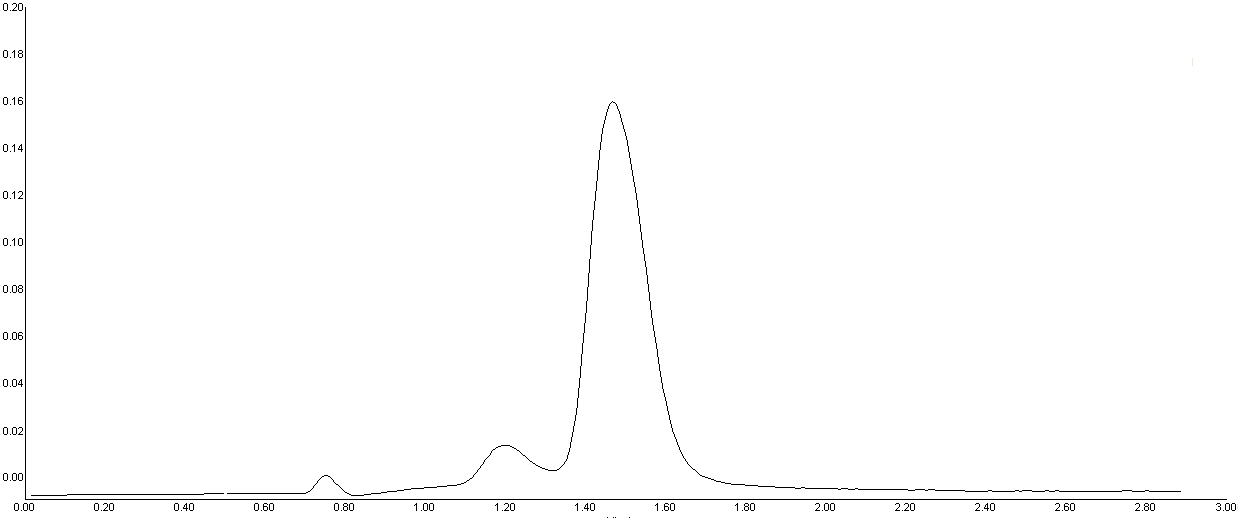


Vanadyl porphyrin

Nickel

porphyrin

Chloroform

Abundance

Time (min)

nickel porphyrin

vanadyl porphyrin

**A**

**B**

**C**


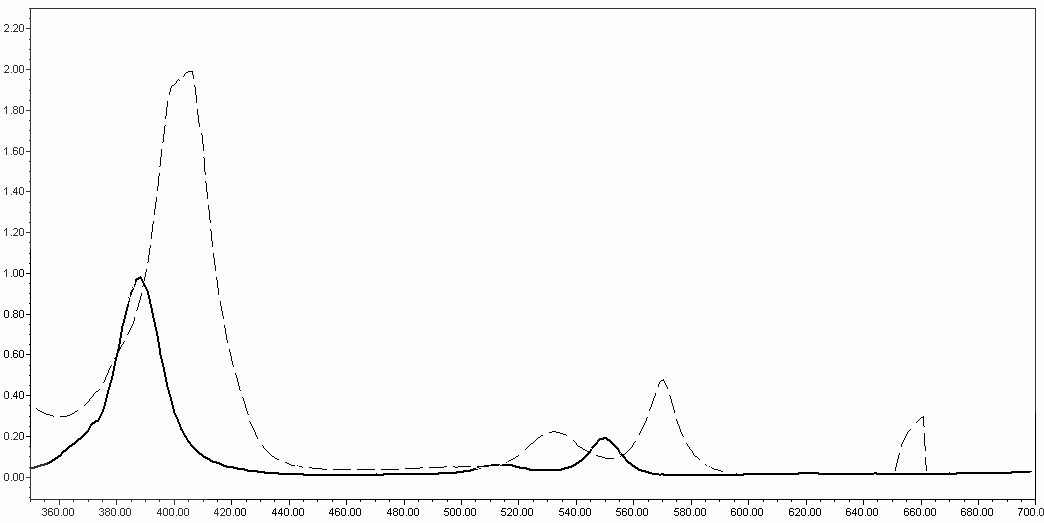


380 nm

401 nm

529 nm

571 nm

516 nm

549 nm

660 nm

Wavelength (nm)

Abundance

**Figure B.2.** High-performance liquid chromatography with photodiode array detector (HPLC-PDA): 3D chromatogram (A), 425 nm chromatogram (B) and UV-Vis spectra, (C) of shale rock (SR)


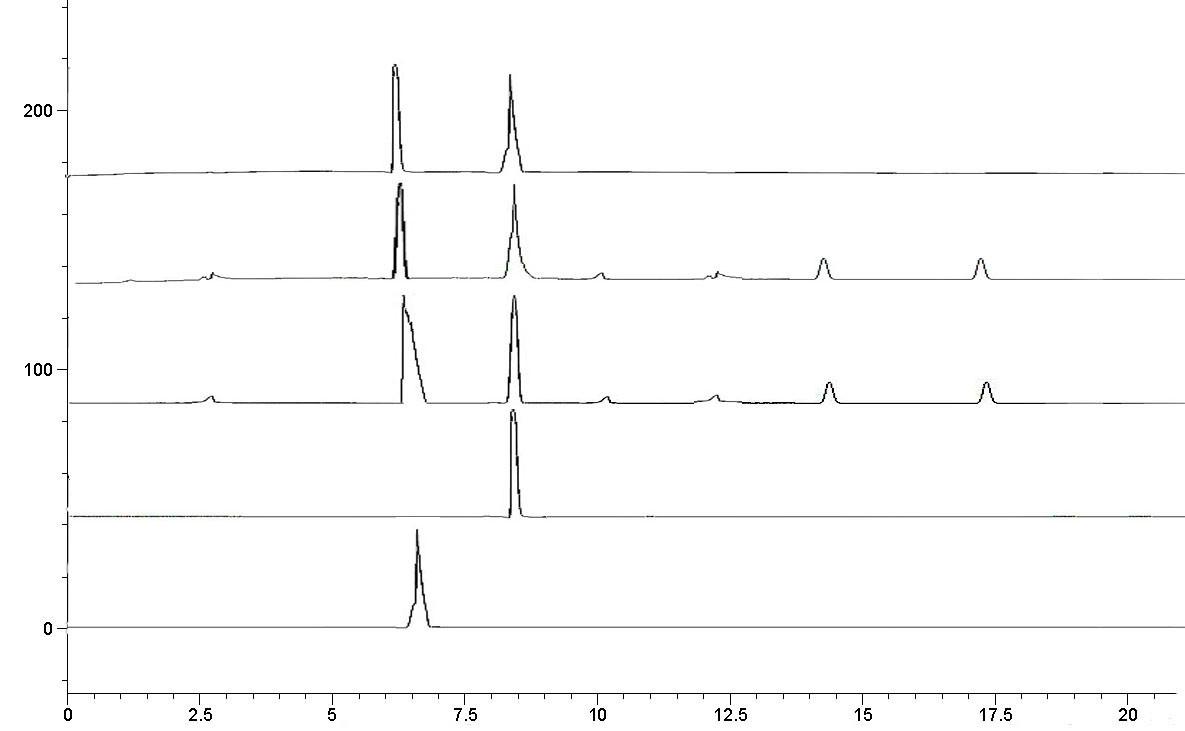


**N**

**H**

**C**

**V**

Time (min)

Abundance

C_44_H_x_N_4_V

**Ni**

C_31_H_x_N_4_Ni

C_20_H_x_

C_22_H_x_

C_30_H_x_

C_36_H_x_

**Figure B.3.** The atomic emission spectrum of shale rock (SR)


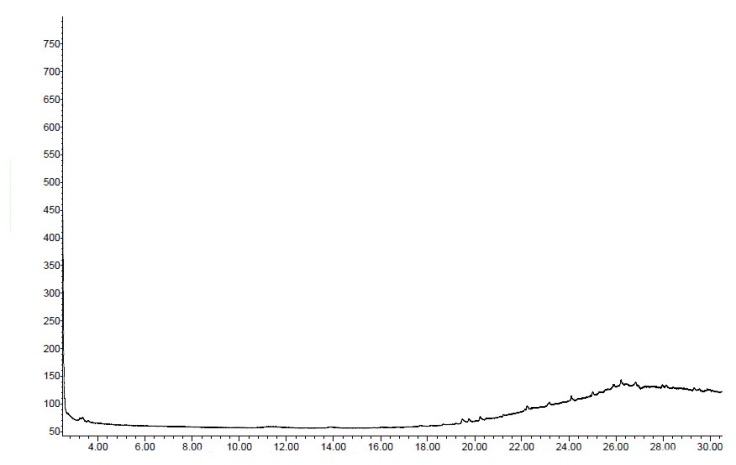


Abundance

Time (min)


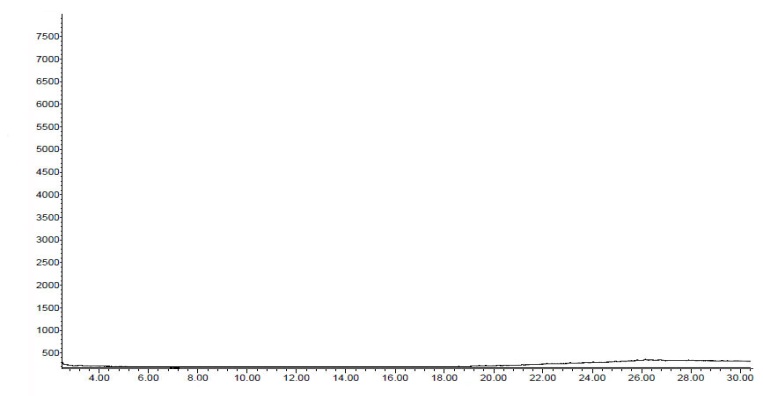


Abundance

Time (min)


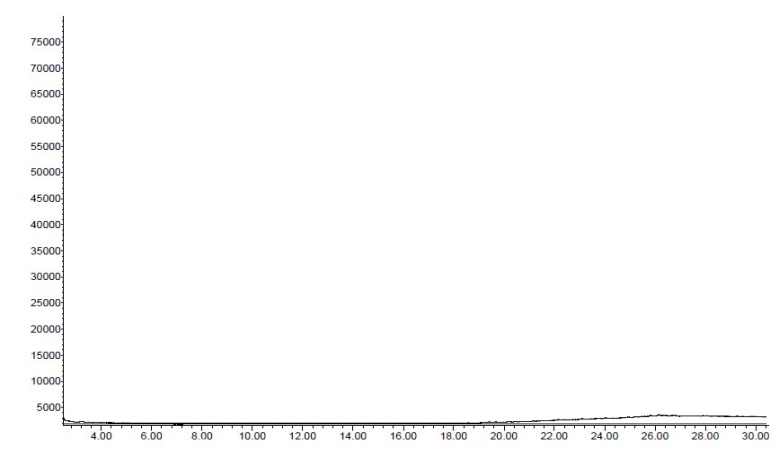


Abundance

Time (min)

*m/z:* 201 - total peak area: 0

*m/z:* 134 - total peak area: 0

*m/z:* 67 - total peak area: 0

**Figure B.4.** Selected ions monitoring chromatograms: *m/z:* 201 (organic compounds containing 3 pyrrole rings), *m/z:* 134 (organic compounds containing 2 pyrrole rings) and *m/z:* 67 (organic compounds containing 1 pyrrole ring) of shale rock (SR)
