# Supplementary material for: Postdiagenetic Bacterial Transformation of Nickel and Vanadyl Sedimentary Porphyrins of Organic-Rich Shale Rock (Fore-Sudetic Monocline, Poland)
Source: Front Microbiol. 2021 Nov 30;12:772007. doi: 10.3389/fmicb.2021.772007 (PMC8669743; doi:10.3389/fmicb.2021.772007)
Supplement: Supplementary file 3 [file Table_3.DOCX]

**Supplementary Material C. Supplementary results for bacteria-inhabited shale rock (BISR)**


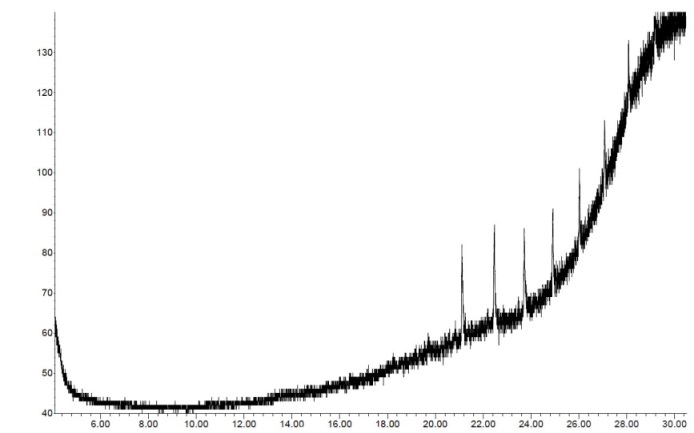


*m/z:* 599 - total peak area: 4501

Abundance


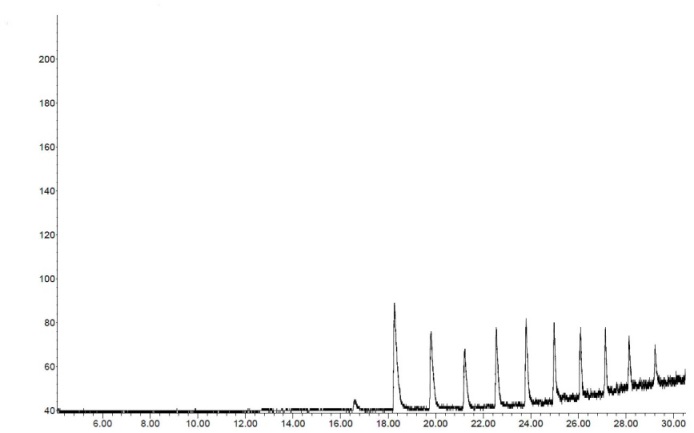


*m/z:* 591 - total peak area: 10081

Abundance


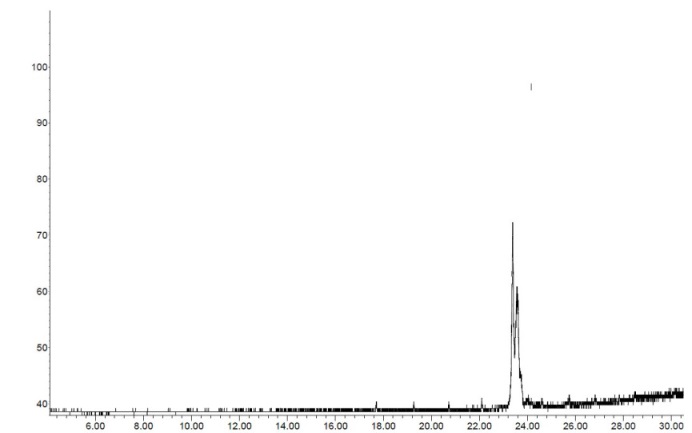


*m/z:* 679 - total peak area: 4818

Abundance


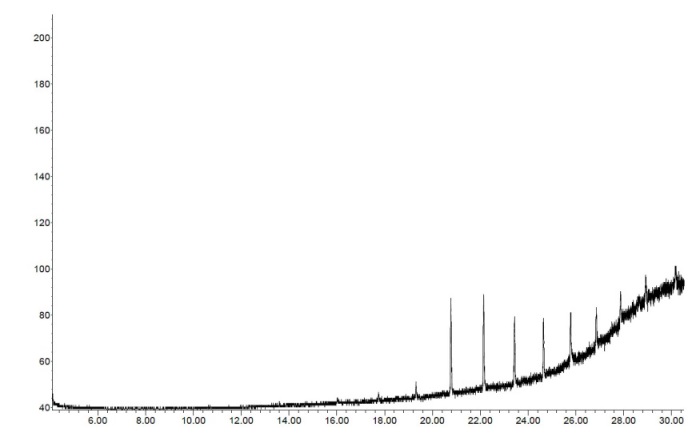


*m/z:* 528 - total peak area: 1651

Abundance


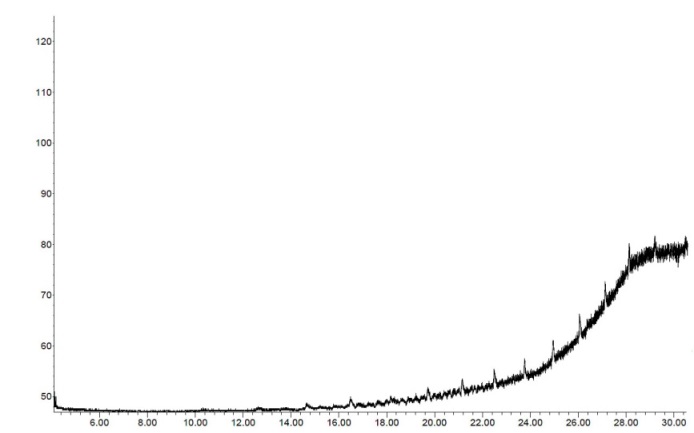


*m/z:* 481 - total peak area: 682

Abundance


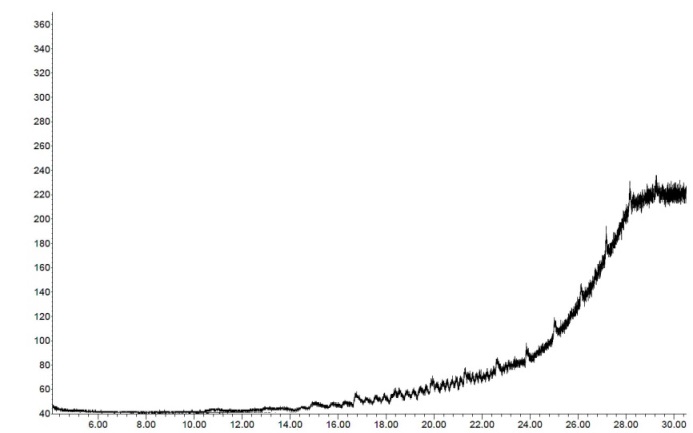


*m/z:* 472 - total peak area: 1002

Abundance


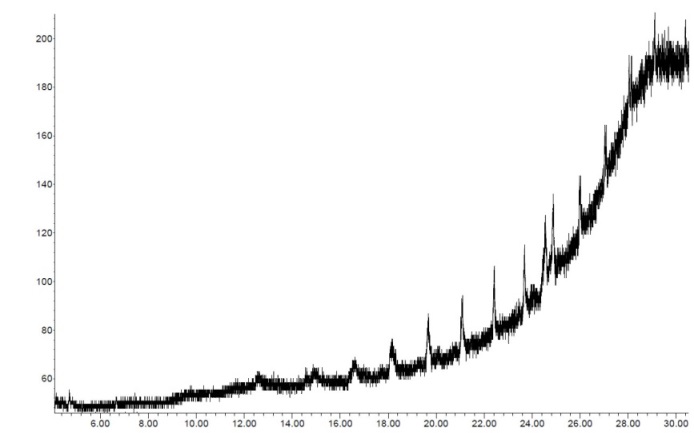


*m/z:* 361 - total peak area: 1286

Time (min)

Abundance


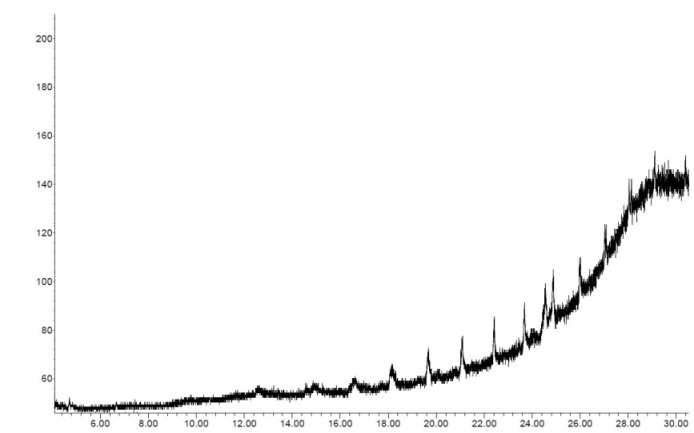


*m/z:* 368 - total peak area: 947

-

Time (min)

Abundance

**Figure C.1.** Selected ions (*m*/*z*: 679, 599, 591, 528, 481, 472, 368, and 361) monitoring chromatograms of bacteria-inhabited shale rock (BISR)


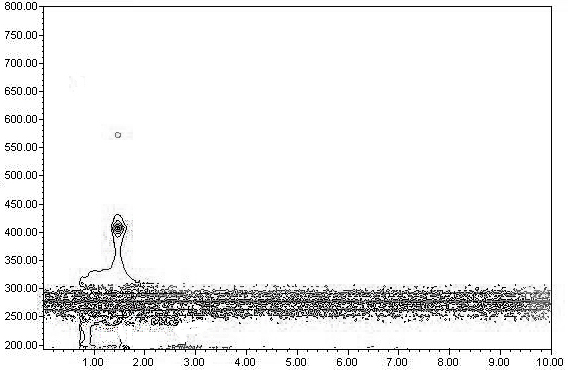


Time (min)

Wavelength (nm)

**A**


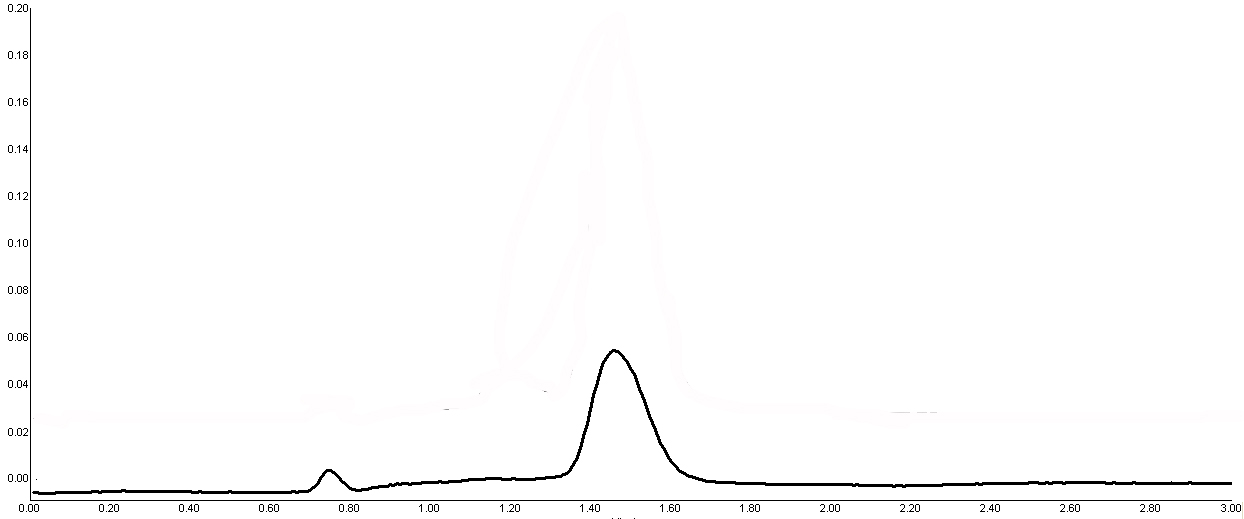


Vanadyl porphyrin

Chloroform

Abundance

Time (min)

**B**


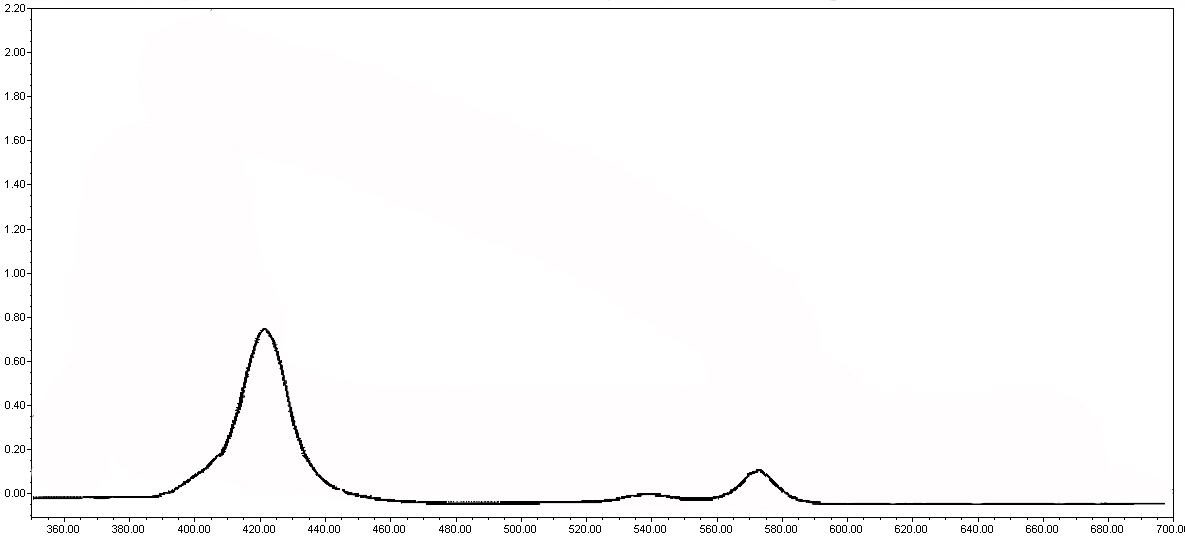


420 nm

538 nm

576 nm

Time (min)

Abundance

**C**

**Figure C.2.** High-performance liquid chromatography with photodiode array detector (HPLC-PDA): 3D chromatogram (A), 425 nm chromatogram (B), and UV-Vis spectrum (C) of bacteria-inhabited shale rock (BISR)


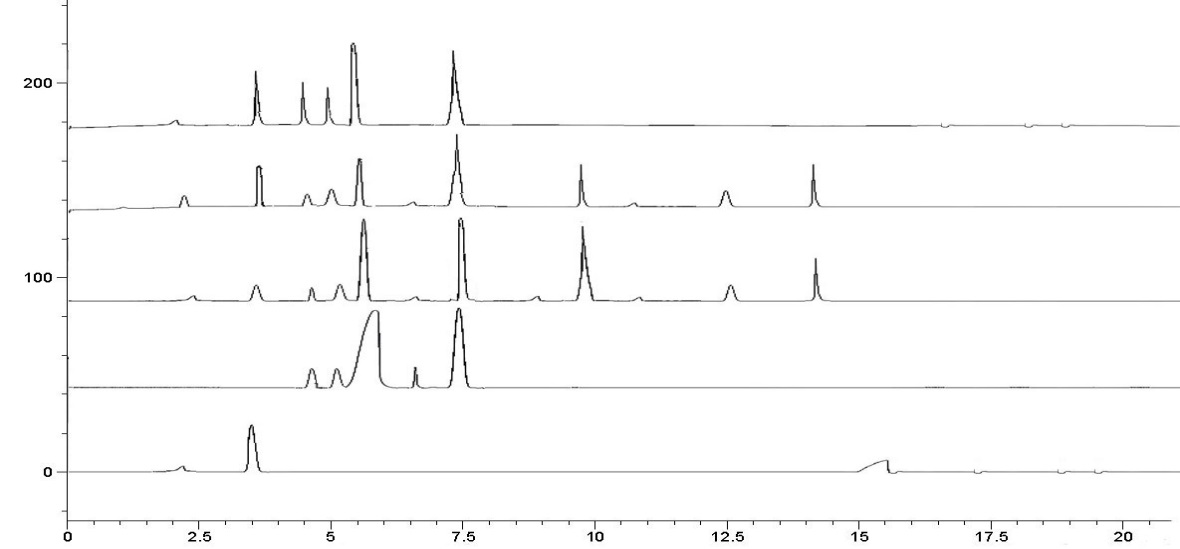


**N**

**H**

**C**

**V**O(MTPP)

Time (min)

Abundance

C_8_HxN_2_V

C_4_H_x_NNi

C_16_H_x_

Ni

C_24_H_x_N_4_V

C_14_H_x_N_2_Ni

**Ni**

C_6_H_x_N_2_V

C_44_H_x_N_4_V

C_4_H_x_V

C_12_H_x_

C_22_H_x_

C_22_H_x_

C

**Figure C.3.** The atomic emission spectrum of bacteria-inhabited shale rock (BISR)


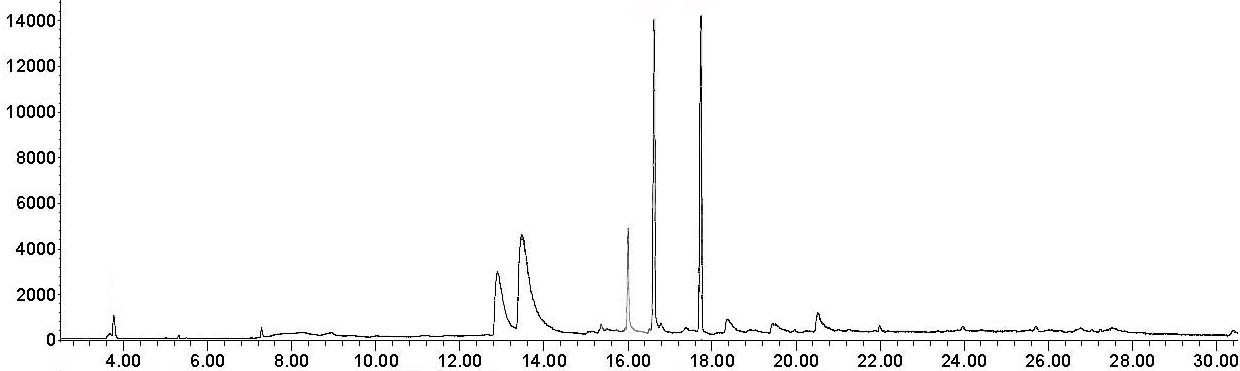


13.362


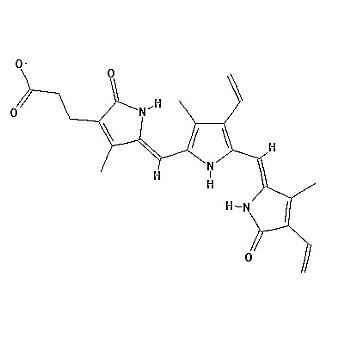


Time (min)

Abundance

**A**

13.667

16.011

16.201

17.906

C_29_H_31_N_3_O_6_

| **Retention time (min)**  **B** | **Organic compounds containing 3 pyrrole rings** | **Peak area (%)** | **Probability** |
| --- | --- | --- | --- |
| 13.362 | 3-[2-[[3-(2-Carboxyethyl)-5-[(3.4-dimethyl-5-oxopyrrol-2-ylidene)methyl]-4-methyl-1H-pyrrol-2-yl]methylidene]-4-methyl-5-oxopyrrol-3-yl]propanoic acid | 19.1 | 98 |
| 13.667 |  | 28.8 | 92 |
| 16.011 | 3-[(5Z)-5-[[4-Ethenyl-5-[(Z)-(4-ethenyl-3-methyl-5-oxopyrrol-2-ylidene)methyl]-3-methyl-1H-pyrrol-2-yl]methylidene]-4-methyl-2-oxopyrrol-3-yl]propanoate | 7.6 | 96 |
| 16.201 |  | 19.4 | 91 |
| 17.906 |  | 21.8 | 94 |

**Figure C.4.** Selected ion (*m*/*z*: 201) monitoring chromatogram of bacteria-inhabited shale rock (BISR) (A) and list of detected organic compounds containing 3 pyrrole rings (B)


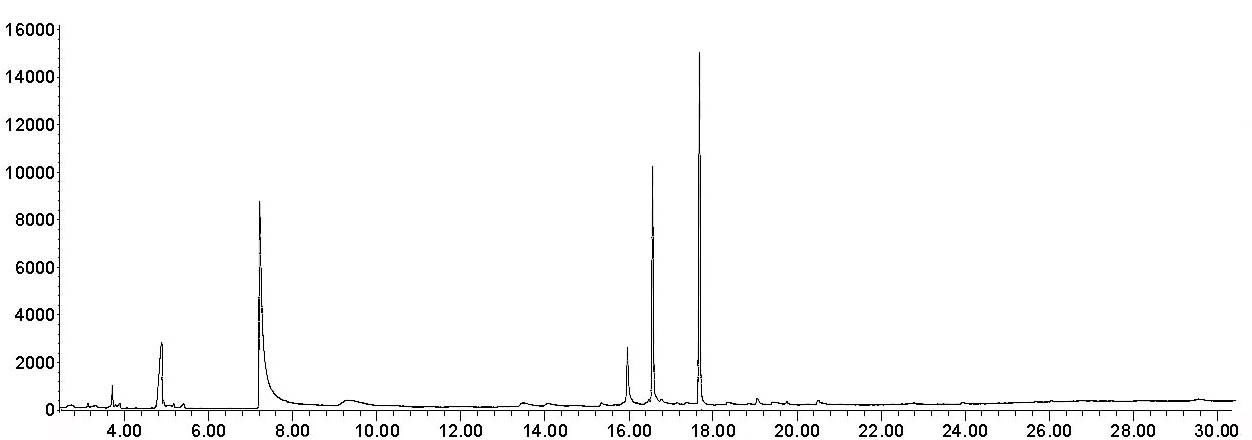


17.861

5.224

15.981

**A**


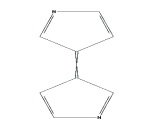


Time (min)

Abundance


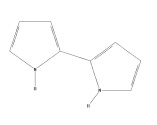


16.530

7.807


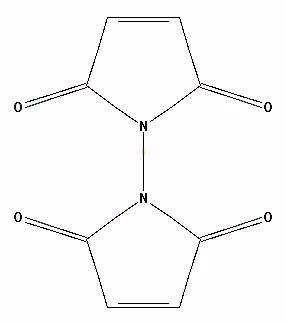


| **Retention time (min)**  **B** | **Organic compounds containing 2 pyrrole rings** | **Peak area (%)** | **Probability** |
| --- | --- | --- | --- |
| 5.224 | 3.3'-Bipyrrole | 9.7 | 91 |
| 7.807 | 2.2'-Bipyrrole | 25.1 | 96 |
| 15.981 | 1.1'-Bipyrrole-2.2'.5.5'-tetraone | 6.8 | 94 |
| 16.530 |  | 24.2 | 98 |
| 17.861 |  | 33.8 | 96 |

**Figure C.5.** Selected ion (*m*/*z*: 134) monitoring chromatogram of bacteria-inhabited shale rock (BISR) (A) and list of detected organic compounds containing 2 pyrrole rings (B)

**B**


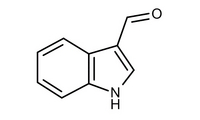


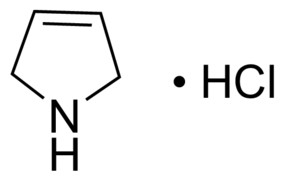


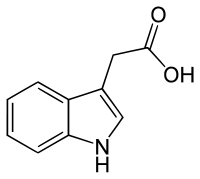


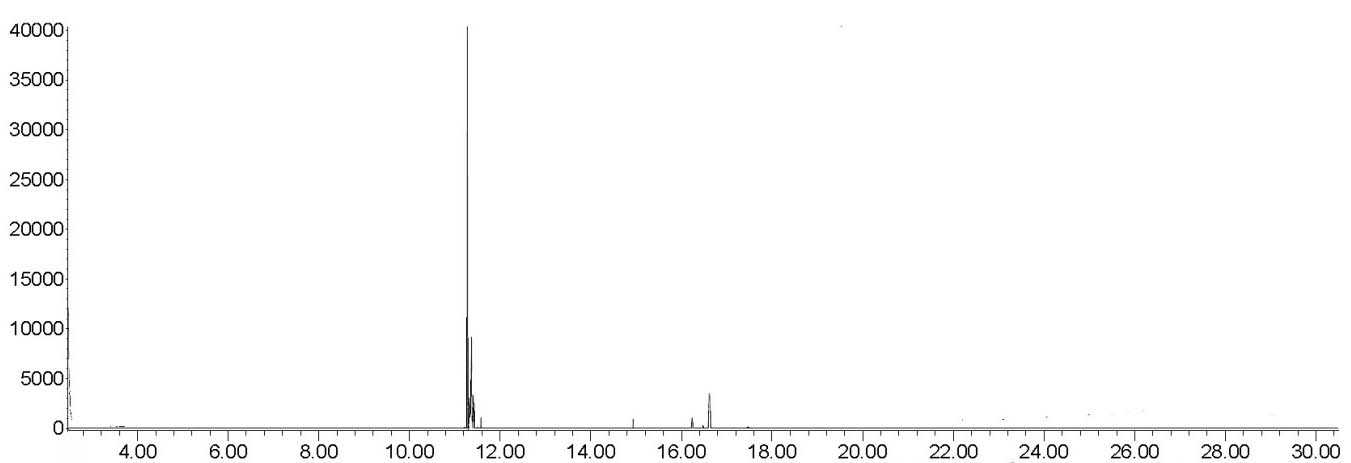


16.290

11.818

11.781

16.081

Time (min)

Abundance

**A**

| **Retention time (min)** | **Organic compounds containing 1 pyrrole ring** | **Peak area (%)** | **Probability** |
| --- | --- | --- | --- |
| 11.781 | 1H-Pyrrole | 43.8 | 96 |
| 11.818 |  | 26.2 | 98 |
| 16.081 | Indole acetic acid | 1.2 | 91 |
| 16.290 | Indole carbaldehyde | 5.8 | 93 |

**Figure C.6.** Selected ion (*m*/*z*: 67) monitoring chromatogram of bacteria-inhabited shale rock (BISR) (A) and list of detected organic compounds containing 1 pyrrole ring (B)
