# Supplementary material for: Postdiagenetic Bacterial Transformation of Nickel and Vanadyl Sedimentary Porphyrins of Organic-Rich Shale Rock (Fore-Sudetic Monocline, Poland)
Source: Front Microbiol. 2021 Nov 30;12:772007. doi: 10.3389/fmicb.2021.772007 (PMC8669743; doi:10.3389/fmicb.2021.772007)
Supplement: Supplementary file 4 [file Table_4.DOCX]

**Supplementary Material D. Supplementary results for the culture of strain LM27 on shale rock (SR-BC) and sterile control (SR-SC)**

| **Parameter** | **SR-BC** |
| --- | --- |
| CFU duplication time 0-30 days | 7.27 |
| Max. CFU/ml 0-30 days | 21x10^6^ |

**Figure D.1.** Growth of strain LM27 on mineral medium supplemented with shale rock (SR)

**Figure D.2.** Selected ions (*m*/*z*: 679, 599, 591, 528, 481, 472, 368, and 361) monitoring chromatograms of SR-BC


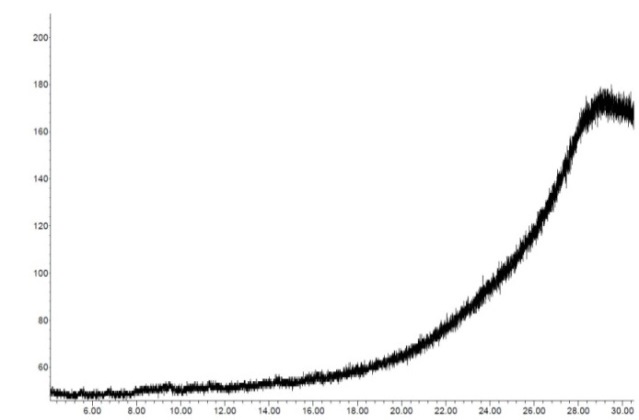


*m/z:* 361 - total peak area: 0

Time (min)

Abundance


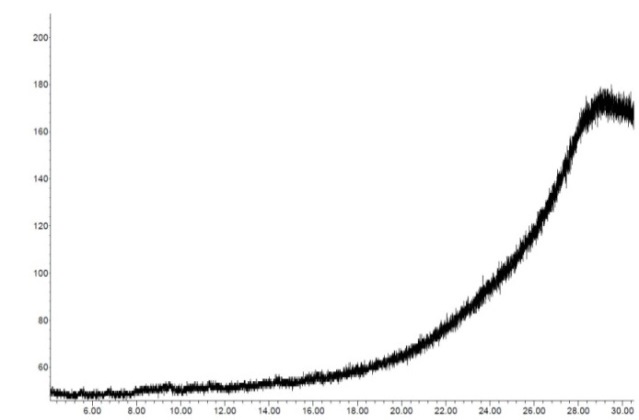


*m/z:* 368 - total peak area: 0

Time (min)

Abundance


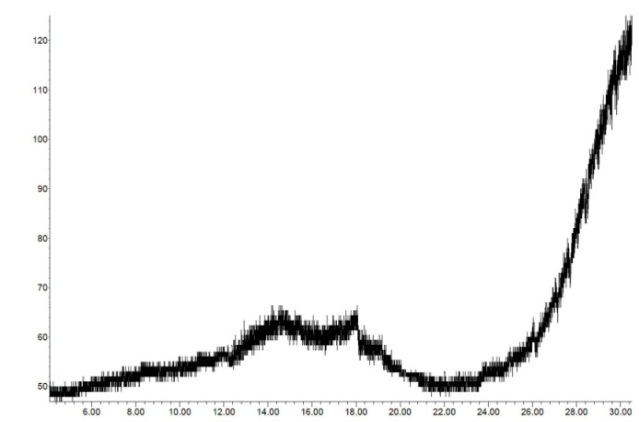


*m/z:* 481 - total peak area: 0

Abundance


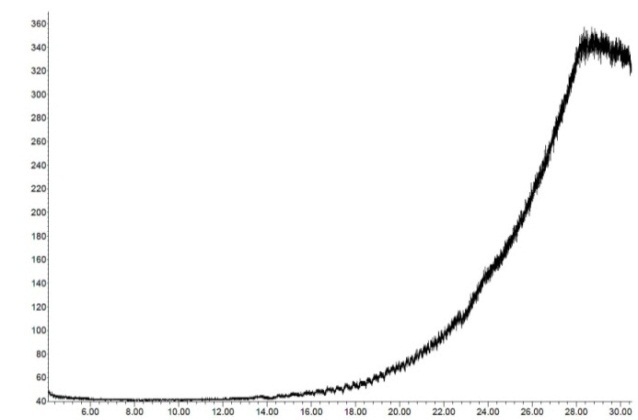


*m/z:* 472- total peak area: 0

Abundance


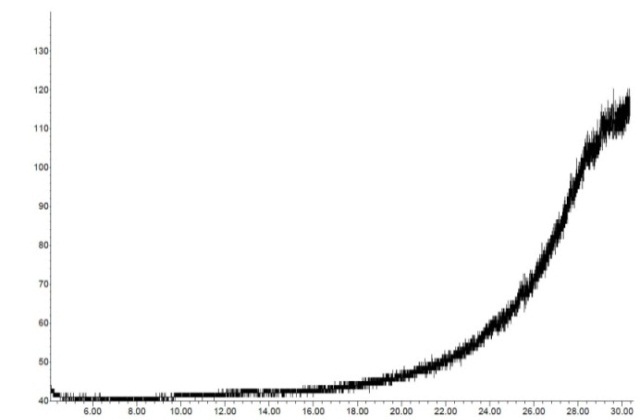


*m/z:* 599 - total peak area: 0

Abundance


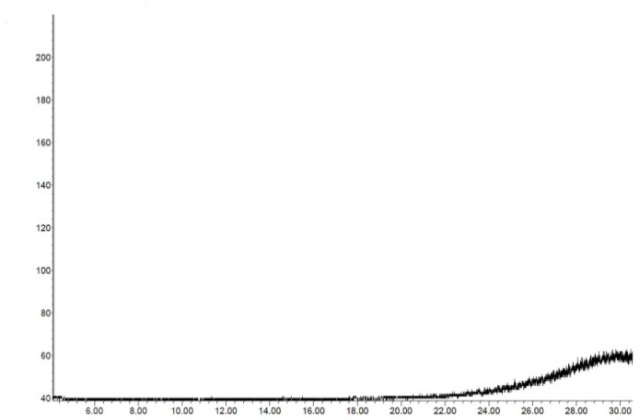


*m/z:* 591- total peak area: 0

Abundance


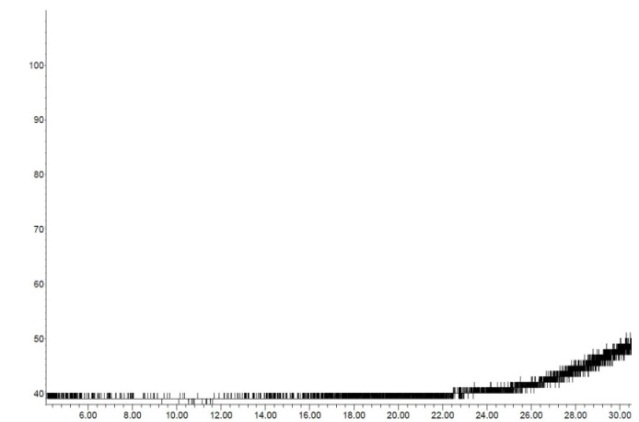


*m/z:* 679 - total peak area: 0

Abundance


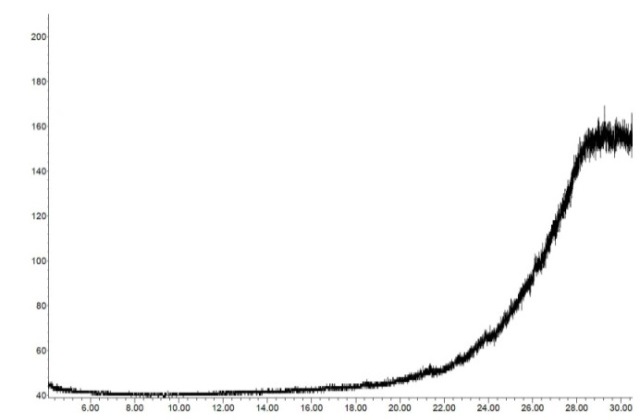


*m/z:* 528 - total peak area: 0

Abundance

**Figure D.3.** High-performance liquid chromatography with photodiode array detector (HPLC-PDA): 3D chromatogram (A), 245 nm chromatogram (B), and UV-Vis spectrum (C) of SR-BC

**A**


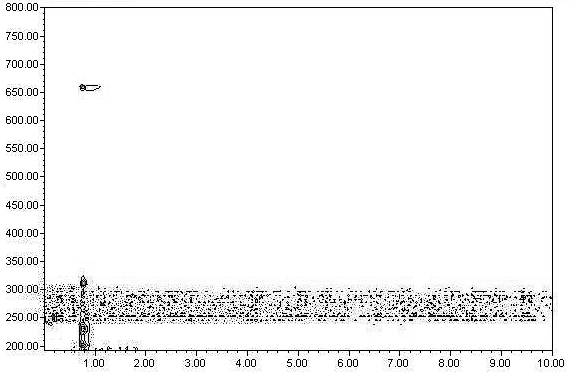


Time (min)

Wavelength (nm)

**C**


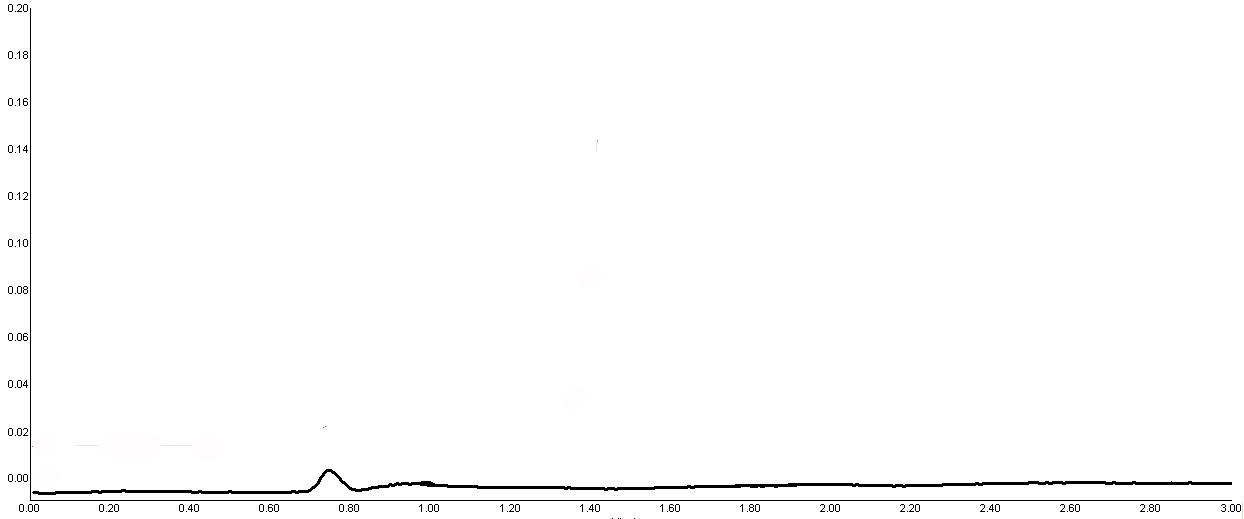


Chloroform

Abundance

Time (min)

**B**


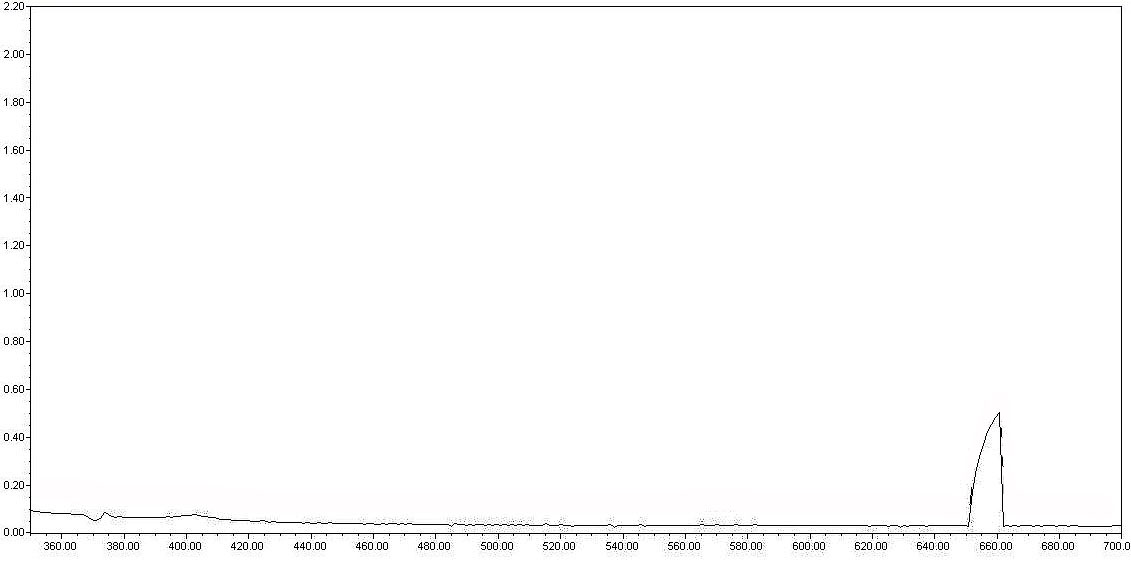


660 nm

Wavelenth (nm)

Abundance

**B**

**A**


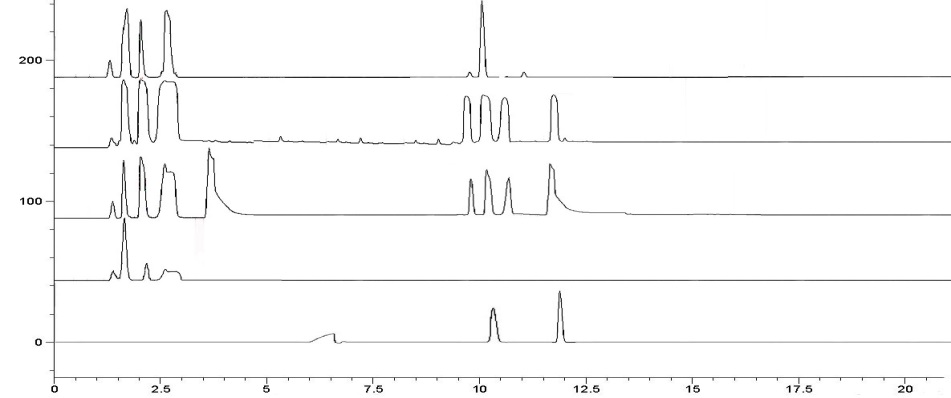


**N**

**H**

**C**

**V**

Time (min)

Abundance

C_8_HxN_2_V

C_10_H_x_Ni

C_8_H_x_N

Ni

C_5_H_x_NV

C_12_H_x_N_2_Ni

**Ni**

C_4_H_x_NV

C_12_H_x_

C

C_12_H_x_N_2_V


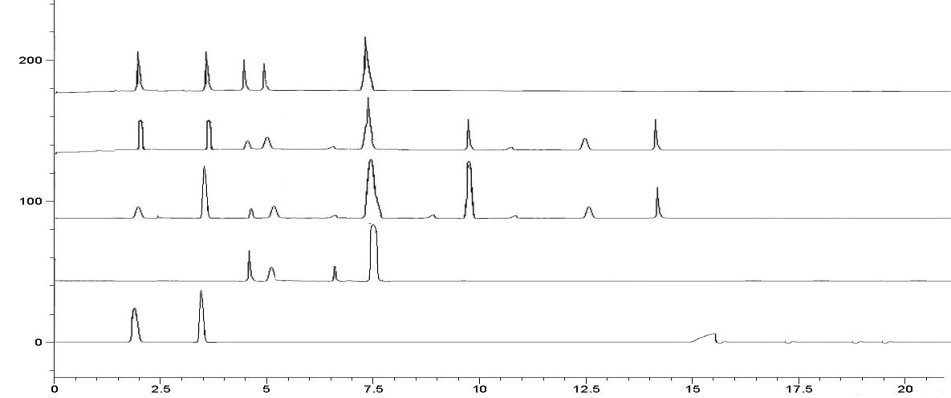


**N**

**H**

**C**

**V**

Time (min)

Abundance

C_14_H_x_NNi

Ni

C_18_H_x_N_2_V

C_8_H_x_N_2_Ni

**Ni**

C_4_H_x_V

C_22_H_x_

C_22_H_x_

C_22_H_x_

C_16_H_x_NNi

C_18_H_x_NNi

**Figure D.4.** The atomic emission spectra of aqueous phase (A) and sediment (B) of SR-BC


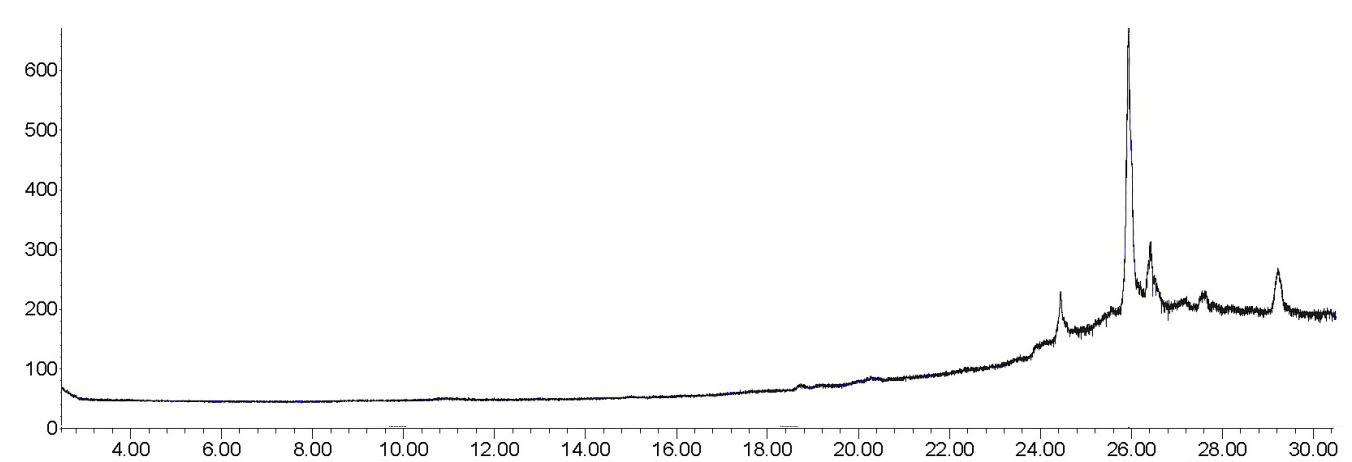


25.986


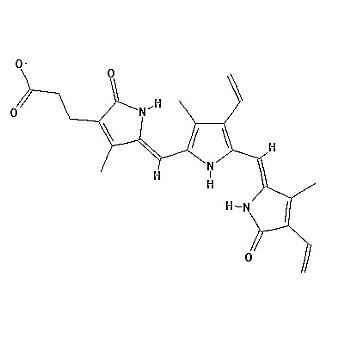


Time (min)

Abundance

**A**

**B**

| **Retention time (min)** | **Organic compounds containing 3 pyrrole rings** | **Peak area (%)** | **Probability** |
| --- | --- | --- | --- |
| 25.986 | 3-[(5Z)-5-[[4-Ethenyl-5-[(Z)-(4-ethenyl-3-methyl-5-oxopyrrol-2-ylidene)methyl]-3-methyl-1H-pyrrol-2-yl]methylidene]-4-methyl-2-oxopyrrol-3-yl]propanoate | 90,6 | 98 |

**Figure D.5.** Selected ion (*m*/*z*: 201) monitoring chromatogram of SR-BC (A) and list of detected organic compounds containing 3 pyrrole rings (B)


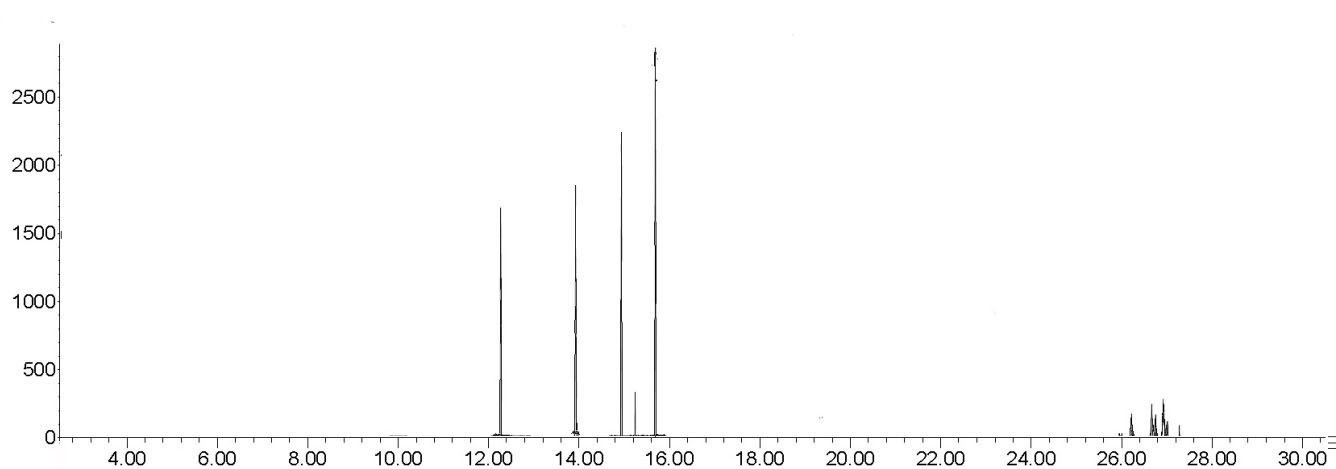


13.988

15.137

12.114

**A**

26.592


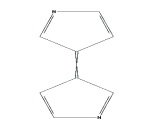


Time (min)

Abundance


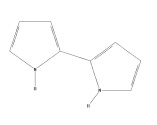


15.328

15.684


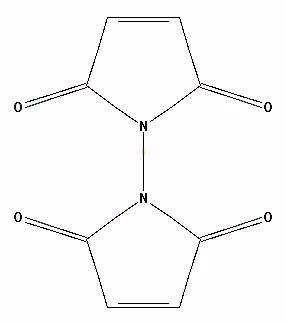


| **Retention time (min)**  **B** | **Organic compounds containing 2 pyrrole rings** | **Peak area (%)** | **Probability** |
| --- | --- | --- | --- |
| 12.114 | 3,3'-Bipyrrole | 11.0 | 98 |
| 13.988 |  | 8.7 | 99 |
| 15.137 | 2,2'-Bipyrrole | 13.7 | 91 |
| 15.328 |  | 2.3 | 92 |
| 15.684 |  | 17.2 | 90 |
| 26.592 | 3,3',4,4'-Tetramethyl-1H,1'H-2,2'-bipyrrole-5,5'-dicarboxylic acid | 7.6 | 94 |

**Figure D.6.** Selected ion (*m*/*z*: 134) monitoring chromatogram of SR-BC (A) and list of detected organic compounds containing 2 pyrrole rings (B)

**B**


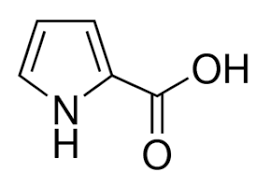


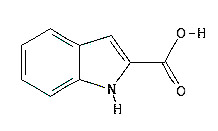


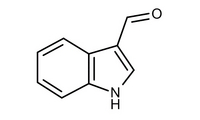


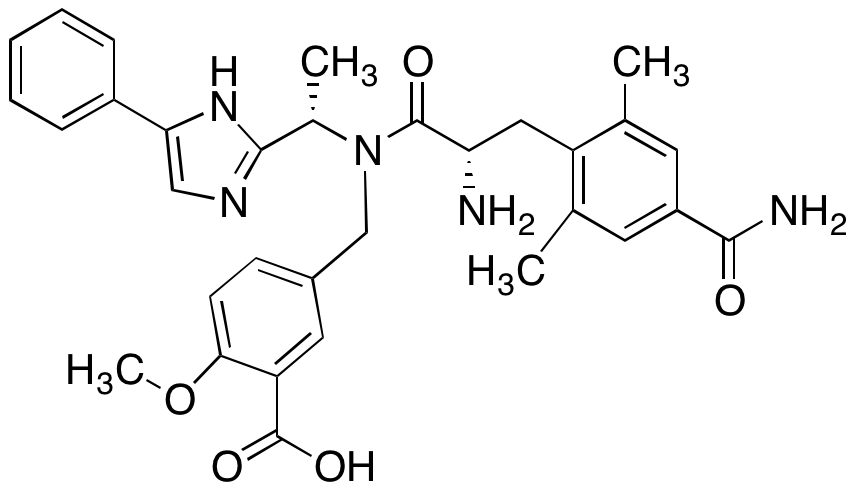


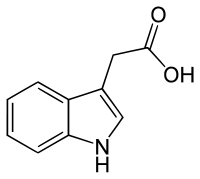


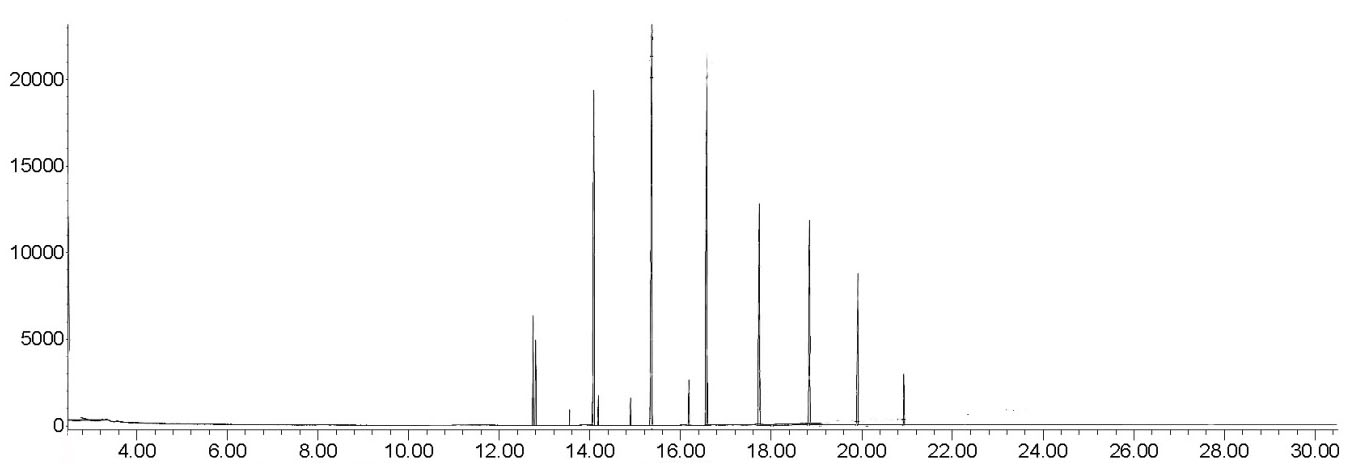


12.993

16.224

Time (min)

Abundance

17.834

20.266

19.988

**A**

18.897

13.006

14.066

15.201

16.554

| **Retention time (min)** | **Organic compounds containing 1 pyrrole ring** | **Peak area (%)** | **Probability** |
| --- | --- | --- | --- |
| 12.993 | 1H-Pyrrole-2-carboxylic acid | 3.4 | 91 |
| 13.006 |  | 2.1 | 94 |
| 14.066 | Indole acetic acid | 14.8 | 96 |
| 15.201 |  | 14.2 | 98 |
| 16.224 |  | 3.8 | 97 |
| 16.554 |  | 12.1 | 90 |
| 17.834 | 1H-Indole-2-carboxylic acid | 8.2 | 93 |
| 18.897 | Indole carbaldehyde | 7.8 | 91 |
| 19.988 |  | 5.2 | 91 |
| 20.266 | 2H-Pyrrol-2-one, 5-[[2-[(4-aminophenyl)methylene]-3,4-dimethyll]methylene]-3-ethyl-1,5-dihydro-4-methyl | 1.7 | 90 |

**Figure D.7.** Selected ion (*m*/*z*: 67) monitoring chromatogram of SR-BC (A) and list of detected organic compounds containing 1 pyrrole ring (B)

**Figure D.8.** Selected ions (*m*/*z*: 679, 599, 591, 528, 481, 472, 368, and 361) monitoring chromatograms of SR-SC


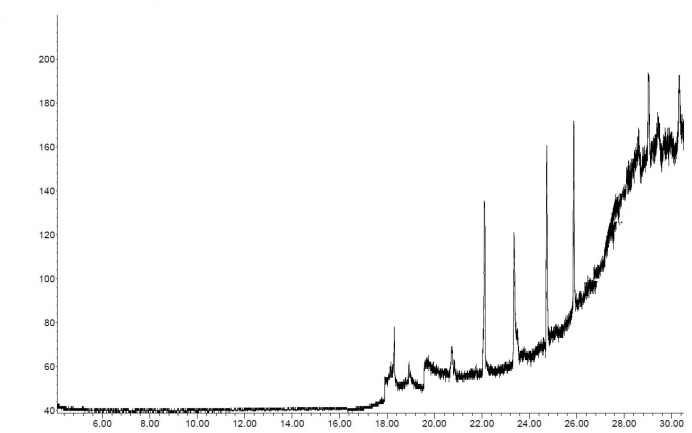


*m/z:* 591- total peak area: 28064

Abundance


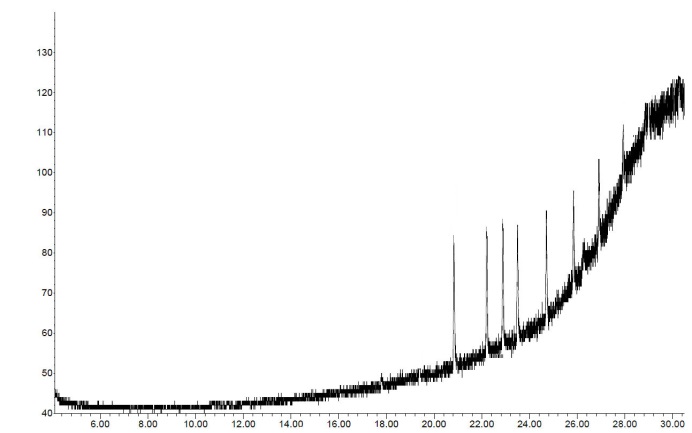


*m/z:* 599 - total peak area: 11108

Abundance


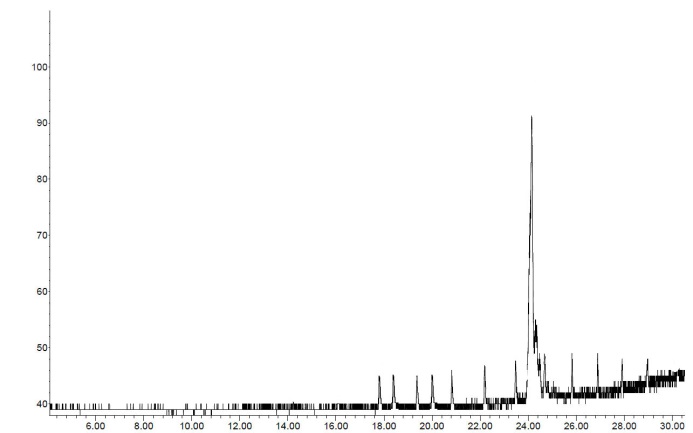


*m/z:* 679 - total peak area: 8631

Abundance


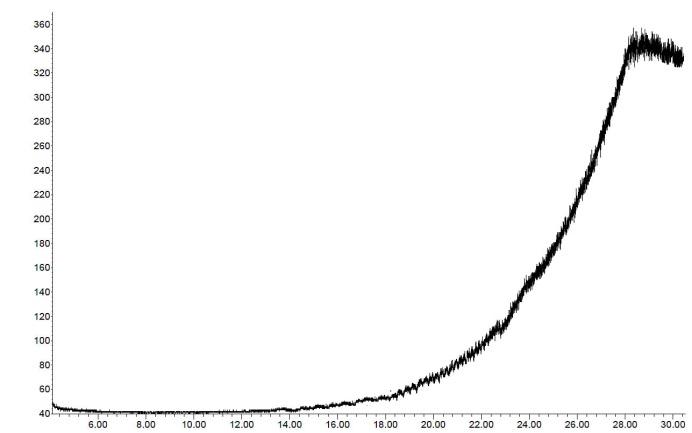


*m/z:* 472 - total peak area: 0

Abundance


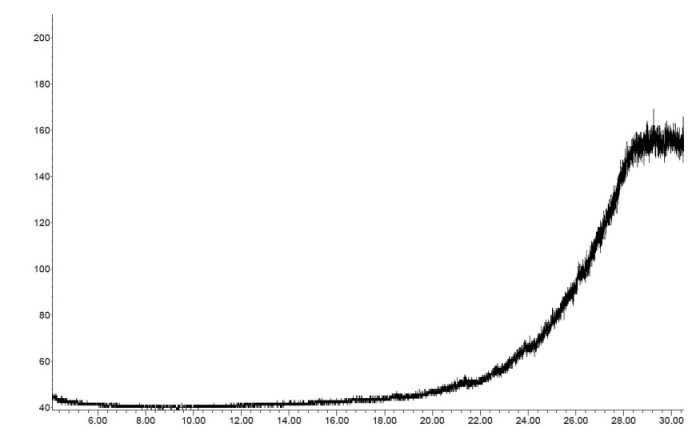


*m/z:* 528 - total peak area: 0

Abundance


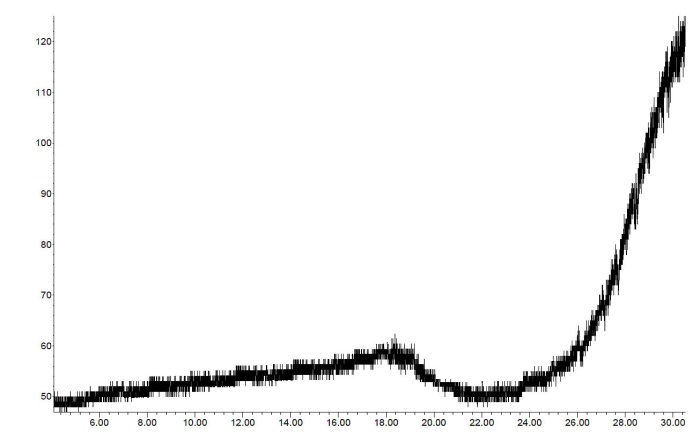


*m/z:* 481 - total peak area: 0

Abundance


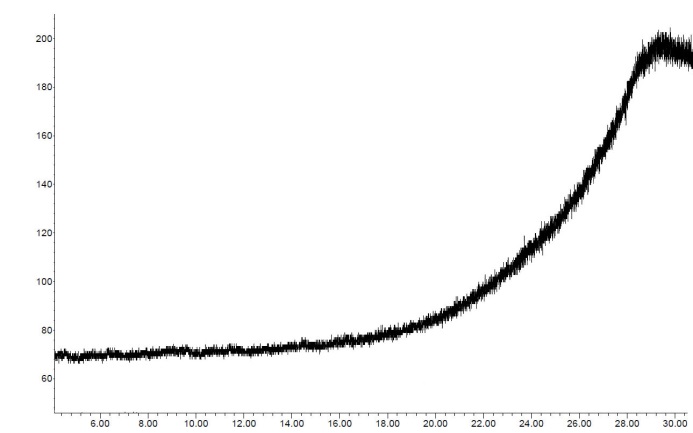


*m/z:* 361 - total peak area: 0

Time (min)

Abundance


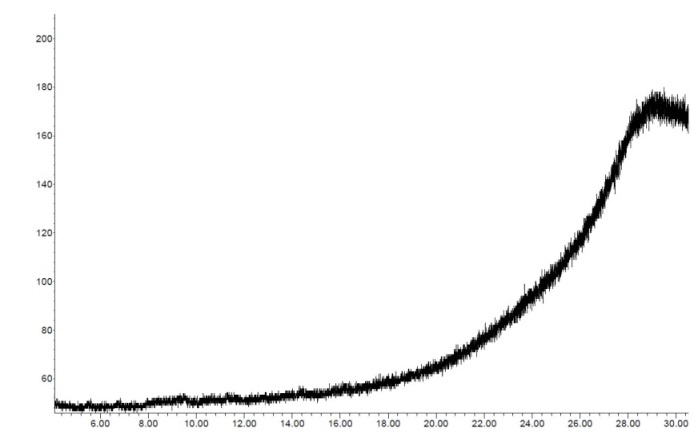


*m/z:* 368 - total peak area: 0

Time (min)

Abundance

**Figure D.9.** High-performance liquid chromatography with photodiode array detector (HPLC-PDA): 3D chromatogram (A), 425 nm chromatogram (B), and UV-Vis spectra (C) of SR-SC

**A**


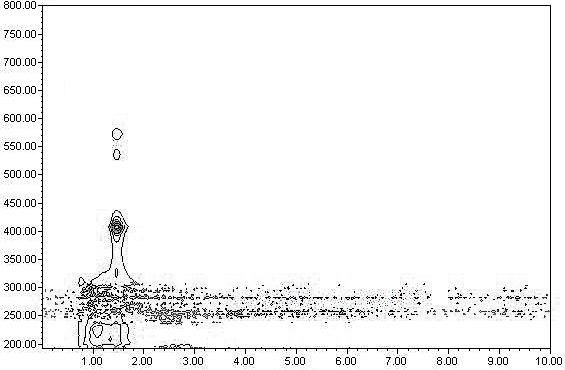


Time (min)

Wavelength (nm)


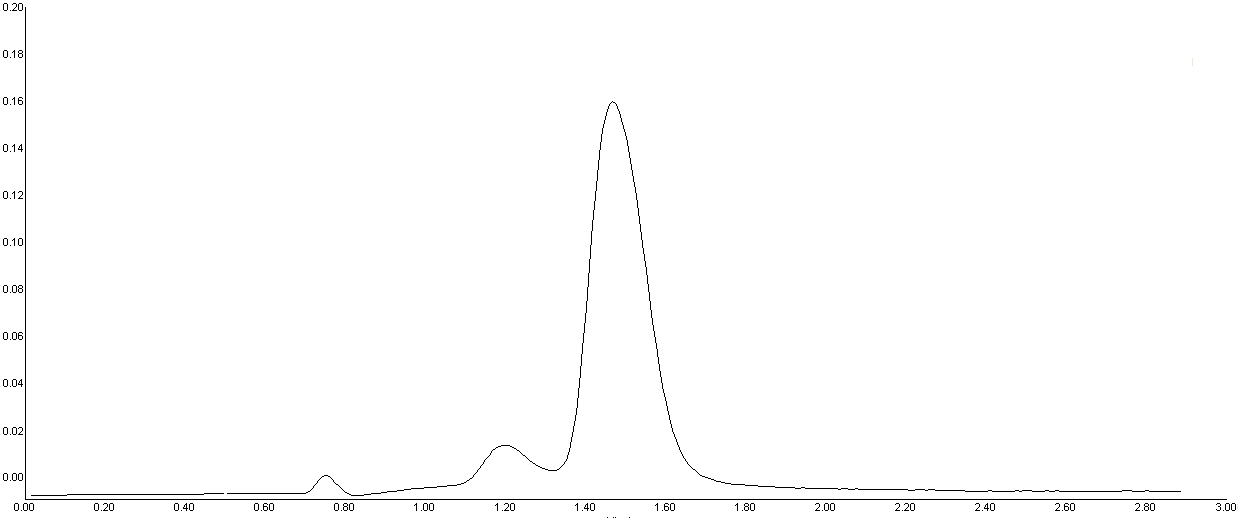


Vanadyl porphyrins

Nickel

porphyrins

Chloroform

Abundance

Time (min)

**C**

**B**

nickiel porphyrine SR - SC

vanadyl porphyrine SR -SC


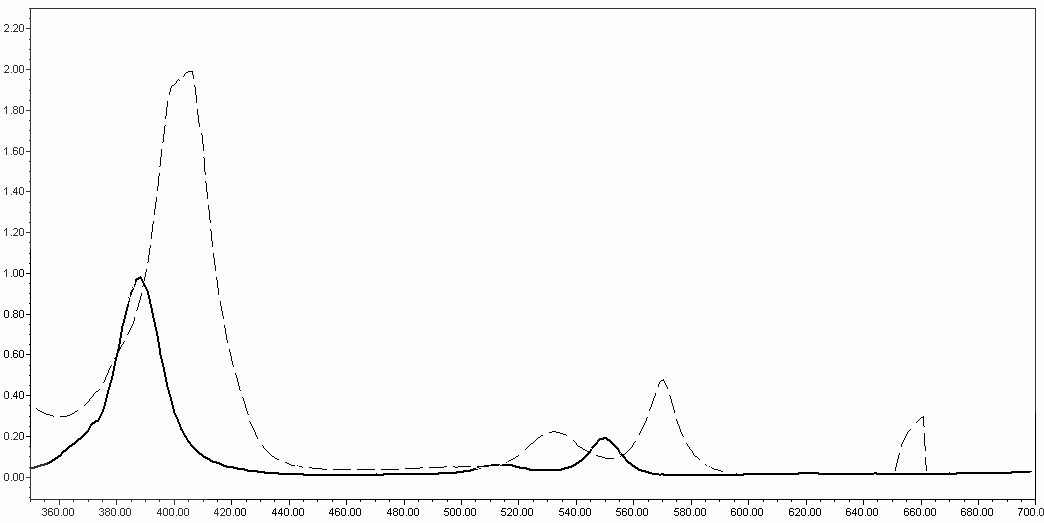


380 nm

401 nm

529 nm

571 nm

516 nm

549 nm

660 nm

Wavelenth (nm)

Abundance

*m/z:* 67 - total peak area: 0

*m/z:* 134 - total peak area: 0

**B**

**A**

vanadyl porphyrine

nickel porphyrine

nickel porphyrine

vanadyl porphyrine

**A**

**B**

**C**


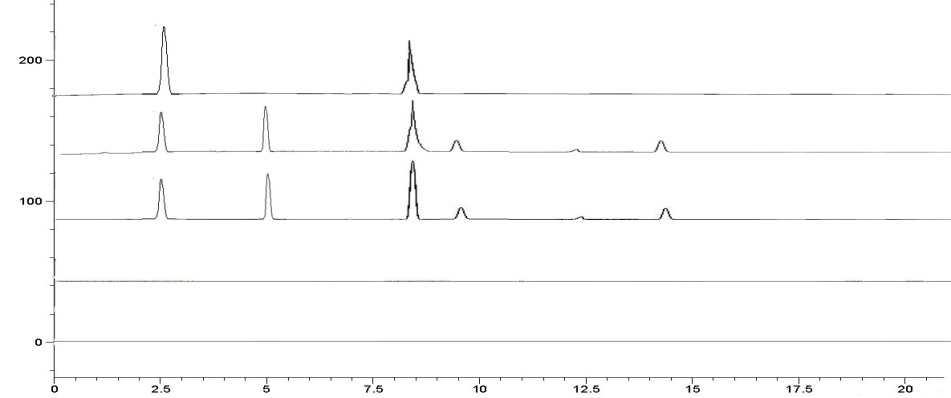


**N**

**H**

**C**

**V**

Time (min)

Abundance

**Ni**

C_19_H_x_N

C_12_H_x_

C_20_H_x_

C_26_H_x_

C_8_H_x_N


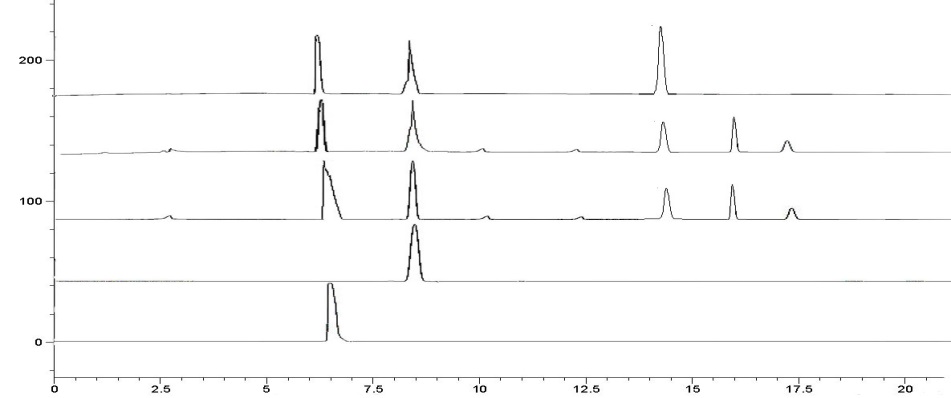


**N**

**H**

**C**

**V**

Time (min)

Abundance

C_44_H_x_N_4_V

**Ni**

C_31_H_x_N_4_Ni

C_42_H_x_

C_30_H_x_

C_36_H_x_

**Figure D.10.** The atomic emission spectra of aqueous phase (A) and sediment (B) of SR-SC

*m/z:* 201 - total peak area: 0


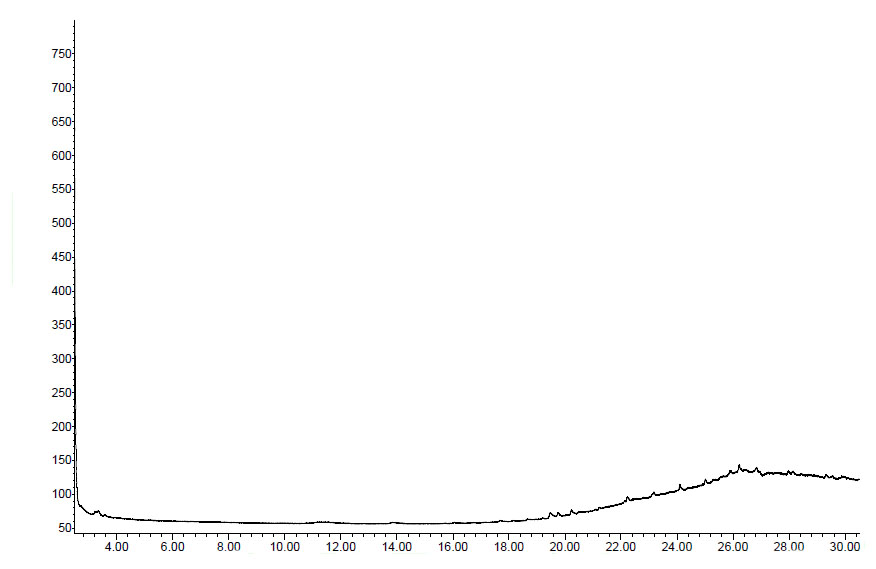


Abundance

Time (min)


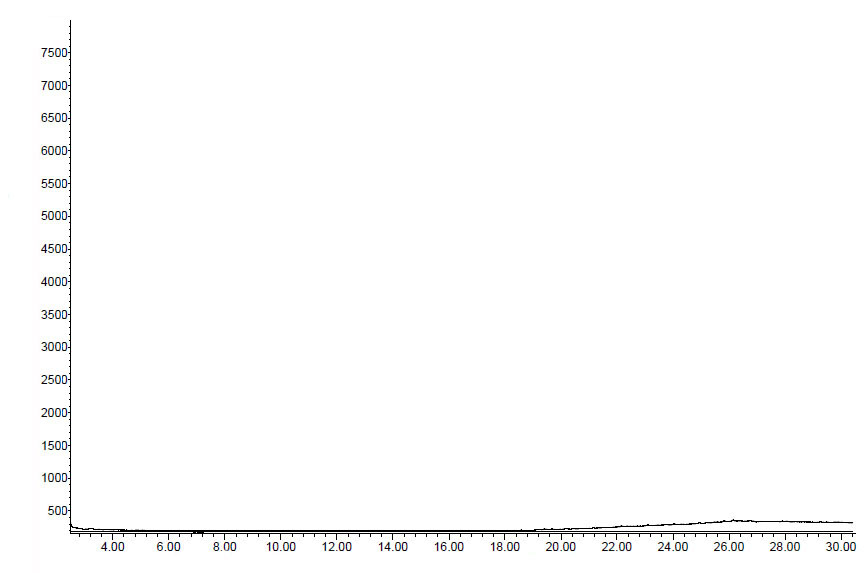


Abundance

Time (min)


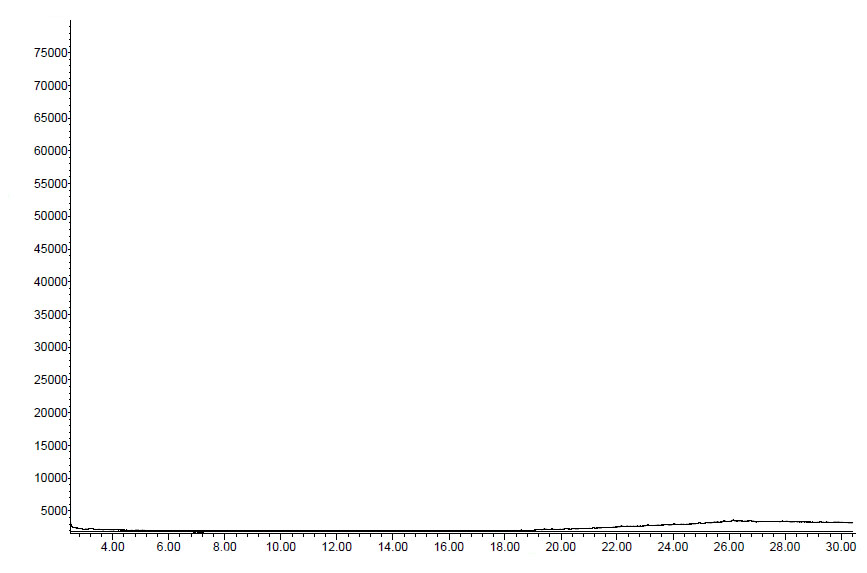


Abundance

Time (min)

**Figure D.11.** Selected ions monitoring chromatograms: *m*/*z*: 201 (organic compounds containing 3 pyrrole rings), *m*/*z*: 134 (organic compounds containing 2 pyrrole rings), and *m*/*z*: 67 (organic compounds containing 1 pyrrole ring) of SR-SC
