# Supplementary material for: Postdiagenetic Bacterial Transformation of Nickel and Vanadyl Sedimentary Porphyrins of Organic-Rich Shale Rock (Fore-Sudetic Monocline, Poland)
Source: Front Microbiol. 2021 Nov 30;12:772007. doi: 10.3389/fmicb.2021.772007 (PMC8669743; doi:10.3389/fmicb.2021.772007)
Supplement: Supplementary file 5 [file Table_5.DOCX]

**Supplementary Material E. Supplementary results for the culture of strain LM27 on octaethyl nickel porphyrin (Ni(OEP**)**-BC) and sterile control (Ni(OEP)-SC)**

**B**

**A**

| Parameter | Ni(OEP)-BC |
| --- | --- |
| CFU duplication time (days) | 2.37 |
| Maximal CFU/ml | 18.5x10^6^ |

**Figure E.1.** Growth of strain LM27 on medium supplemented with Ni(OEP): growth curve (A), duplication time and maximal CFU (B)

**A**


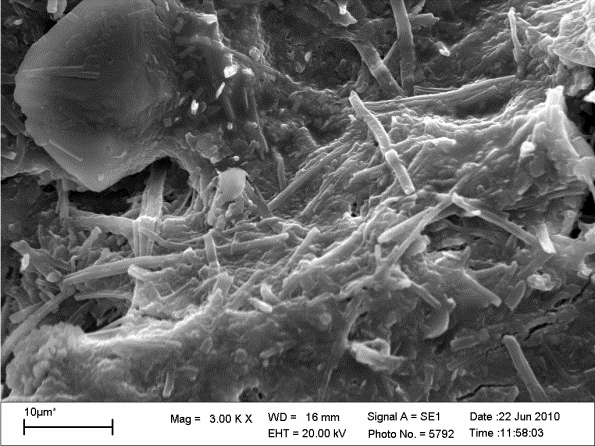

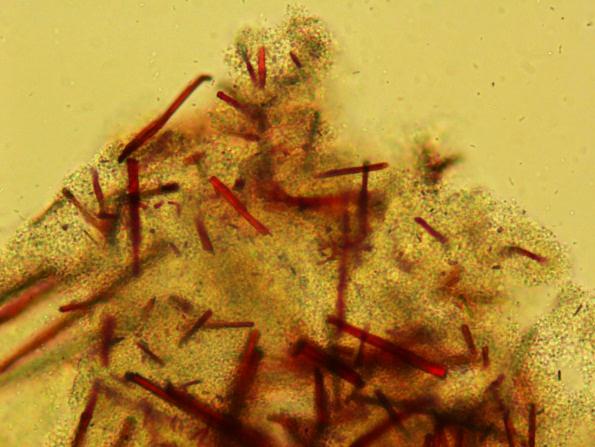

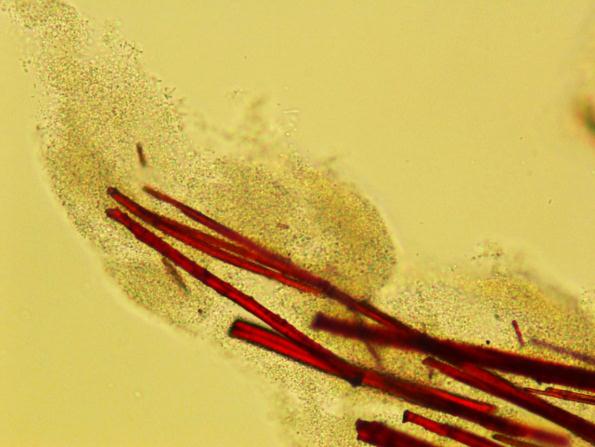

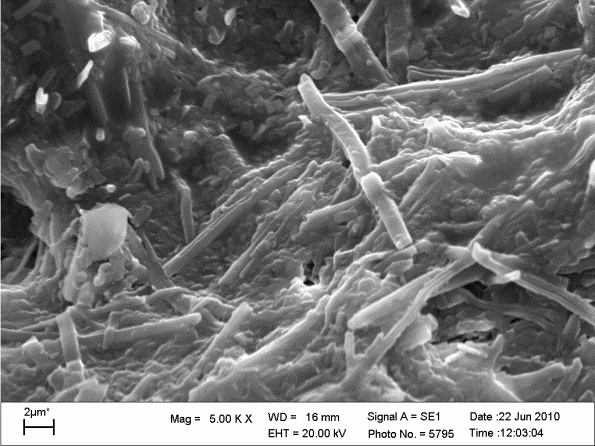

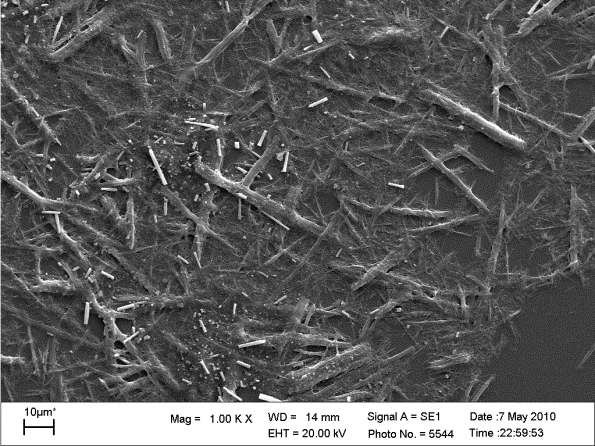


**A**

**B**

**C**

**D**

**E**

**Figure E.2.** Biofilm of strain LM27 on Ni(OEP)): light (A, B) and scanning electron (C, D, E) microphotographs


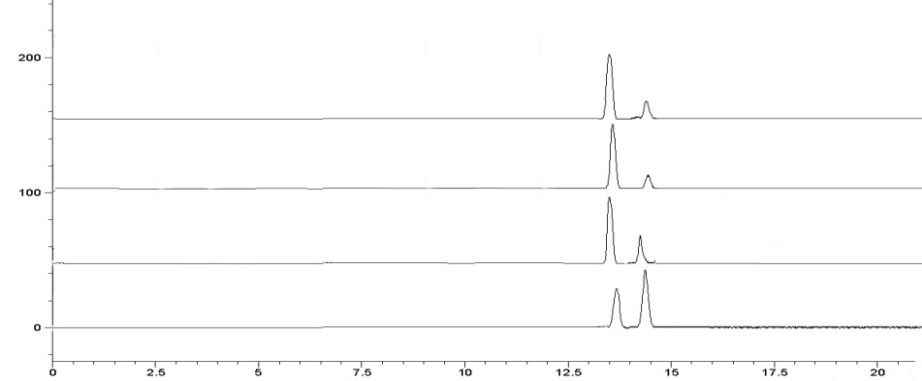


**N**

**H**

**C**

**B**

**Ni**

Time (min)

Abundance

C_15_H_x_NNi

C_20_H_x_N_4_Ni


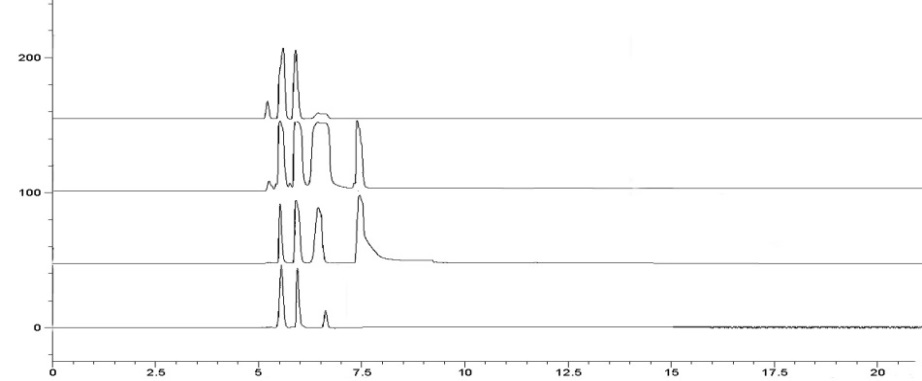


**N**

**H**

**C**

**A**

**Ni**

Time (min)

Abundance

C_5_H_x_NNi

C_5_H_x_N_2_Ni

C_10_H_x_N_2_Ni

CHN

C_12_H_x_

**Figure E.3.** Atomic emission spectra of aqueous phase (A) and sediment (B) of Ni(OEP)-BC


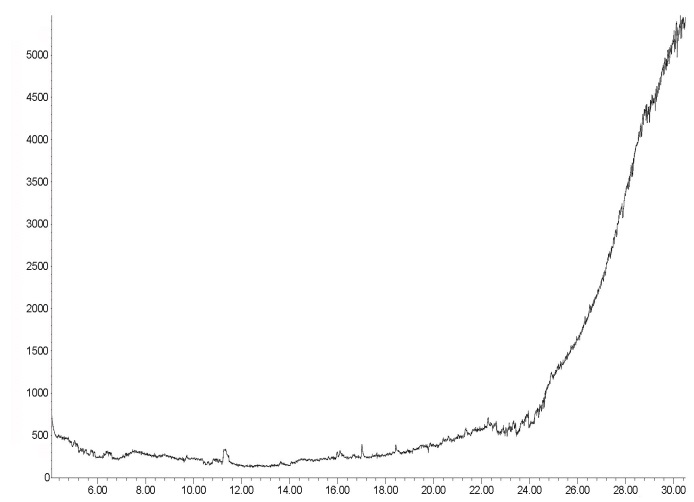


*m/z:* 591 - total peak area: 0

Time (min)

Abundance


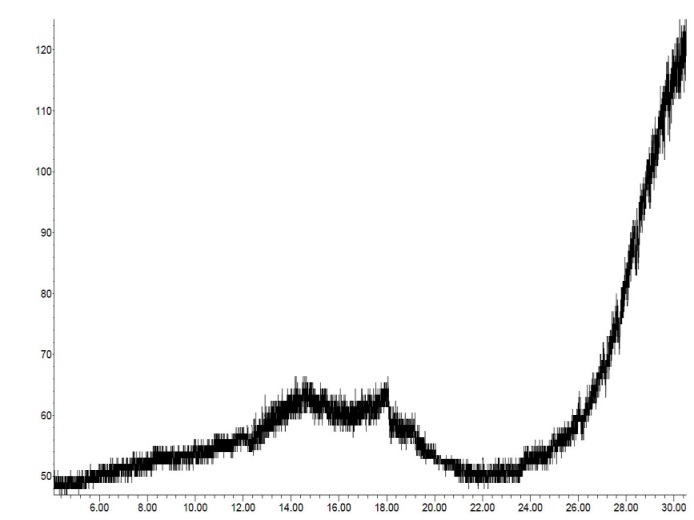


*m/z:*481 - total peak area: 0

Time (min)

Abundance


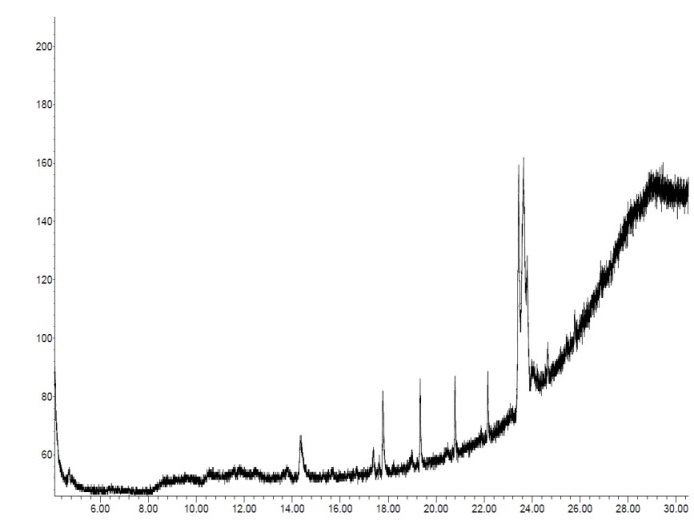


*m/z:*368 - total peak area: 42641

Time (min)

Abundance

**Figure E.4.** Selected ion monitoring chromatograms: *m*/*z*: 368 (NiP), *m*/*z*: 481 (Ni(TEP)), and *m*/*z*: 591 (Ni(OEP) of Ni(OEP)-BC


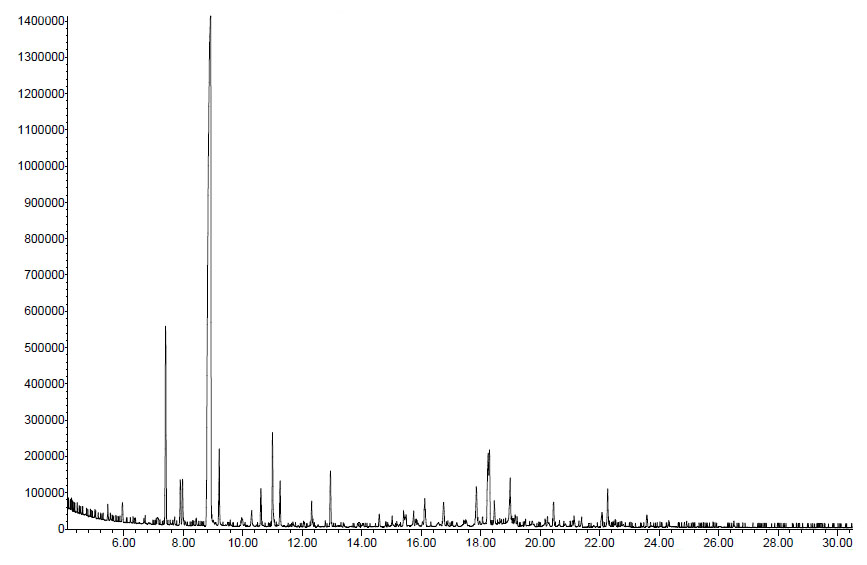


7.901


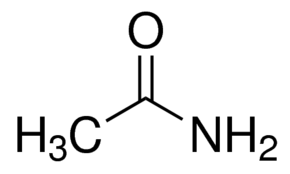


Time (min)

Abundance

**A**

7.409

7.901

11.248


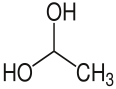


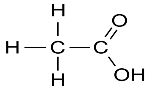


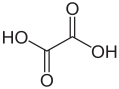


**B**

| **Retention time (min)** | **Organic compounds containing ethyl group** | **Peak area (%)** | **Probability** |
| --- | --- | --- | --- |
| 7.409 | Ethanimidic acid | 7.1 | 99 |
| 7.901 | Ethanoic acid | 1.54 | 97 |
| 7.969 | Ethanedioic acid | 1.53 | 72 |
| 11.248 | Ethane-2,2-diol | 1.64 | 78 |

**Figure E.5.** Selected ion (*m*/*z*: 45) monitoring chromatogram of Ni(OEP)-BC (A) and list of detected organic compounds containing ethyl group (B)

.


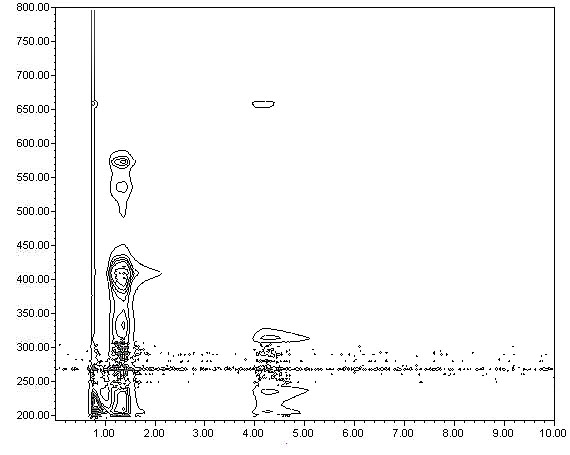


Time (min)

Wavelength (nm)

**A**


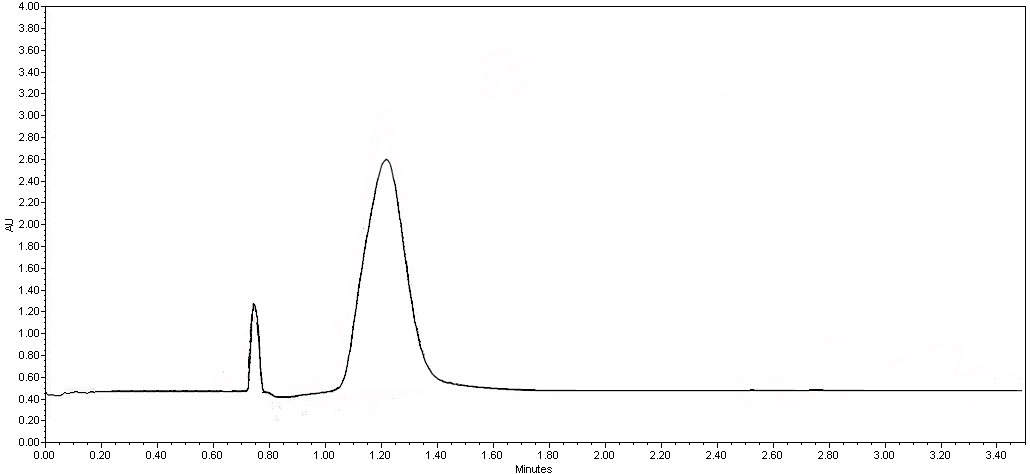


Ni(OEP)-BC

Chloroform

Abundance

Time (min)

**B**

**Figure E.6.** High-performance liquid chromatography with photodiode array detector (HPLC-PDA): 3D chromatogram (A), 425 nm chromatogram (B) and UV-Vis spectrum, (C) of Ni(OEP)-BC


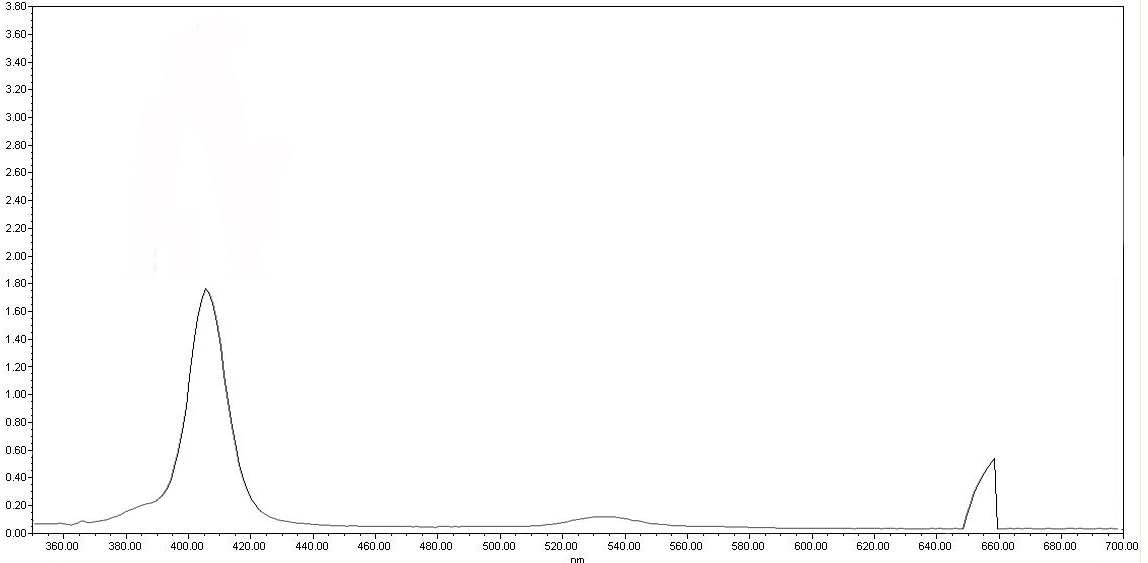


405 nm

550 nm

660 nm

Wavelength (nm)

Abundance

**C**

**B**


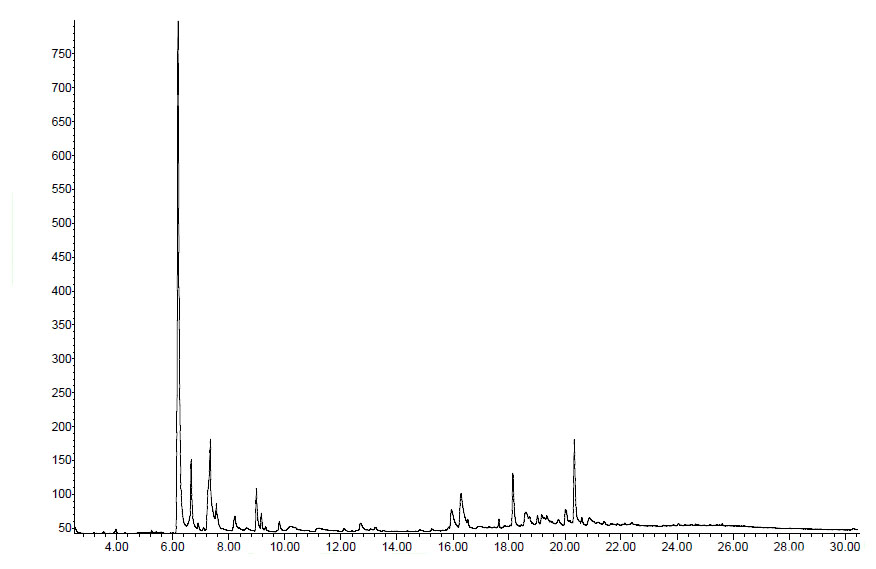


6.667


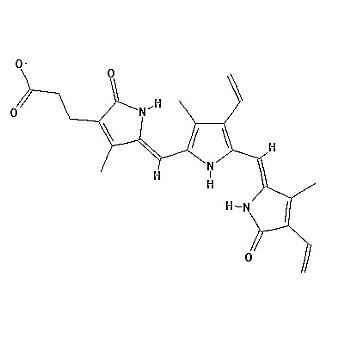


Time (min)

Abundance

**A**

16.011

20.906

6.362

6.698

C_29_H_31_N_3_O_6_

| **Retention time (min)** | **Organic compounds containing 3 pyrrole rings** | **Peak area (%)** | **Probabilty** |
| --- | --- | --- | --- |
| 6.362 | 3-[2-[[3-(2-Carboxyethyl)-5-[(3,4-dimethyl-5-oxopyrrol-2-ylidene)methyl]-4-methyl-1H-pyrrol-2-yl]methylidene]-4-methyl-5-oxopyrrol-3-yl]propanoic acid | 35.6 | 80 |
| 6.667 |  | 16.6 | 78 |
| 6.928 |  | 19.8 | 81 |
| 16.011 | 3-[(5Z)-5-[[4-Ethenyl-5-[(Z)-(4-ethenyl-3-methyl-5-oxopyrrol-2-ylidene)methyl]-3-methyl-1H-pyrrol-2-yl]methylidene]-4-methyl-2-oxopyrrol-3-yl]propanoate | 10.6 | 95 |
| 20.906 |  | 11.8 | 84 |

**Figure E.7.** Selected ion (*m*/*z*: 201) monitoring chromatogram of Ni(OEP)-BC (A) and list of detected organic compounds containing 3 pyrrole rings (B)


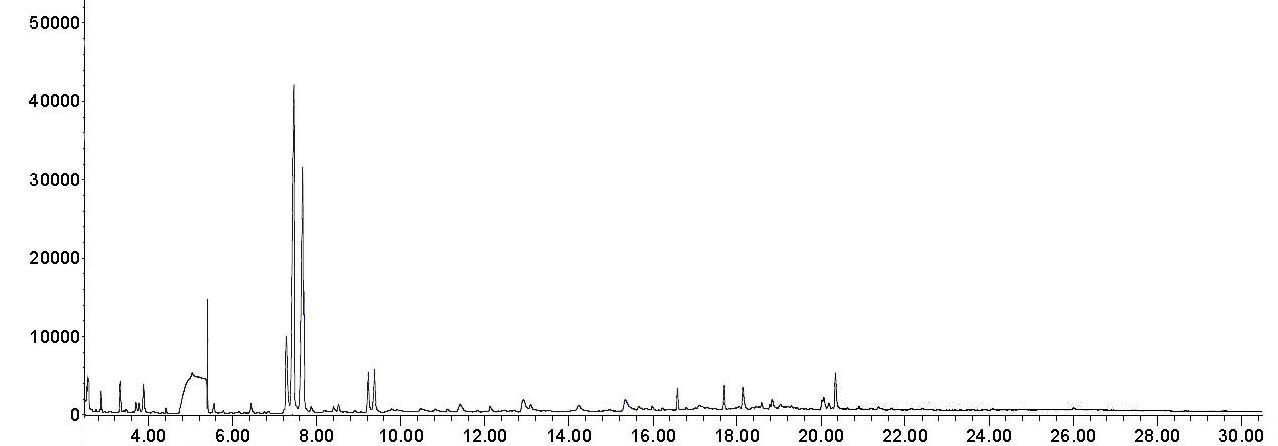


5.378

7.383

5.291

9.219

**A**

20.792


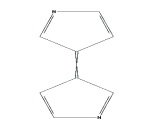


Time (min)

Abundance


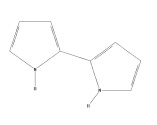


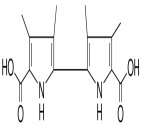


9.367

7.646

7.884


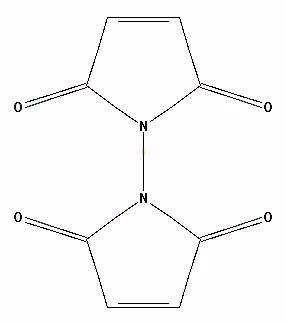


**B**

| **Retention time (min)** | **Organic compounds containing 2 pyrrole rings** | **Peak area (%)** | **Probabilty** |
| --- | --- | --- | --- |
| 5.291 | 3,3'-Bipyrrole | 11.0 | 88 |
| 5.378 |  | 8.7 | 92 |
| 7.383 | 2,2'-Bipyrrole | 7.7 | 90 |
| 7.646 |  | 17.2 | 91 |
| 7.884 |  | 10.3 | 87 |
| 9.219 | 1,1'-Bipyrrole-2,2',5,5'-tetraone | 15.4 | 94 |
| 9.367 |  | 7.5 | 96 |
| 20.792 | 3,3',4,4'-Tetramethyl-1H,1'H-2,2'-bipyrrole-  5,5'-dicarboxylic acid | 7.6 | 98 |

**Figure E.8.** Selected ion (*m*/*z*: 134) monitoring chromatogram of Ni(OEP)-BC (A) and list of detected organic compounds containing 2 pyrrole rings (B)


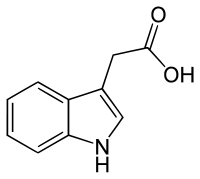


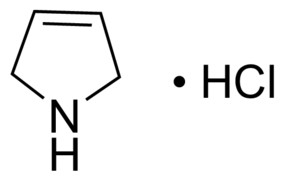


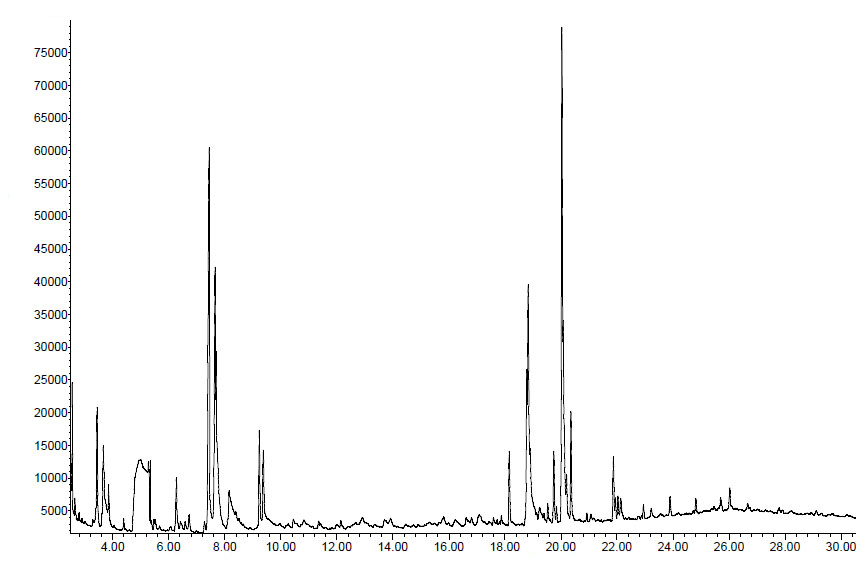


5.569

6.528

18.240

Time (min)

Abundance

19.276

20.083

20.279

**A**

19.913

**B**

| **Retention time (min)** | **Organic compounds containing 1 pyrrole ring** | **Peak area (%)** | **Probability** |
| --- | --- | --- | --- |
| 5.596 | 1H-Pyrrole | 11.1 | 82 |
| 6.528 |  | 4.2 | 91 |
| 18.240 | Indole acetic acid | 5.0 | 74 |
| 19.276 |  | 8.2 | 82 |
| 19.913 |  | 4.6 | 90 |
| 20.083 |  | 19.4 | 85 |
| 20.279 |  | 4.8 | 78 |

**Figure E.9.** Selected ion (*m*/*z*: 67) monitoring chromatogram of Ni(OEP)-BC (A) and list of detected organic compounds containing 1 pyrrole ring (B)


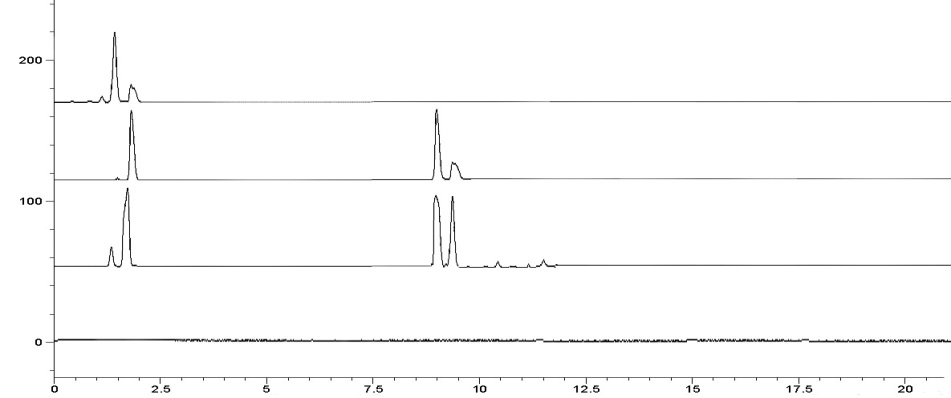


**N**

**H**

**C**

**A**

**Ni**

Time (min)

Abundance

C_2_HN

C_4_H_x_N

C_10_H_x_

C_12_H_x_


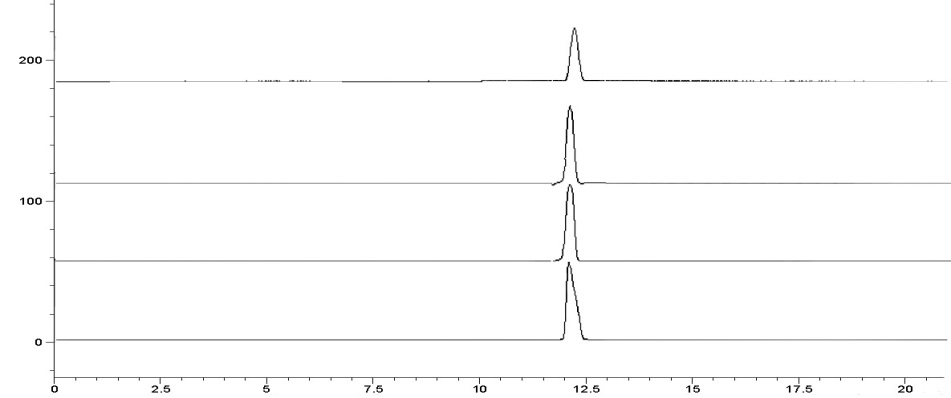


**N**

**H**

**C**

**B**

**Ni**

Time (min)

Abundance

C_28_H_20_N_4_Ni

**Figure E.10.** The atomic emission spectra of aqueous phase (A) and sediment (B) of Ni(OEP)-SC


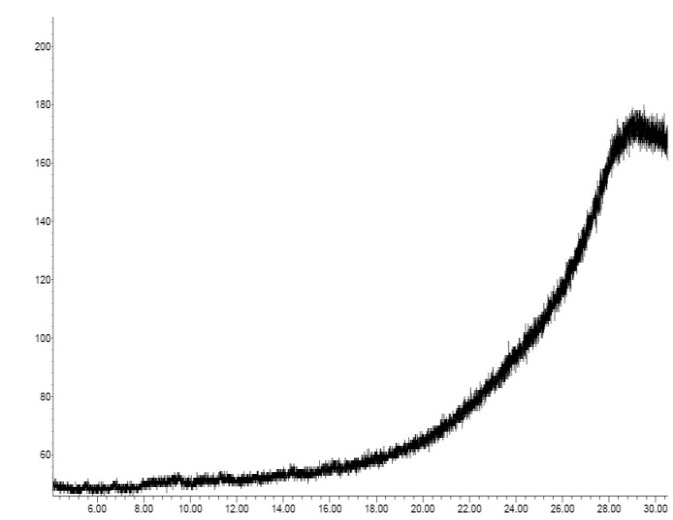


*m/z:*368 - total peak area: 0

Time (min)

Abundance


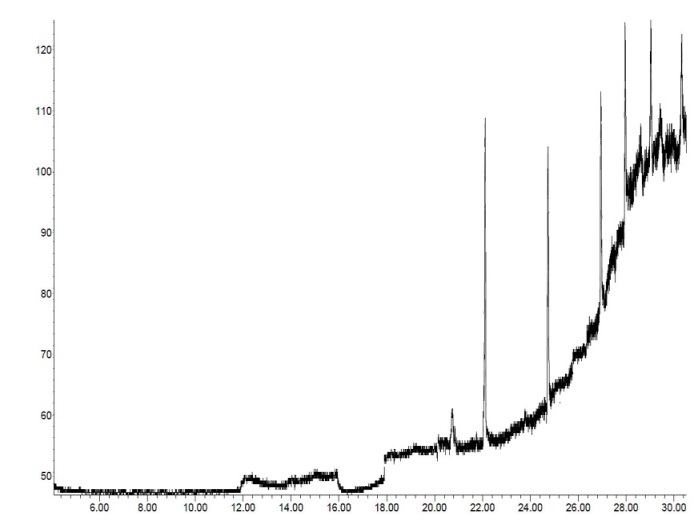


*m/z:*481 - total peak area: 3217

Time (min)

Abundance


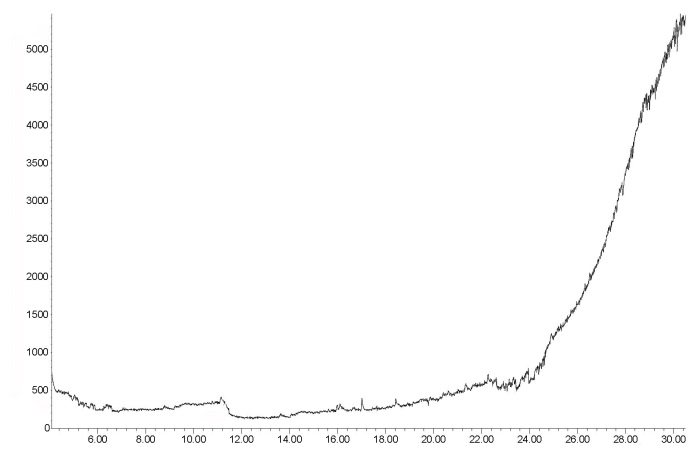


*m/z:*591 - total peak area: 0

Time (min)

Abundance

**Figure E.11.** Selected ion monitoring chromatograms: *m*/*z*: 368 (NiP), *m*/*z*: 481 (Ni(TEP)), and *m*/*z*: 591 (Ni(OEP) of Ni(OEP)-SC


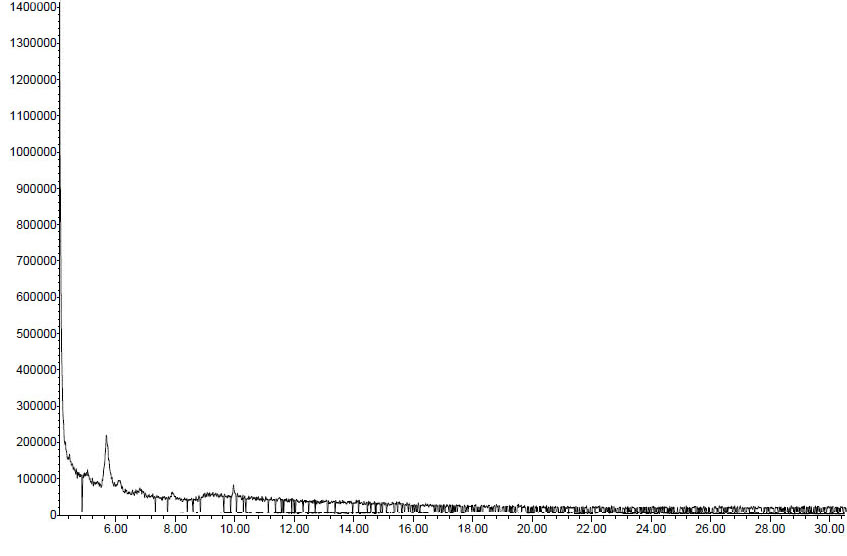


Time (min)

Abundance

**A**

5.886


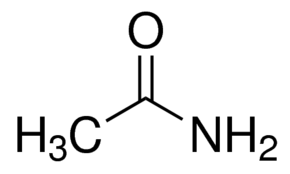


**B**

| **Retention time (min)** | **Organic compounds containing ethyl group** | **Peak area (%)** | **Probability** |
| --- | --- | --- | --- |
| 5.886 | Ethanimidic acid | 91.12 | 80 |

**Figure E.12.** Selected ion (*m*/*z*: 45) monitoring chromatogram of Ni(OEP)-SC (A) and list of detected organic compounds containing ethyl group (B)


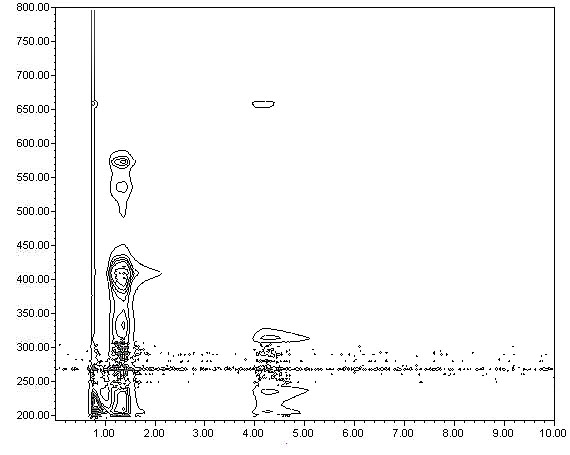


Time (min)

Wavelength (nm)

**A**


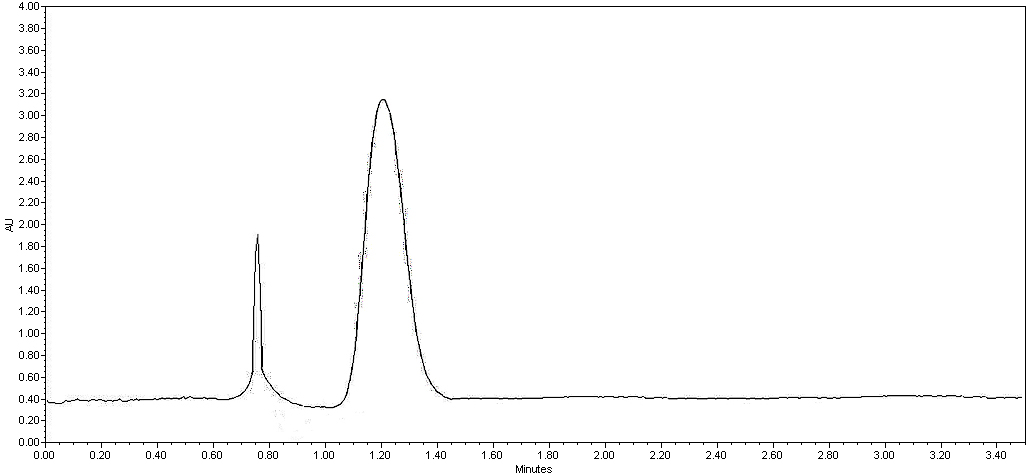


Ni(OEP)-SC

Chloroform

Abundance

Time (min)

**B**


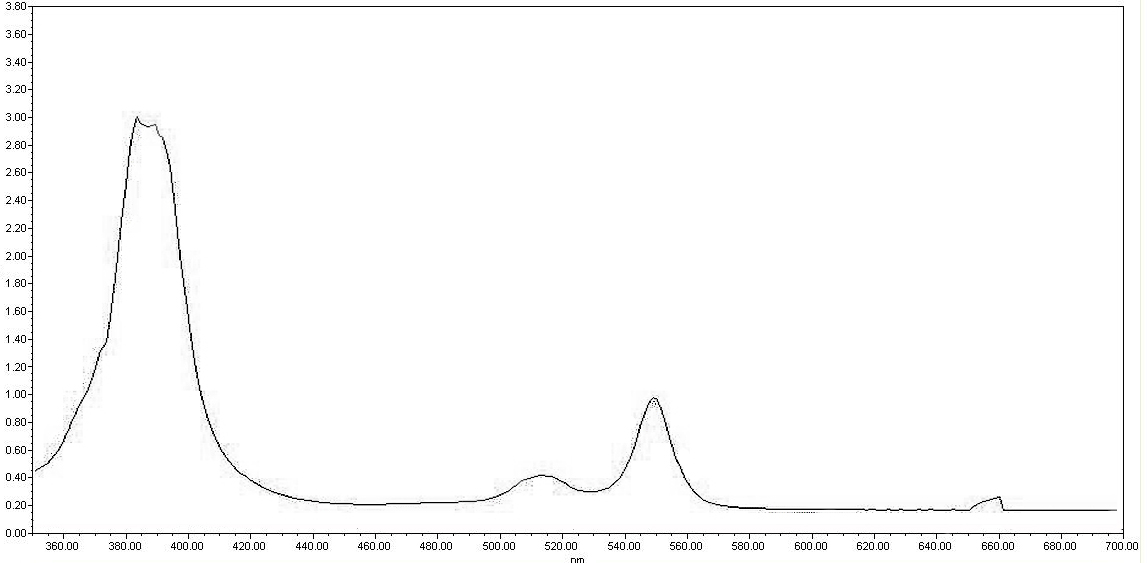


380 nm

516 nm

549 nm

660 nm

Wavelength (nm)

Abundance

**C**

**Figure E.13.** High-performance liquid chromatography with photodiode array detector (HPLC-PDA): 3D chromatogram (A), 425 nm chromatogram (B), and UV-Vis spectrum (C) of Ni(OEP)-SC

*m/z:* 67 - total peak area: 0

*m/z:*134 - total peak area: 0

*m/z:*201 - total peak area: 0


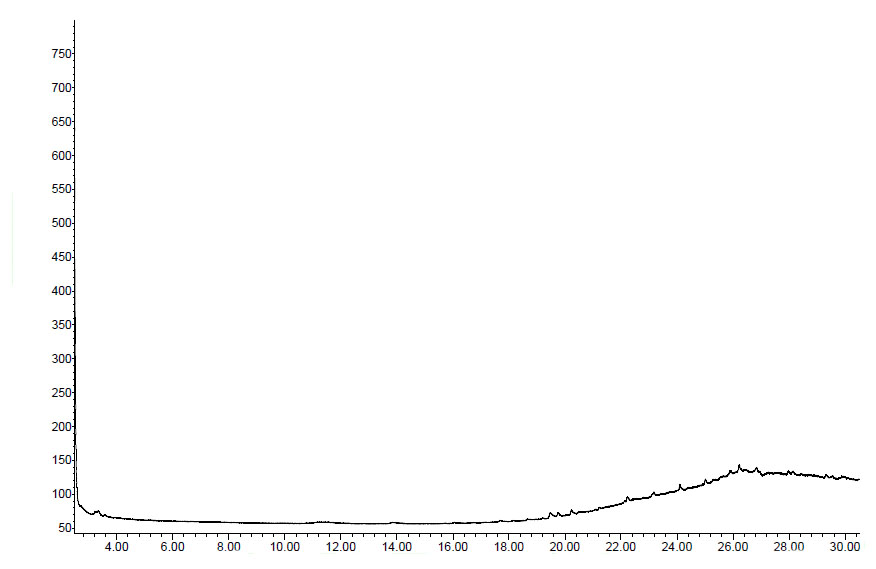


Abundance

Time (min)


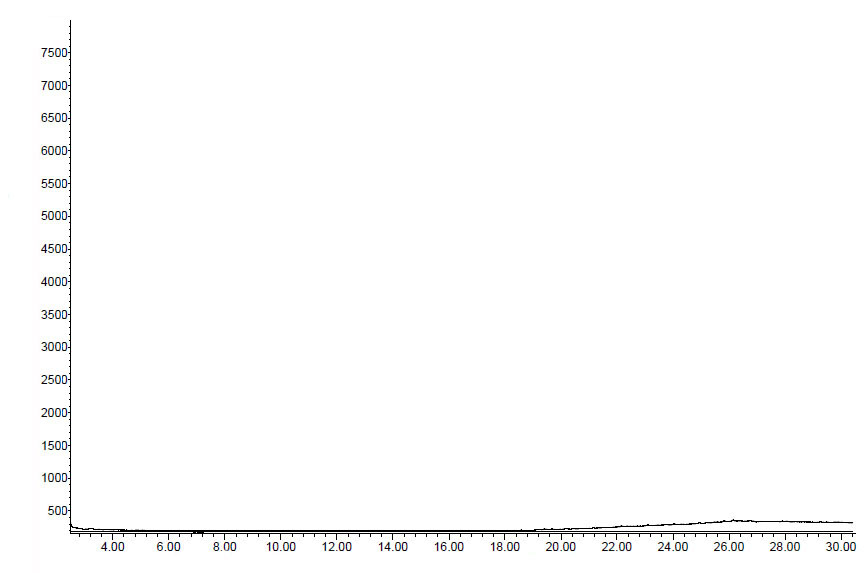


Abundance

Time (min)


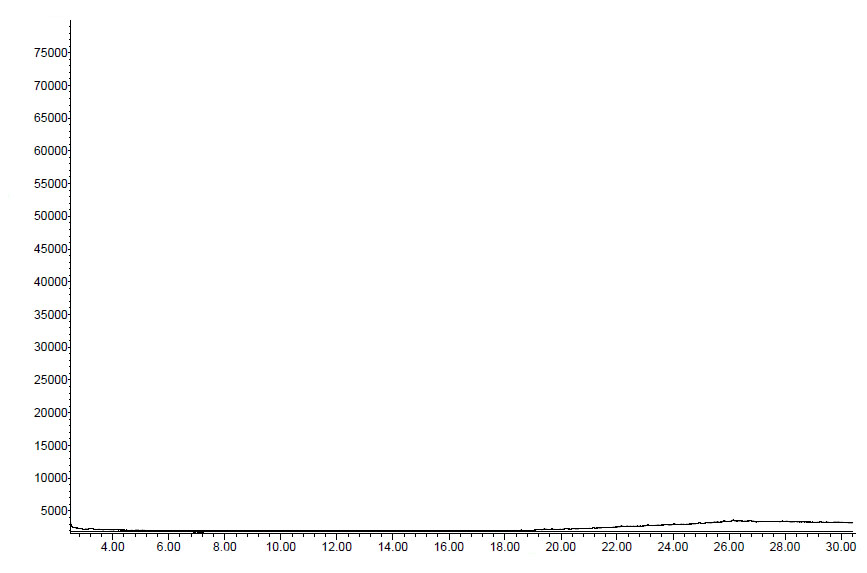


Abundance

Time (min)

**Figure E.14.** Selected ion monitoring chromatograms: *m*/*z*: 201 (organic compounds containing 3 pyrrole rings), *m*/*z*: 134 (organic compounds containing 2 pyrrole rings), and *m*/*z*: 67 (organic compounds containing 1 pyrrole ring) of Ni(OEP)-SC
