# Supplementary material for: Postdiagenetic Bacterial Transformation of Nickel and Vanadyl Sedimentary Porphyrins of Organic-Rich Shale Rock (Fore-Sudetic Monocline, Poland)
Source: Front Microbiol. 2021 Nov 30;12:772007. doi: 10.3389/fmicb.2021.772007 (PMC8669743; doi:10.3389/fmicb.2021.772007)
Supplement: Supplementary file 6 [file Table_6.DOCX]

**Supplementary Material F. Supplementary results for culture of strain LM27 on octaethyl vanadyl porphyrin (VO(OEP**)**-BC) and sterile control (VO(OEP)-SC)**

**A**

**B**

| Parameter | VO(OEP)-BC |
| --- | --- |
| CFU duplication time (days) | 1.43 |
| Maximal CFU/ml | 81x10^6^ |

**Figure F.1.** Growth curve of strain LM27 on medium supplemented with VO(OEP): growth curve (A), duplication time and maximal CFU (B)


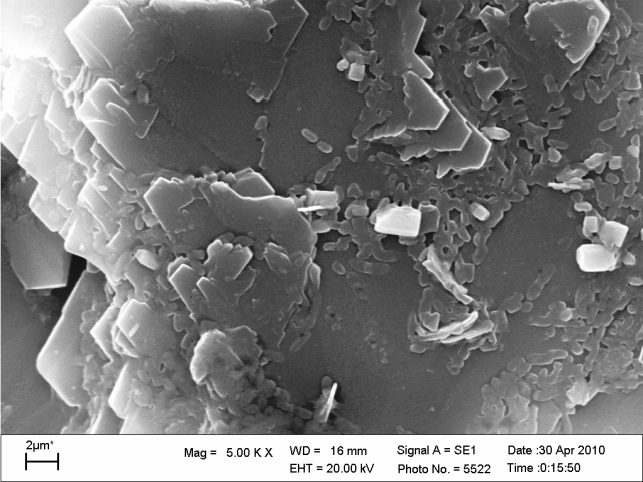

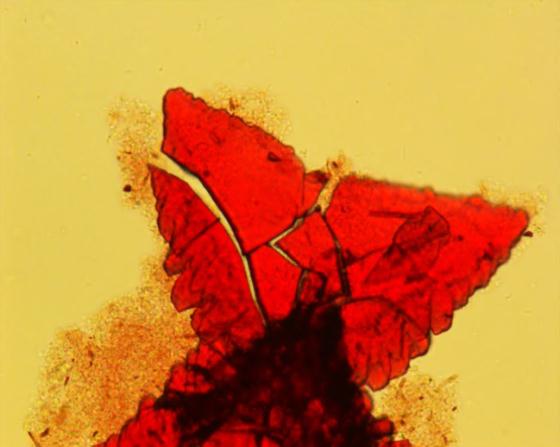

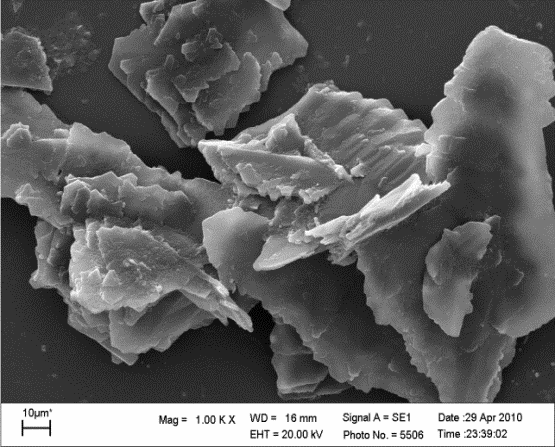

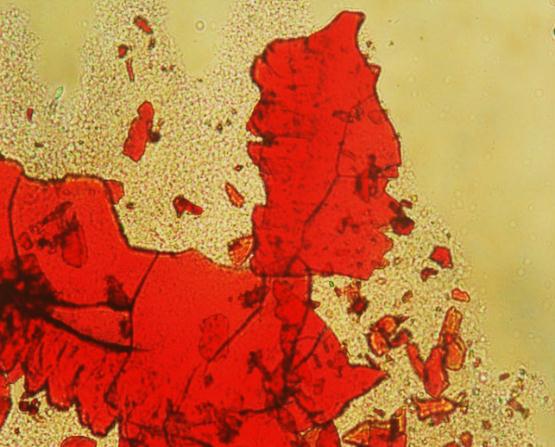

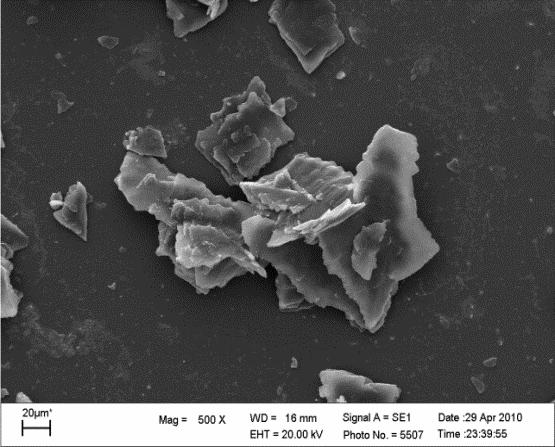


**A**

**B**

**C**

**D**

**E**

**Figure F.2.** Biofilm of strain LM27 on VO(OEP): light (A, B) and scanning electron (C, D, E) microphotographs


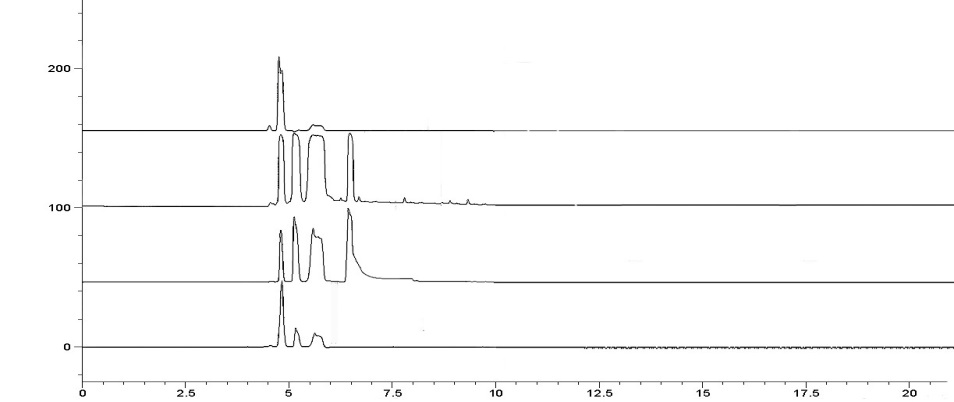


**N**

**H**

**C**

**A**

**V**O(MTPP)

Time (min)

Abundance

C_7_H_x_V

C_5_H_x_N_2_V

C_8_H_x_

C_4_H_x0_V


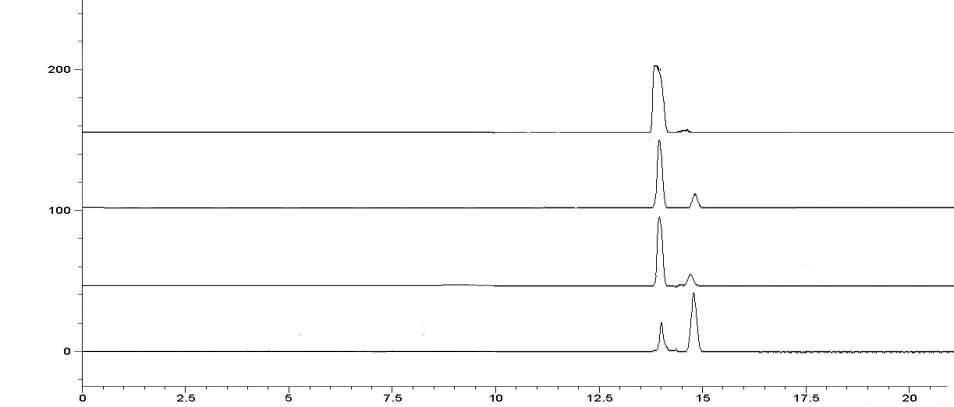


**N**

**H**

**C**

**B**A

**V**O(MTPP)

Time (min)

Abundance

C_12_H_x_V

C_20_H_x_N_4_V

**Figure F.3.** The atomic emission spectra of aqueous phase (A) and sediment (B) of VO(OEP)-BC


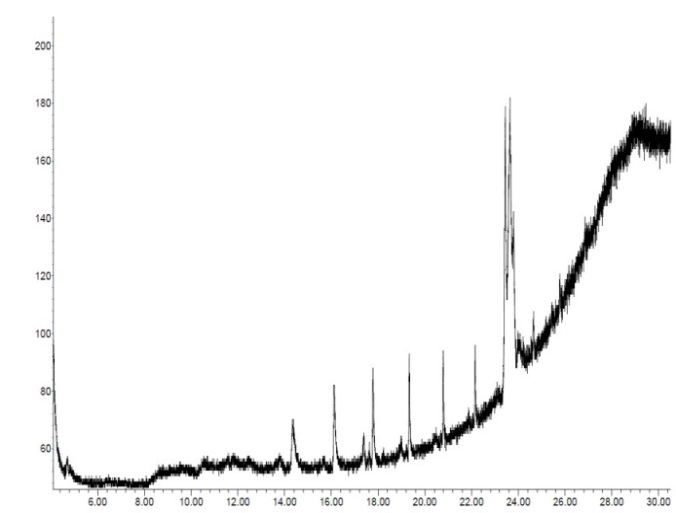


*m/z:* 361 - total peak area: 55872

Time (min)

Abundance


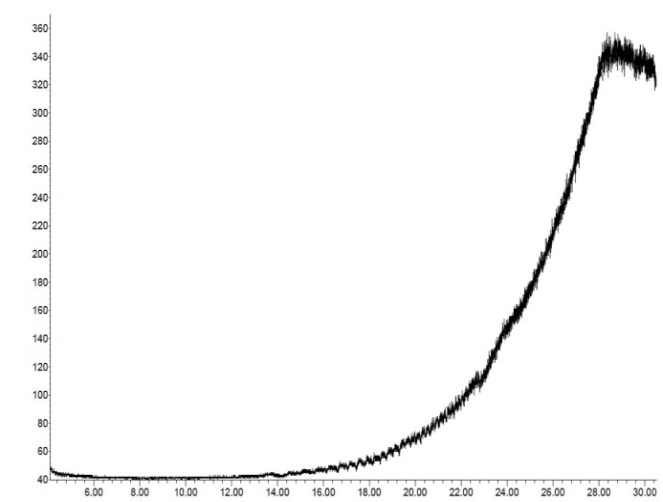


*m/z:* 472 - total peak area: 0

Time (min)

Abundance


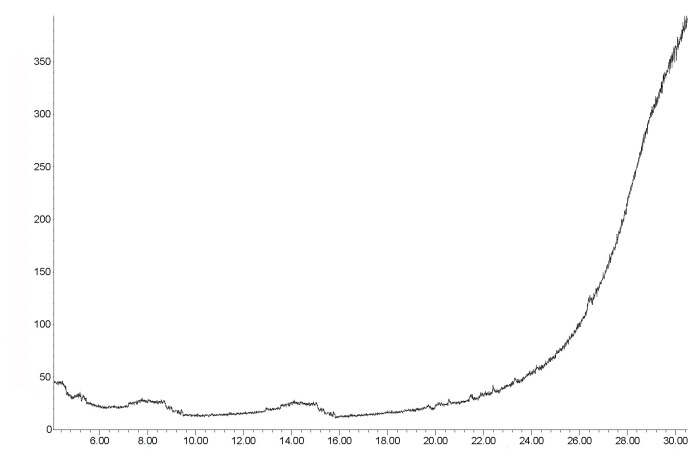


*m/z:* 599 - total peak area: 0

Time (min)

Abundance

**Figure F.4.** Selected ion monitoring: *m*/*z*: 361 (VOP), *m*/*z*: 472 (VO(TEP)), and *m*/*z*: 599 (VO(OEP), chromatograms of VO(OEP)-BC


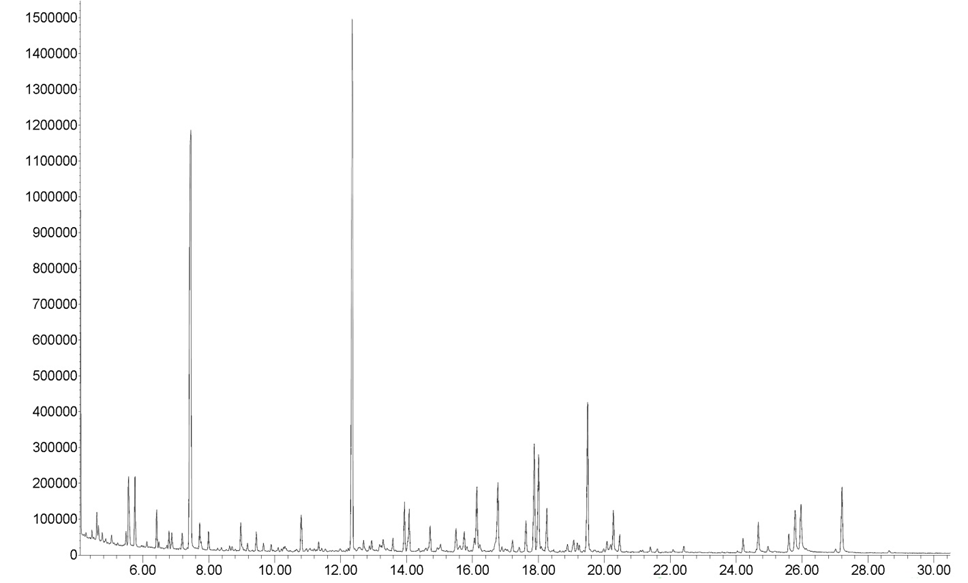


Time (min)

Abundance

**B**

4.452

14.715


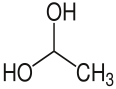


5.055

6.491

6.117

5.489

89

10.654

89

16.881


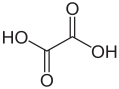


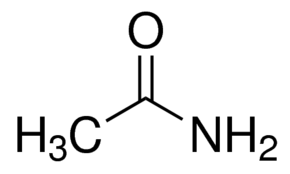


**C_2_H_2_O_4_**

89

| **Retention time (min)** | **Organic compounds containing ethyl group** | **Peak area (%)** | **Probability** |
| --- | --- | --- | --- |
| 4.452 | Ethanimidic acid | 0.21 | 72 |
| 5.055 |  | 0.25 | 64 |
| 6.117 |  | 0.14 | 82 |
| 6.491 |  | 0.17 | 88 |
| 5.489 | Ethanedioic acid | 0.37 | 73 |
| 10.654 |  | 0.14 | 69 |
| 14.715 |  | 0.90 | 81 |
| 16.881 | Ethane-1,2-diol | 2.09 | 87 |

**Figure F.5.** Selected ion (*m*/*z*: 45) monitoring chromatogram of VO(OEP)-BC (A) and list of detected organic compounds containing ethyl group (B)


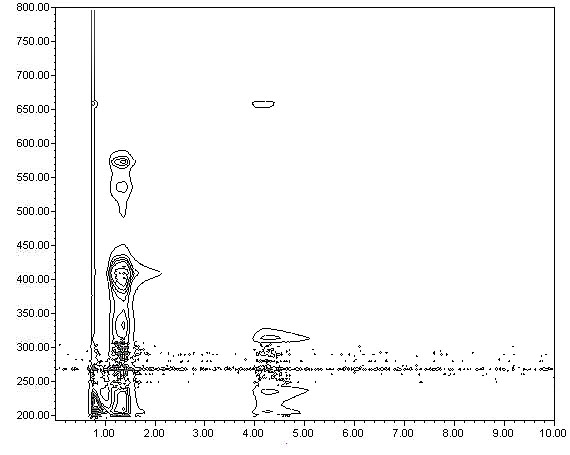


Time (min)

Wavelength (nm)

**A**


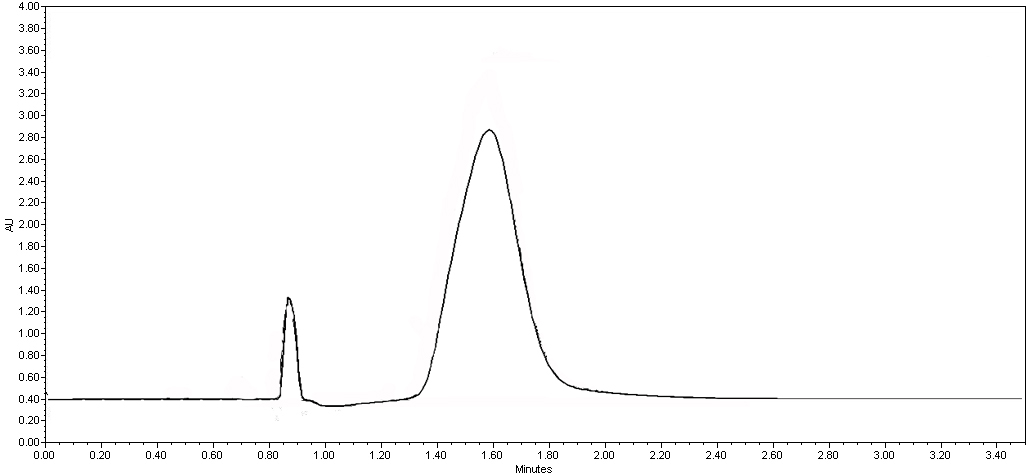


VO(OEP)-BC

Chloroform

Abundance

Time (min)

**B**

**Figure F.6.** High-performance liquid chromatography with photodiode array detector (HPLC-PDA): 3D chromatogram (A), 425 nm chromatogram (B) and UV-Vis spectrum, (C) of VO(OEP)-BC


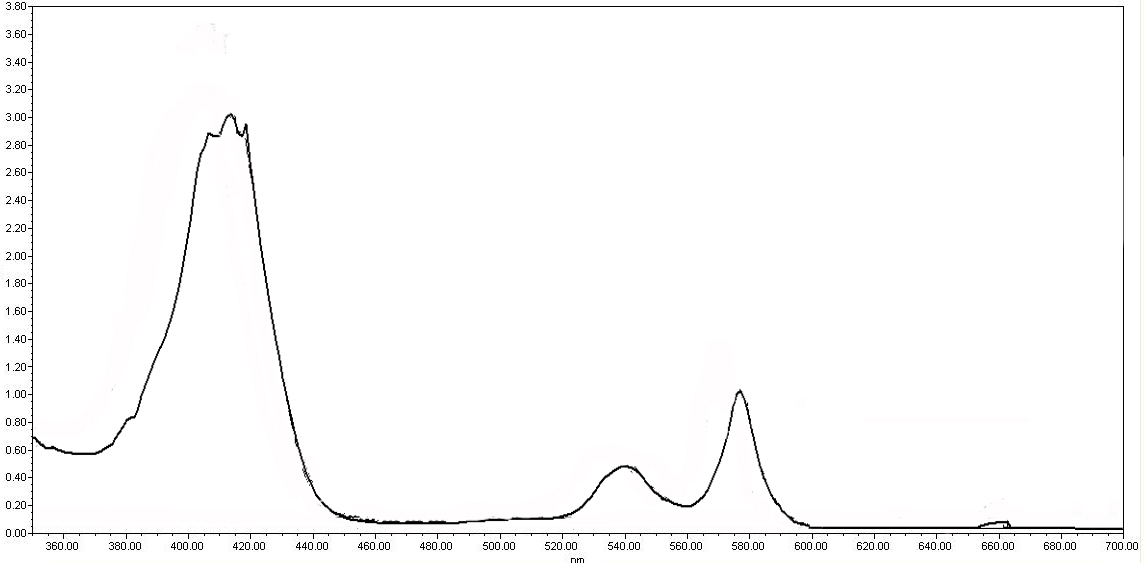


407 nm

532 nm

660 nm

Wavelength (nm)

Abundance

**C**

575 nm


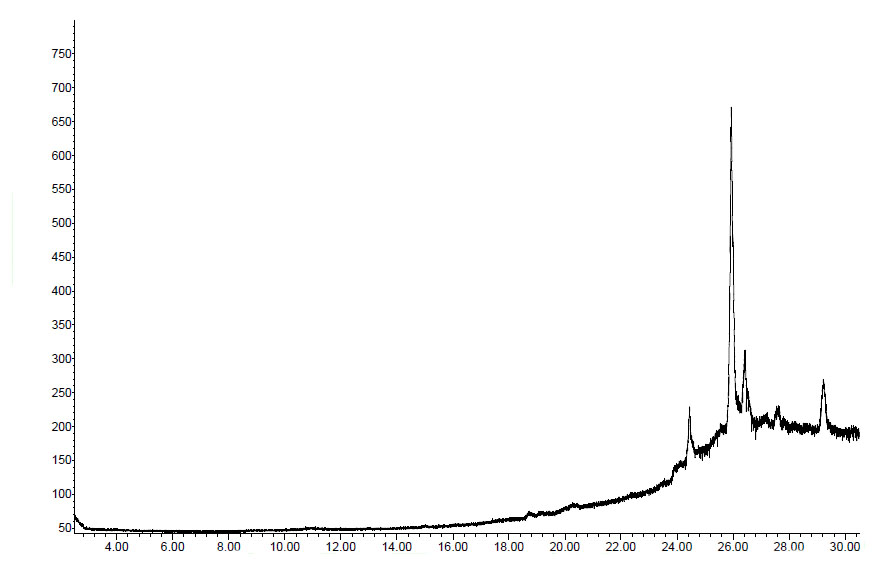


25.891


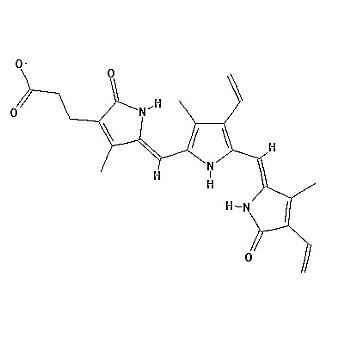


Time (min)

Abundance

**A**

| **Retention time (min)**  **B** | **Organic compounds containing 3pyrrole rings** | **Peak area (%)** | **Probability** |
| --- | --- | --- | --- |
| 25.891 | 3-[(5Z)-5-[[4-Ethenyl-5-[(Z)-(4-ethenyl-3-methyl-5-oxopyrrol-2-ylidene)methyl]-3-methyl-1H-pyrrol-2-yl]methylidene]-4-methyl-2-oxopyrrol-3-yl]propanoate | 89.1 | 95 |

**Figure F.7.** Selected ion (*m*/*z*: 201) monitoring chromatogram of VO(OEP)-BC (A) and list of detected organic compounds containing 3 pyrrole rings (B)


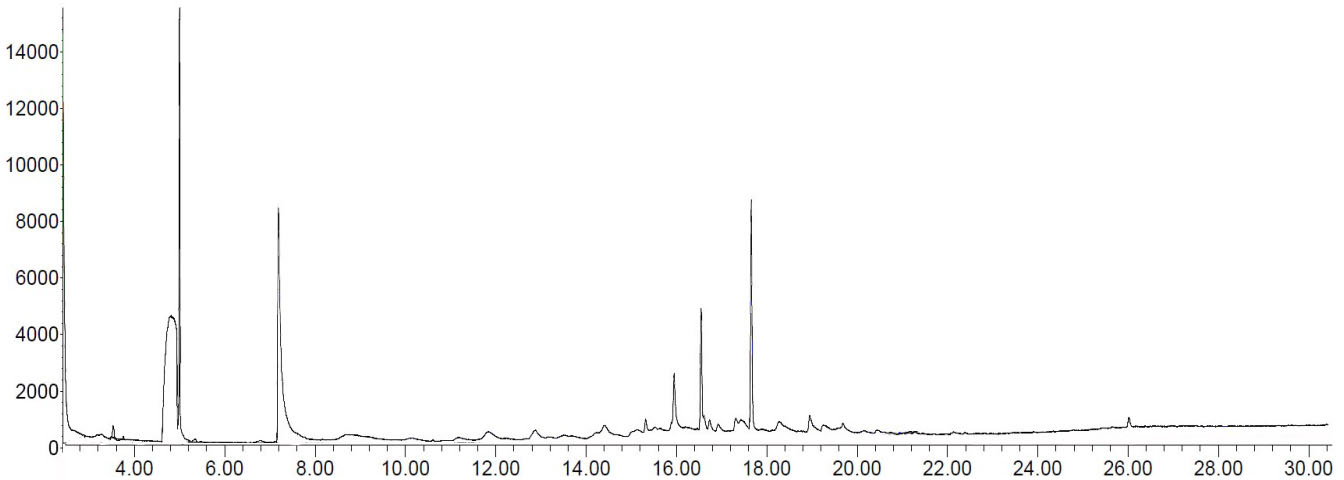


5.191

7.400

5.684

16.288

18.064

**A**

C_21_H_23_N_3_O

26.829


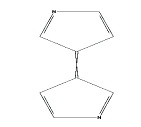


Time (min)

Abundance


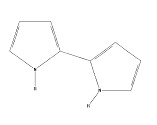


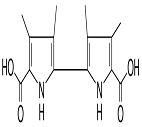


16.373

| **Retention time (min)**  **B** | **Organic compounds containing 2 pyrrole rings** | **Peak area (%)** | **Probability** |
| --- | --- | --- | --- |
| 5.191 | 3,3'-Bipyrrole | 23.4 | 92 |
| 5.684 |  | 20.6 | 89 |
| 7.400 | 2,2'-Bipyrrole | 18.6 | 91 |
| 16.288 | 3,3',4,4'-Tetramethyl-1H,1'H-2,2'-bipyrrole-5,5'-dicarboxylic acid | 8.2 | 96 |
| 16.373 |  | 12.6 | 94 |
| 18.064 |  | 14.1 | 97 |
| 26.829 | 2H-Pyrrol-2-one, 5-[[2-[(4 aminophenyl)methylene]-3,4-dimethyl-2H pyrrol-5-yl]methylene]-3-ethyl-1,5-dihydro-4-methyl-, (Z,Z) | 1.52 | 90 |

**Figure F.8.** Selected ion (*m*/*z*: 134) monitoring chromatogram of VO(OEP)-BC (A) and list of detected organic compounds containing 2 pyrrole rings (B)


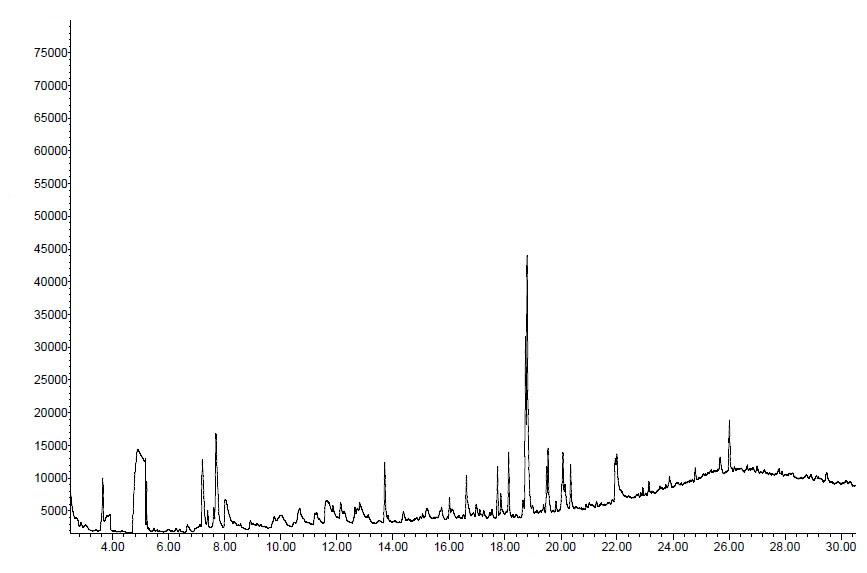


4.124

7.230

7.765

7.901


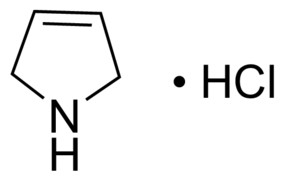


18.871

19.505

21.400

21.494

22.299

26.221


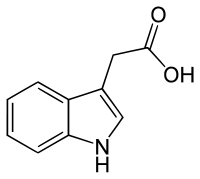


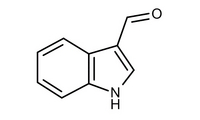


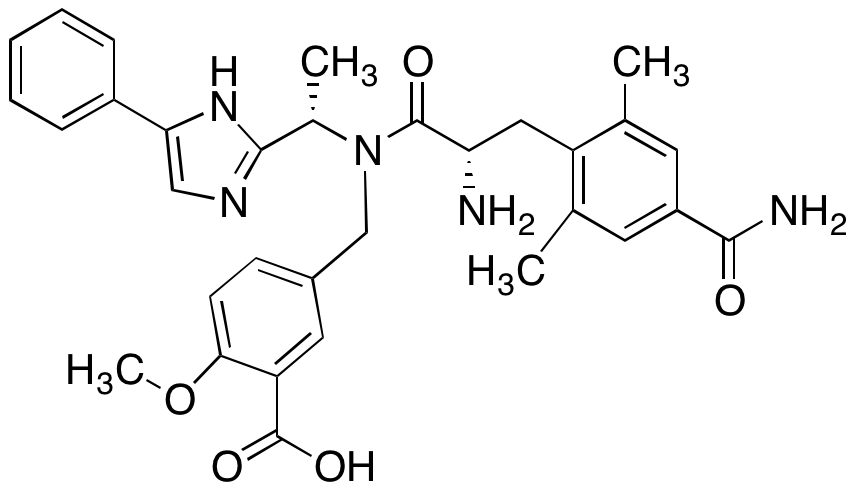


Time (min)

Abundance

8.488

16.881

9.150

8.148

C_18_H_26_N


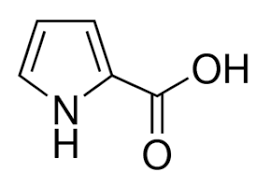


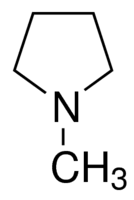


C_19_H_26_N_2_O_2_Br

**A**

**B**

| **Retention time (min)** | **Organic compounds containing 1 pyrrole ring** | **Peak area (%)** | **Probability** |
| --- | --- | --- | --- |
| 4.124 | 1H-Pyrrole | 10.3 | 91 |
| 7.230 |  | 5.4 | 88 |
| 7.765 |  | 3.8 | 72 |
| 7.901 |  | 2.2 | 84 |
| 8.148 | 2-Pentanone, 5-(5-methyl-4-cyclononen-1-ylidene) | 0.59 | 73 |
| 8.488 | 1H-Pyrrole-2-carboxylic acid | 0.74 | 92 |
| 9.150 | 1-Methylpyrroline | 0.14 | 71 |
| 16.881 | (+-)-(3AR*,4R*,11BR*)-Methyl 3-((Z)-2-bromobut-2-en-1-yl)-2,3,3A,4,5,7-hexahydro-4-hydroxy-1H-pyrrolo[2,3-D]carbazole-6-carboxylate | 0.87 | 80 |
| 18.871 | Indole acetic acid | 4.8 | 82 |
| 19.505 |  | 4.2 | 79 |
| 21.400 |  | 3.8 | 91 |
| 21.494 |  | 2.1 | 84 |
| 22.299 | Indole carbaldehyde | 5.8 | 87 |
| 26.221 | 2H-Pyrrol-2-one, 5-[[2-[(4-aminophenyl)methylene]-3,4-dimethyll]methylene]-3-ethyl-1,5-dihydro-4-methyl | 4.7 | 73 |

**Figure F.9.** Selected ion (*m*/*z*: 67) monitoring chromatogram of VO(OEP)-BC (A) and list of detected organic compounds containing 1 pyrrole ring (B)


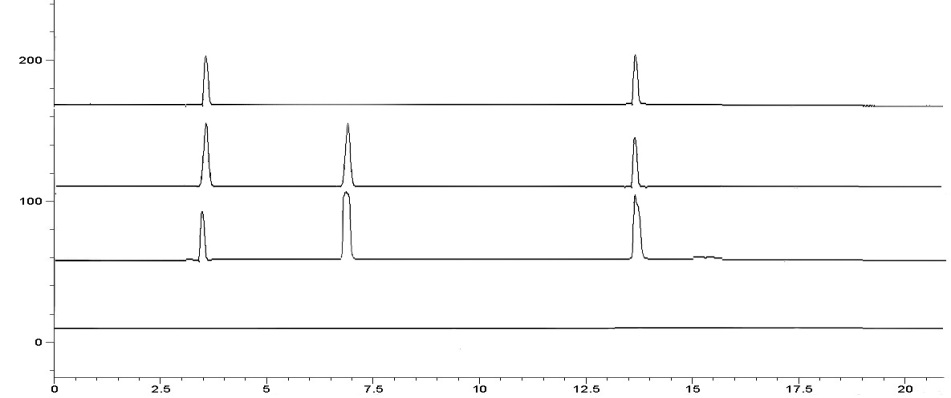


**N**

**H**

**C**

**A**

**V**

Time (min)

Abundance

C_12_H_x_N

C_4_H_x_N

C_8_H_x_


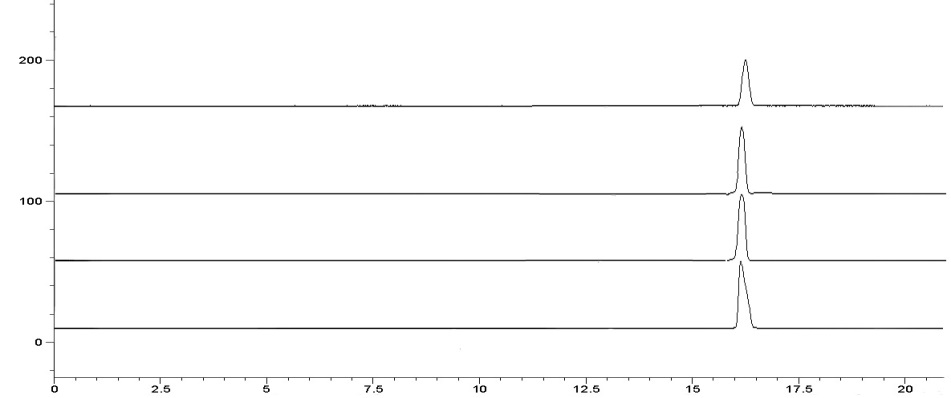


**N**

**H**

**C**

**B**

**V**

Time (min)

Abundance

C_28_H_x_N_4_V

**Figure F.10.** The atomic emission spectra of aqueous phase (A) and sediment (B) of VO(OEP)-SC


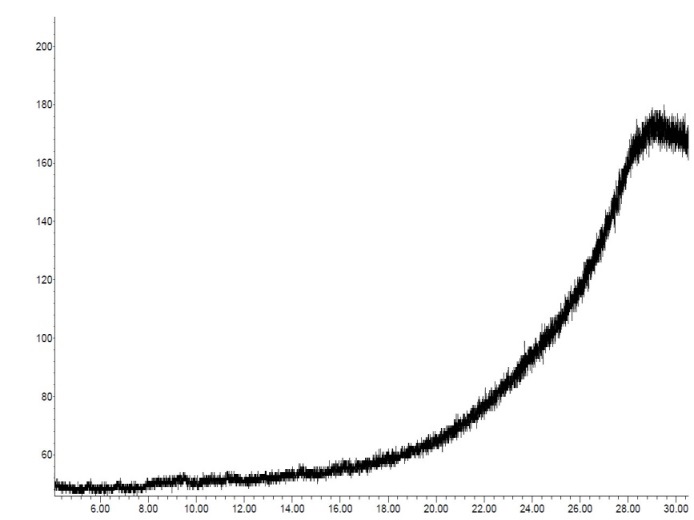


*m/z:*361 - total peak area: 0

Time (min)

Abundance


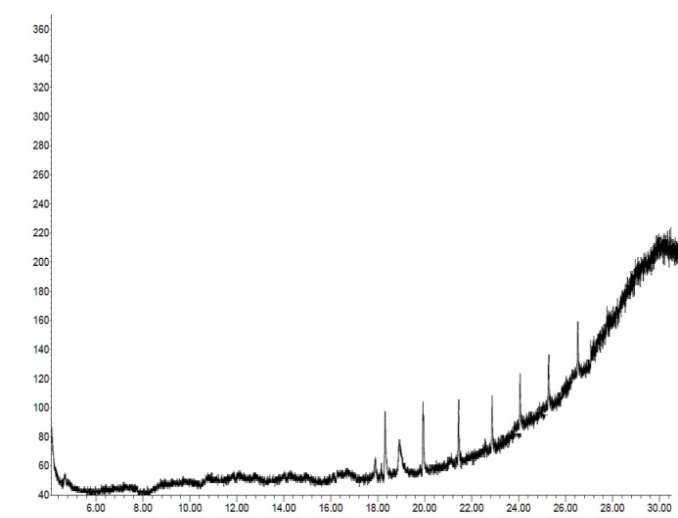


*m/z:*472 - total peak area: 28457

Time (min)

Abundance


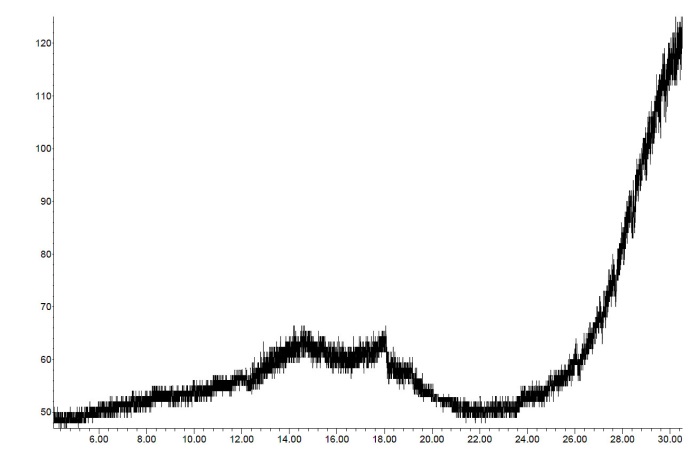


*m/z:* 599 - total peak area: 0

Time (min)

Abundance

**Figure F.11.** Selected ion monitoring: *m*/*z:* 361 (VOP), *m*/*z:* 472 (VO(TEP)), and *m*/*z:* 599 (VO(OEP), chromatograms of VO(OEP)-SC


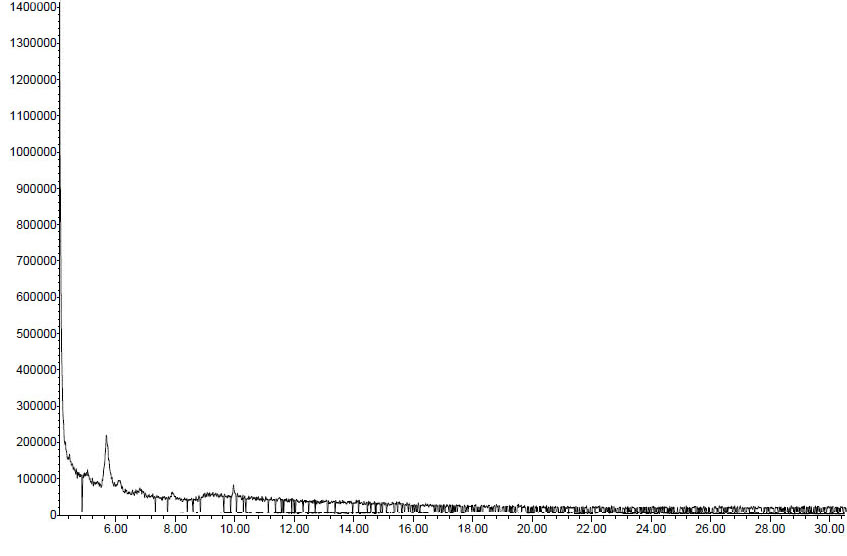


Time (min)

Abundance

**A**

5.893


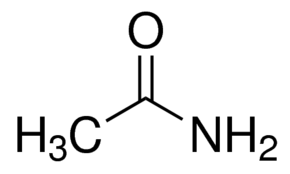


**B**

| **Retention time (min)** | **Organic compounds containing ethyl group** | **Peak area (%)** | **Probability** |
| --- | --- | --- | --- |
| 5.893 | Ethanimidic acid | 81.14 | 80 |

**Figure F.12.** Selected ion (*m*/*z* 45) monitoring chromatogram of VO(OEP)-SC (A) and list of detected organic compounds containing ethyl group (B)

**Figure F.13.** High-performance liquid chromatography with photodiode array detector (HPLC-PDA): 3D chromatogram (A), 425 nm chromatogram (B) and UV-Vis spectrum, (C) of VO(OEP)-SC


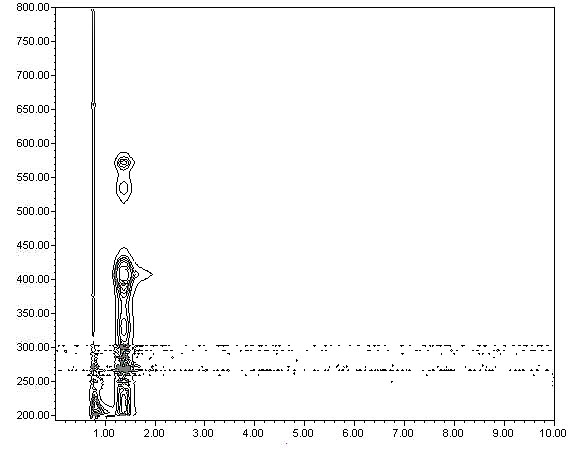


Time (min)

Wavelength (nm)

**A**


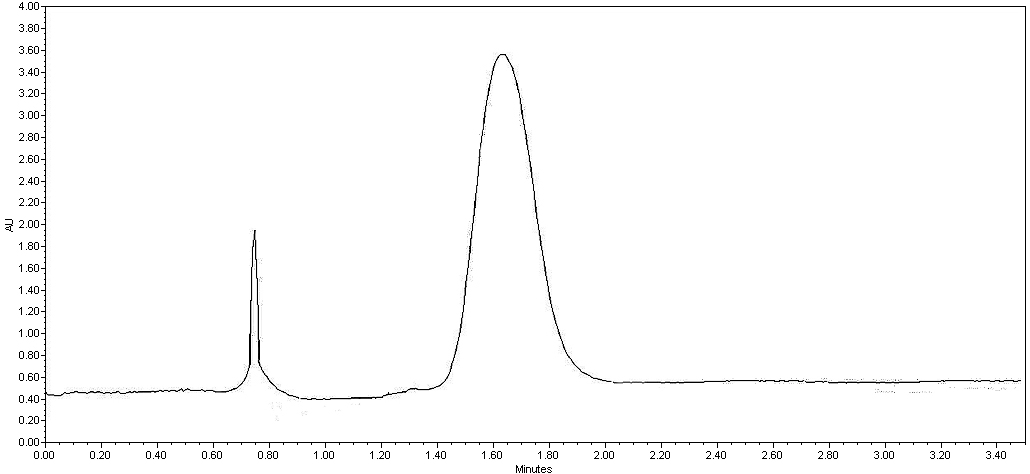


VO(OEP)-SC

Chloroform

Abundance

Time (min)

**B**


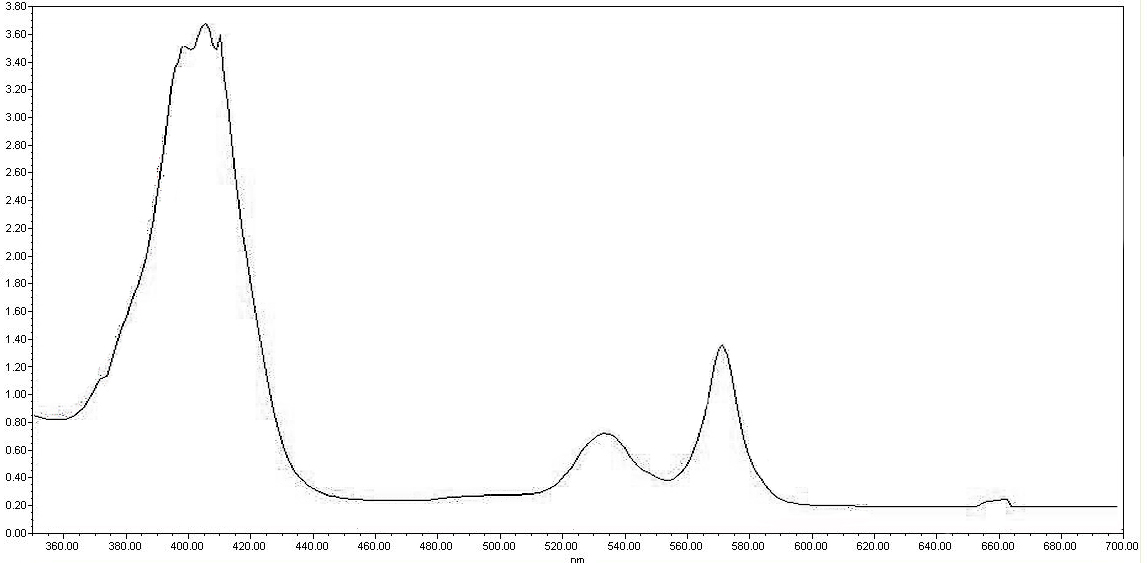


401 nm

529 nm

571 nm

660 nm

Wavelength (nm)

Abundance

**C**


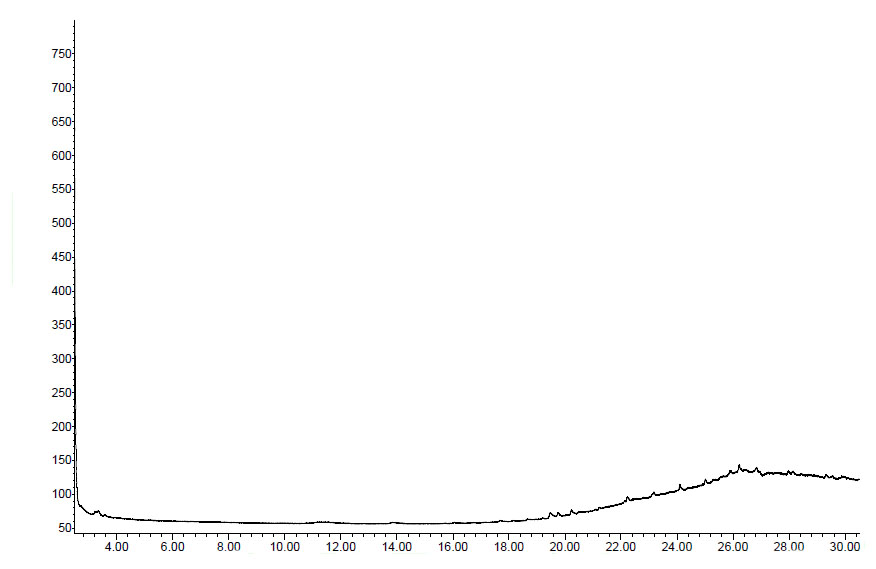


Abundance

Time (min)


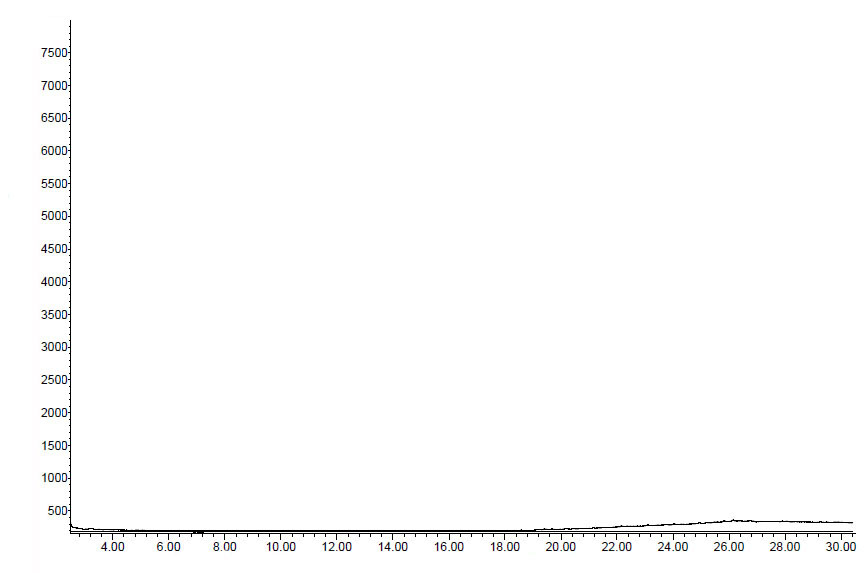


Abundance

Time (min)


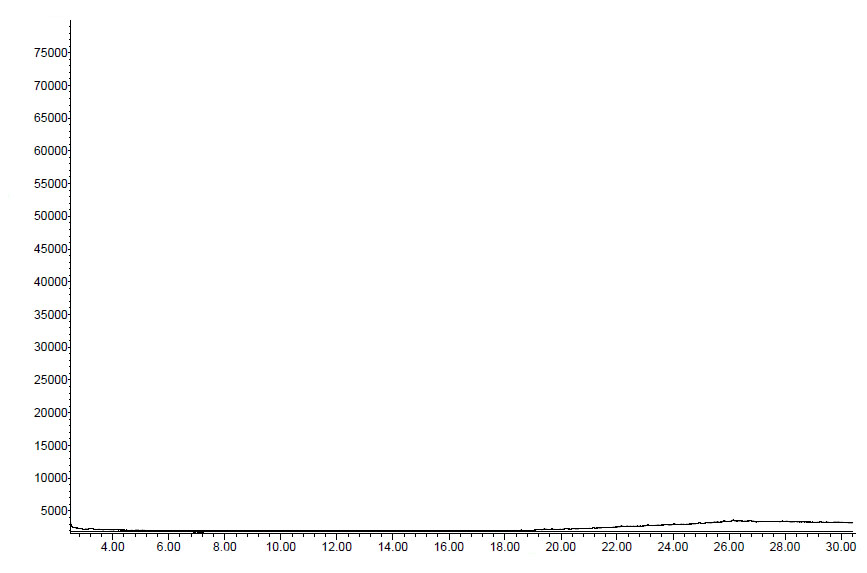


Abundance

Time (min)

**Figure F.14.** Selected ion monitoring chromatograms: *m*/*z*: 201 (organic compounds containing 3 pyrrole rings), *m*/*z*: 134 (organic compounds containing 2 pyrrole rings), and *m*/*z*: 67 (organic compounds containing 1 pyrrole ring) of VO(OEP)-SC

*m/z:* 201 - total peak area: 0

*m/z:* 134 - total peak area: 0

*m/z:* 67 - total peak area: 0
