# Supplementary material for: Postdiagenetic Bacterial Transformation of Nickel and Vanadyl Sedimentary Porphyrins of Organic-Rich Shale Rock (Fore-Sudetic Monocline, Poland)
Source: Front Microbiol. 2021 Nov 30;12:772007. doi: 10.3389/fmicb.2021.772007 (PMC8669743; doi:10.3389/fmicb.2021.772007)
Supplement: Supplementary file 7 [file Table_7.DOCX]

**Supplementary Material H. Supplementary results for the culture of strain LM27 on mineral salt medium with glucose (MBS-BC) and sterile control (MBS-SC)**

**B**

**A**

| Parameter | MBS-BC |
| --- | --- |
| CFU duplication time (days) | 4.28 |
| Maximal CFU/ml | 10.9x10^6^ |

**Figure H.1.** Growth of strain LM27 on MBS medium: growth curve (A), duplication time and maximal CFU (B)


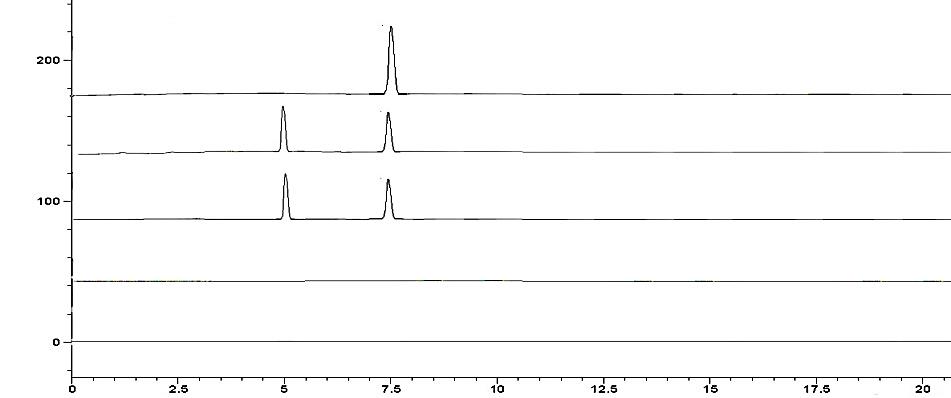


**N**

**H**

**C**

**A**

**V**

Time (min)

Abundance

**Ni**

C_16_H_x_N

C_6_H_x_


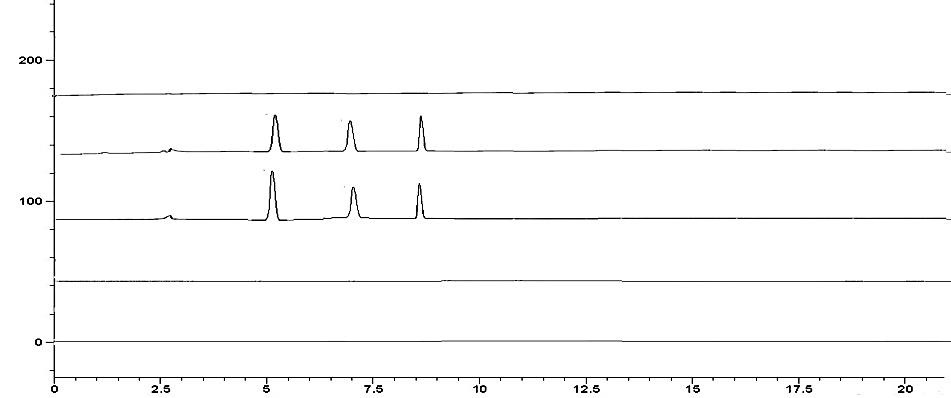


**N**

**H**

**C**

**B**

**V**

Time (min)

)

Abundance

**Ni**

C_18_H_x_

C_10_H_x_

C_22_H_x_

**Figure H.2.** The atomic emission spectra of aqueous phase (A) and sediment (B) of MBS-BC

**Figure H.3.** High-performance liquid chromatography with photodiode array detector (HPLC-PDA): 3D chromatogram (A), 425 nm chromatogram (B), and UV-Vis spectrum (C) of MBS-BC

**B**

**A**


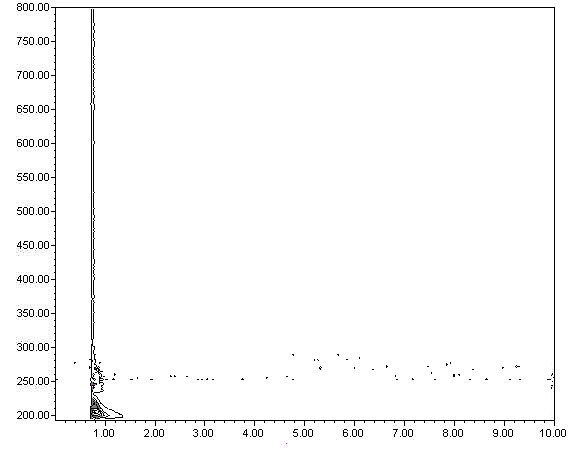


Time (min)

Wavelength (nm)

**C**


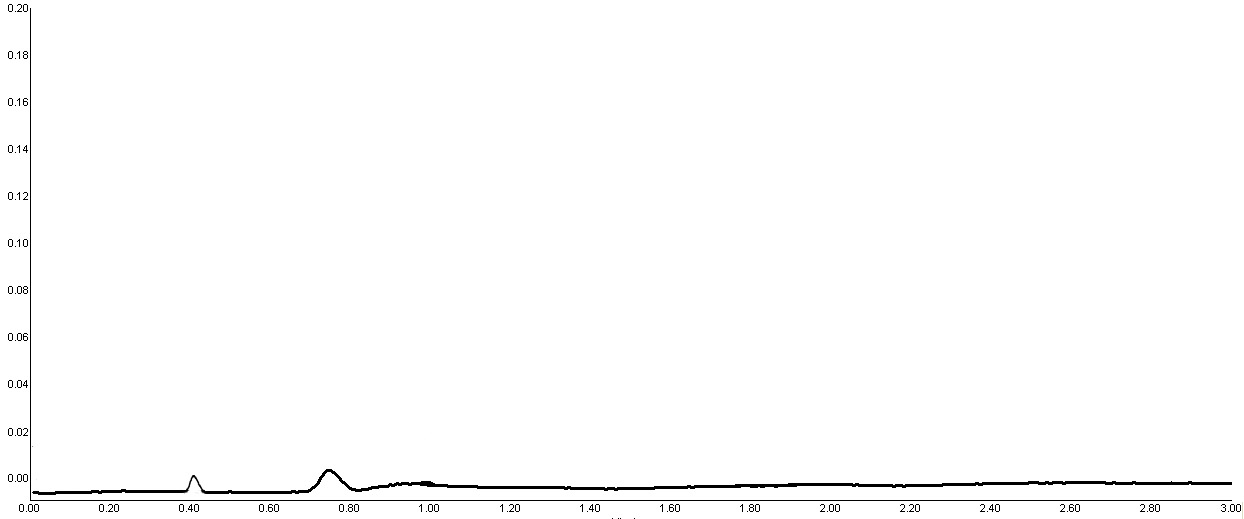


Chloroform

Abundance

Time (min)


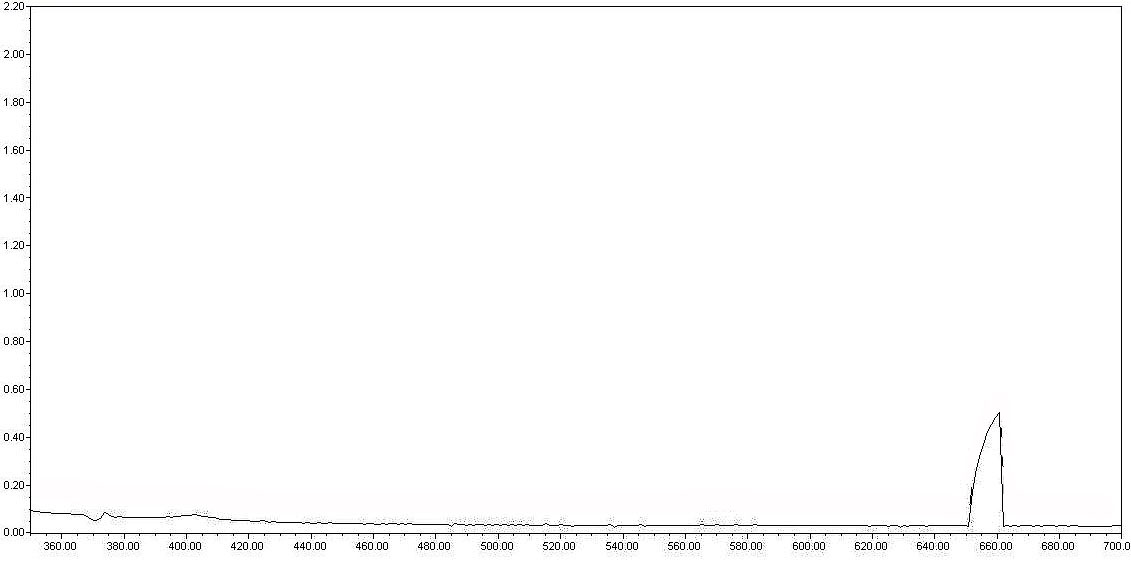


660 nm

Wavelength (nm)

Abundance

*m/z:* 201 - total peak area: 0

*m/z:* 134 - total peak area: 0

*m/z:* 67 - total peak area: 0


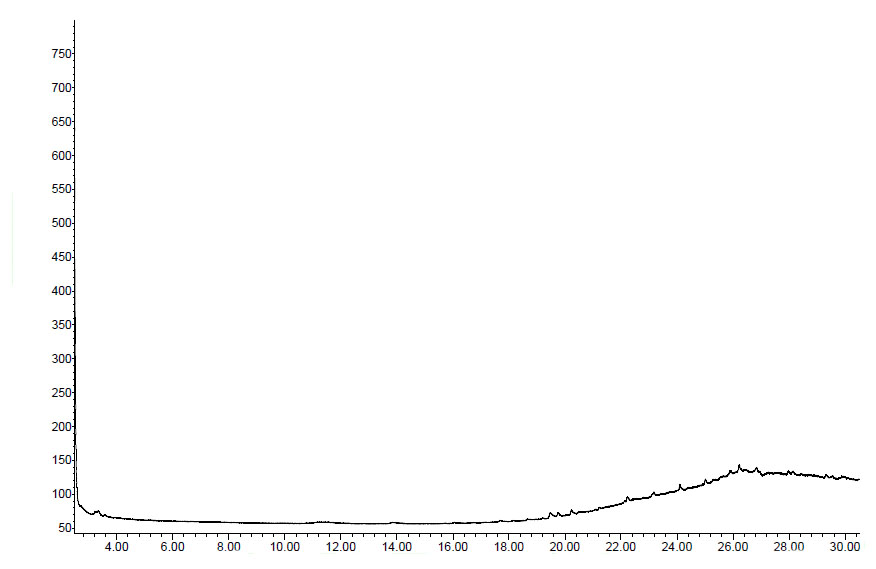


Abundance

Time (min)


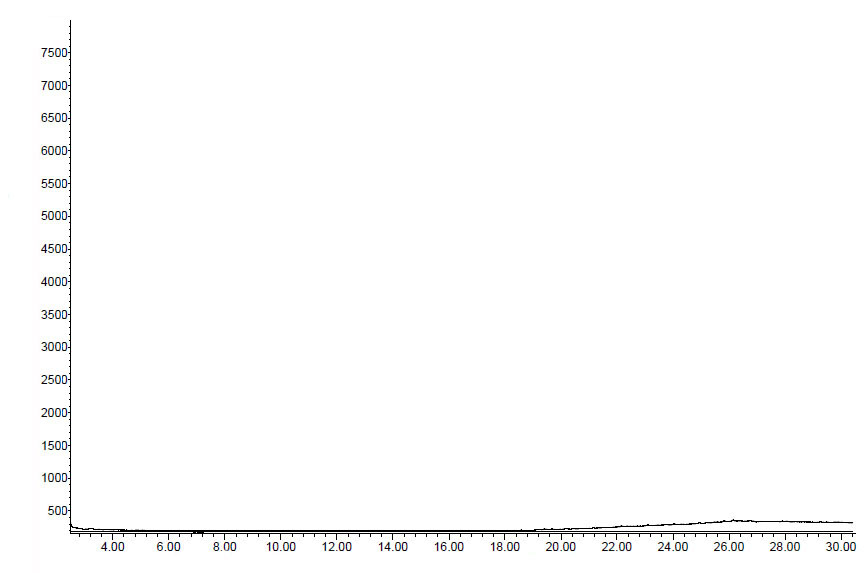


Abundance

Time (min)


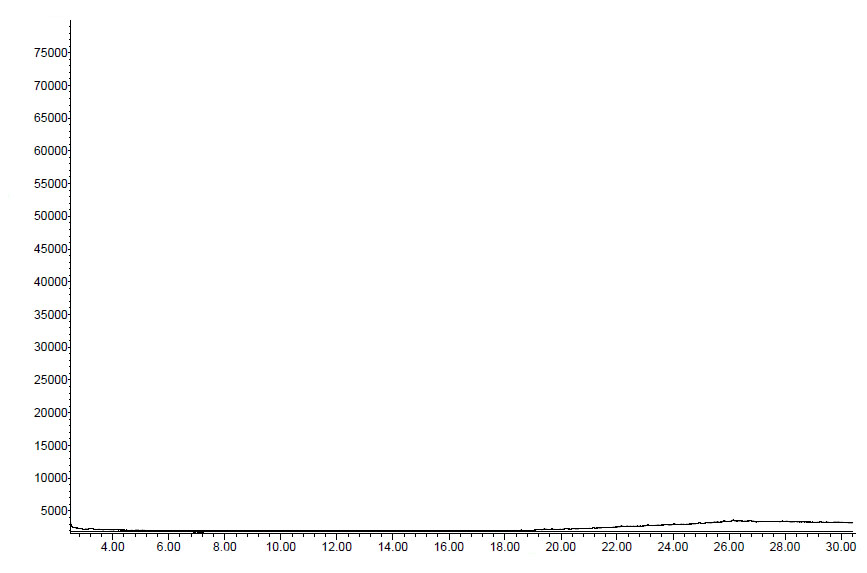


Abundance

Time (min)

**Figure H.4.** Selected ion monitoring chromatograms: *m*/*z* 201 (organic compounds containing three pyrrole group), *m*/*z*: 134 (organic compounds containing two pyrrole group), and *m*/*z*: 67 (organic compounds containing one pyrrole group) of MBS-BC

*m/z:* 118 - total peak area: 0

*m/z:* 88 - total peak area: 0

*m/z:* 77 - total peak area: 0

*m/z:* 45 - total peak area: 0


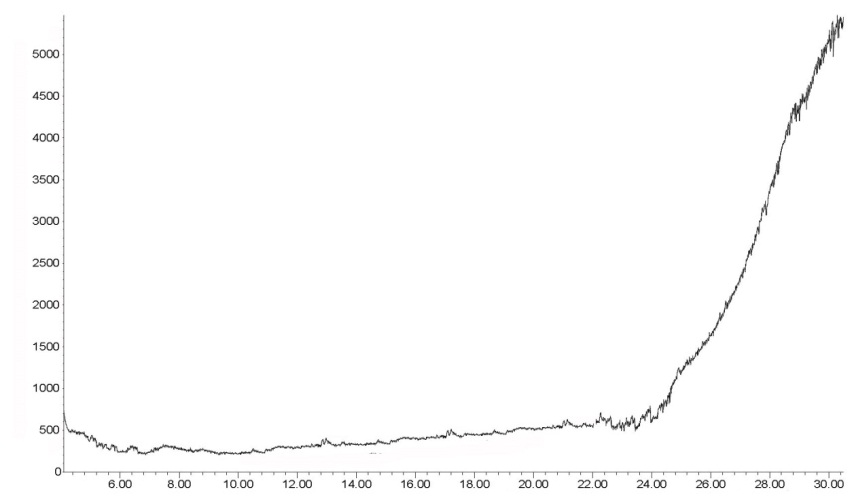


Abundance


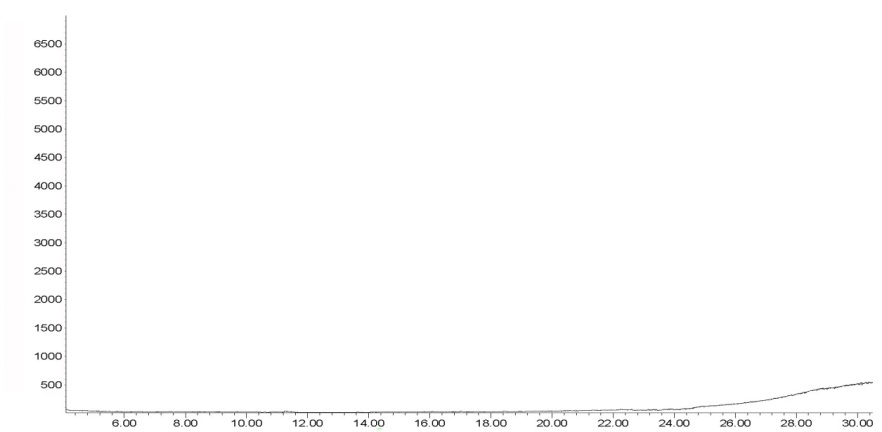


Abundance


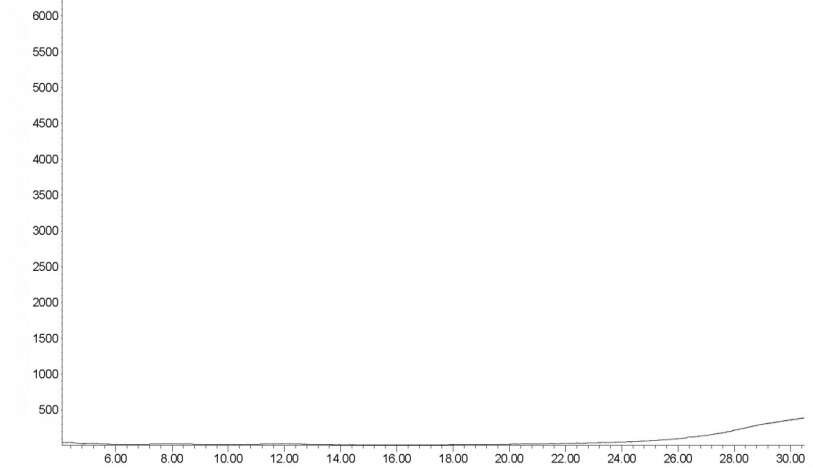


Abundance


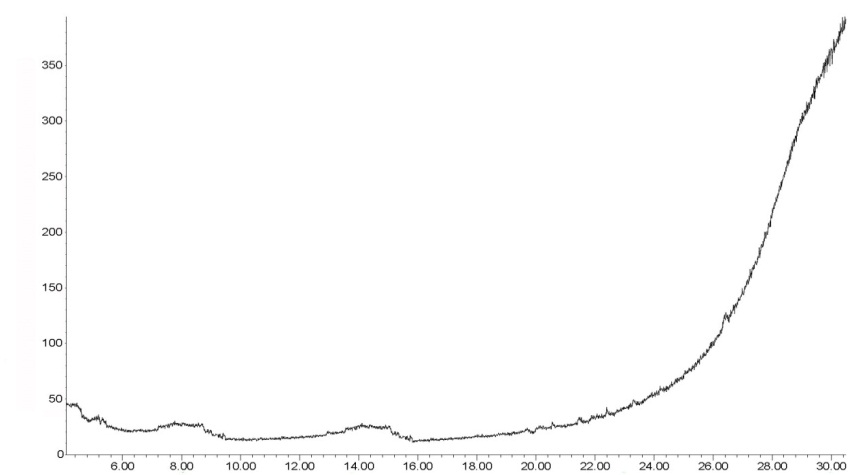


Abundance

Time (min)

**Figure H.5.** Selected ions monitoring chromatograms: *m*/*z*: 45 (organic compounds containing ethyl group), *m*/*z*: 77 (organic compounds containing phenyl group), *m*/*z*: 88 (butanoic acid), and *m*/*z*: 118 (butanedioic acid) of MBS-BC


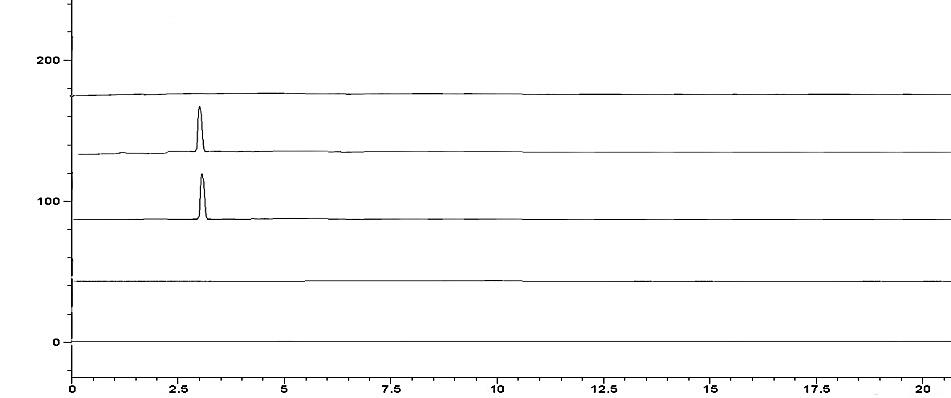


**N**

**H**

**C**

**A**

**V**

Time (min)

Abundance

**Ni**

C_6_H_x_


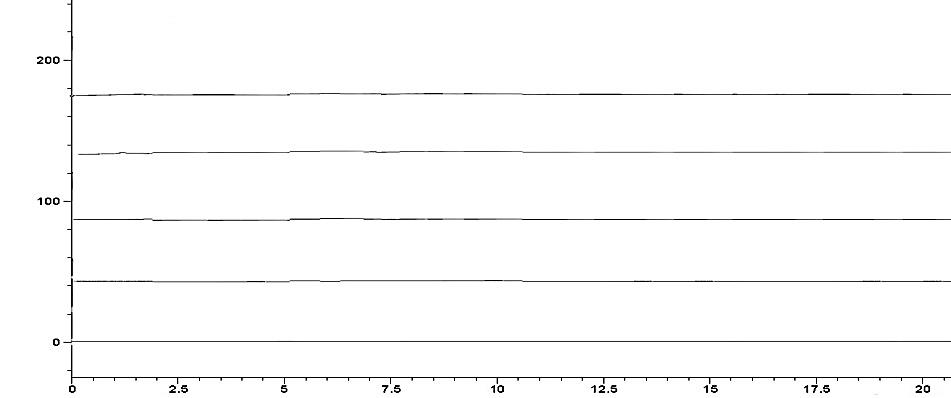


**N**

**H**

**C**

**B**

**V**

Time (min)

Abundance

**Ni**

**Figure H.6.** The atomic emission spectra of aqueous phase (A) and sediment (B) of MBS-SC.

**Figure H.7.** High-performance liquid chromatography with photodiode array detector (HPLC-PDA): 3D chromatogram (A), 425 nm chromatogram (B), and UV-Vis spectrum (C) of MBS-SC

**A**

**B**


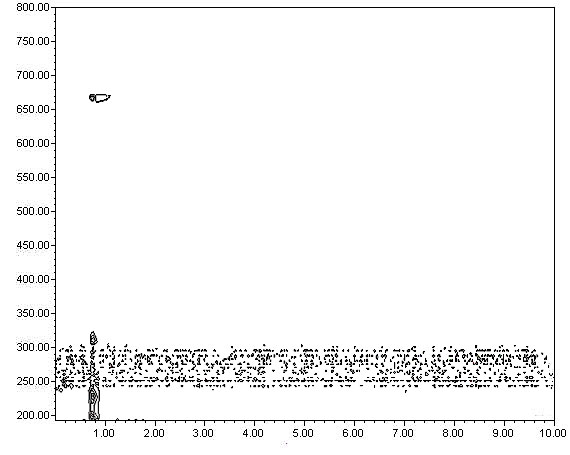


Time (min)

Wavelength (nm)

**C**


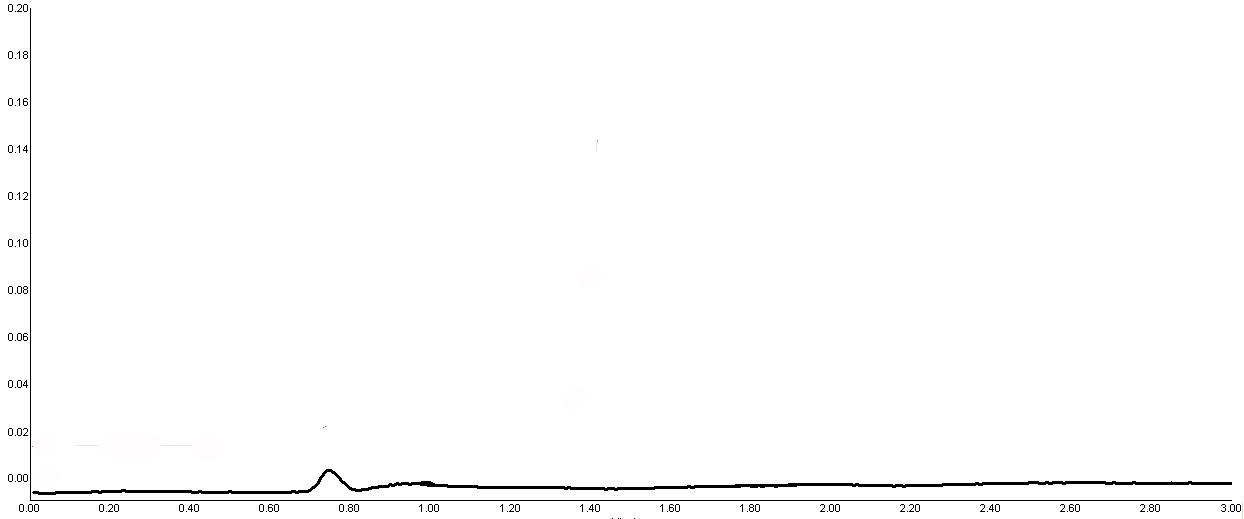


Chloroform

Abundance

Time (min)


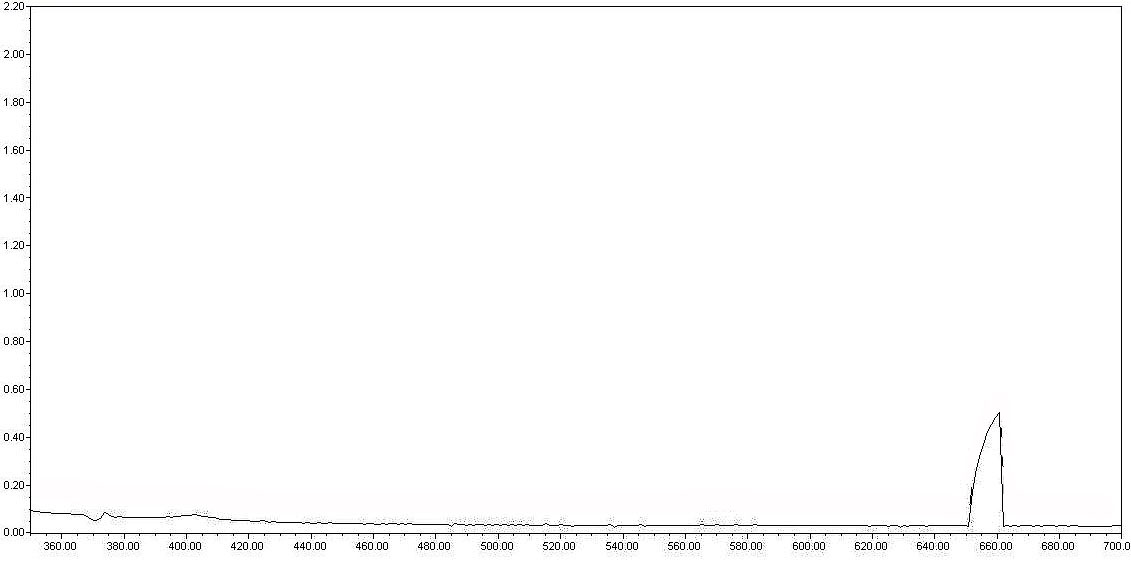


660 nm

Wavelength (nm)

Abundance

*m/z:* 201 - total peak area: 0

*m/z:* 134 - total peak area: 0

*m/z:* 67 - total peak area: 0


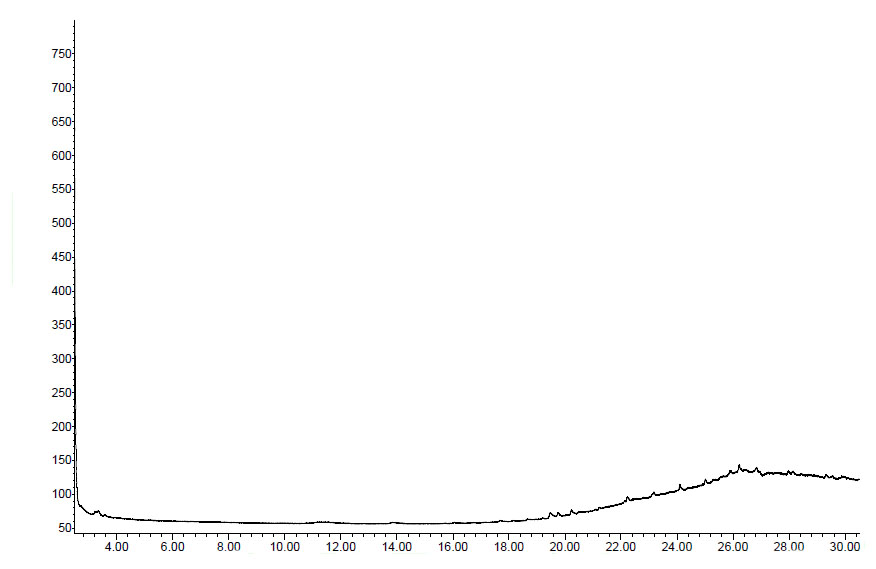


Abundance


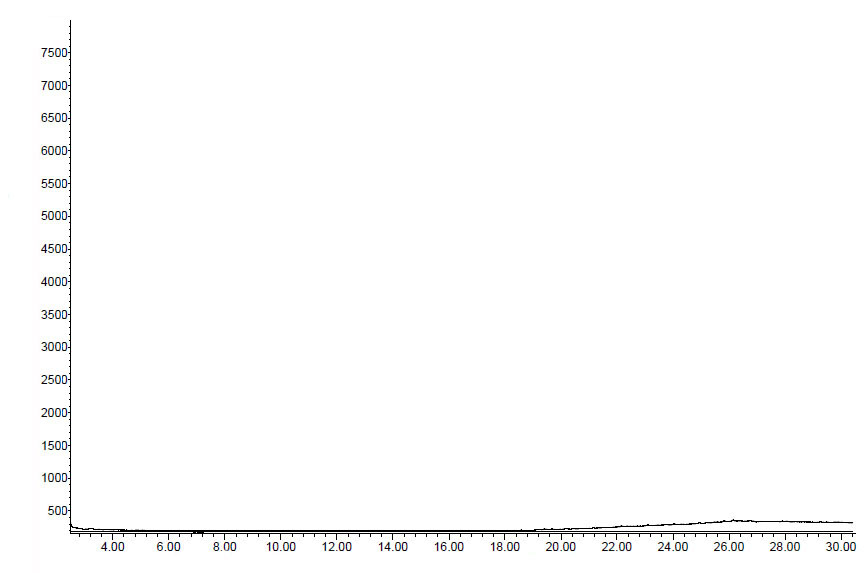


Abundance


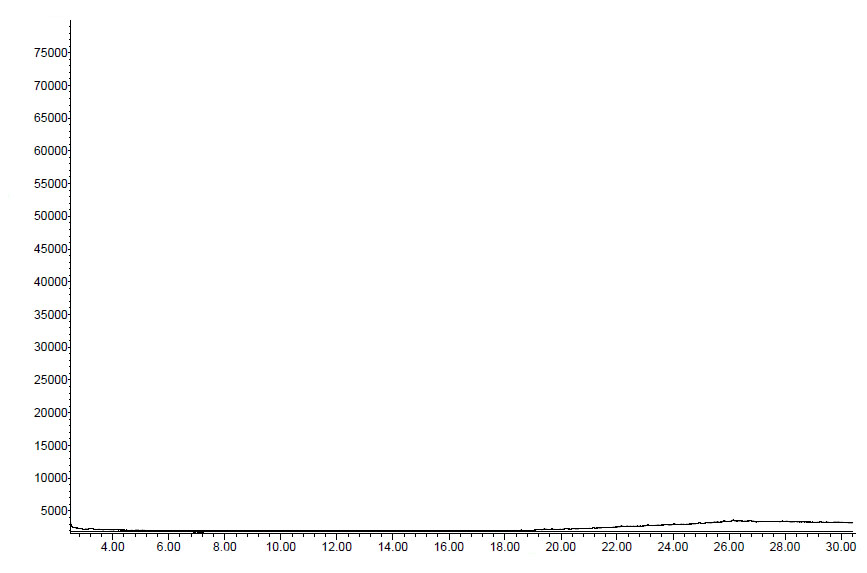


Abundance

Time (min)

**Figure H.8.** Selected ion monitoring chromatograms: *m*/*z*: 201 (organic compounds containing 3 pyrrole rings), *m*/*z*: 134 (organic compounds containing 2 pyrrole rings), and *m*/*z*: 67 (organic compounds containing 1 pyrrole rings) of MBS-SC


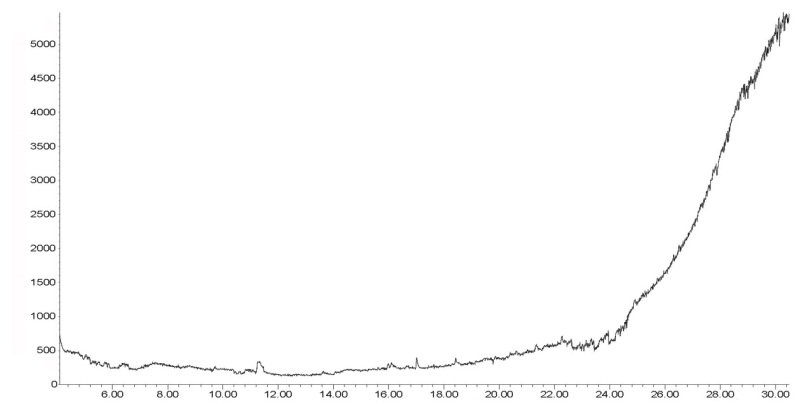


Abundance


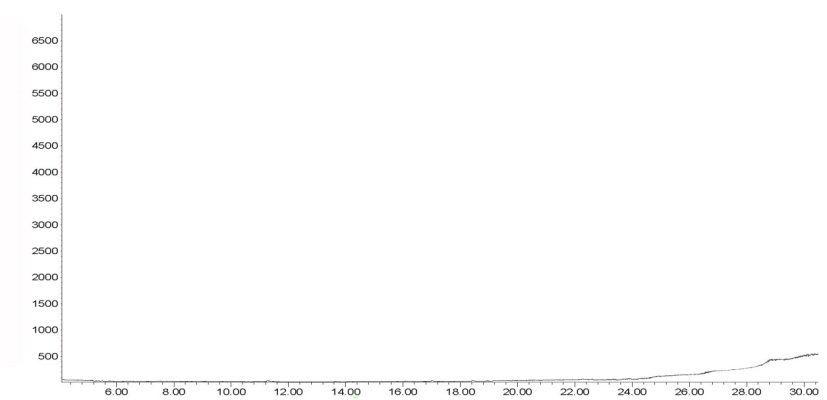


Abundance


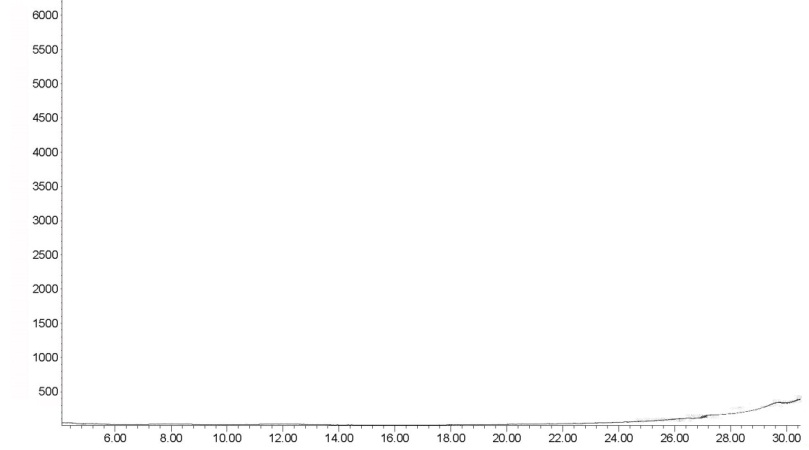


Abundance


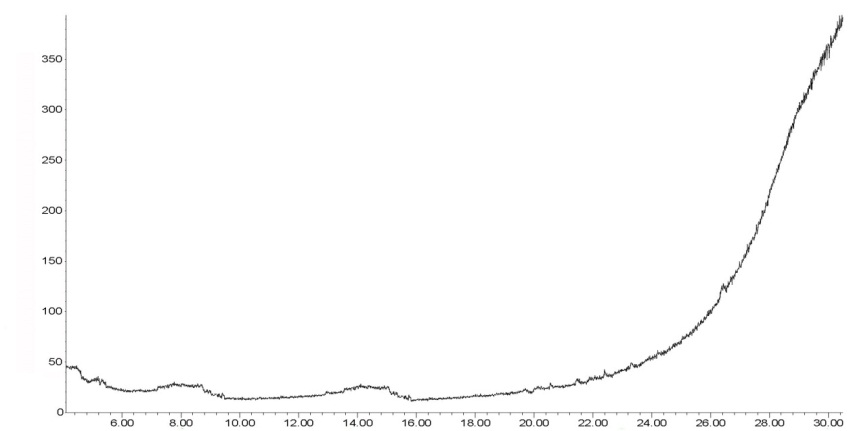


Abundance

Time (min)

**Figure H.9.** Selected ion monitoring chromatograms: *m*/*z*: 45 (organic compounds containing ethyl group), *m*/*z*: 77 (organic compounds containing phenyl group), *m*/*z*: 88 (butanoic acid), and *m*/*z*: 118 (butanedioic acid) of MBS-SC

*m/z:* 118 - total peak area: 0

*m/z:* 88 - total peak area: 0

*m/z:* 77 - total peak area: 0

*m/z:* 45 - total peak area: 0
