# Supplementary material for: Postdiagenetic Bacterial Transformation of Nickel and Vanadyl Sedimentary Porphyrins of Organic-Rich Shale Rock (Fore-Sudetic Monocline, Poland)
Source: Front Microbiol. 2021 Nov 30;12:772007. doi: 10.3389/fmicb.2021.772007 (PMC8669743; doi:10.3389/fmicb.2021.772007)
Supplement: Supplementary file 8 [file Table_8.DOCX]

**Supplementary Material G. Supplementary results for the culture of strain LM27 on mesotetraphenyl vanadyl porphyrin (VO(MTPP)-BC) and sterile control (VO(MTPP)-SC)**

**A**

**B**

| **Parameter** | **VO(MTPP)-BC** |
| --- | --- |
| CFU duplication time (days) | 1.24 |
| Maximal CFU/ml | 127x10^6^ |

**Figure G.1.** Growth of strain LM27 on medium supplemented with VO(MTPP): growth curve (A), CFU duplication time and maximal CFU (B)

**B**

**D**

**C**


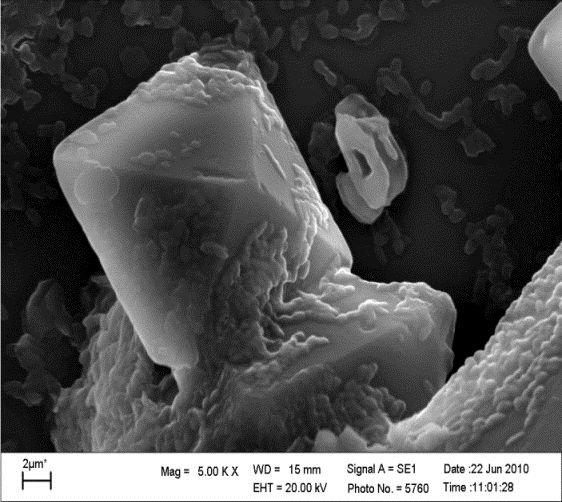

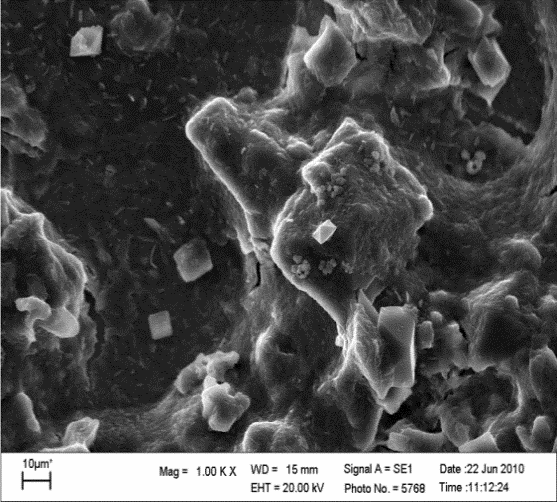

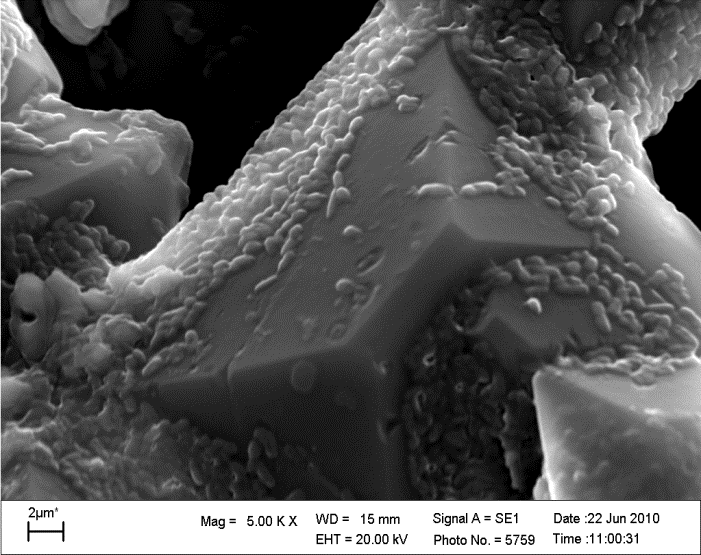

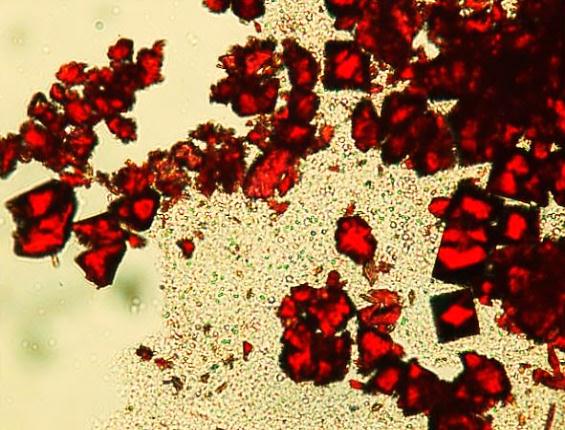

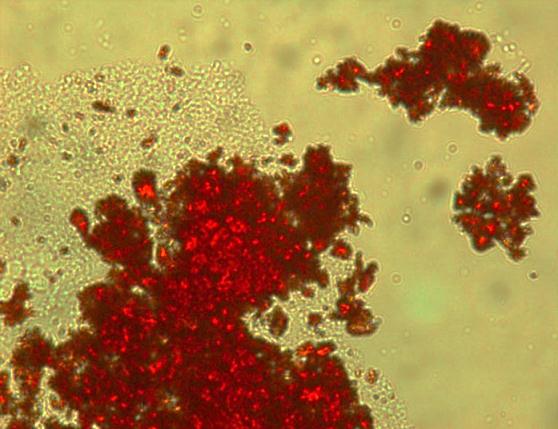


**A**

**B**

**C**

**D**

**E**

**Figure G.2.** Biofilm of strain LM27 on VO(MTPP): light (A, B) and scanning electron (C, D, E) microphotographs


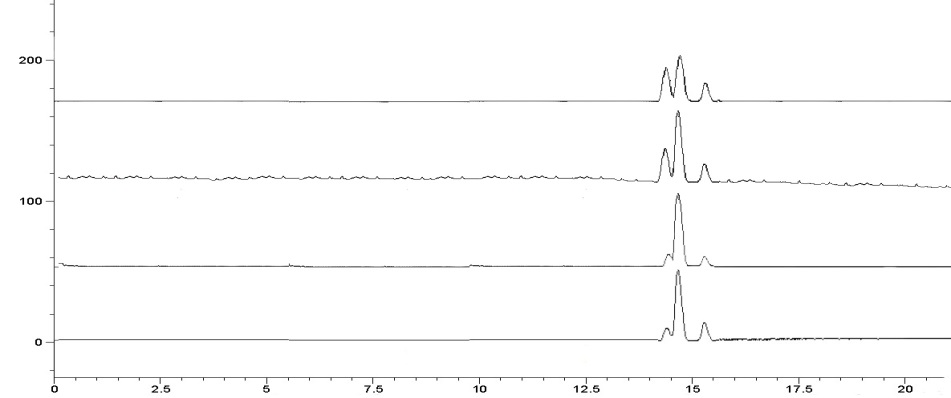


**N**

**H**

**C**

**A**

**V**O(MTPP)

Time (min)

Abundance

C_20_H_x_N_2_V

C_28_H_x_N_4_V

C_25_H_x_N_2_V

**B**


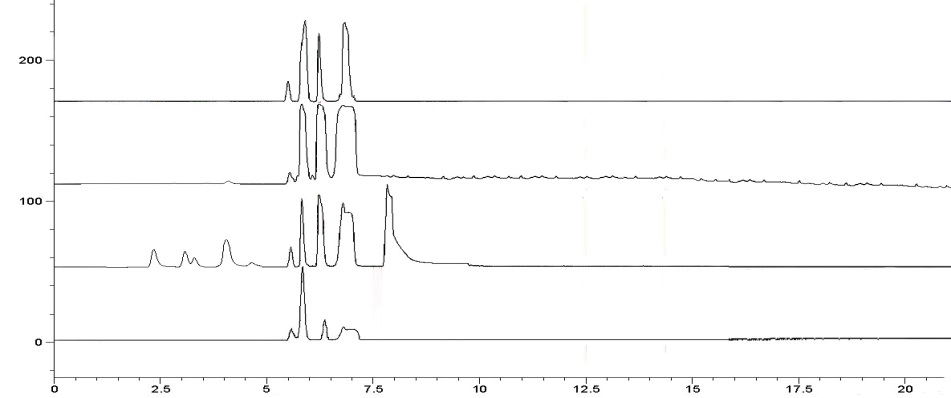


**N**

**H**

**C**

**V**MTPP)

Time (min)

Abundance

C_8_H_x_N_2_V

C_10_H_x_N_2_V

C

C_3_H_x_NV

C_2_H_x_

C_5_H_x_N_2_V

C

C

**Figure G.3.** The atomic emission spectra of aqueous phase (A) and sediment (B) of VO(MTPP)-BC


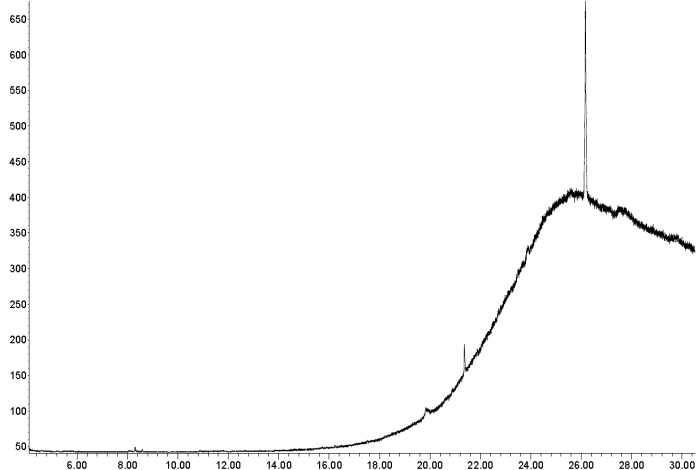


*m/z:* 361 - total peak area: 1062

Time (min)

Abundance


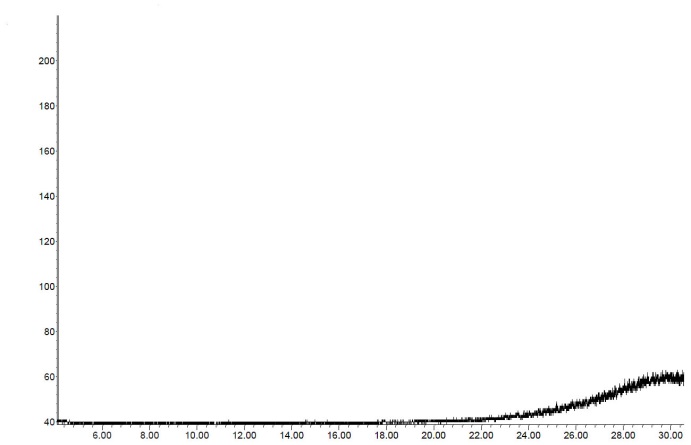


*m/z:* 679 - total peak area: 0

Time (min)

Abundance


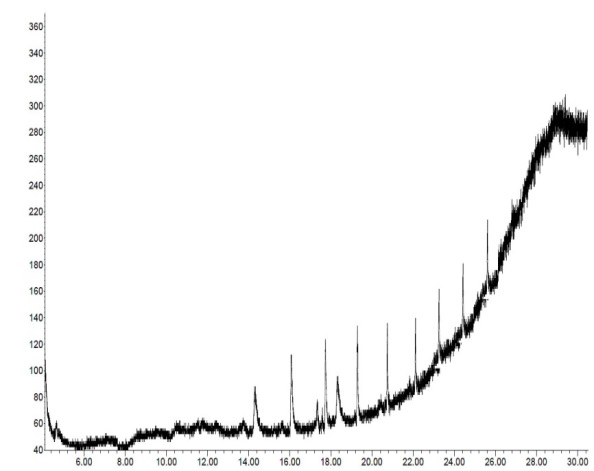


*m/z:* 472 - total peak area: 58671

Time (min)

Abundance


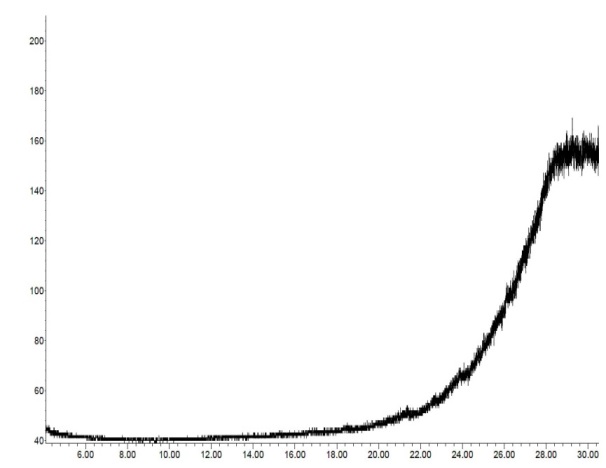


*m/z:* 528 - total peak area: 0

Time (min)

Abundance

**Figure G.4.** Selected ion monitoring chromatograms: *m*/*z*: 472 (VO(TEP)), *m*/*z*: 528 (VO(DPP)), *m*/*z*: 679 (VO(MTPP)), and *m*/*z*: 361 (VOP) of VO(MTPP)-BC


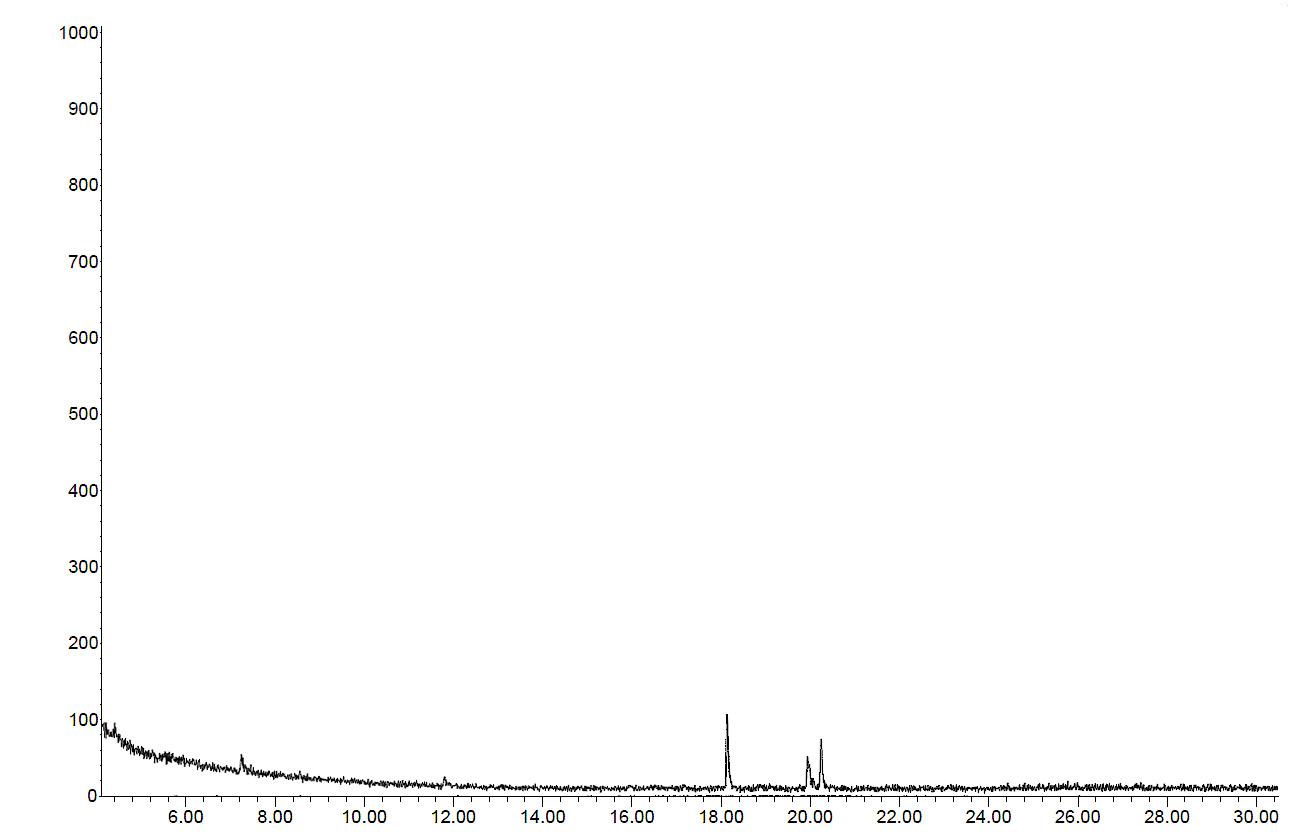


Time (min)

Abundance

20.087

20.357


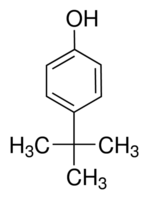


7.121

89


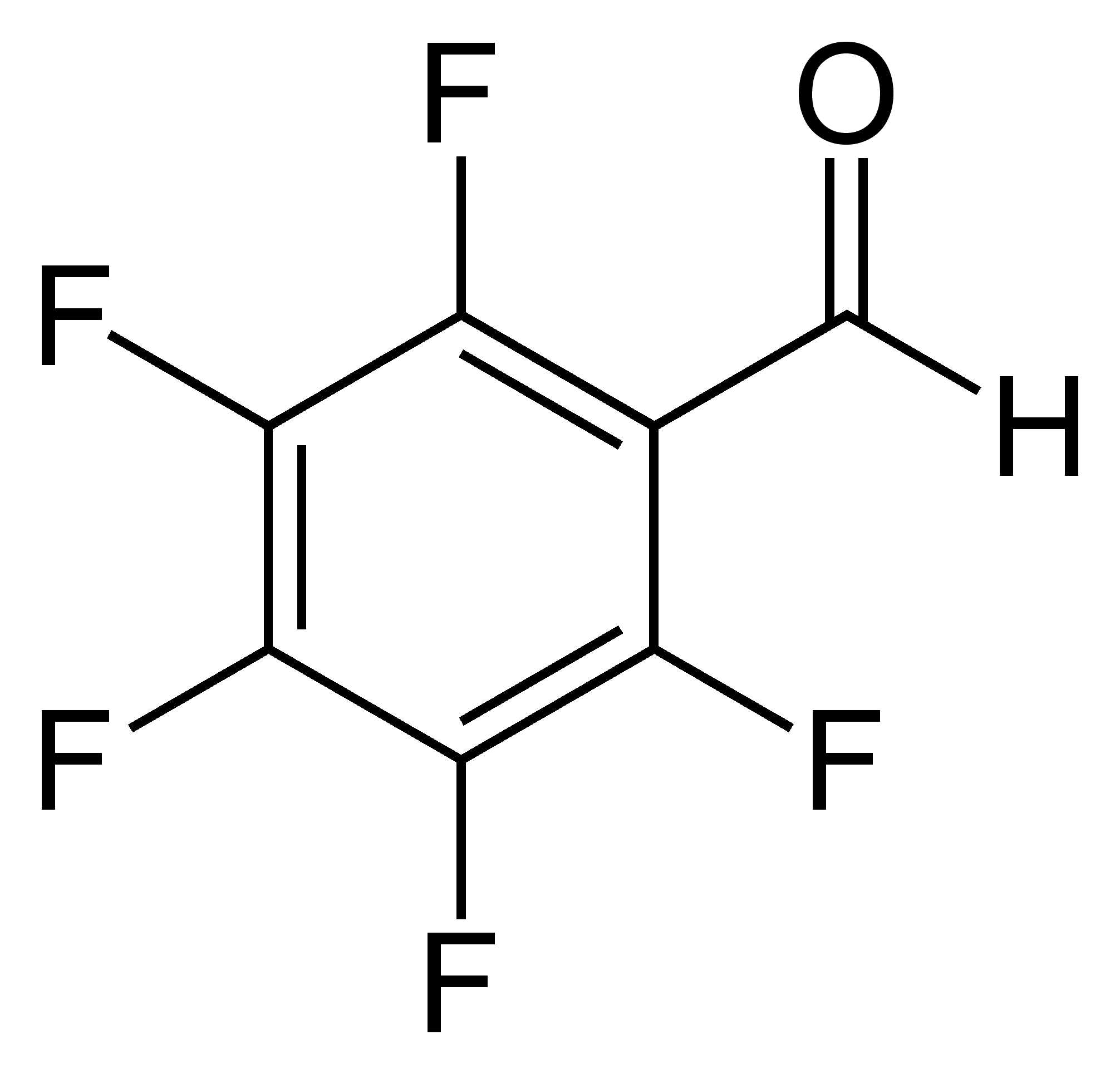


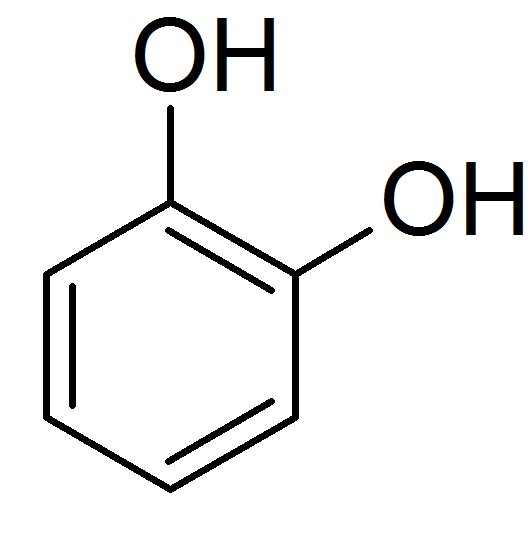


18.162

89

**A**

**B**

| **Retention time (min)** | **Organic compounds containing phenyl group** | **Peak area (%)** | **Probability.** |
| --- | --- | --- | --- |
| 7.121 | 1,2-Benzenediol | 16.99 | 95 |
| 18.162 | Pentafluorobenzaaldehyde | 36.32 | 99 |
| 20.087 | o-tert-Butylphenol | 14.39 | 98 |
| 20.357 |  | 28.66 | 99 |

**Figure G.5.** Selected ion (*m*/*z*: 77) chromatogram of VO(MTPP)-BC (A) and list of detected organic compounds containing phenyl group (B)


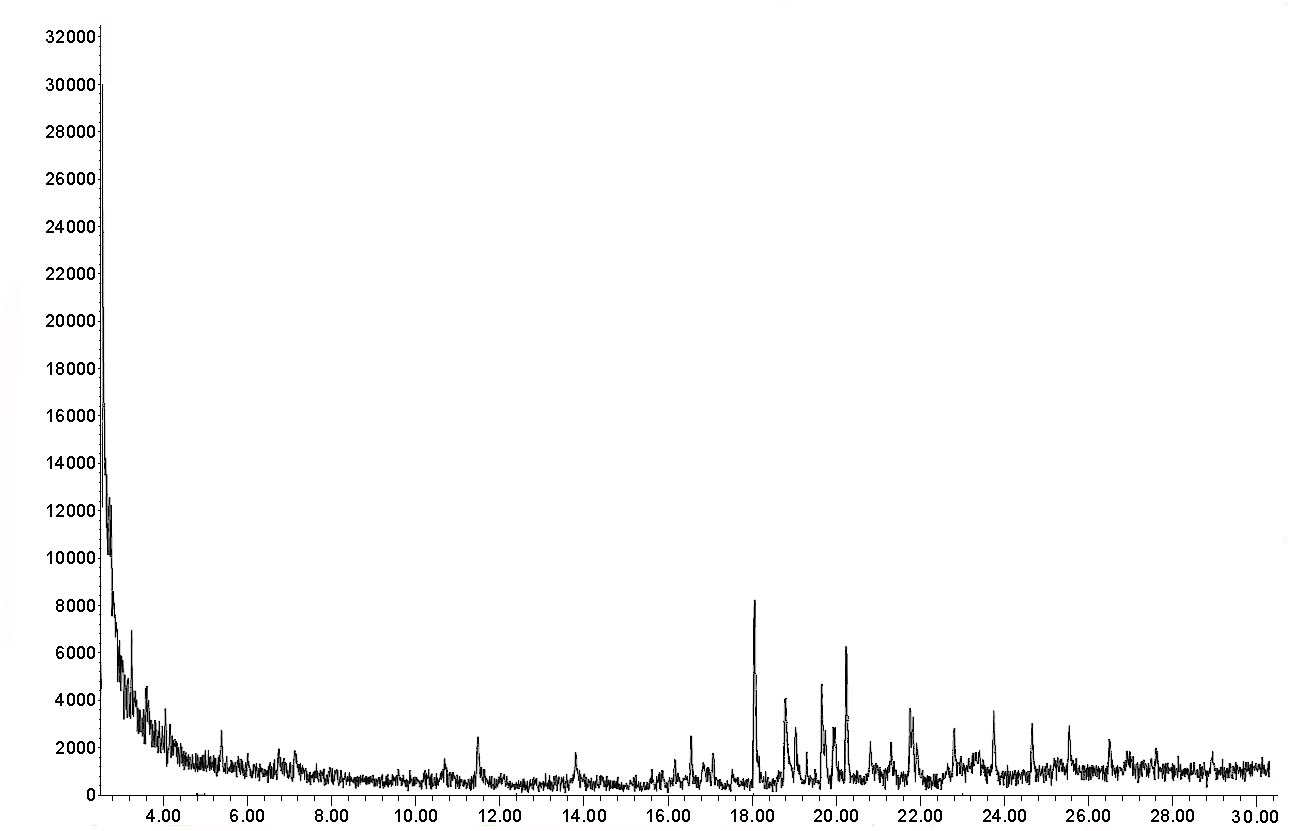


Time (min)

Abundance

11.621

89


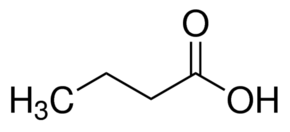


**A**

**B**

| **Retention time (min)** | **Organic compound** | **Peak area (%)** | **Probability** |
| --- | --- | --- | --- |
| 11.621 | Butanoic acid | 2.88 | 91 |

**Figure G.6.** Selected ion (*m*/*z*: 88) monitoring chromatogram of VO(MTPP)-BC (A) and detection of butanoic acid (B)


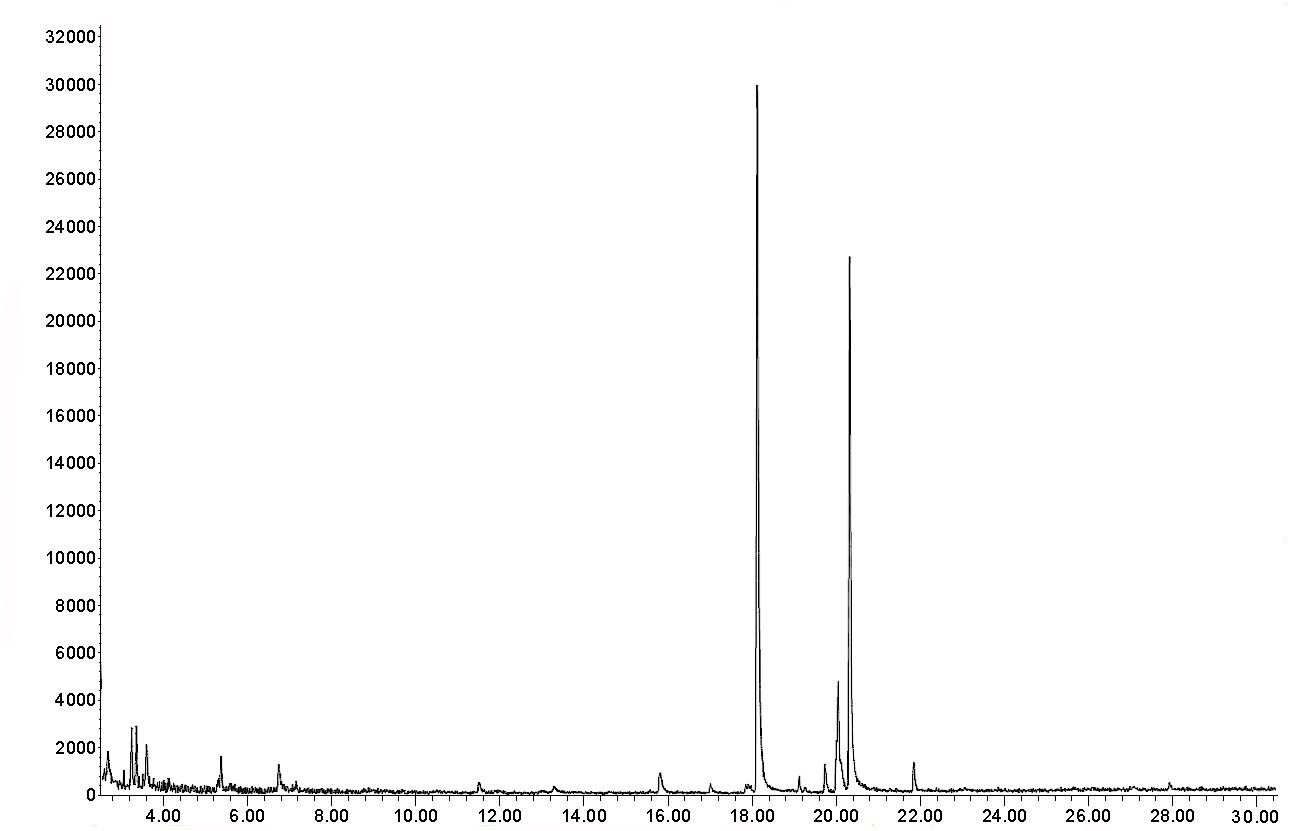


Time (min)

Abundance

20.187

20.264


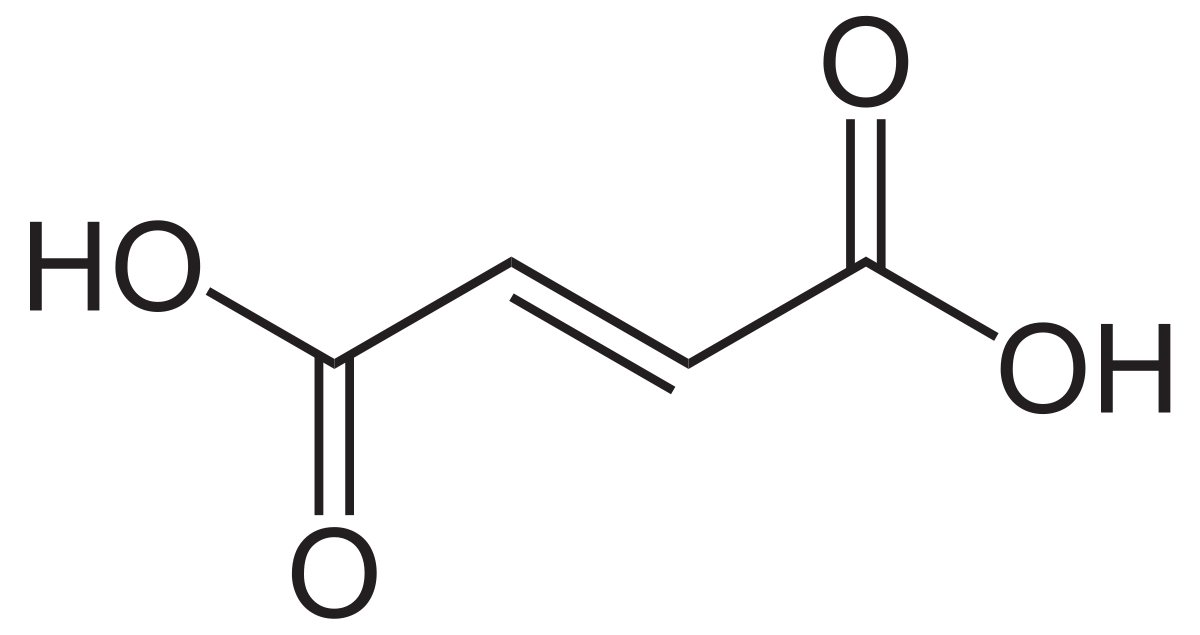


18.002

89

**A**

**B**

| **Retention time (min)** | **Organic compound** | **Peak area (%)** | **Probability** |
| --- | --- | --- | --- |
| 18.002 | Butanedioic acid | 26.22 | 90 |
| 20.187 |  | 4.71 | 91 |
| 20.264 |  | 18.21 | 91 |

**Figure G.7.** Selected ion (*m*/*z*: 118) monitoring chromatogram of VO(MTPP)-BC (A) and detection of butanedioic acid (B)


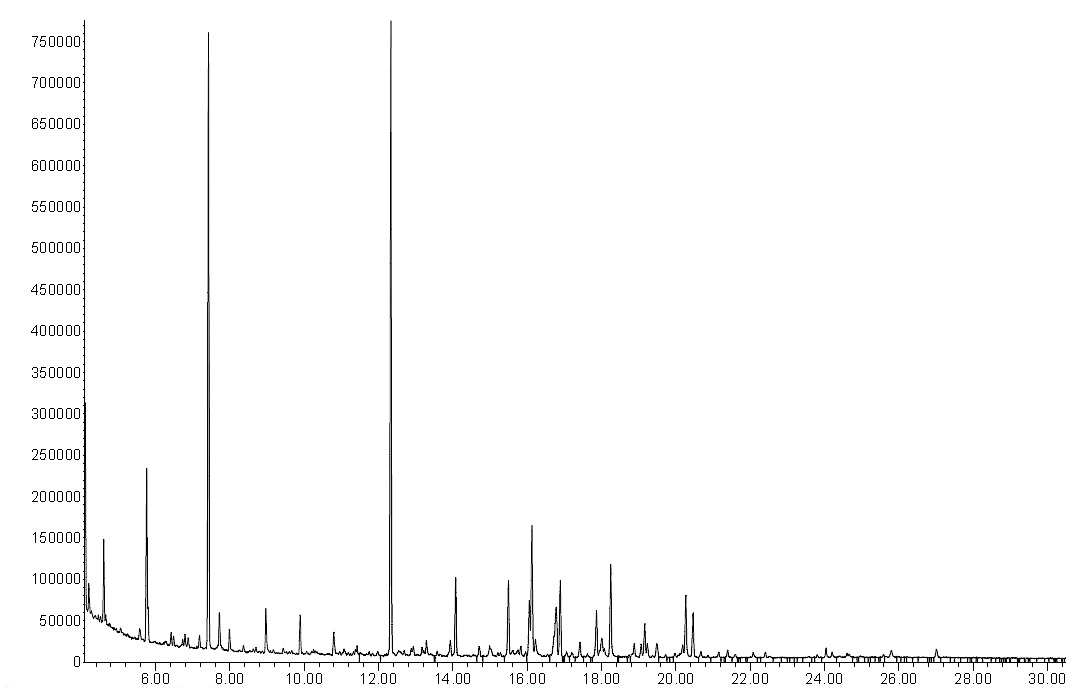


Time (min)

Abundance

**B**

4.468

14.284

6.491

6.282


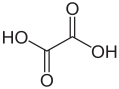


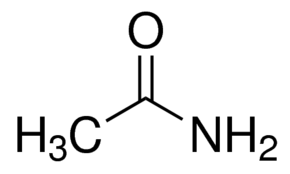


| **Retention time(min)** | **Organic compounds containg ethyl group** | **Peak area (%)** | **Probability** |
| --- | --- | --- | --- |
| 4.468 | Ethanimidic acid | 0.91 | 90 |
| 6.282 |  | 0.22 | 79 |
| 6.478 |  | 0.26 | 84 |
| 14.284 | Ethanedioic acid | 0.41 | 82 |

**Figure G.8.** Selected ion (*m*/*z*: 45) monitoring chromatogram of VO(MTPP)-BC (A) and list of detected organic compound


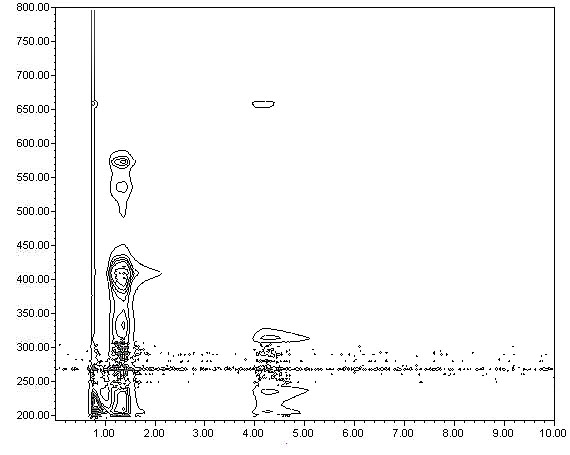


Time (min)

Wavelength (nm)

**A**


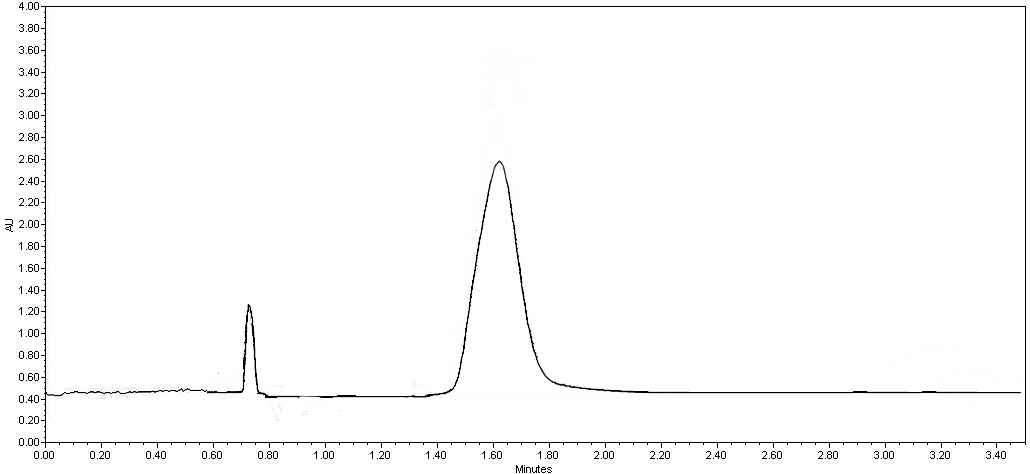


VO(MTPP)-BC

Chloroform

Abundance

Time (min)

**B**


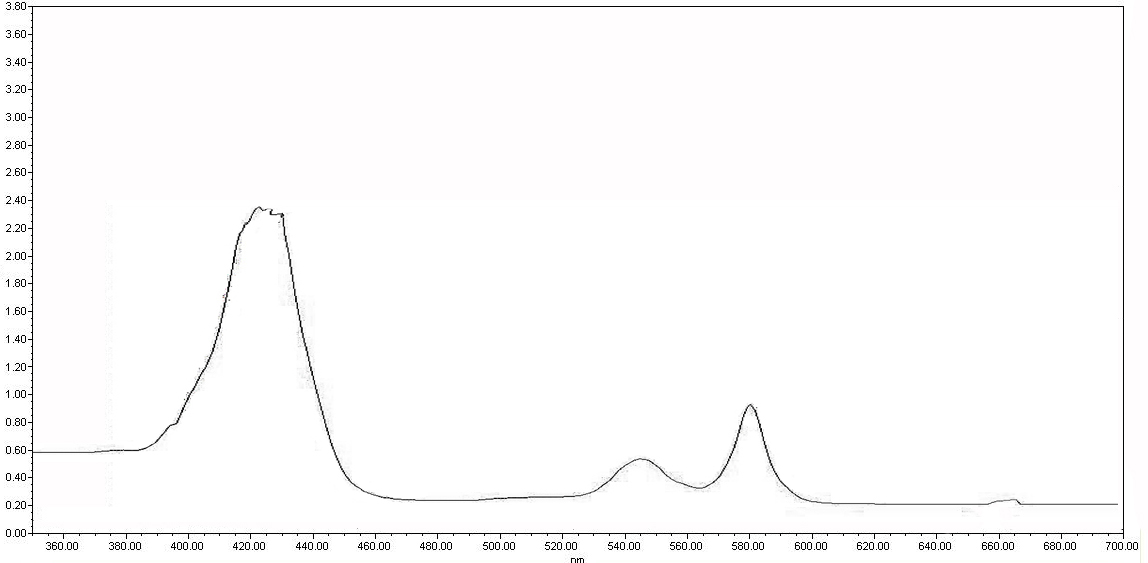


419 nm

547 nm

660 nm

Wavelength (nm)

Abundance

**C**

584 nm

**Figure G.9.** High-performance liquid chromatography with photodiode array detector (HPLC-PDA): 3D chromatogram (A), 425 nm chromatogram (B), and UV-Vis spectrum (C) of VO(MTPP)-BC

**B**


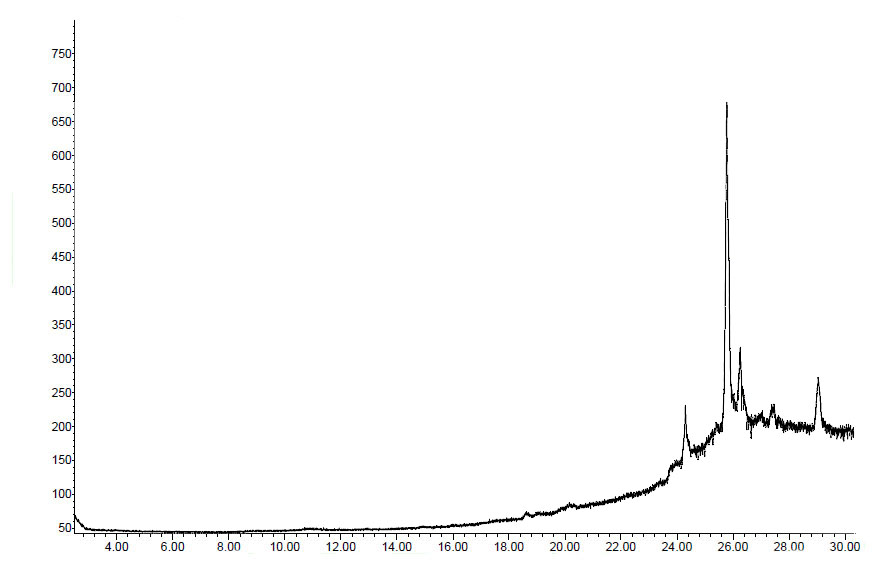


25.787


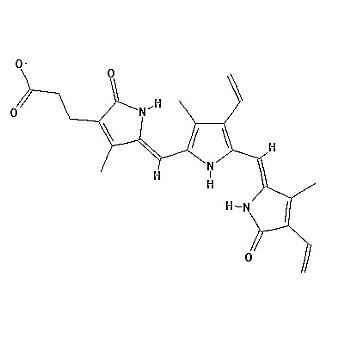


Time (min)

Abundance

**A**

| **Retention time (min)** | **Organic compounds containing 3 pyrrole ring** | **Peak area (%)** | **Probability** |
| --- | --- | --- | --- |
| 25.787 | 3-[(5Z)-5-[[4-Ethenyl-5-[(Z)-(4-ethenyl-3-methyl-5-oxopyrrol-2-ylidene)methyl]-3-methyl-1H-pyrrol-2-yl]methylidene]-4-methyl-2-oxopyrrol-3-yl]propanoate | 93.2 | 92 |

**Figure G.10.** Selected ion (*m*/*z*: 201) monitoring chromatogram of VO(MTPP)-BC (A) and list of detected organic compounds containing 3 pyrrole rings (B)

**A**


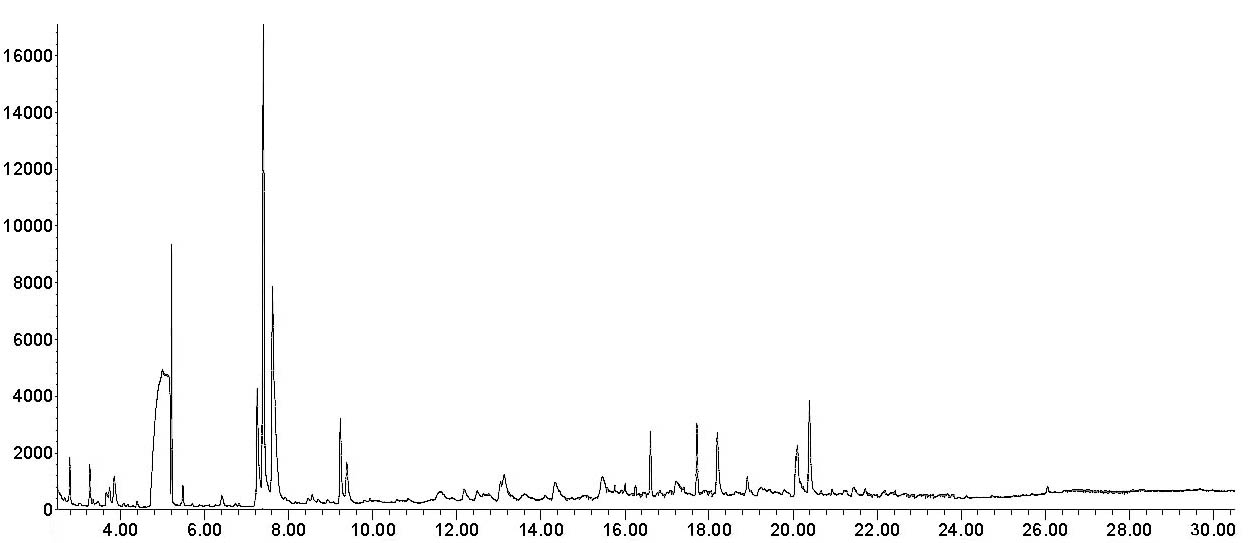


5.404

7.400

5.174

9.167

**B**

20.743


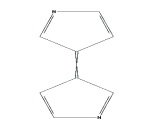


Time (min)

Abundance


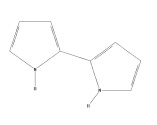


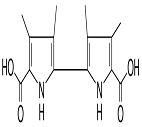


9.261

7.205

7.638


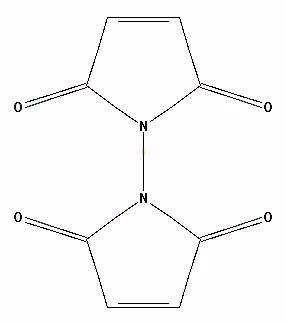


| **Retention time (min)** | **Organic compounds containing 2 pyrrole rings** | **Peak area (%)** | **Probability** |
| --- | --- | --- | --- |
| 5.174 | 3,3'-Bipyrrole | 10.0 | 99 |
| 5.404 |  | 6.7 | 87 |
| 7.205 | 2,2'-Bipyrrole | 2.7 | 92 |
| 7.400 |  | 10.3 | 96 |
| 7.638 |  | 14.2 | 91 |
| 9.167 | 1,1'-Bipyrrole-2,2',5,5'-tetraone | 11.4 | 89 |
| 9.261 |  | 6.5 | 90 |
| 20.743 | 3,3',4,4'-Tetramethyl-1H,1'H-2,2'-bipyrrole-5,5'-dicarboxylic acid | 6.6 | 99 |

**Figure G.11.** Selected ion (*m*/*z*: 134) monitoring chromatogram of VO(MTPP)-BC (A) and list of detected organic compounds containing 2 pyrrole rings (B)

**B**


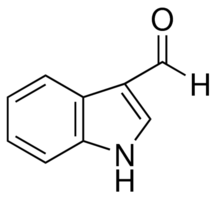


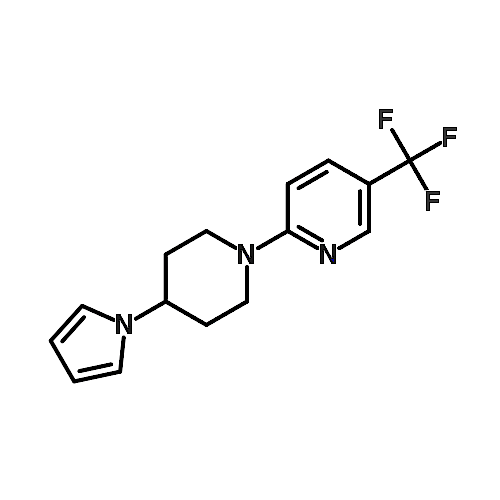


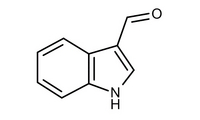


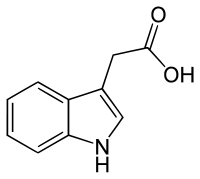


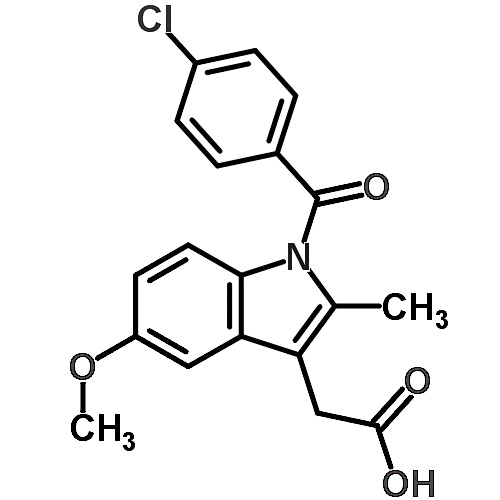


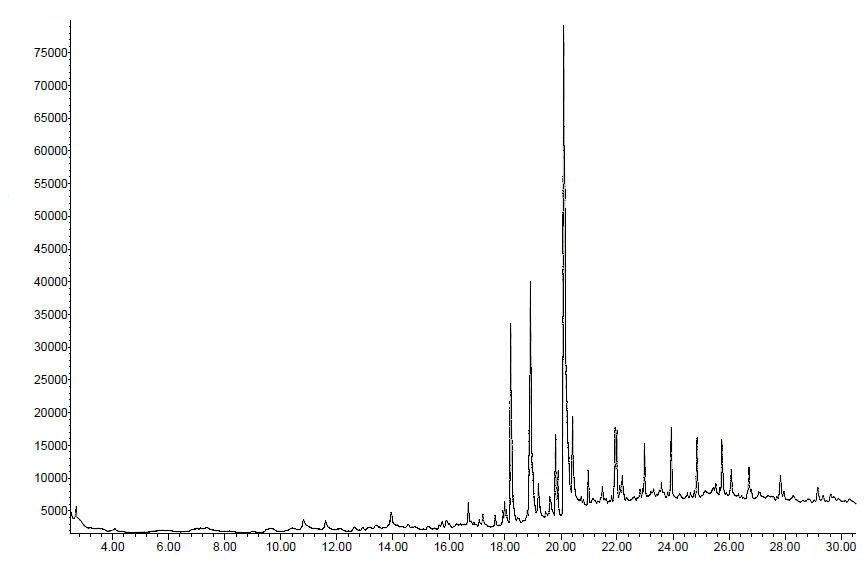


6.882

10.085

11.282

17.424

22.174

23.949

25.155

25.852

27.746

Time (min)

Abundance

19.234

23.042

20.066

20.637

**A**

C_21_H_23_N_3_O

| **Retention time (min)** | **Organic compounds containing 1 pyrrole ring** | **Peak area (%)** | **Probability** |
| --- | --- | --- | --- |
| 6.882 | 1H-Indene, 2,3-dihydro-1,1,5-trimethyl | 10.18 | 86 |
| 10.085 | 1H-Indene, 2,3-dihydro-1,4,7-trimethyl- | 1.08 | 74 |
| 11.282 | 1H-Inden-1-one, 2,3-dihydro-3,3,5,7-tetramethyl- | 5.28 | 88 |
| 17.424 | Indole acetic acid | 3.68 | 92 |
| 19.234 |  | 4.68 | 79 |
| 19.897 |  | 4.08 | 81 |
| 20.066 |  | 3.68 | 83 |
| 20.637 |  | 1.98 | 86 |
| 22.174 | Indole carbaldehyde | 5.68 | 95 |
| 23.042 |  | 4.58 | 93 |
| 23.949 | Piperidine, 1-(5-trifluoromethyl-2-pyridyl)-4-(1H-pyrrol-1-yl) | 10.18 | 71 |
| 25.155 | 2-[1-(4-Chlorobenzoyl)-5-methoxy-2-methylindol-3-yl]acetic acid | 1.08 | 75 |
| 25.852 | 2H-Pyrrol-2-one, 5-[[2-[(4-aminophenyl)methylene]-3,4-dimethyll]methylene]-3-ethyl-1,5-dihydro-4-methyl | 5.28 | 74 |
| 27.746 | Piperidine, 1-(5-trifluoromethyl-2-pyridyl)-4-1H-pyrrol-1-yl)- | 3.68 | 73 |

**Figure G.12.** Selected ion (*m*/*z*: 67) monitoring chromatogram of VO(MTPP)-BC (A) and list of detected organic compounds containing 1 pyrrole ring (B)


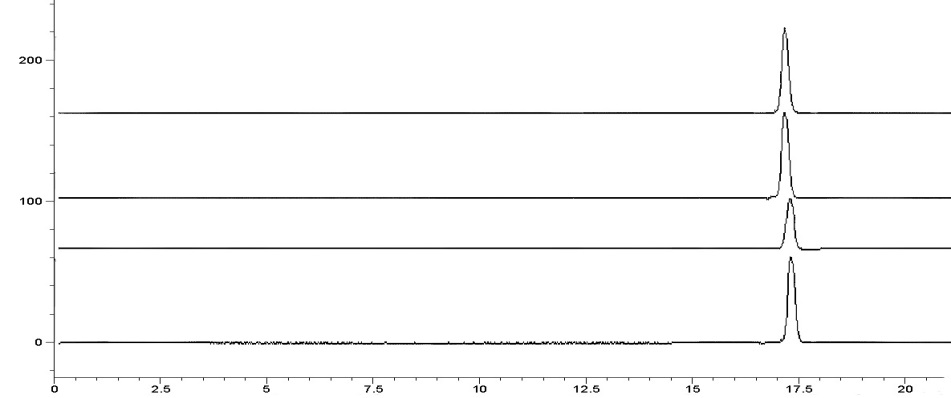


**N**

**H**

**C**

**A**A

**V**

Time (min)

Abundance

C_32_H_x_N_4_V


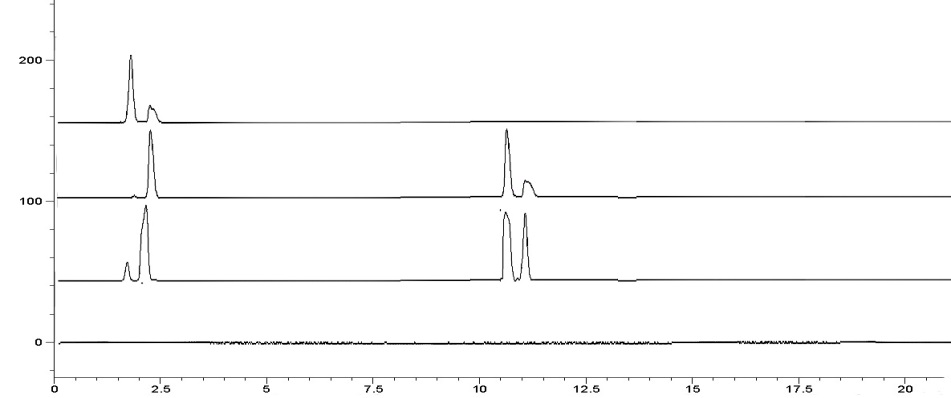


**N**

**H**

**C**

**V**

Time (min)

Abundance

C_10_Hx

C_2_H_x_N

CHN

C_12_H_x_

**Figure G.13.** The atomic emission spectra of aqueous phase (A) and sediment (B) of VO(MTPP)-SC


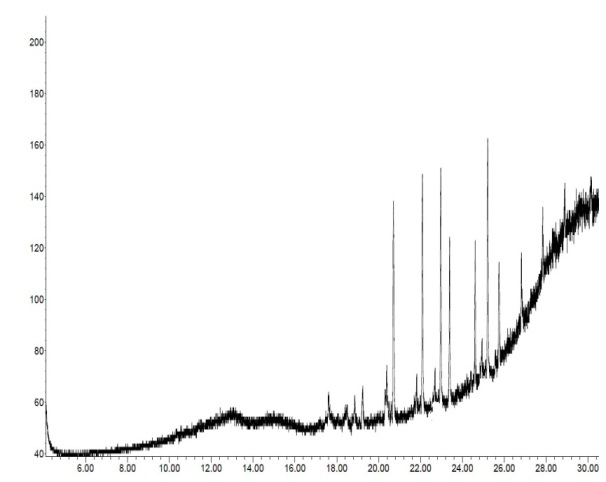


*m/z:* 528 - total peak area: 68241

Time (min)

Abundance


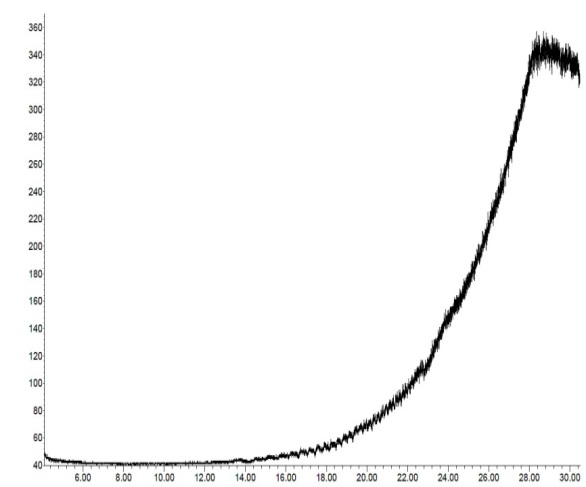


*m/z:* 472 - total peak area: 0

Time (min)

Abundance


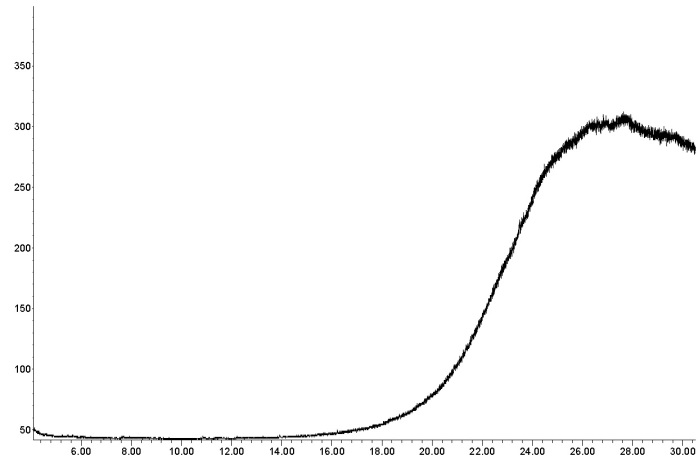


*m/z:* 361 - total peak area: 0

Time (min)

Abundance


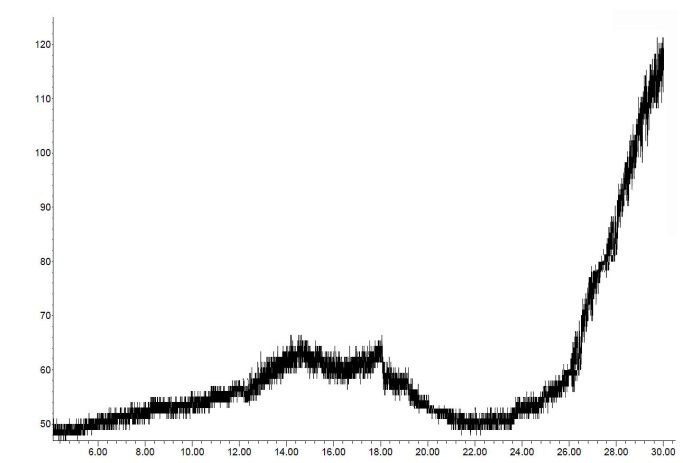


*m/z:* 679 - total peak area: 0

Time (min)

Abundance

**Figure G.14.** Selected ion monitoring chromatograms: *m*/*z*: 472 (VO(TEP)), *m*/*z*: 528 (VO(DPP)), *m*/*z*: 679 (VO(MTPP)), and *m*/*z* 361 (VOP) of VO(MTPP)-SC


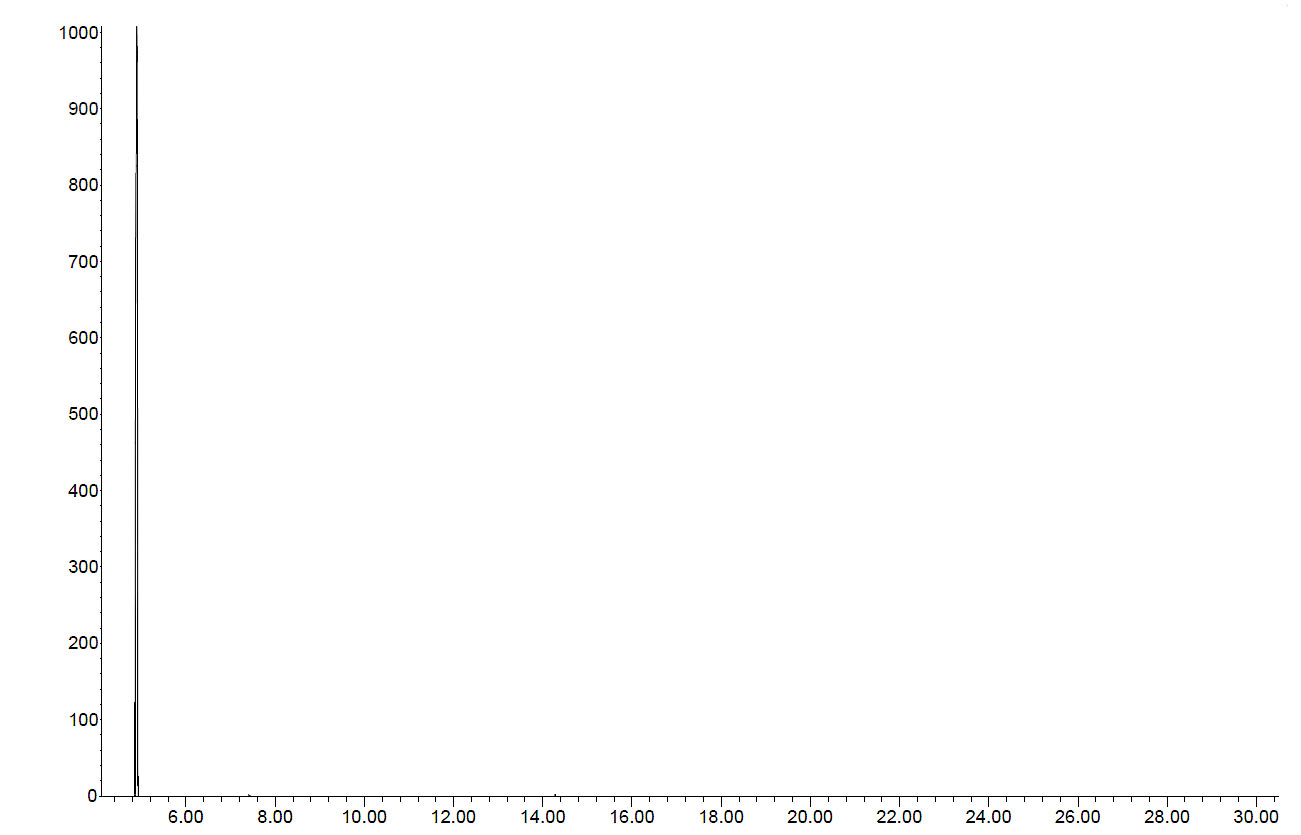


Time (min)

Abundance

**A**

4.995

**B**

| **Retention time (min)**  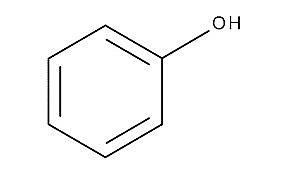 | **Organic compound containing phenyl group** | **Peak area (%)** | **Probability** |
| --- | --- | --- | --- |
| 4.995 | Benzenol | 100 | 85 |

**Figure G.15.** Selected ion (*m*/*z*: 77) chromatogram of VO(MTPP)-SC (A) and list detected organic compounds containing phenyl group (B)

*m/z:* 88 - total peak area: 0


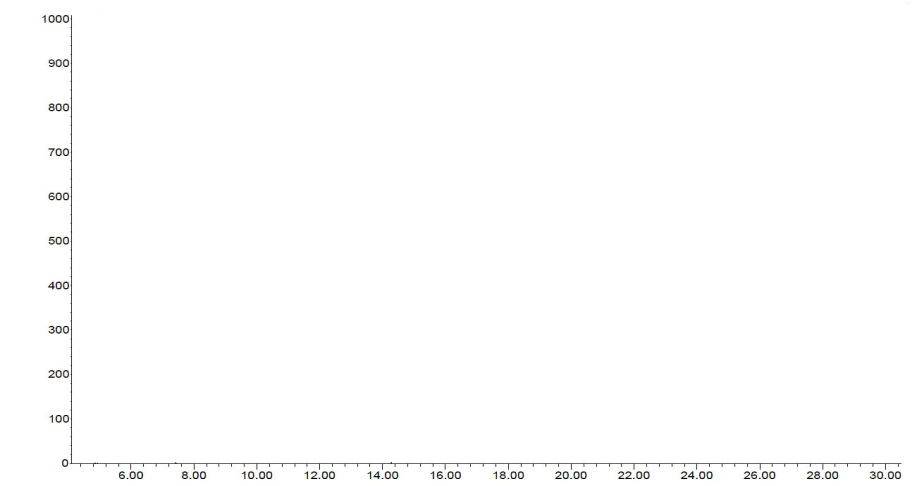


Time (min)

Abundance

*m/z:* 118 - total peak area: 0


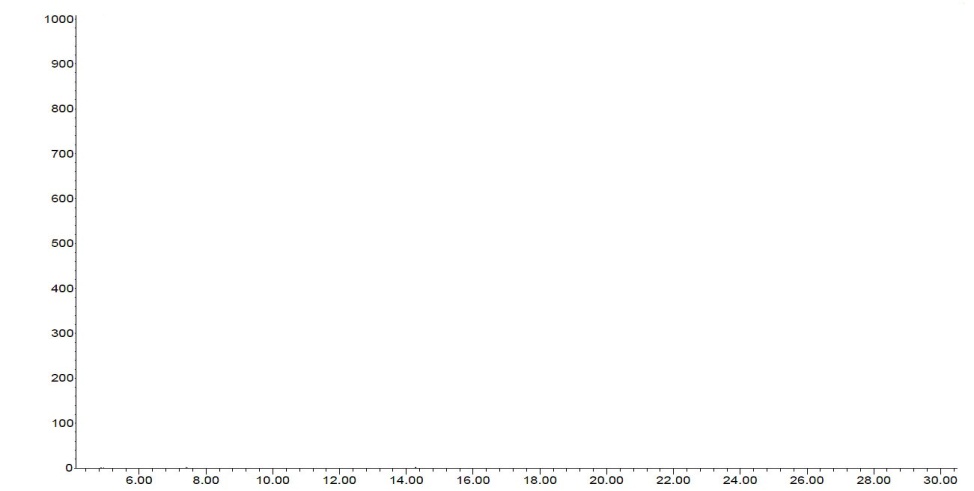


Time (min)

Abundance

*m/z:* 45 - total peak area: 0


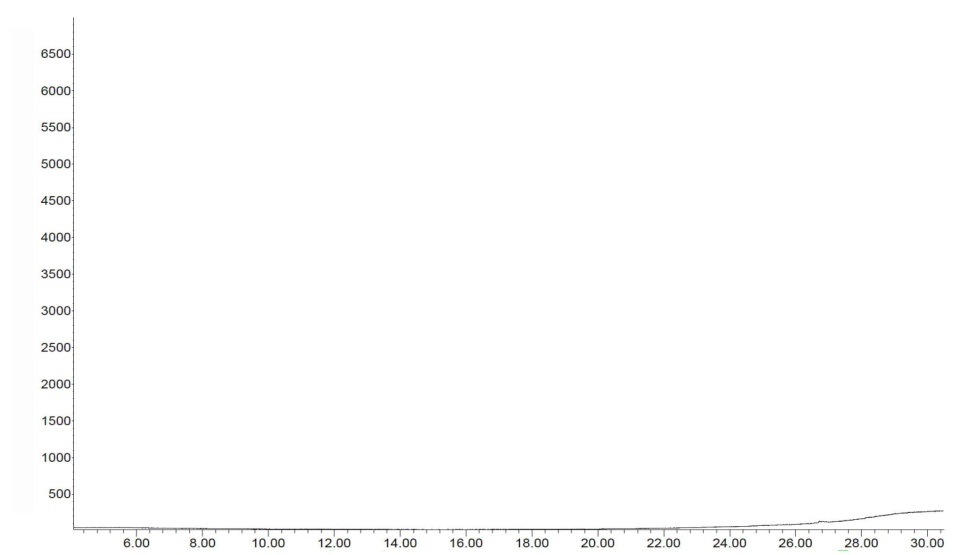


Time (min)

Abundance

**Figure G.16.** Selected ion monitoring chromatograms: *m*/*z*: 88 (butanoic acid), *m*/*z*: 118 (butanedioic acid), and *m*/*z*: 45 (ethyl group) of VO(MTPP)-SC


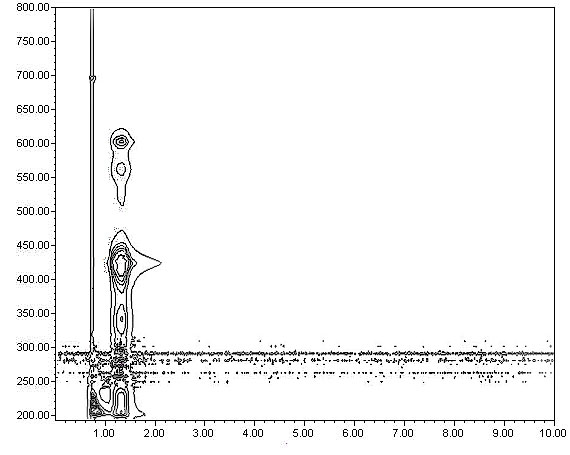


Time (min)

Wavelength (nm)

**A**


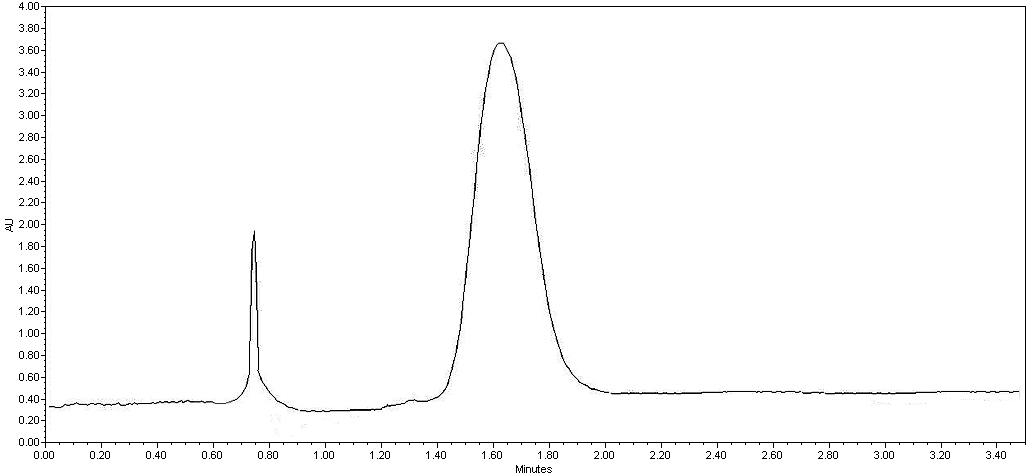


VO(MTPP)-SC

Chloroform

(425 nm)

Abundance

Time (min)

**B**


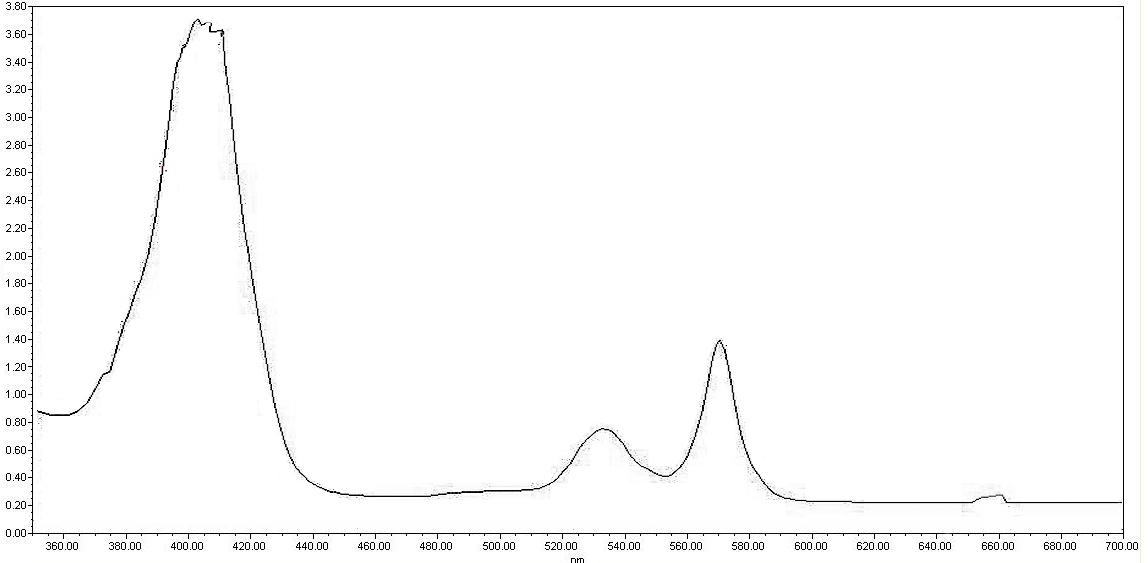


401 nm

529 nm

571 nm

660 nm

Wavelength (nm)

Abundance

**C**

**Figure G.17.** High-performance liquid chromatography with photodiode array detector (HPLC-PDA): 3D chromatogram (A), 425 nm chromatogram (B) and UV-Vis spectrum, (C) of VO(MTPP)-SC

*m/z:*201 - total peak area: 0

*m/z:*134 - total peak area: 0


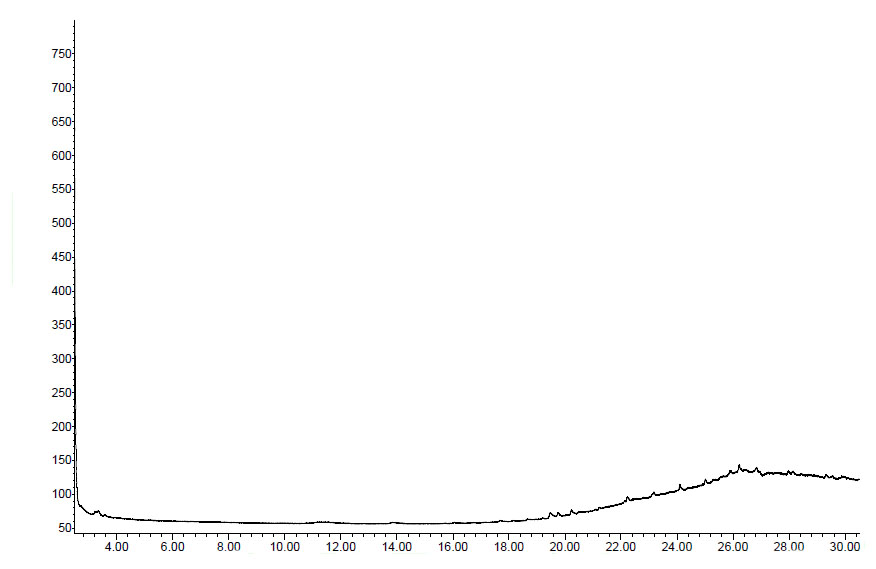


Abundance

Time (min)


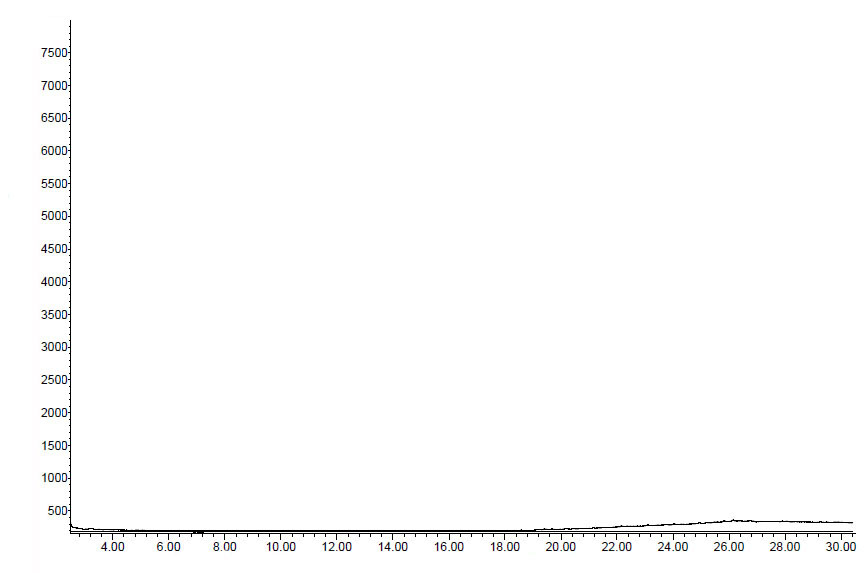


Abundance

Time (min)

*m/z:* 67 - total peak area: 0


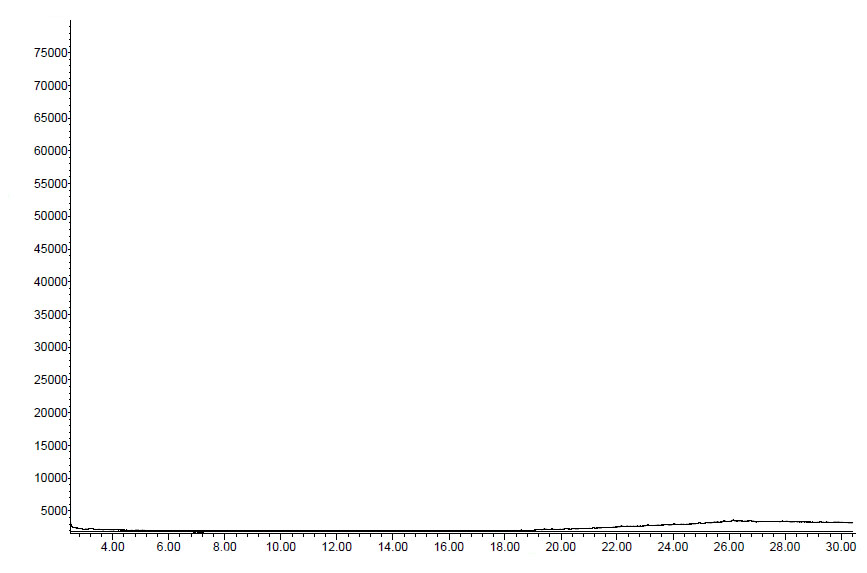


Abundance

Time (min)

**Figure G.18.** Selected ions monitoring chromatograms: *m*/*z*: 201 (organic compounds containing 3 pyrrole rings), *m*/*z*: 134 (organic compounds containing 2 pyrrole rings), and *m*/*z*: 67 (organic compounds containing 1 pyrrole ring) of VO(MTPP)-SC
