# Supplementary material for: Postdiagenetic Bacterial Transformation of Nickel and Vanadyl Sedimentary Porphyrins of Organic-Rich Shale Rock (Fore-Sudetic Monocline, Poland)
Source: Front Microbiol. 2021 Nov 30;12:772007. doi: 10.3389/fmicb.2021.772007 (PMC8669743; doi:10.3389/fmicb.2021.772007)
Supplement: Supplementary file 10 [file Data_Sheet_1.pdf]

Supplementary Material I. Mass spectra

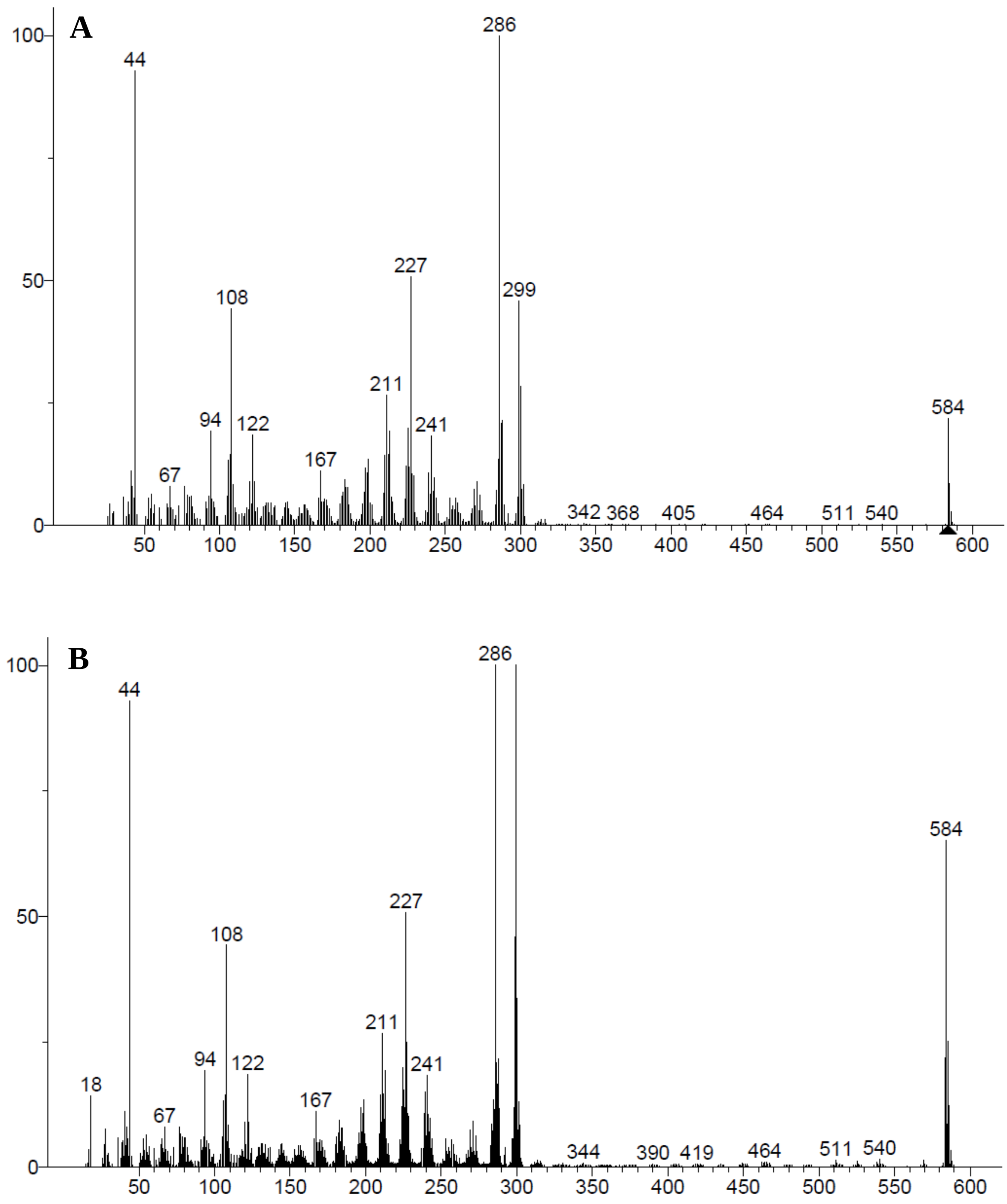

Figure I.1. Mass spectrum of 3-[2-[[3-(2-carboxyethyl)-5-[(3.4-dimethyl-5-oxopyrrol-2-ylidene)methyl]-4-methyl-1H-pyrrol-2-yl]methylidene]-4-methyl-5-oxopyrrol-3-yl]propanoic acid: mass spectrum from Wiley library(A), mass spectrum from total ion current chromatogram (B)

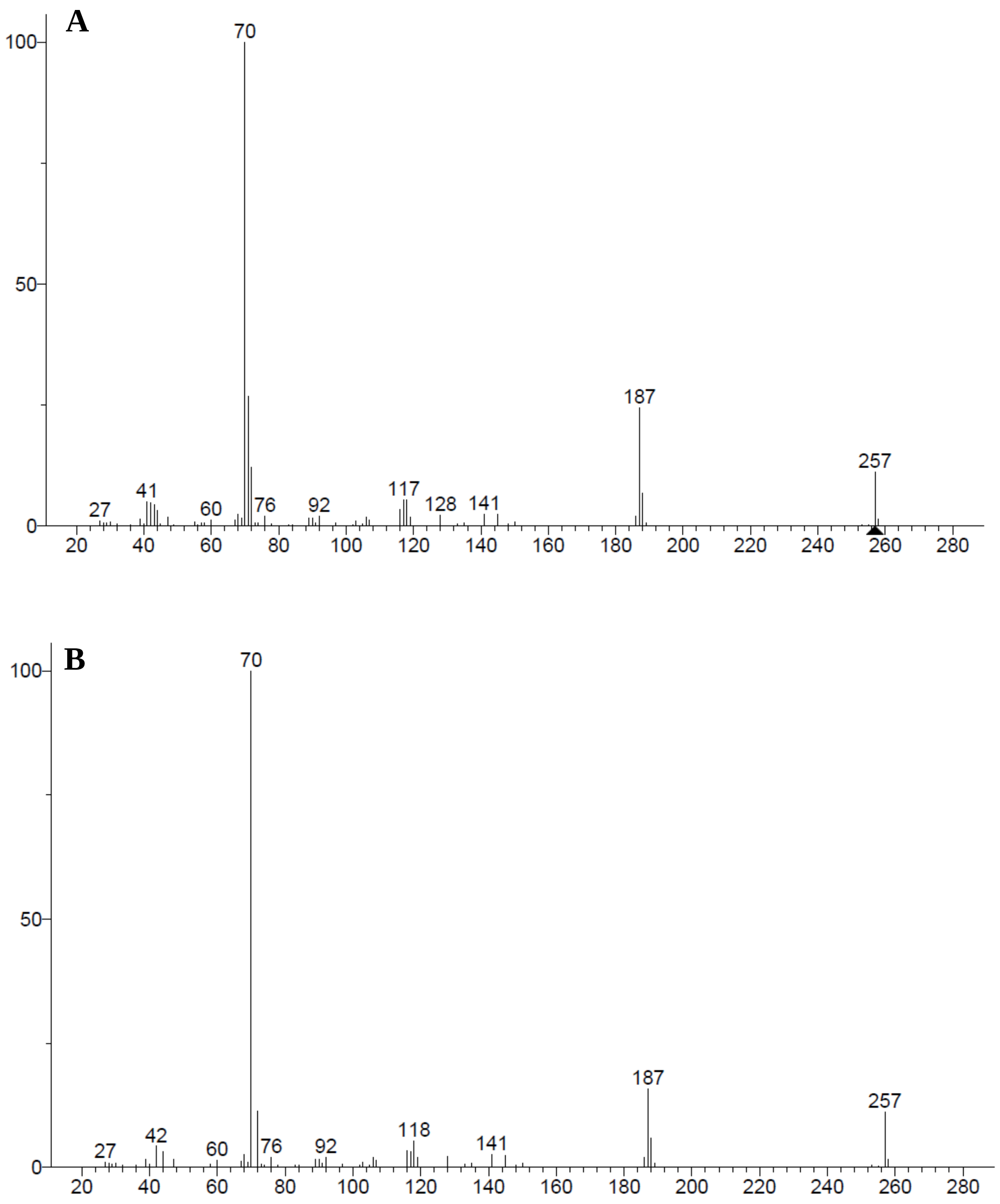

Figure I.2. Mass spectrum of 3-[(5Z)-5-[[4-ethenyl-5-[(Z)-(4-ethenyl-3-methyl-5-oxopyrrol-2-ylidene)methyl]-3-methyl-1H-pyrrol-2-yl]methylidene]-4-methyl-2-oxopyrrol-3-yl]propanoate: (A) mass spectrum from Wiley library, (B) mass spectrum from total ion current chromatogram

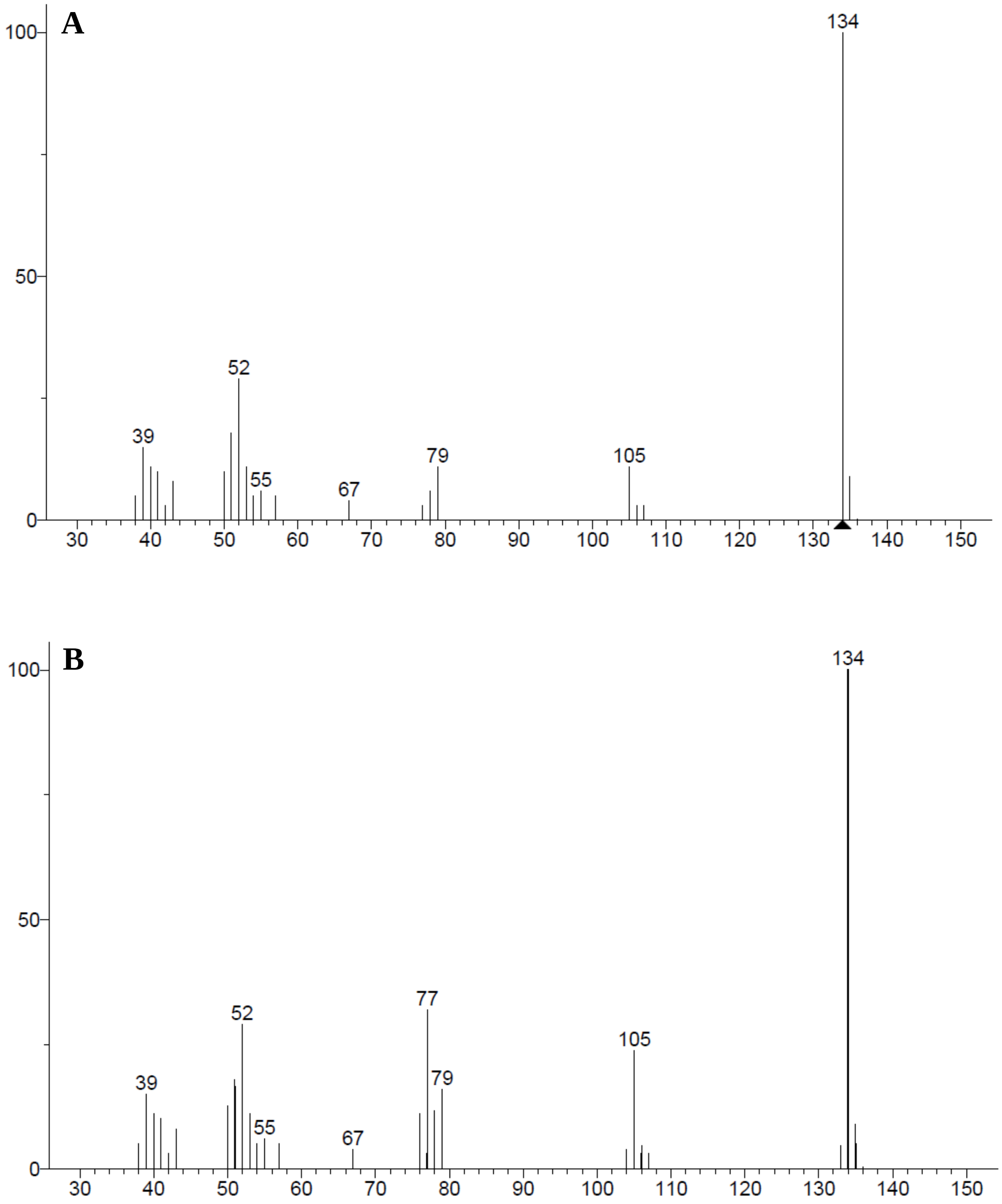

Figure I.3. Mass spectrum of 3.3'-bipyrrole: mass spectrum from Wiley library(A), mass spectrum from total ion current chromatogram (B)

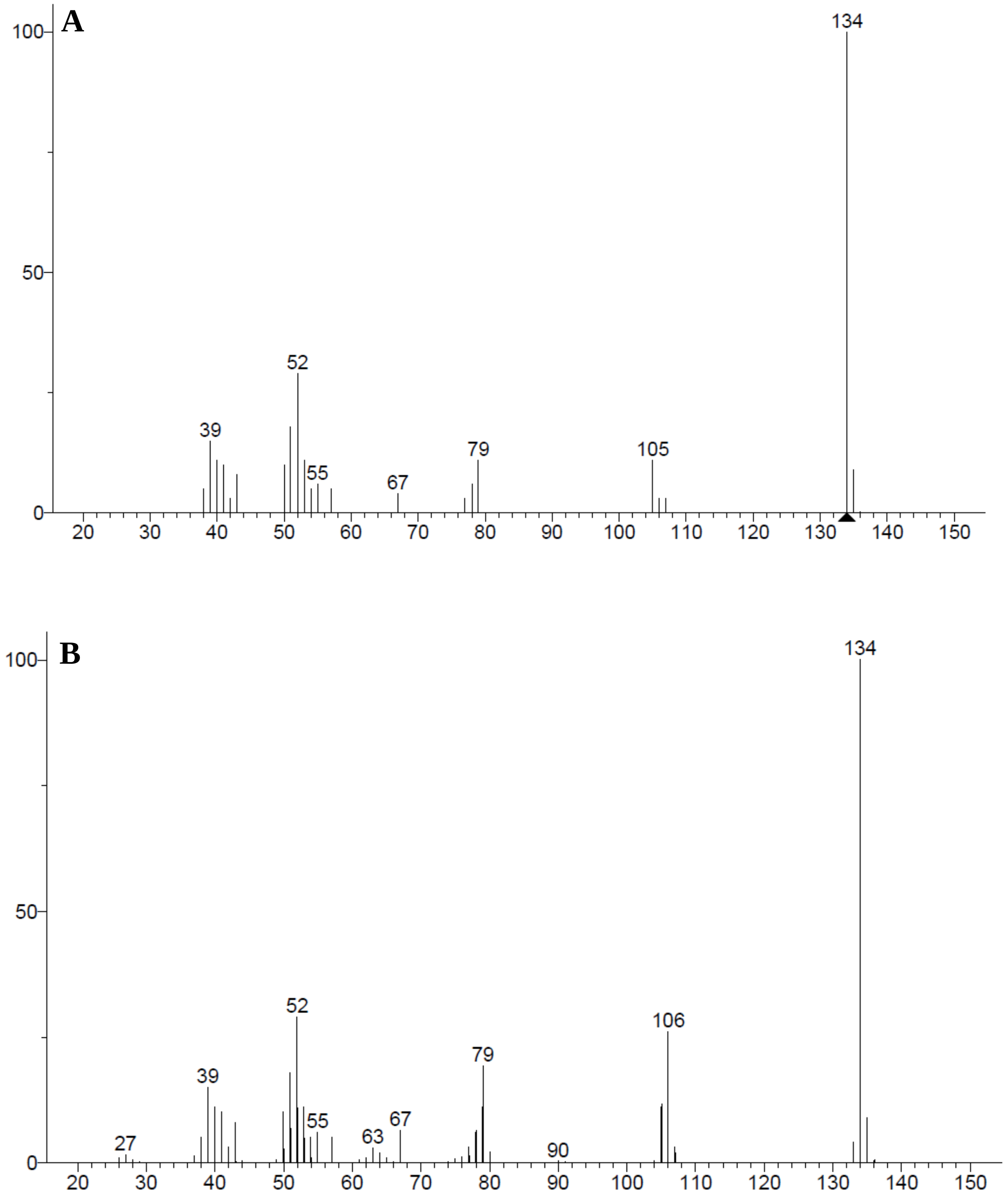

Figure I.4. Mass spectrum of 2.2'-bipyrrole: mass spectrum from Wiley library(A), mass spectrum from total ion current chromatogram (B)

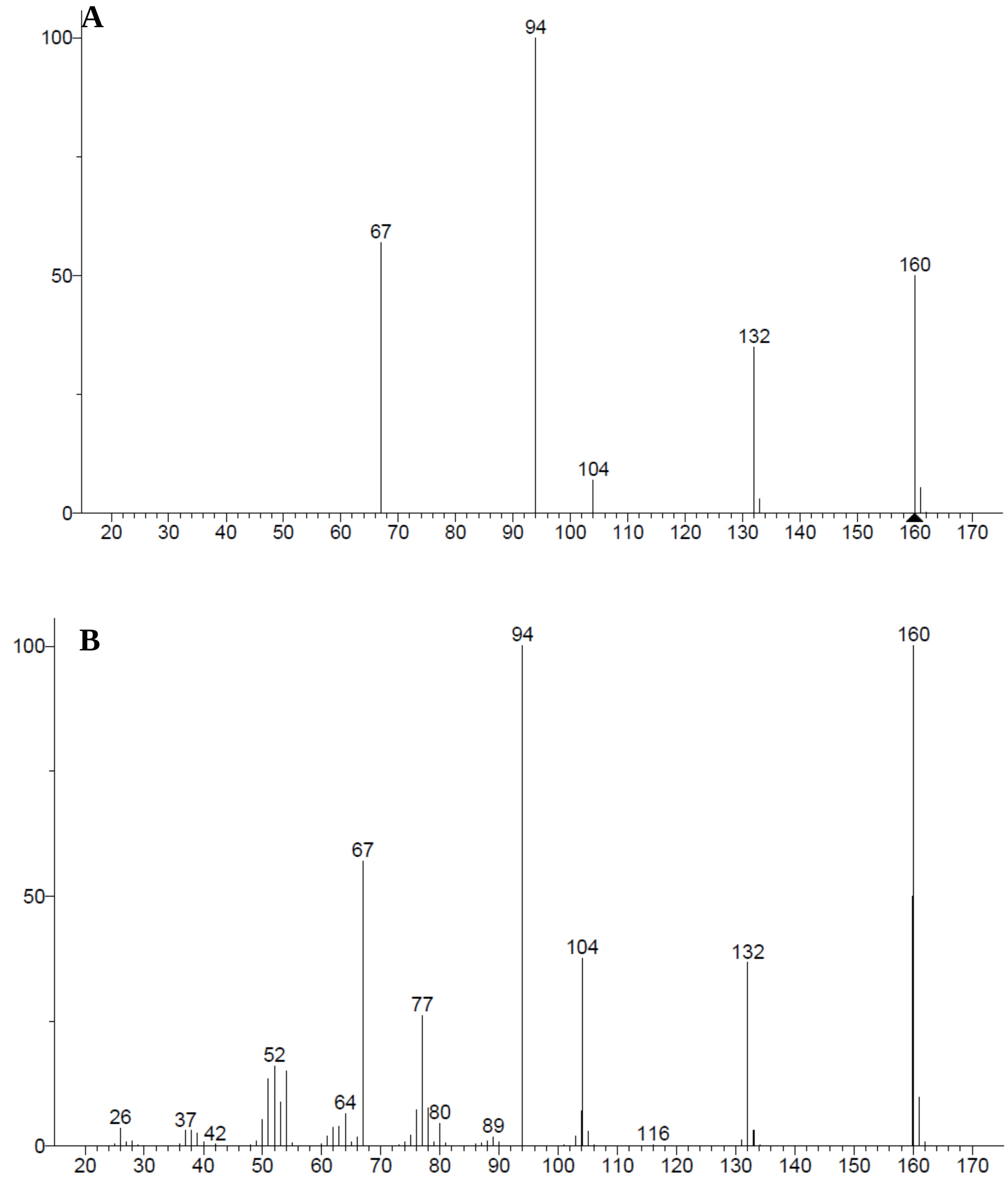

Figure I.5. Mass spectrum of 1.1'-bipyrrole-2.2'.5.5'-tetraone: mass spectrum from Wiley library(A), mass spectrum from total ion current chromatogram (B)

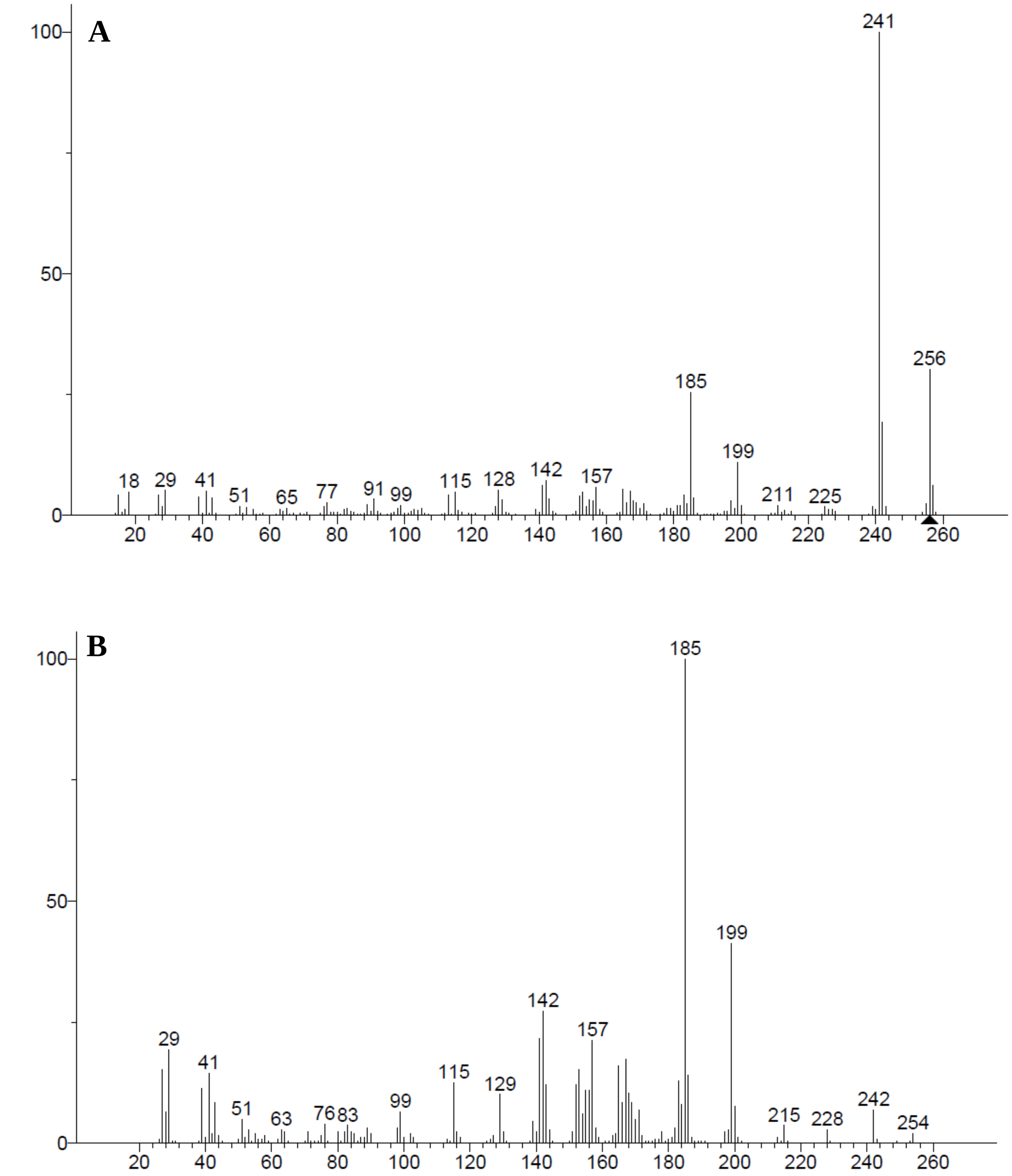

Figure I.6. Mass spectrum of 3,3',4,4'-tetramethyl-1H,1'H-2,2'-bipyrrole-5,5'-dicarboxylic acid: mass spectrum from Wiley library(A), mass spectrum from total ion current chromatogram (B)

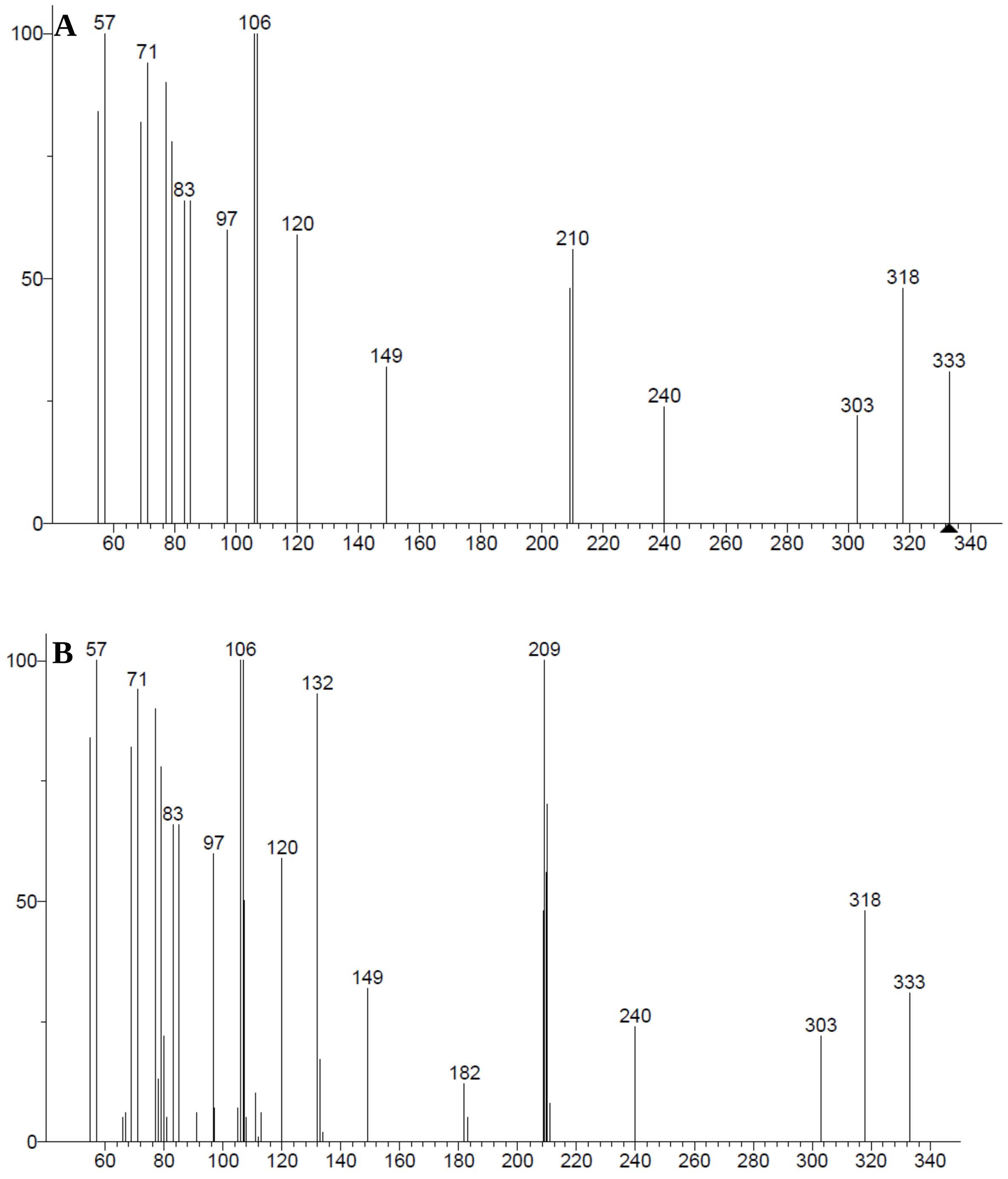

Figure I.7. Mass spectrum of 2H-pyrrol-2-one, 5-[[2-[(4 aminophenyl)methylene]-3,4-dimethyl-2H pyrrol-5-yl]methylene]-3-ethyl-1,5-dihydro-4-methyl-, (Z,Z) (A): mass spectrum from Wiley library, (B) mass spectrum from total ion current chromatogram

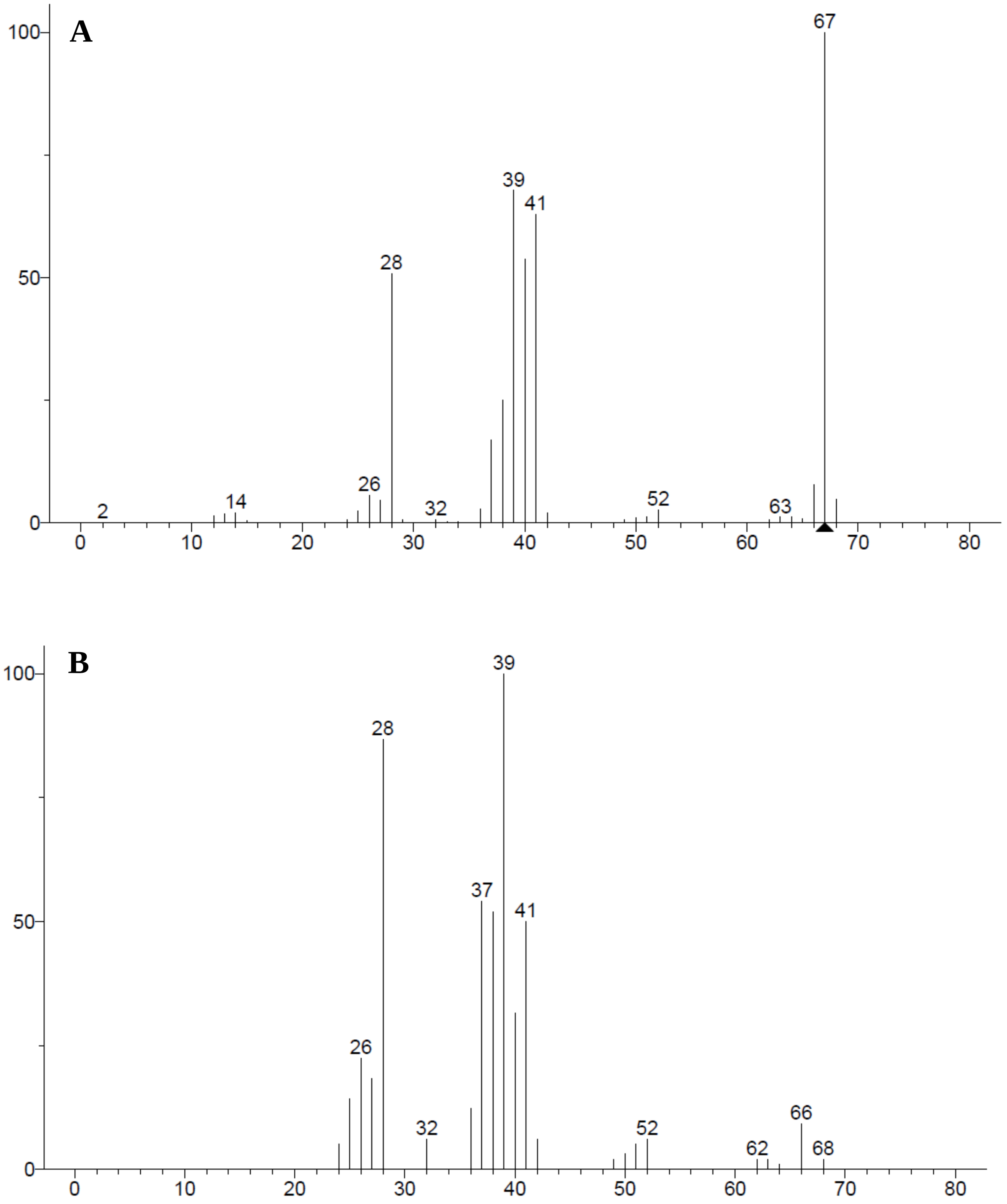

Figure I.8. Mass spectrum of 1H-pyrrole: mass spectrum from Wiley library(A), mass spectrum from total ion current chromatogram (B)

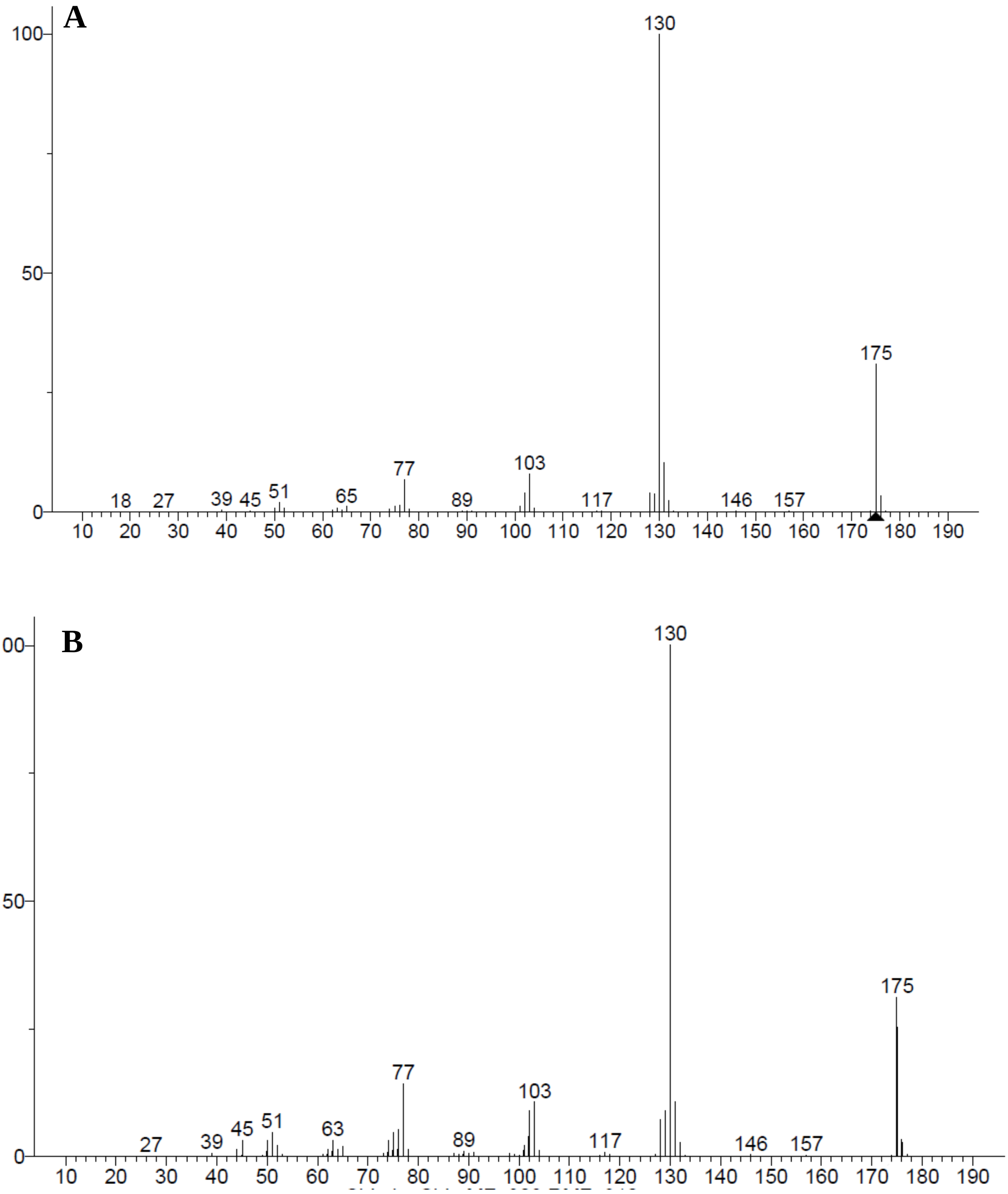

Figure I.9. Mass spectrum of indole acetic acid: mass spectrum from Wiley library(A), mass spectrum from total ion current chromatogram (B)

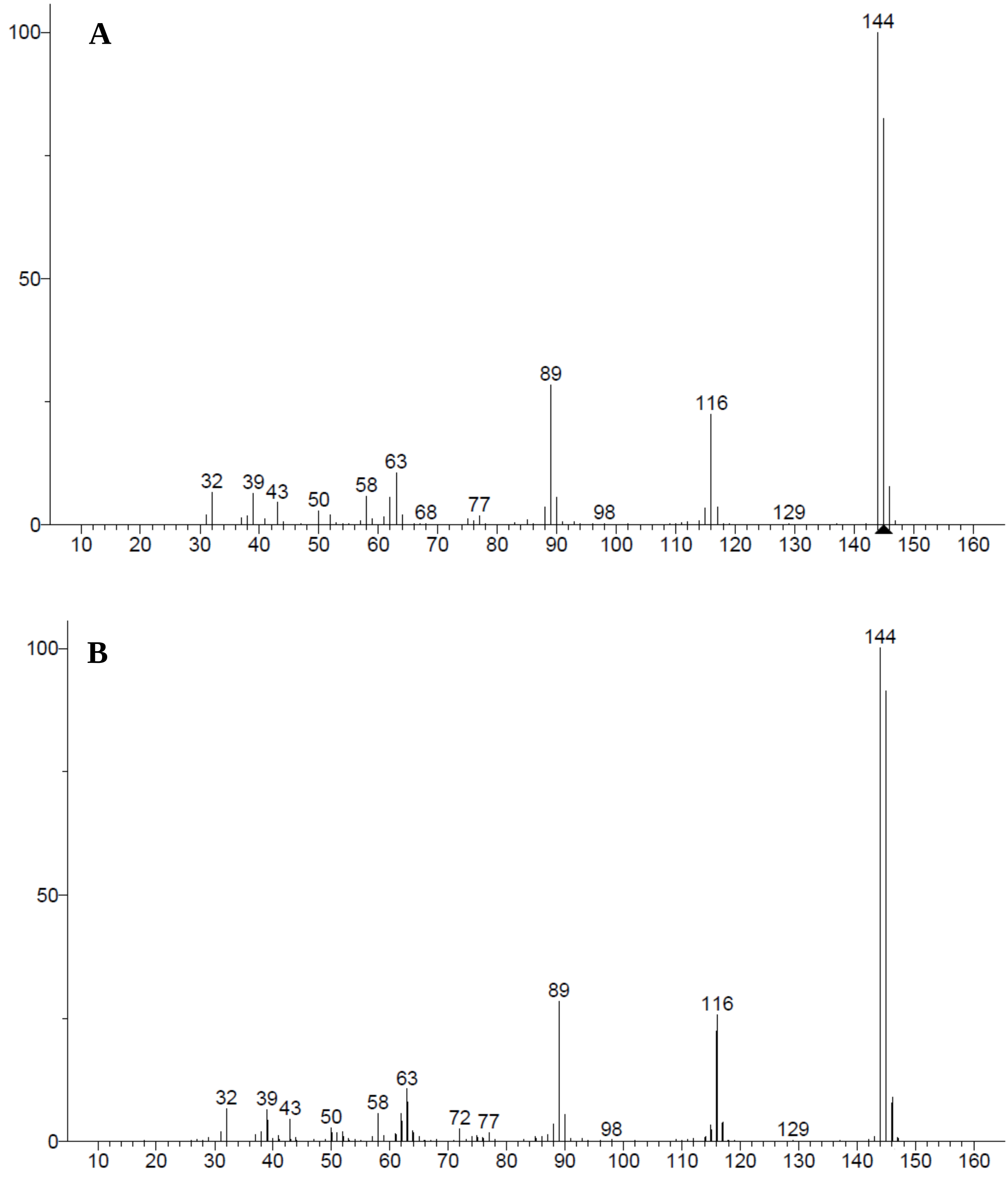

Figure I.10. Mass spectrum of indole carbaldehyde: mass spectrum from Wiley library(A), mass spectrum from total ion current chromatogram (B)

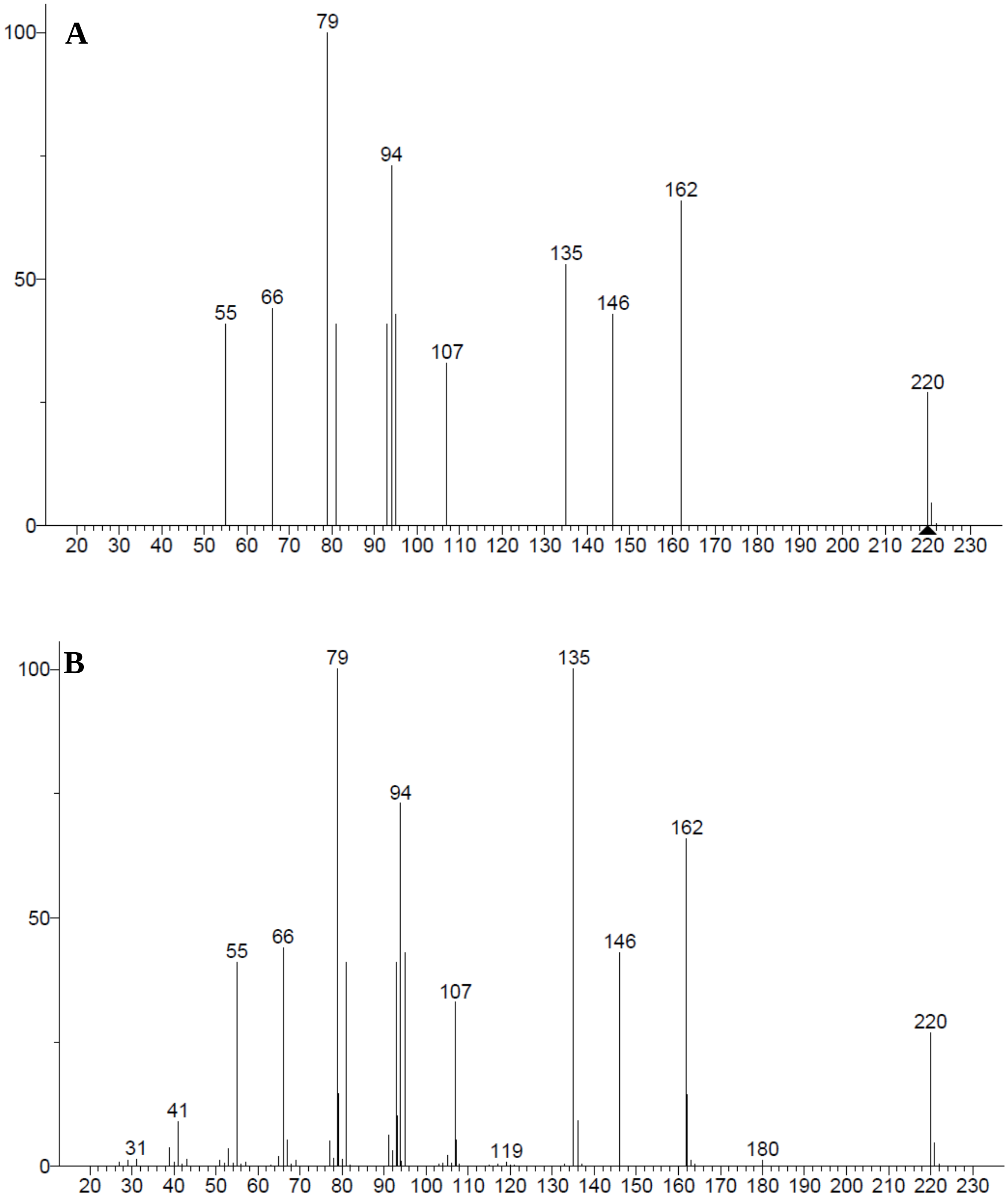

Figure I.11. Mass spectrum of 2-pentanone, 5-(5-methyl-4-cyclononen-1-ylidene): mass spectrum from Wiley library(A), mass spectrum from total ion current chromatogram (B)

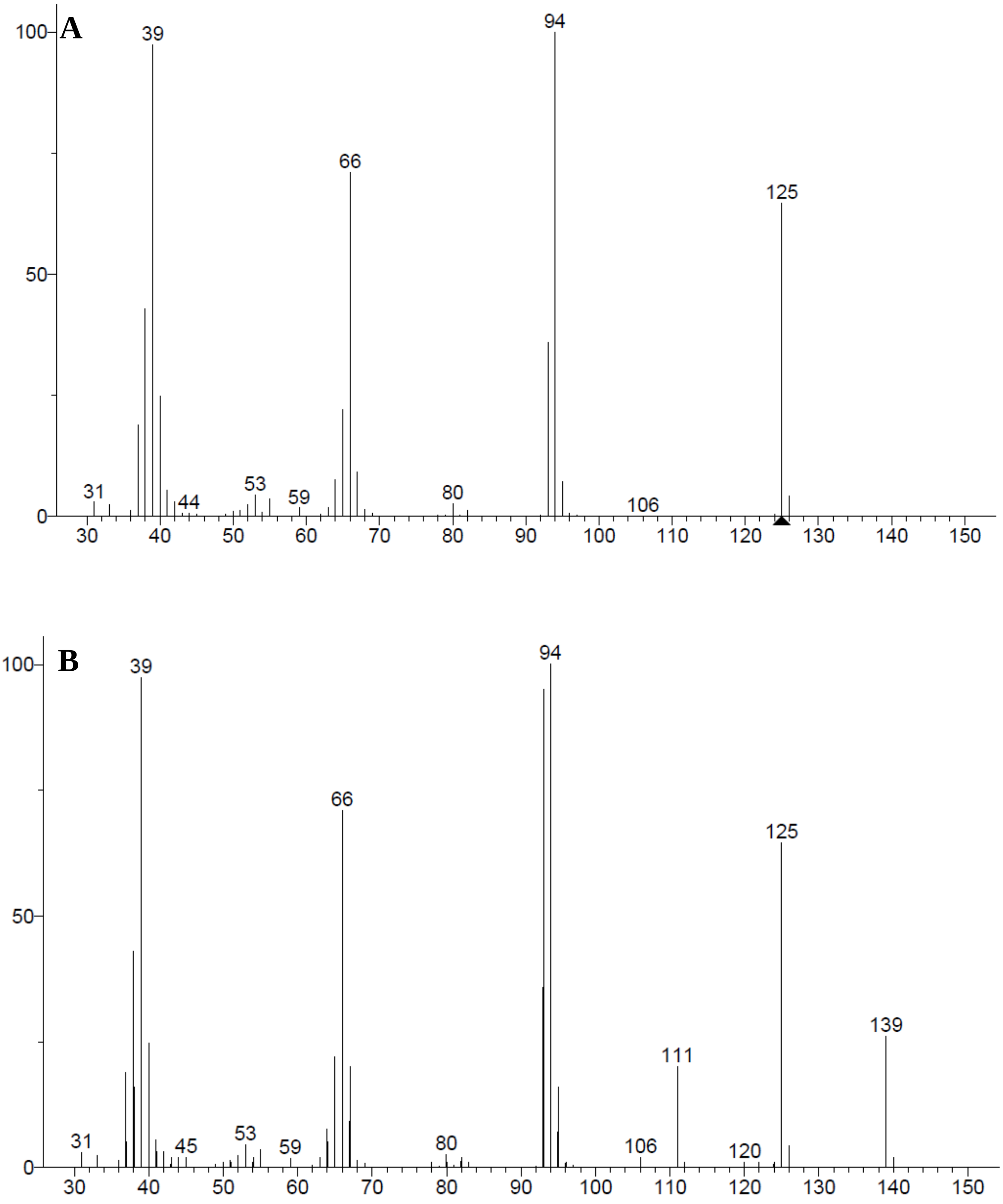

Figure I.12. Mass spectrum of 1H-pyrrole-2-carboxylic acid: mass spectrum from Wiley library(A), mass spectrum from total ion current chromatogram (B)

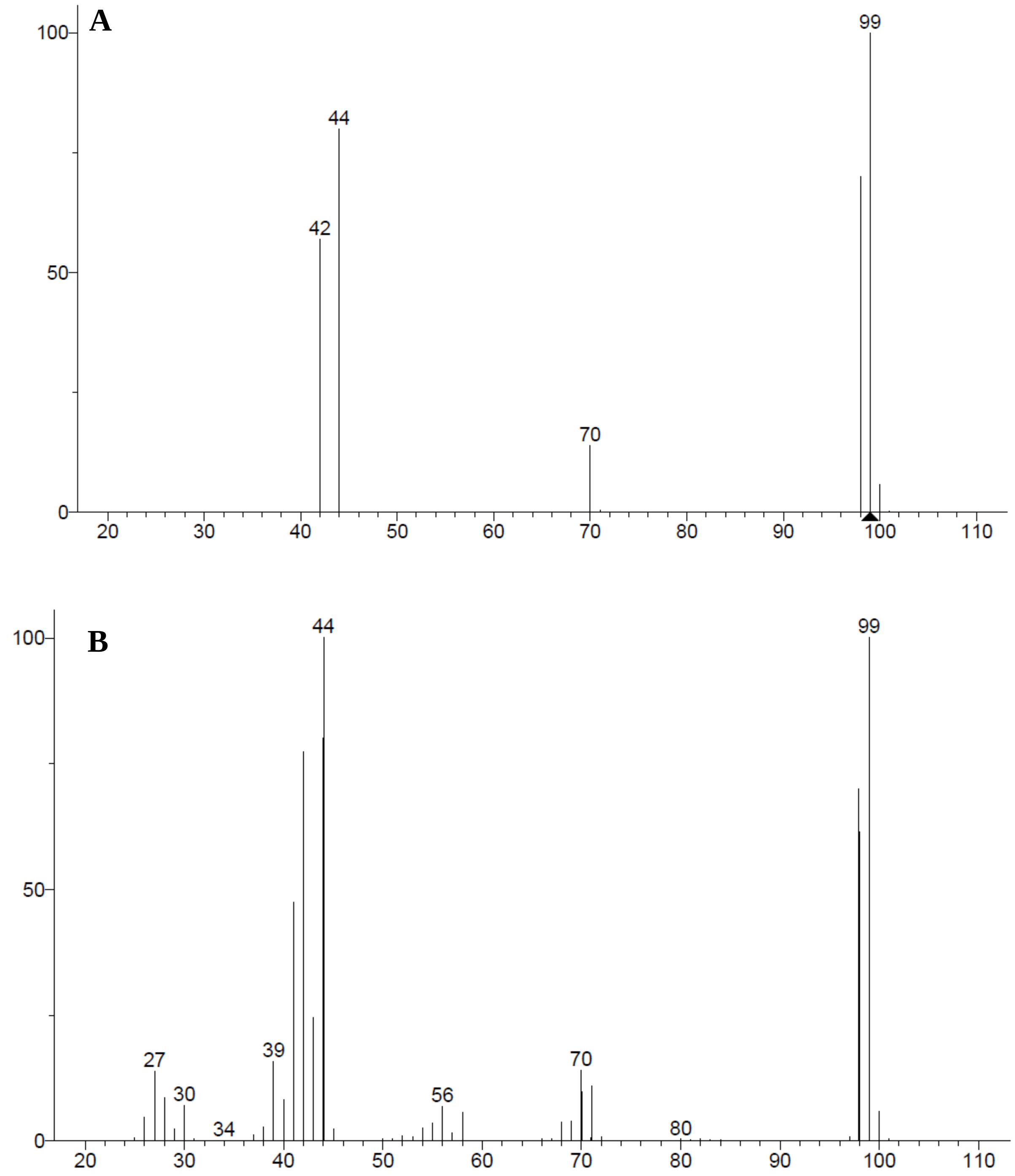

Figure I.13. Mass spectrum of 1-methylpyrroline: mass spectrum from Wiley library(A), mass spectrum from total ion current chromatogram (B)

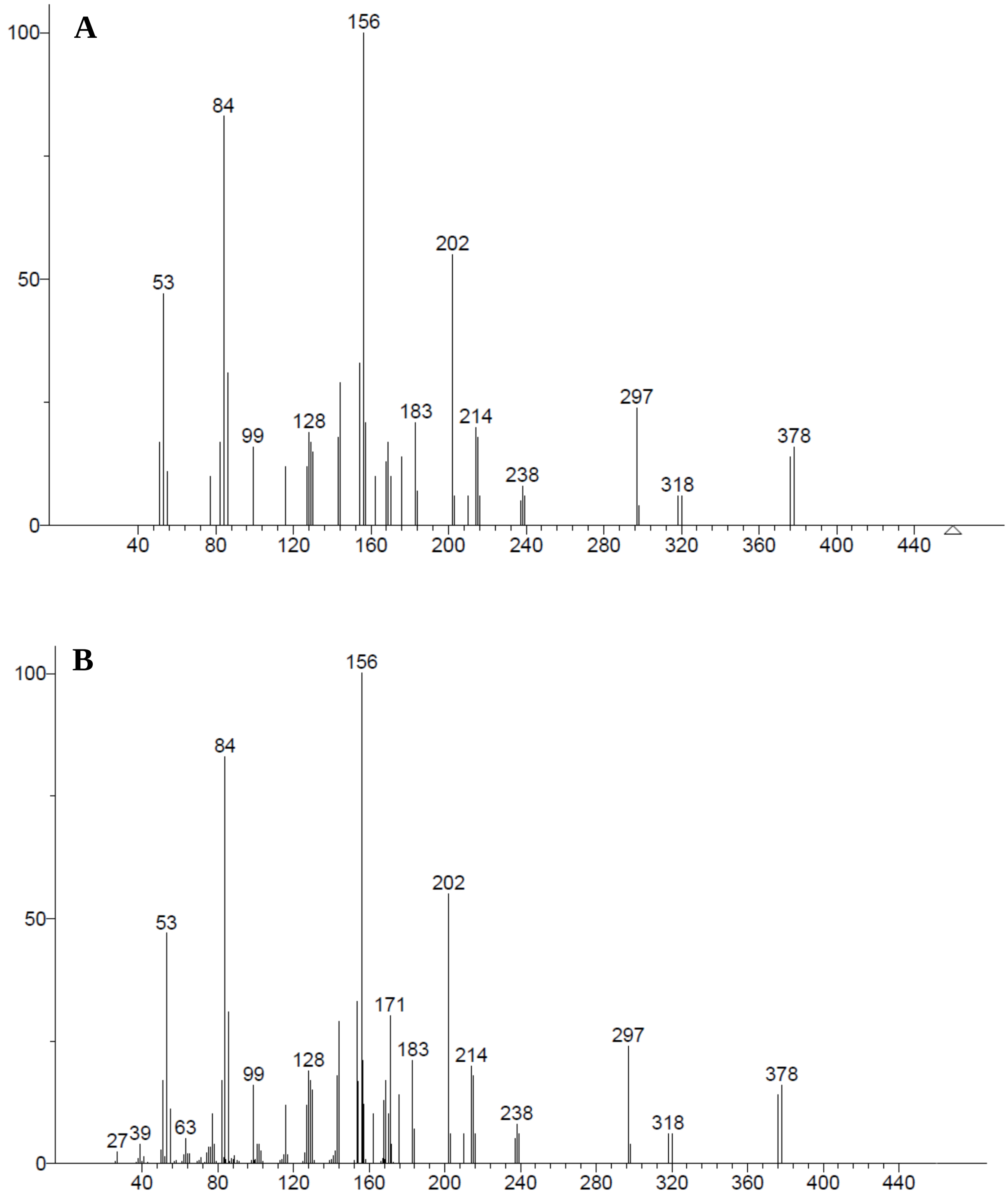

Figure I.14. Mass spectrum of (+-)-(3AR*,4R*,11BR*)-methyl 3-((Z)-2-bromobut-2-en-1-yl)-2,3,3A,4,5,7-hexahydro-4-hydroxy-1H-pyrrolo[2,3-D]carbazole-6-carboxylate: mass spectrum from Wiley library(A), mass spectrum from total ion current chromatogram (B)

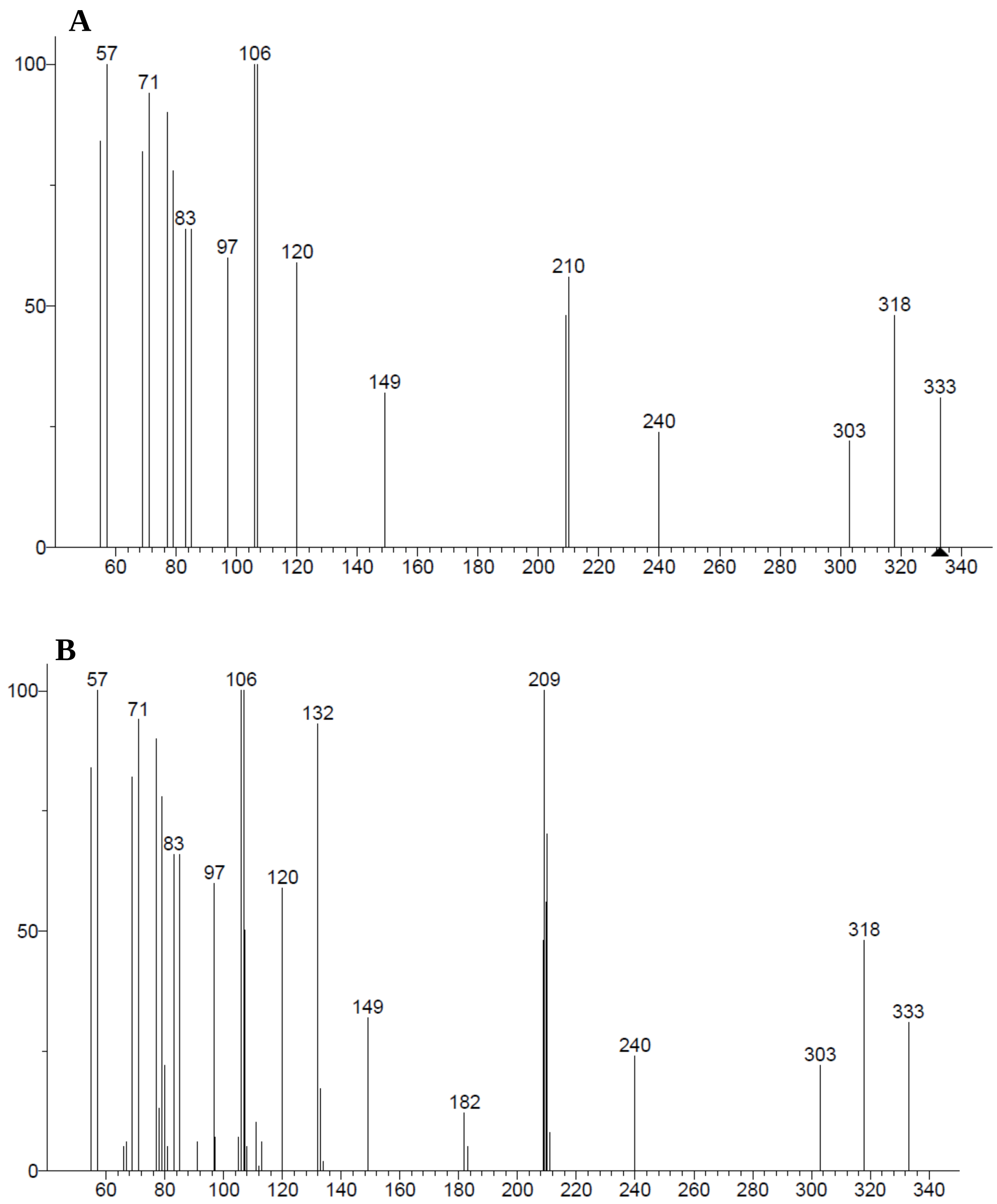

Figure I.15. Mass spectrum of 2H-pyrrol-2-one, 5-[[2-[(4-aminophenyl)methylene]-3,4-dimethyll]methylene]-3-ethyl-1,5-dihydro-4-methyl: mass spectrum from Wiley library(A), mass spectrum from total ion current chromatogram (B)

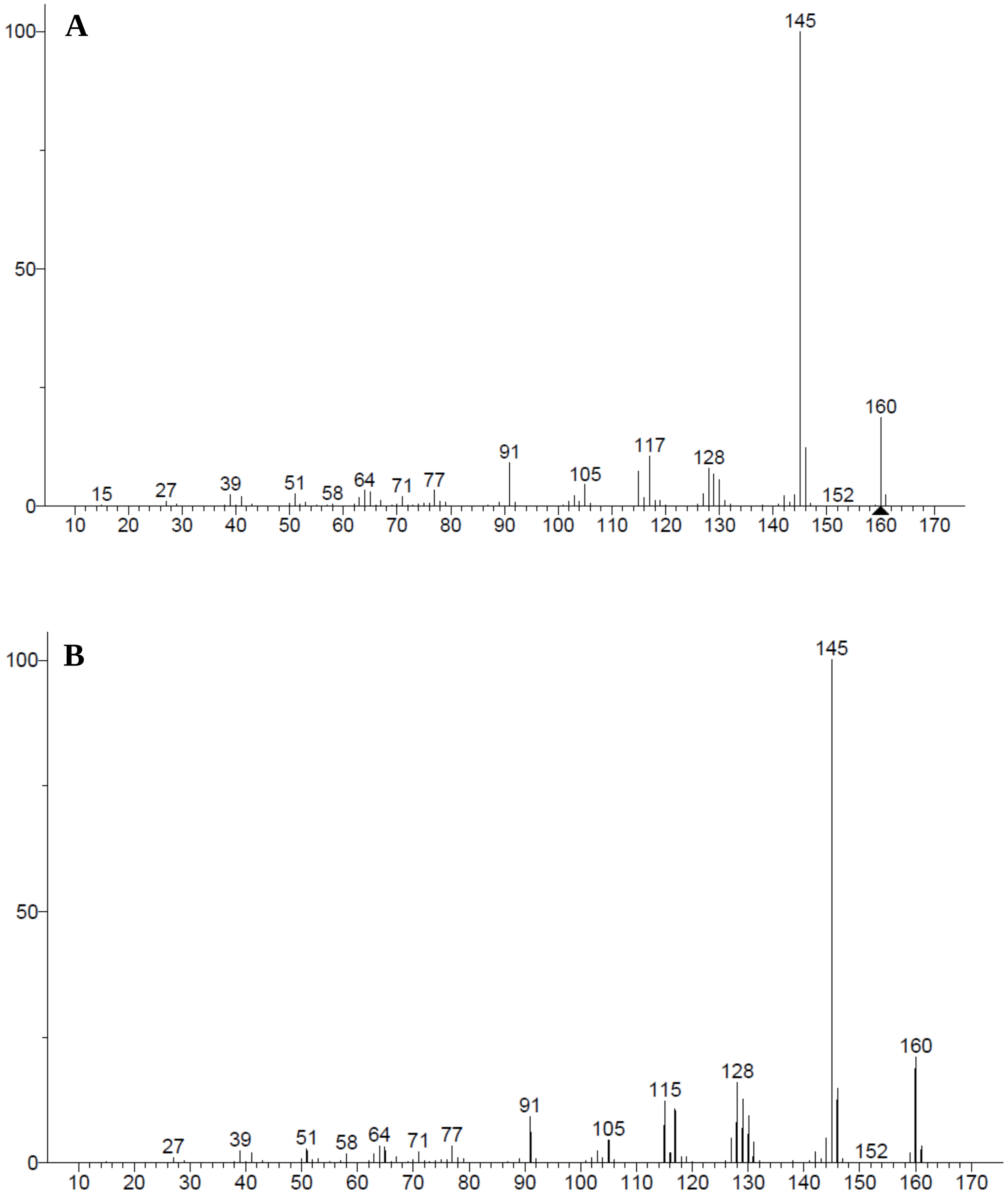

Figure I.16. Mass spectrum of 1H-indene, 2,3-dihydro-1,1,5-trimethyl: mass spectrum from Wiley library(A), mass spectrum from total ion current chromatogram (B)

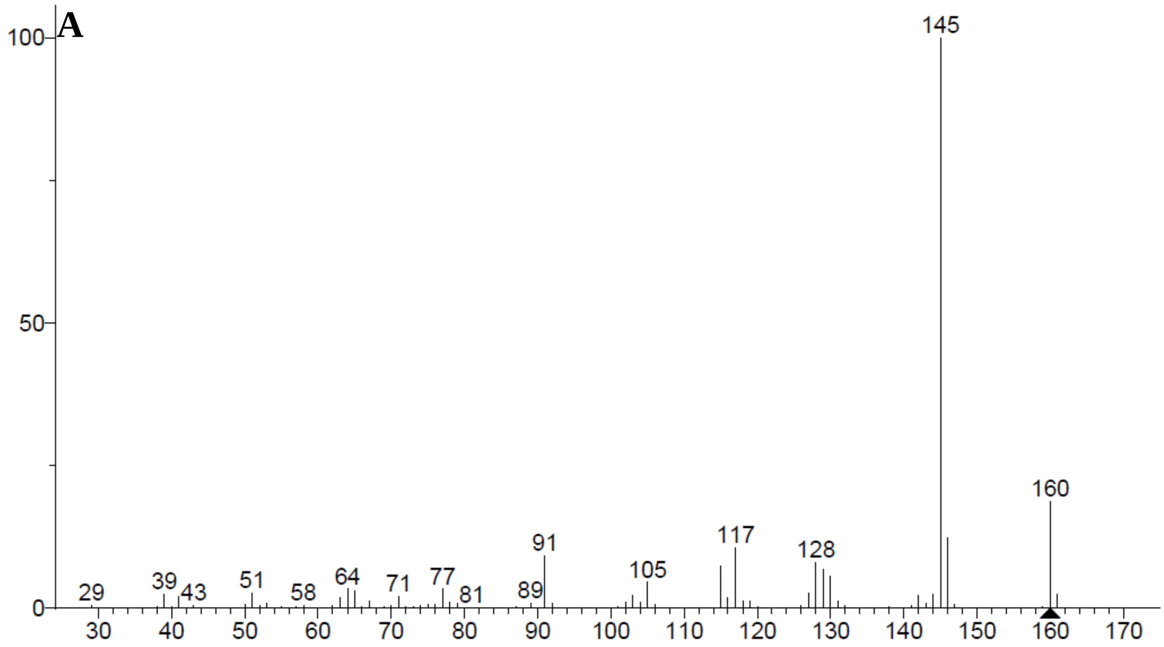

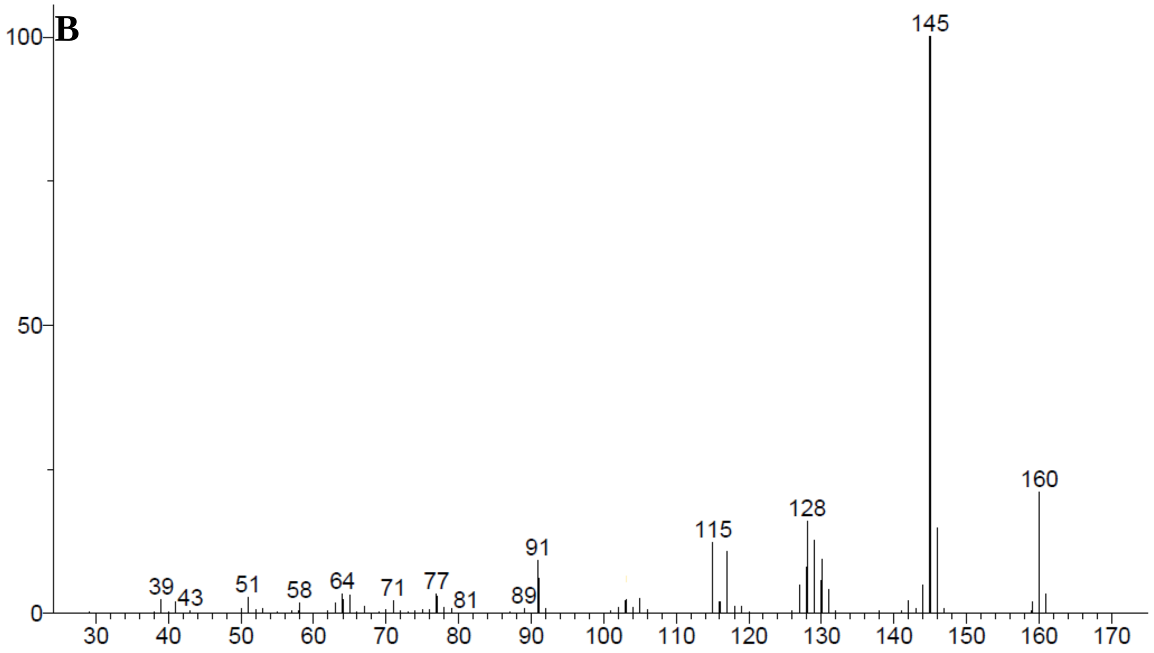

Figure I.17. Mass spectrum of 1H-Indene, 2,3-dihydro-1,4,7-trimethyl: mass spectrum from Wiley library(A), mass spectrum from total ion current chromatogram (B)

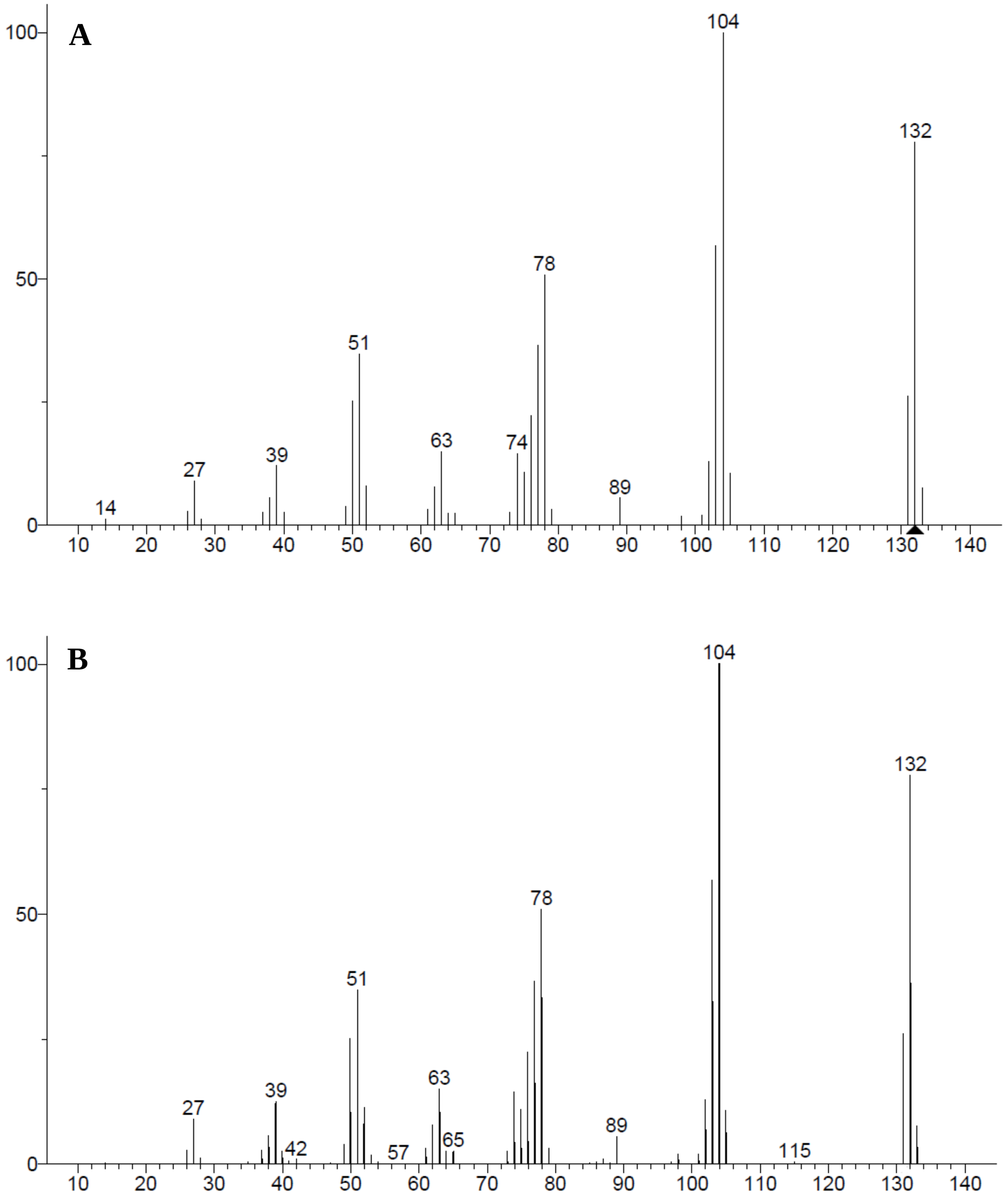

Figure I.18. Mass spectrum of 1H-inden-1-one, 2,3-dihydro-3,3,5,7-tetramethyl: mass spectrum from Wiley library(A), mass spectrum from total ion current chromatogram (B)

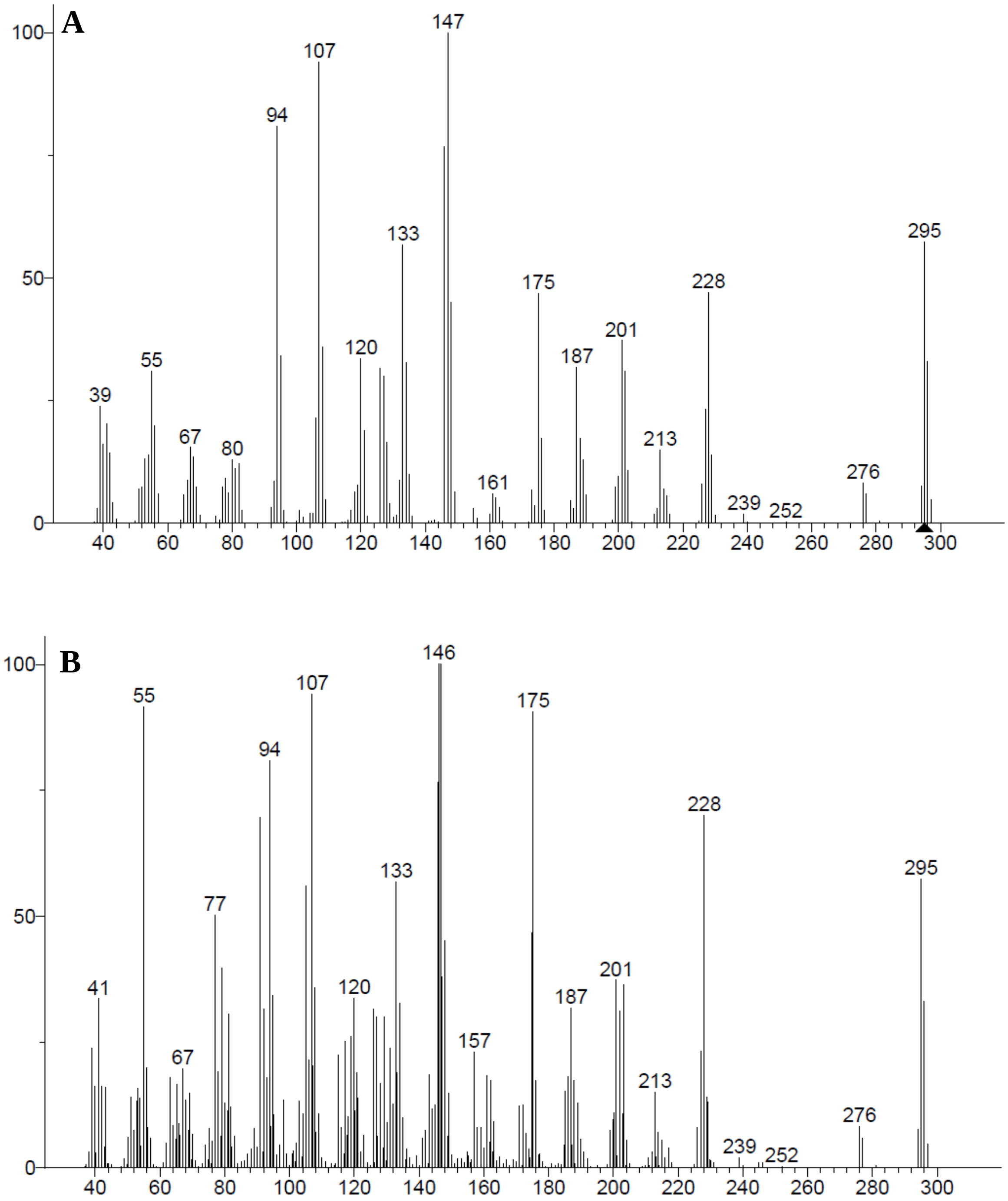

Figure I.19. Mass spectrum of piperidine, 1-(5-trifluoromethyl-2-pyridyl)-4-(1H-pyrrol-1-yl): mass spectrum from Wiley library(A), mass spectrum from total ion current chromatogram (B)

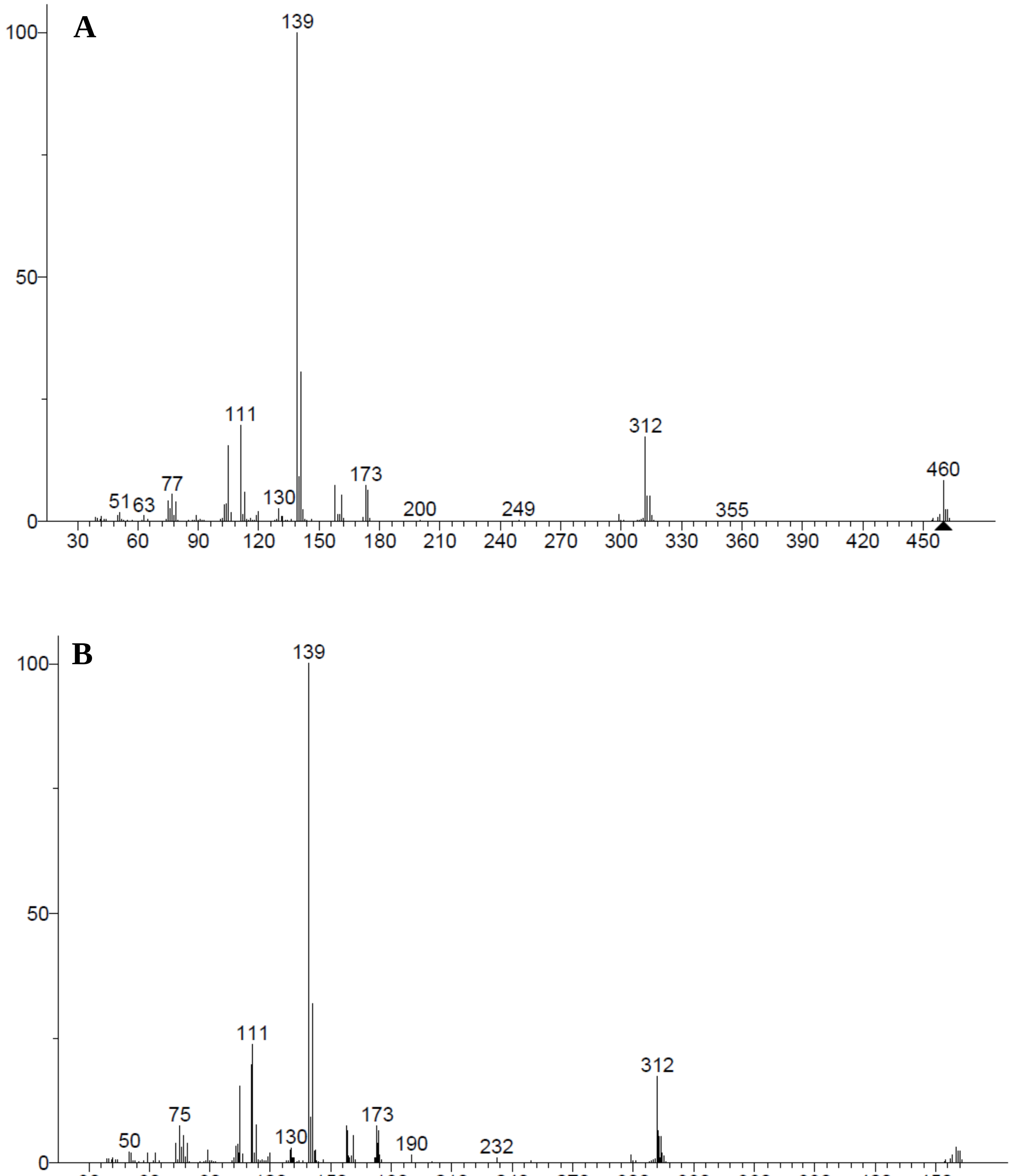

Figure I.20. Mass spectrum of 2-[1-(4-chlorobenzoyl)-5-methoxy-2-methylindol-3-yl]acetic acid: mass spectrum from Wiley library(A), mass spectrum from total ion current chromatogram (B)

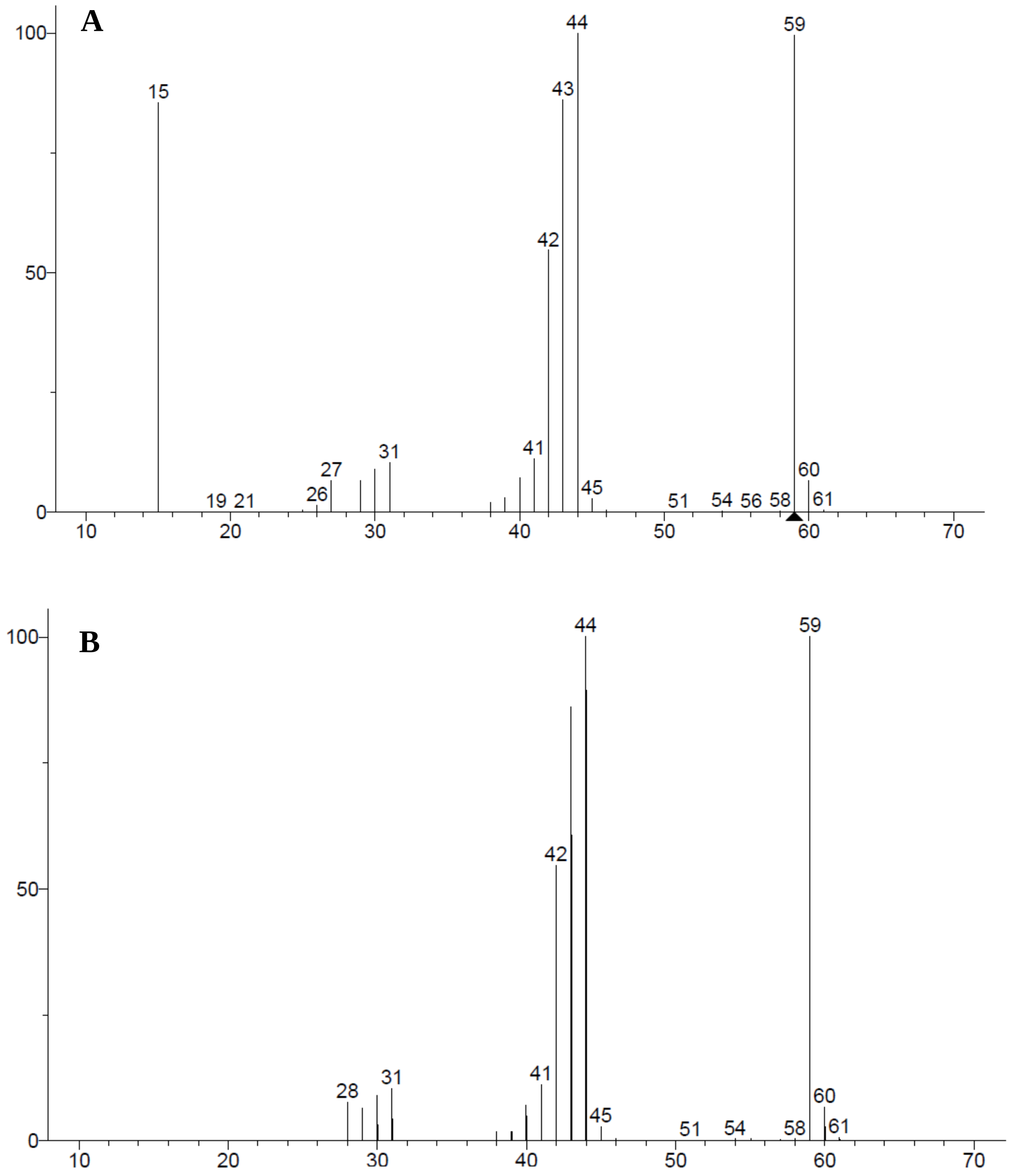

Figure I.21. Mass spectrum of ethanimidic acid: mass spectrum from Wiley library(A), mass spectrum from total ion current chromatogram (B)

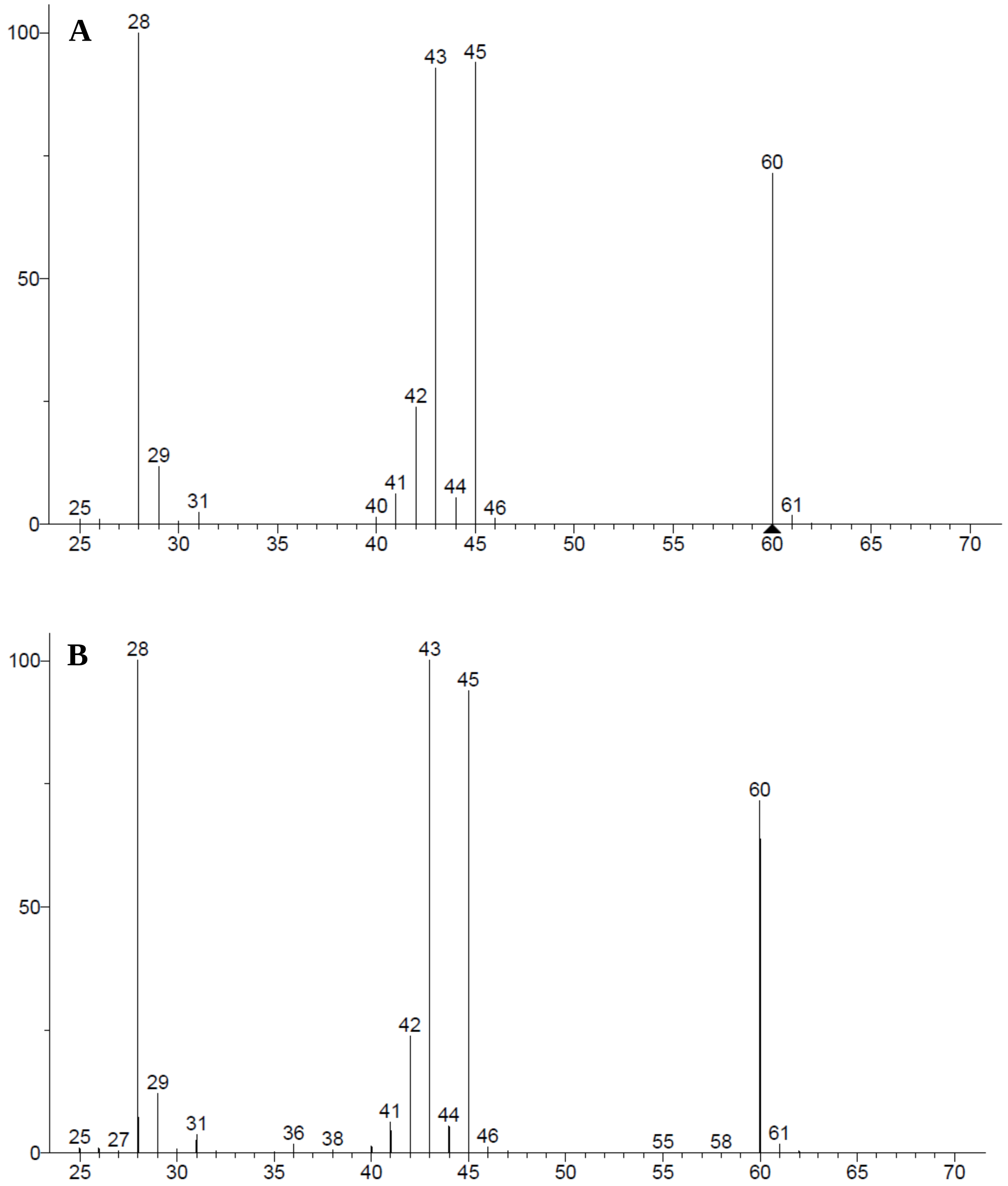

Figure I.22. Mass spectrum of ethanoic acid: mass spectrum from Wiley library(A), mass spectrum from total ion current chromatogram (B)

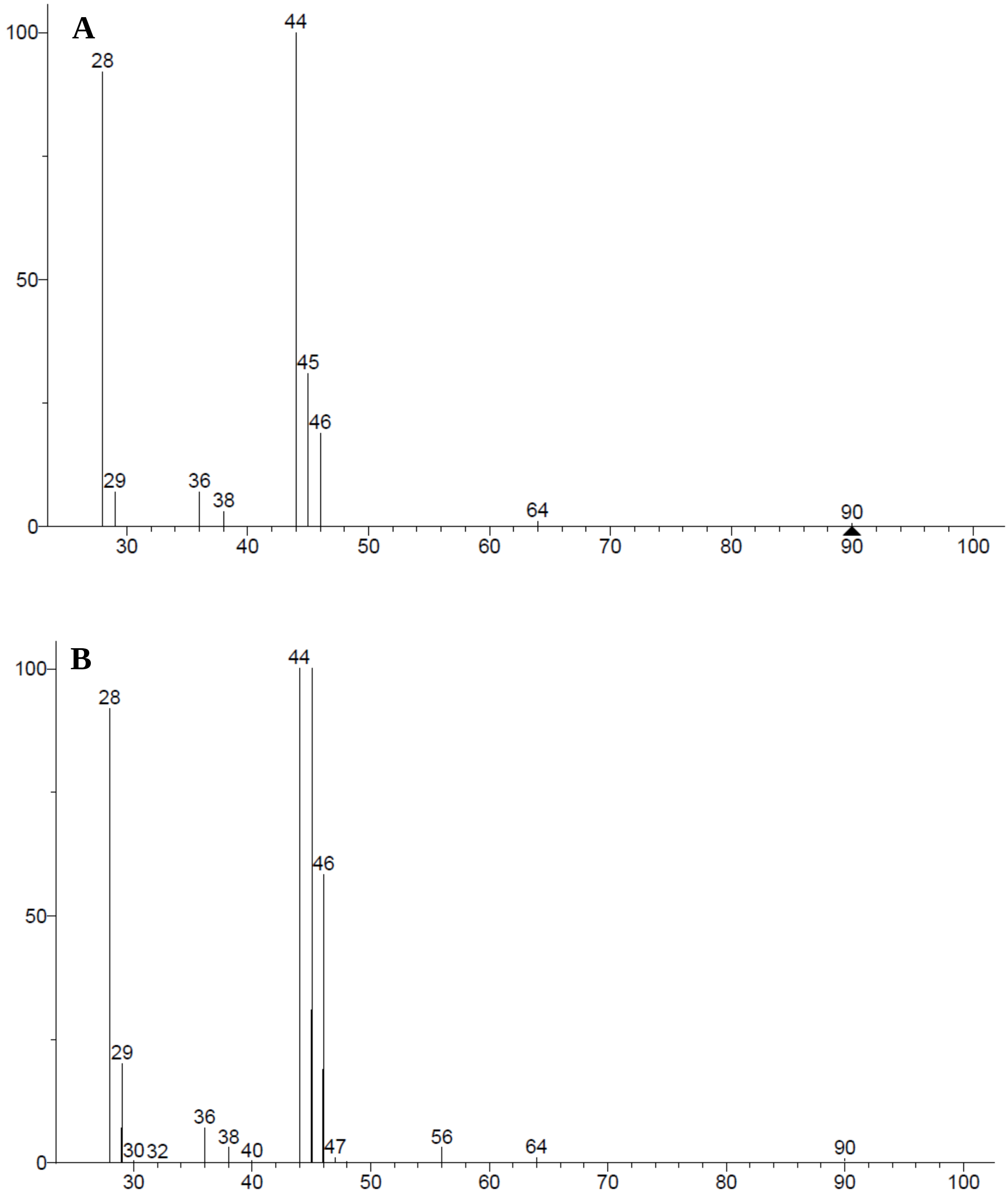

Figure I.23. Mass spectrum of ethanedioic acid: mass spectrum from Wiley library(A), mass spectrum from total ion current chromatogram (B)

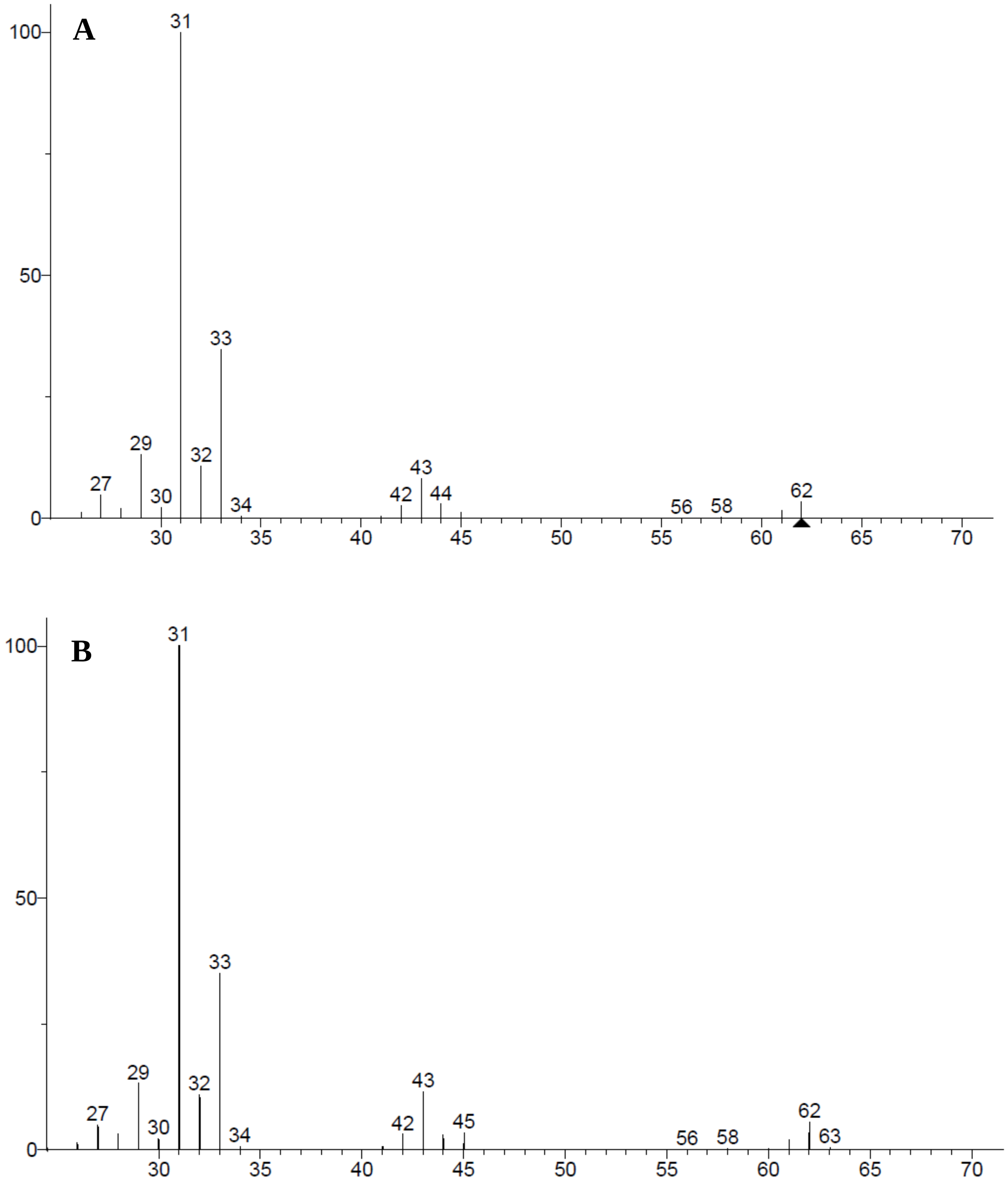

Figure I.24. Mass spectrum of ethane-2,2-diol: mass spectrum from Wiley library(A), mass spectrum from total ion current chromatogram (B)

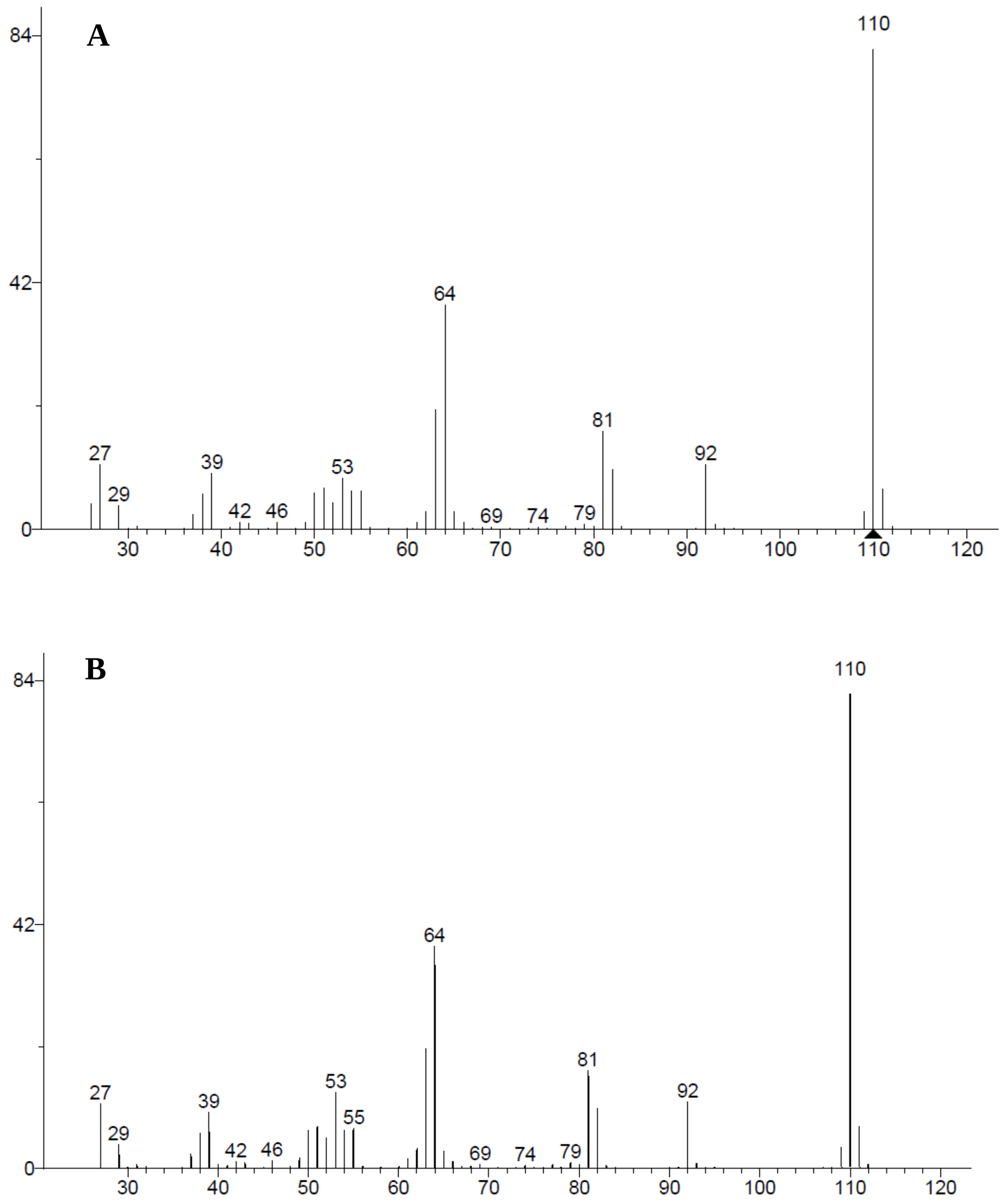

Figure I.25. Mass spectrum of 1,2-benzenediol: mass spectrum from Wiley library(A), mass spectrum from total ion current chromatogram (B)

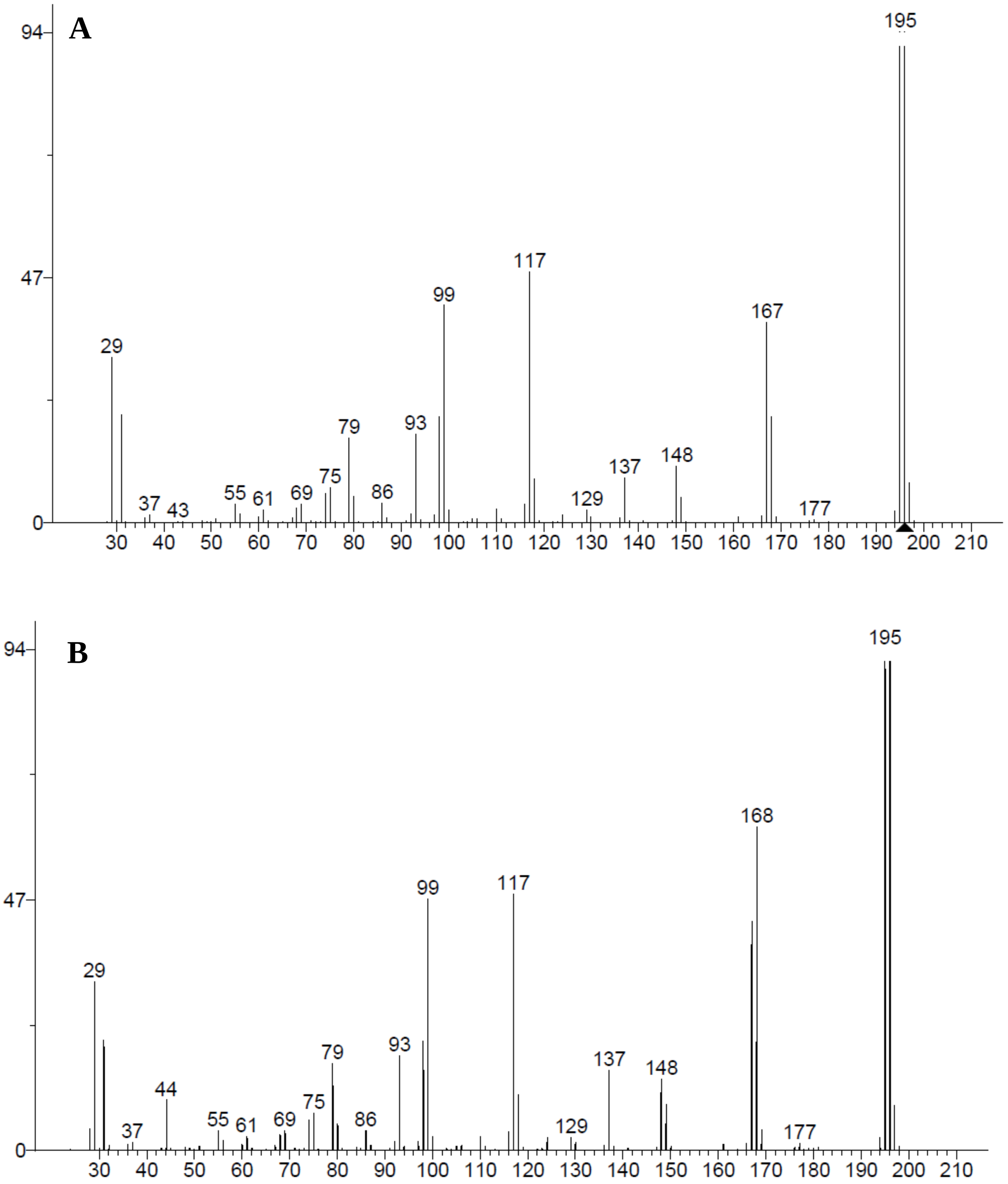

Figure I.26. Mass spectrum of pentafluorobenzaaldehyde: mass spectrum from Wiley library(A), mass spectrum from total ion current chromatogram (B)

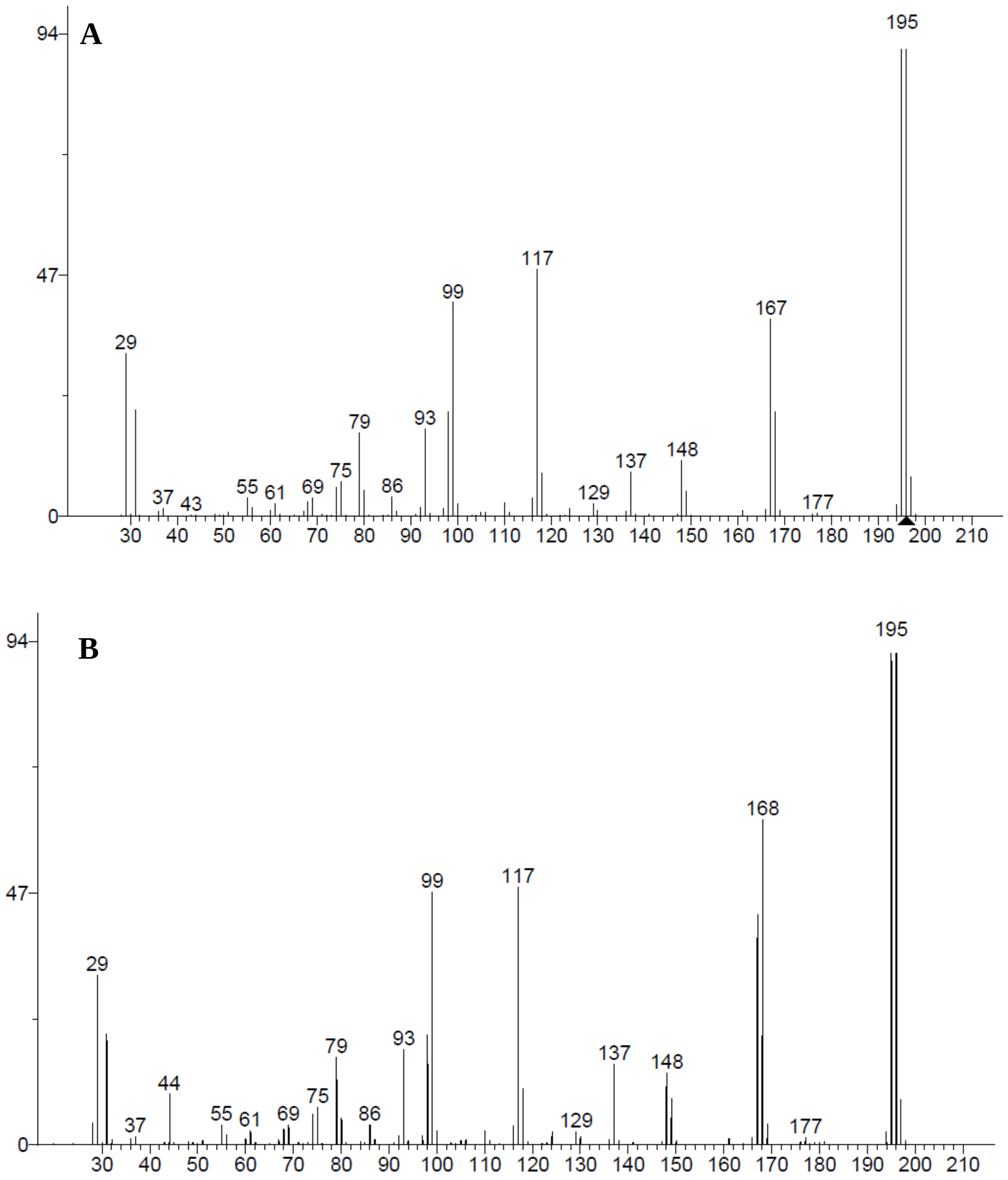

Figure I.27. Mass spectrum of o-tert-butylphenol: mass spectrum from Wiley library(A), mass spectrum from total ion current chromatogram (B)

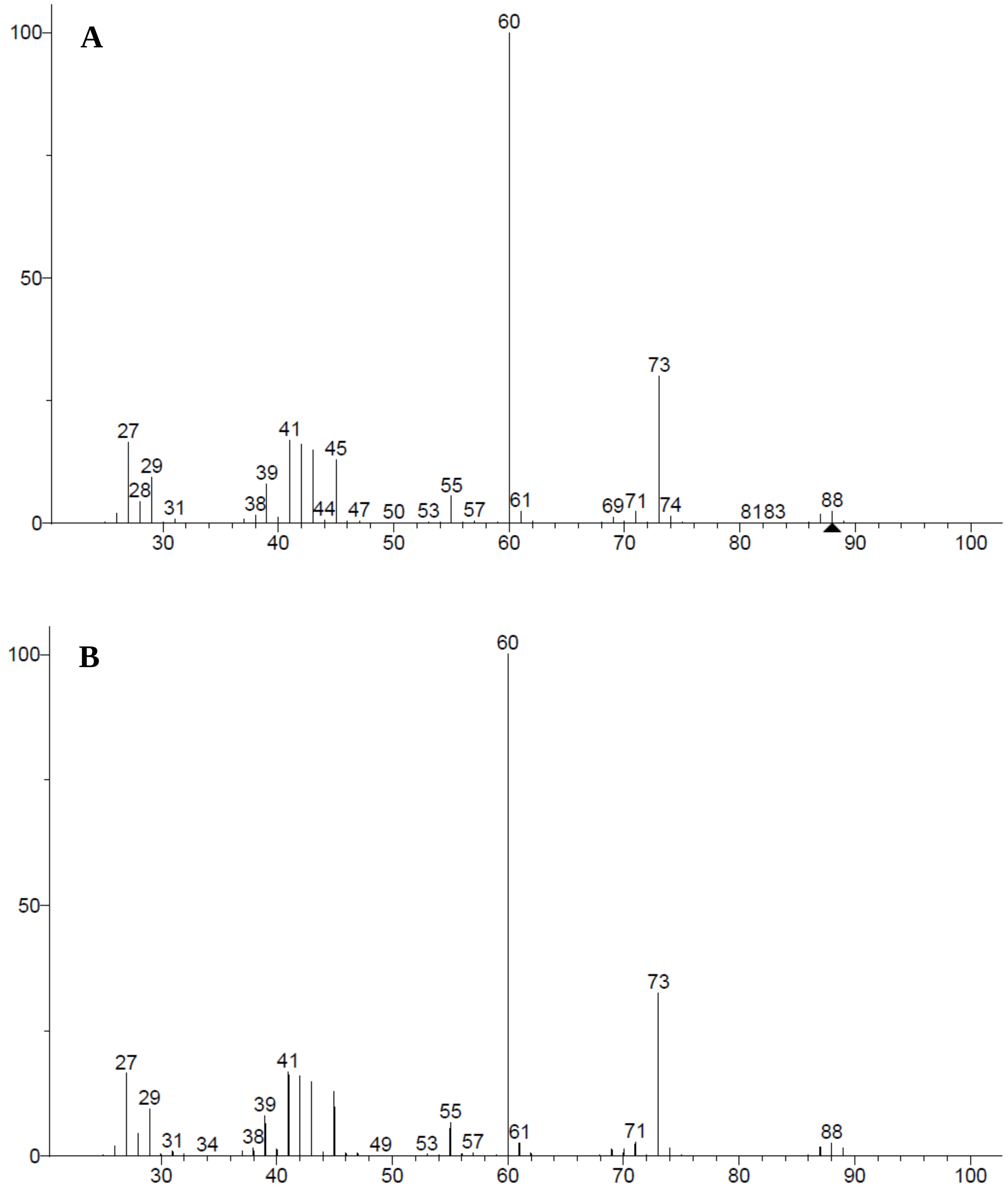

Figure I.28. Mass spectrum of butanoic acid: mass spectrum from Wiley library(A), mass spectrum from total ion current chromatogram (B)

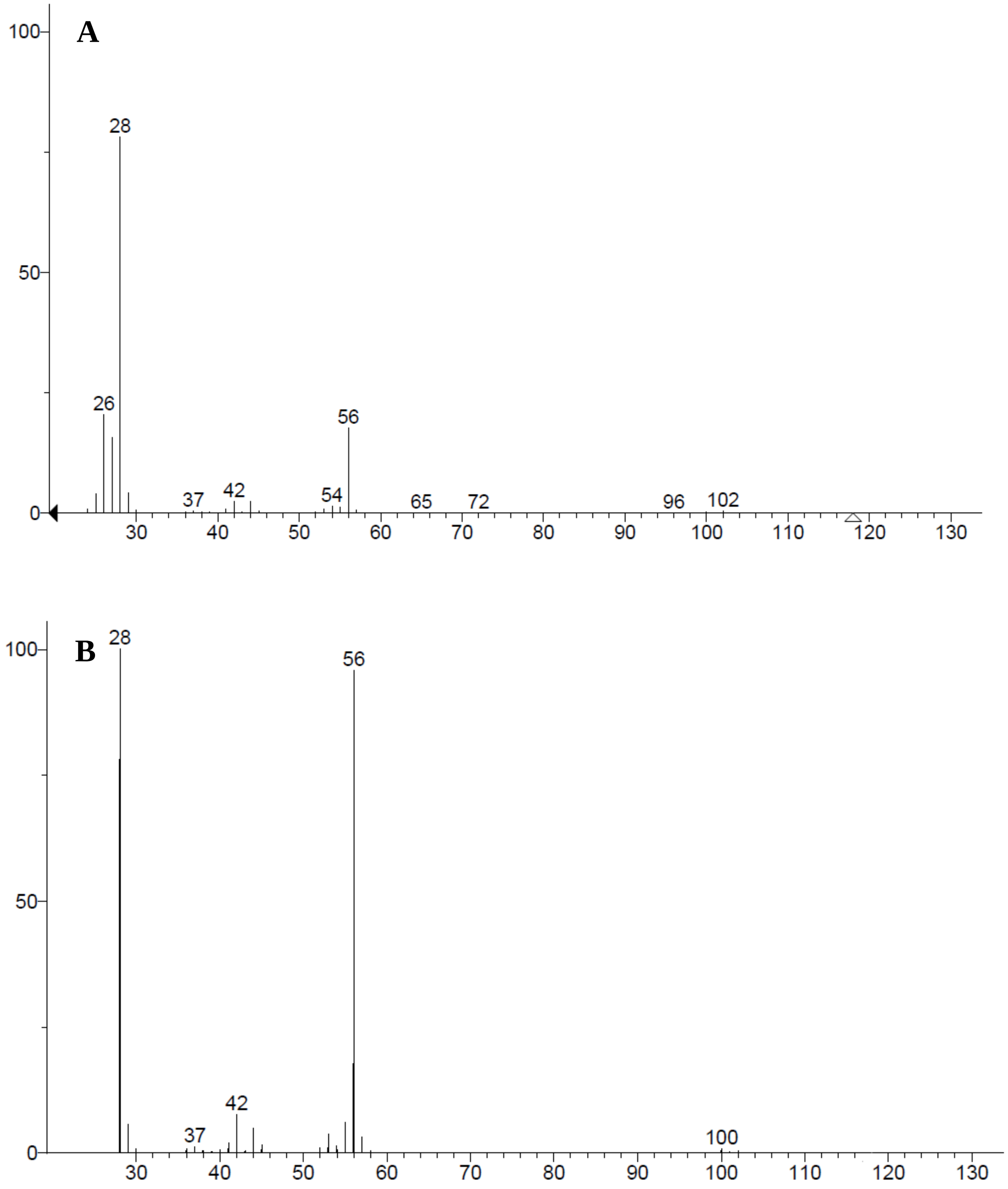

Figure I.29. Mass spectrum of butanedioic acid: mass spectrum from Wiley library(A), mass spectrum from total ion current chromatogram (B)

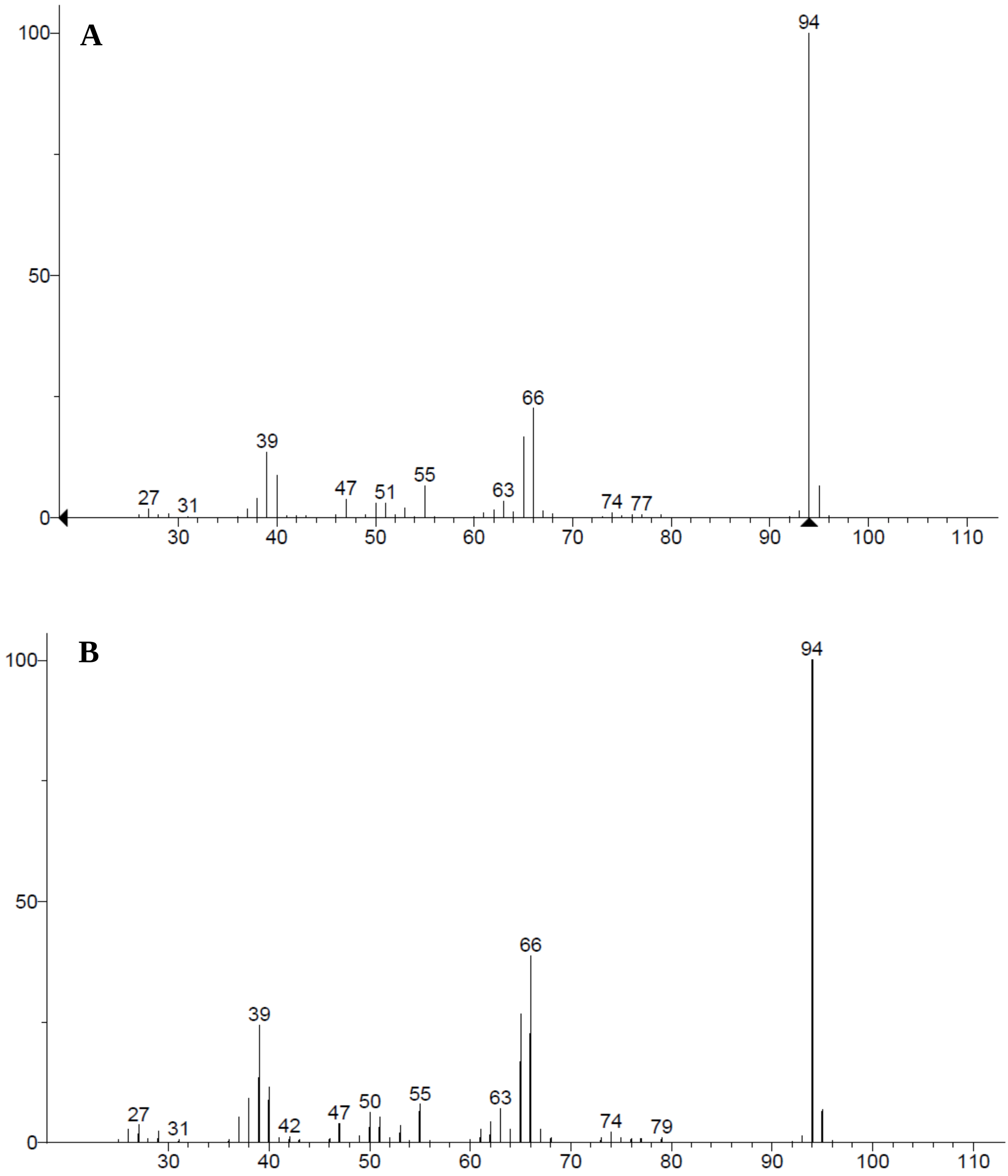

Figure I.30. Mass spectrum of benzenol: mass spectrum from Wiley library(A), mass spectrum from total ion current chromatogram (B)
